# Synthesis and Reactivity of Lewis-Base-Supported Terminal Thorium Imido Metallocene, (η^5^‑C_5_Me_5_)_2_Th=N(*p*‑tolyl)(dmap)_2_

**DOI:** 10.1021/acs.inorgchem.5c01481

**Published:** 2025-06-10

**Authors:** Yi Heng, Enwei Zhou, Dongwei Wang, Wanjian Ding, Guohua Hou, Guofu Zi, Marc D. Walter

**Affiliations:** † Department of Chemistry, 47836 Beijing Normal University, Beijing 100875, China; ‡ Institut für Anorganische und Analytische Chemie, 26527 Technische Universität Braunschweig, Hagenring 30, 38106 Braunschweig, Germany

## Abstract

Treatment of (η^5^-C_5_Me_5_)_2_ThMe_2_ (**1**) with *p*-tolylNH_2_ in toluene, in the presence of 4-dimethylaminopyridine (dmap), affords a Lewis base-supported terminal thorium imido metallocene, (η^5^-C_5_Me_5_)_2_Th=N­(*p*-tolyl)­(dmap)_2_ (**5**), alongside the release of methane. In toluene solution, an equilibrium is established among complex **5**, dmap, and the amido pyridyl complex (η^5^-C_5_Me_5_)_2_Th­[NH­(*p*-tolyl)]­[κ^2^-*C*,*N*-4-(Me_2_N)­C_5_H_3_N] (**5′**), setting the stage for diverse reactivity. Complex **5** may initiate [2 + 2], [2 + 4], [2 + 1], or [2 + 3] cycloadditions with elemental sulfur and selenium, alkynes, carbodiimides, ketones, thio-ketones, isothiocyanates, CS_2_, organic nitriles and isonitriles, as well as organic azides. Moreover, imido moiety can act as a nucleophile toward metal halides, esters, and azidosilanes; and it may promote deprotonation reactions with 1-methylimidazole, 2,6-Me_2_C_5_H_3_NO, Me_3_PO, silanes, amidate PhCONH­(*p*-tolyl), and nitriles (PhCH_2_CN and Ph_2_CHCN). Notably, reaction of **5** with Me_3_SiCHN_2_ forms the bimetallic complex [(η^5^-C_5_Me_5_)_2_Th]_2_(μ-N=NN=CSiMe_3_)_2_ (**44**) with toluene elimination. In contrast, complex **5′** undergoes reactions with elemental selenium and tellurium, PhSiH_2_Cl, organic isonitriles (2,6-Me_2_C_6_H_3_NC, Me_3_CNC, and C_6_H_11_NC), and organic azides (*p*-tolylN_3_ and Ph_3_CN_3_) to afford amido selenido, amido tellurido, chloro pyridyl, amido pyridyl, amido alkenyl, and bis-amido complexes, respectively. Furthermore, a comparison with related terminal imido thorium metallocenes illustrates how substituent effects on the cyclopentadienyl and imido ligands influence the reactivity of these molecules.

## Graphical abstract

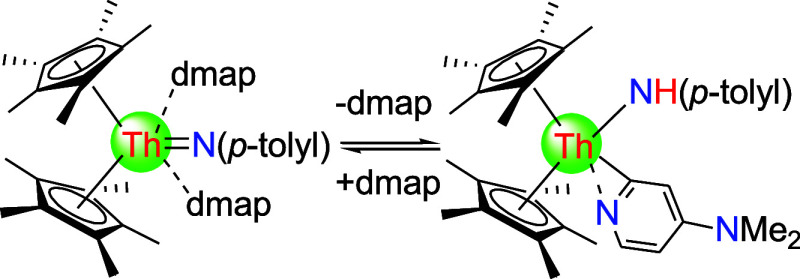

## Introduction

Over the last four decades, terminal imido organoactinide complexes, with their characteristic An=N double bond, have captivated the scientific community due to their promise for advancing small molecule activation and/or catalysis.^1–4^ Although many of these complexes have been synthesized and their structures elucidated, fundamental questions remain concerning how structural nuances dictate reactivity.^2–4^ This inquiry not only drives the broader field of small molecule activation using organoactinide species^5^ but also delves into the foundational aspects of actinide bonding, particularly the extent of covalency and the interplay between the 6d and 5f orbitals.^6^ In our long-standing studies on thorium and uranium complexes that feature multiple bonds with actinides,^7^ we have also reported terminal imido thorium complexes, including a notable example: the base-free terminal thorium imido metallocene, [η^5^-1,2,4-(Me_3_C)_3_C_5_H_2_]_2_Th=N­(*p*-tolyl) (Figure S1),^7a,8^ which readily activates a wide range of small molecules such as elemental sulfur and selenium, C–H bonds of pyridine derivatives, N–H bonds of amines, B–H bonds of boranes, Si–H bonds of silanes, Si–Cl bonds of chlorosilanes and last but not least the NN bond in diazoalkanes (Figure S1).^7c,8^ Moreover, it plays a pivotal role as an intermediate in the catalytic hydroamination of internal acetylenes,^7b^ functions as an exceptionally efficient trimerization catalyst for PhCN,^7b^ and serves as a versatile precursor in the synthesis of terminal oxido and sulfido thorium metallocenes [η^5^-1,2,4-(Me_3_C)_3_C_5_H_2_]_2_Th=E (E = O, S) by cycloaddition–elimination reactions with Ph_2_C=E (E = O, S).^7a^ Motivated by the broad reactivity of these systems, we shifted our focus from the bulky 1,2,4-(Me_3_C)_3_C_5_H_2_ ligand to the widely used, less sterically demanding pentamethylcyclopentadienyl (C_5_Me_5_) ligand. This transition allowed us to explore how reduced steric hindrance impacts the reactivity of thorium imido complexes, a decisive factor in organoactinide chemistry.^7^ In this work, we present our findings on the synthesis, structure, and reactivity of the terminal thorium imido metallocene (η^5^-C_5_Me_5_)_2_Th=N­(p-tolyl)­(dmap)_2_ (**5**) and compare its behavior to that of its more sterically congested counterparts.^8^

## Results and Discussion

### Synthesis of (η^5^-C_5_Me_5_)_2_Th=N­(*p*-tolyl)­(dmap)_2_ (**5**)

Treatment of the thorium dimethyl complex (η^5^-C_5_Me_5_)_2_ThMe_2_ (**1**) with 2 equiv of *p*-tolylNH_2_ affords the bis-amido complex (η^5^-C_5_Me_5_)_2_Th­(NH-*p*-tolyl)_2_ (**2**) quantitatively, as depicted in Scheme 1. The molecular structure of **2** is illustrated in Figure 1, with selected bond distances and angles listed in Table 1. Th­(1)–N (1) distance is 2.329(3) Å, and the N(1)–Th(1)–N­(1A) angle measures 103.5(2)°. Intriguingly, whereas prior studies demonstrated imido complex [η^5^-1,2,4-(Me_3_C)_3_C_5_H_2_]_2_Th=N­(*p*-tolyl) formation from the reaction of dimethyl complex [η^5^-1,2,4-(Me_3_C)_3_C_5_H_2_]_2_ThMe_2_ and bis-amido complex [η^5^-1,2,4-(Me_3_C)_3_C_5_H_2_]_2_Th­(NH-*p*-tolyl)_2_,^7a,7b^ the treatment of (η^5^-C_5_Me_5_)_2_ThMe_2_ (**1**) and (η^5^-C_5_Me_5_)_2_Th­(NH-*p*-tolyl)_2_ (**2**) instead produces the μ-imido bridged bimetallic complex [(η^5^-C_5_Me_5_)_2_Th]_2_[μ-N­(*p*-tolyl)]_2_ (**3**) (Scheme 1). This outcome reflects to lower steric hindrance of the C_5_Me_5_ ligand compared to that of the 1,2,4-(Me_3_C)_3_C_5_H_2_ ligand. Moreover, complex **3** can also be synthesized upon reaction of (η^5^-C_5_Me_5_)_2_ThMe_2_ (**1**) with 1 equiv of *p*-tolylNH_2_ (Scheme 1). The molecular structure of **3** is displayed in Figure 2, with selected bond distances and angles provided in Table 1. The Th(1)–N (1) and Th(1)–N­(1A) distances are 2.271(5) and 2.401(5) Å, respectively, while the N(1)–Th(1)–N­(1A) angle is 71.4(2)°. In a further reaction, treating (η^5^-C_5_Me_5_)_2_ThMe_2_ (**1**) with 1 equiv of *p*-tolylNH_2_ in the presence of pyridine results in the formation of the amido pyridyl complex (η^5^-C_5_Me_5_)_2_Th­[NH­(*p*-tolyl)]­(κ^2^-*C*,*N*-C_5_H_4_N) (**4**) in quantitative conversion (Scheme 1). We propose that an initial imido pyridine adduct (η^5^-C_5_Me_5_)_2_Th=N­(*p*-tolyl)­(py) is formed, which subsequently undergoes intramolecular deprotonation of the coordinated pyridine in α-position to furnish complex **4** (Scheme 1). The molecular structure of **4** is presented in Figure 3, and selected bond distances and angles are listed in Table 1. The Th(1)–N (1) and Th(1)–N(2) distances measure 2.355(5) and 2.475(6) Å, respectively, while the Th(1)–C(28) distance is 2.454(7) Å. This series of reactions underscores not only the versatile coordination chemistry of thorium complexes but also the delicate interplay of steric effects and reagent choice in directing the formation of distinct products. We then hypothesized that increasing the basicity of the Lewis base could help overcome these challenges and promote the formation of the desired terminal imido thorium metallocene. Indeed, the reaction of (η^5^-C_5_Me_5_)_2_ThMe_2_ (**1**) with 1 equiv of *p*-tolylNH_2_ in the presence of 2 equiv of 4-dimethylaminopyridine (dmap) in toluene at room temperature forms the terminal imido complex (η^5^-C_5_Me_5_)_2_Th=N­(*p*-tolyl)­(dmap)_2_ (**5**) in 86% isolated yield (Scheme 1). Moreover, contrary to (η^5^-C_5_Me_5_)_2_Th=N­(mesityl)­(dmap),^7f^ complex **5** crystallizes as an adduct with two equivalents of dmap, most likely reflecting the reduced steric hindrance of its *p*-tolyl group versus the mesityl analogue group. Nevertheless, when complex **1** is treated with 1 equiv of *p*-tolylNH_2_ and 1 equiv of dmap, the reaction affords a complex mixture, and no pure compound can be isolated. This outcome further confirms our earlier conclusion that forming actinide imido metallocenes via methane elimination is highly sensitive to both the basicity of the coordinating Lewis base and the substituent effects on the cyclopentadienyl ring.^1d^ The molecular structure of **5** is shown in Figure 4, with selected bond distances and angles summarized in Table 1. The Th–N(2) and Th–N(4) distances, measured at 2.673(3) and 2.634(3) Å, respectively, are consistent with the presence of a dative nitrogen atom coordination. In stark contrast, the significantly shorter Th–N (1) bond of 2.082(3) Å mirrors the bond lengths typically observed in other thorium imido compounds such as [η^5^-1,2,4-(Me_3_C)_3_C_5_H_2_]_2_Th=N­(*p*-tolyl) (2.038(3) Å),^7b^ (η^5^-C_5_Me_5_)_2_Th=N­(mesityl)­(dmap) (2.091(7) Å),^7f^ [η^5^-1,2,4-(Me_3_Si)_3_C_5_H_2_]_2_Th=N­(*p*-tolyl)­(bipy) (2.073(6) Å) and [η^5^-1,3-(Me_3_C)_2_C_5_H_3_]_2_Th=N­(dipp)­(dmap) (2.090(7) Å).^7y,7z^ However, in contrast to the imido complex (η^5^-C_5_Me_5_)_2_Th=N­(mesityl)­(dmap),^7f^ while imido **5** is isolable as a solid, only the amido pyridyl complex (η^5^-C_5_Me_5_)_2_Th­[NH­(*p*-tolyl)]­[κ^2^-*C*,*N*-4-(Me_2_N)­C_5_H_3_N] (**5′**) and dmap are observed by ^1^H NMR spectroscopy in C_7_D_8_ solution over the temperature range of 20–100 °C. This observation suggests that the equilibrium lies toward the formation of **5′** and dmap, a phenomenon attributable to the reduced steric hindrance of the *p*-tolyl group compared with the mesityl analogue. Density functional theory (DFT) investigations suggest that the formation of **5′** begins with the transfer of an α-H atom from dmap to the imido Th=N­(*p*-tolyl) moiety via transition state **TS5a**, forming intermediate **INT5** (Figure 5). In the following step, dmap dissociates from **INT5** through the transition state **TS5b**, yielding product **5′**. The conversion of **5** to **5′** + dmap is slightly exergonic (Δ*G*(298 K) = −1.6 kcal/mol) and encounters an overall barrier of Δ*G* ^‡^(298 K) = 21.6 kcal/mol. This energy profile suggests that an equilibrium between **5** and **5′** + dmap (Scheme 2) exists in solution and favors the side of **5′** + dmap, consistent with the NMR spectroscopic observations. This observation further implies that complex **5** is poised to exhibit a diverse reactivity pattern in small-molecule activation.

**Table 1:** Selected Distances (Å) and Angles (°) for Compounds **2**–**23**, **25**–**39**, and **41**–**43** ^a^

compound	C(Cp)-Th^b^	C(Cp)-Th^c^	Cp(cent)-Th	Th-X	Cp(cent)-Th-Cp(cent)	X-Th-X/Y
**2**	2.813(4)	2.797(4)–2.826(4)	2.539(4), 2.539(4)	N (1) 2.329(3), N(1A) 2.329(3)	133.3(2)	103.5(2)
**3**	Th (1) 2.909(17)	Th (1) 2.859(6)–3.002(5)	Th (1) 2.641(5), 2.653(5)	Th (1) N (1) 2.271(5), N(1A) 2.401(5)	Th (1) 117.3(2)	Th (1) 71.4(2)^d^
**4**	2.828(7)	2.806(6)–2.862(6)	2.570(6), 2.545(6)	N (1) 2.355(5), N(2) 2.475(6), C(28) 2.454(7)	136.2(2)	31.7(2)^e^
**5**	2.926(14)	2.869(4)–2.995(4)	2.673(4), 2.660(4)	N (1) 2.082(3), N(2) 2.673(3), N(4) 2.634(3)	135.4(1)	164.1(1)^f^
**6**	2.842(12)	2.786(7)–2.900(7)	2.591(9), 2.581(9)	N (1) 2.365(7), N(2) 2.448(7), C(28) 2.485(7)	133.1(2)	31.7(2)^e^
**7**	2.859(12)	2.802(7)–2.900(8)	2.603(7), 2.574(7)	N(2) 2.351(6), O (1) 2.418(5), C(21) 2.598(8)	134.9(2)	62.6(2)^g^
**8**	2.867(9)	2.829(6)–2.908(6)	2.599(6), 2.597(6)	N (1) 2.489(6), N(2) 2.348(5), C(28) 2.700(7)	130.5(2)	60.4(2)^h^
**9**	2.854(7)	2.827(4)–2.886(5)	2.588(4), 2.586(4)	N (1) 2.402(4), N(2) 2.661(4), S (1) 2.886(1)	127.4(1)	68.1(1)^i^
**10**	2.826(2)	2.815(12)–2.836(10)	2.552(10), 2.558(10)	N (1) 2.380(11), S (1) 2.819(4)	128.7(3)	109.8(3)
**11**	2.829(8)	2.794(9)–2.853(9)	2.560(9), 2.568(9)	N (1) 2.351(8), Se (1) 2.927(1)	128.3(3)	113.3(2)
**12**	2.842(11)	2.798(5)–2.881(5)	2.565(5), 2.582(5)	N (1) 2.590(4), N(3) 2.348(4), Se (1) 2.959(1)	134.4(5)	59.2(2)^j^
**13**	2.832(11)	2.778(6)–2.881(6)	2.576(6), 2.556(6)	N (1) 2.333(5), N(2) 2.594(5), Te (1) 3.162(2)	134.8(2)	60.3(2)^k^
**14**	2.818(7)	2.789(10)–2.852(9)	2.550(9), 2.545(9)	N (1) 2.607(6), F (1) 2.182(5), F(2) 2.184(5)	140.7(3)	149.0(2)^l^
**15**	2.888(7)	2.851(10)–2.911(10)	2.621(10), 2.628(10)	Cl (1) 2.732(1), Cl(2) 2.735(1), N (1) 2.746(4), N(3) 2.737(4)	121.4(3)	156.6(1)^m^
**16**	2.826(6)	2.791(6)–2.847(5)	2.564(6), 2.545(6)	N (1) 2.628(5), Br (1) 2.872(1), Br(2) 2.921(1)	133.4(2)	148.1(1)^n^
**17**	2.781(4)	2.767(10)–2.801(9)	2.504(9), 2.513(9)	N (1) 2.407(8), Cl (1) 2.678(3), C(21) 2.428(10)	137.7(3)	32.2(3)^o^
**18**	2.871(19)	2.783(6)–2.936(15)	2.567(6), 2.658(6)	N (1) 2.356(4), N(2) 2.630(4), C(21) 2.514(5)	137.8(6)	69.2(1)^p^
**19**	2.837(12)	2.793(3)–2.884(3)	2.572(3), 2.567(3)	N (1) 2.363(3), N(2) 2.624(3), C(21) 2.488(4)	135.6(1)	68.8(1)^p^
**20**	2.855(9)	2.813(8)–2.891(8)	2.590(7), 2.581(7)	N (1) 2.425(6), N(2) 2.432(6), C(46) 2.455(7)	133.9(2)	32.1(2)^q^
**21**	2.864(13)	2.807(4)–2.912(4)	2.601(4), 2.599(4)	N (1) 2.658(4), N(3) 2.377(4), N(5) 2.373(4)	132.6(1)	57.0(1)^r^
**22**	2.873(13)	2.814(6)–2.921(7)	2.606(7), 2.610(7)	N (1) 2.613(6), N(3) 2.365(5), N(5) 2.344(6)	130.8(2)	57.0(2)^r^
**23**	2.887(15)	2.816(4)–2.949(4)	2.612(4), 2.632(4)	N (1) 2.608(3), N(3) 2.436(3), O (1) 2.206(3)	126.1(1)	58.0(1)^s^
**25**	2.849(9)	2.804(4)–2.880(4)	2.583(4), 2.583(4)	N (1) 2.618(4), S (1) 2.766(1), S(2) 2.764(1)	133.4(1)	65.3(1)^t^
**26**	2.858(9)	2.823(12)–2.907(12)	2.600(12), 2.580(12)	N (1) 2.416(14), N(3) 2.633(7), S (1) 2.780(5)	131.5(3)	61.1(3)^u^
**27**	Th (1) 2.861(9)	Th (1) 2.819(5)–2.899(6)	Th (1) 2.611(6), 2.579(6)	Th (1) N (1) 2.597(5), S (1) 2.982(1), S(1A) 3.039(1), S(2A) 2.889(1)	Th (1) 120.8(2)	Th (1) 59.9(1)^v^, 54.8(4)^u^
**28**	2.874(18)	2.813(9)–2.969(11)	2.624(9), 2.598(9)	N (1) 2.674(10), N(3) 2.336(10), O (1) 2.271(8)	128.2(3)	76.7(3)^s^
**29**	Th (1) 2.870(11)	Th (1) 2.824(7)–2.927(7)	Th (1) 2.619(7), 2.593(7)	Th (1) N (1) 2.562(6), O (1) 2.491(5), O(3A) 2.695(5), O(4A) 2.205(5)	Th (1) 124.0(2)	Th (1) 52.1(2)^w^, 63.7(2)^x^
**30**	2.842(11)	2.798(3)–2.886(3)	2.581(3), 2.567(3)	N (1) 2.557(3), N(2) 2.565(3), O (1) 2.462(2), O(2) 2.452(2)	136.3(1)	52.4(1)^w^, 52.3(1)^y^
**31a**	2.859(7)	2.827(9)–2.884(9)	2.588(9), 2.595(9)	N (1) 2.427(11), N(2) 2.276(10), N(3) 2.650(5)	133.8(2)	59.1(3)^p^
**31b**	2.850(13)	2.786(5)–2.888(5)	2.575(5), 2.592(5)	N (1) 2.503(4), N(2) 2.522(5), N(3) 2.512(4), C(35) 2.479(5)	139.5(2)	52.4(2)^p^
**32**	2.819(9)	2.787(6)–2.867(6)	2.559(6), 2.540(6)	N (1) 2.647(5), N(2) 2.463(5), N(4) 2.246(5)	133.2(2)	120.9(2)^z^
**33**	2.811(8)	2.792(3)–2.851(3)	2.554(3), 2.521(3)	N (1) 2.466(3), N(2) 2.440(3), N(3) 2.430(3)	134.8(1)	54.1(1)^p^
**34**	2.816(11)	2.777(13)–2.865(16)	2.541(13), 2.546(13)	N (1) 2.461(10), N(2) 2.468(11), N(3) 2.411(11)	134.1(4)	53.1(3)^p^
**35**	2.865(12)	2.818(6)–2.927(6)	2.611(6), 2.582(6)	N (1) 2.729(5), N(2) 2.513(4), N(3) 2.456(5), C(37) 2.496(6)	128.8(2)	51.4(2)^p^
**36**	2.820(5)	2.796(5)–2.834(5)	2.549(5), 2.552(5)	N (1) 2.334(4), N(2) 2.486(4), C(28) 2.467(5)	135.5(2)	32.2(1)^e^
**37**	2.823(7)	2.791(4)–2.854(4)	2.548(4), 2.557(4)	N (1) 2.356(4), N(2) 2.494(3), C(28) 2.480(4)	132.4(1)	30.1(1)^e^
**38**	2.834(7)	2.801(6)–2.862(6)	2.560(6), 2.568(6)	N (1) 2.368(4), N(2) 2.465(5), C(28) 2.443(5)	133.3(2)	30.7(2)^e^
**39**	2.879(17)	2.811(15)–2.970(15)	2.629(15), 2.599(15)	N (1) 2.350(16), N(2) 2.374(17), N(4) 2.651(8)	131.1(4)	56.9(5)^p^
**41**	2.832(6)	2.811(5)–2.870(5)	2.573(5), 2.554(5)	N (1) 2.344(4), N(2) 2.551(4), N(4) 2.496(4)	134.0(1)	51.8(1)^f^
**42**	2.841(7)	2.809(5)–2.862(5)	2.565(5), 2.583(5)	N (1) 2.304(5), N(2) 2.597(4), N(4) 2.494(4)	138.3(2)	52.1(1)^f^
**43**	2.825(8)	2.798(3)–2.880(3)	2.560(3), 2.548(3)	N (1) 2.318(3), N(4) 2.343(3)	130.4(1)	87.6(1)

a Cp = cyclopentadienyl ring.

b The average value, the value in parentheses, is the standard deviation of the mean.

c Range.

d The angle of N(1)–Th(1)–N­(1A).

e The angle of N(2)–Th(1)–C(28).

f The angle of N(2)–Th(1)–N(4).

g The angle of C(21)–Th(1)–O (1).

h The angle of N(1)–Th(1)–C(28).

i The angle of S(1)–Th(1)–N (1).

j The angle of N(1)–Th(1)–Se (1).

k The angle of N(2)–Th(1)–Te (1).

l The angle of F(1)–Th(1)–F(2).

m The angle of Cl(1)–Th(1)–Cl(2).

n The angle of Br(1)–Th(1)–Br(2).

o The angle of N(1)–Th(1)–C(21).

p The angle of N(1)–Th(1)–N(2).

q The angle of N(2)–Th(1)–C(46).

r The angle of N(3)–Th(1)–N(5).

s The angle of O(1)–Th(1)–N(3).

t The angle of S(1)–Th(1)–S(2).

u The angle of N(1)–Th(1)–S (1).

v The angle of S­(1A)-Th(1)–S­(2A).

w The angle of O(1)–Th(1)–N (1).

x The angle of O­(3A)-Th(1)–O­(4A).

y The angle of O(2)–Th(1)–N(2).

z The angle of N(1)–Th(1)–N(4).

**Figure 1:**
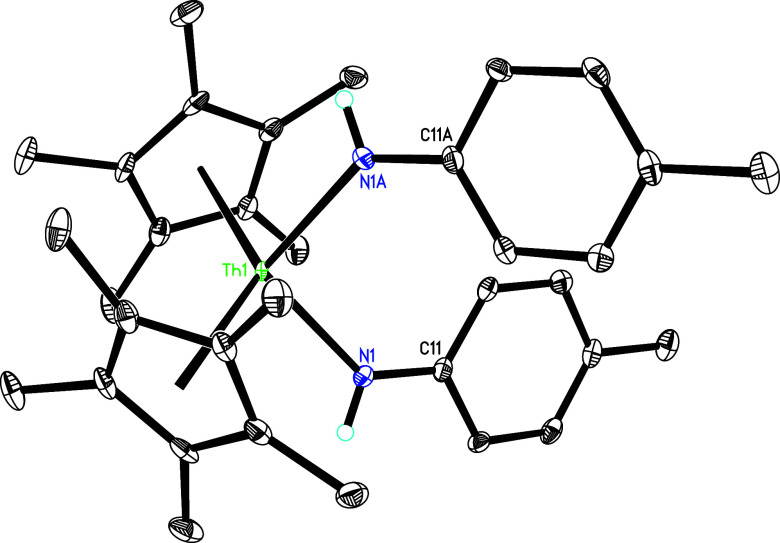
Molecular structure of **2** (thermal ellipsoids drawn at the 35% probability level).

**Scheme 1:**
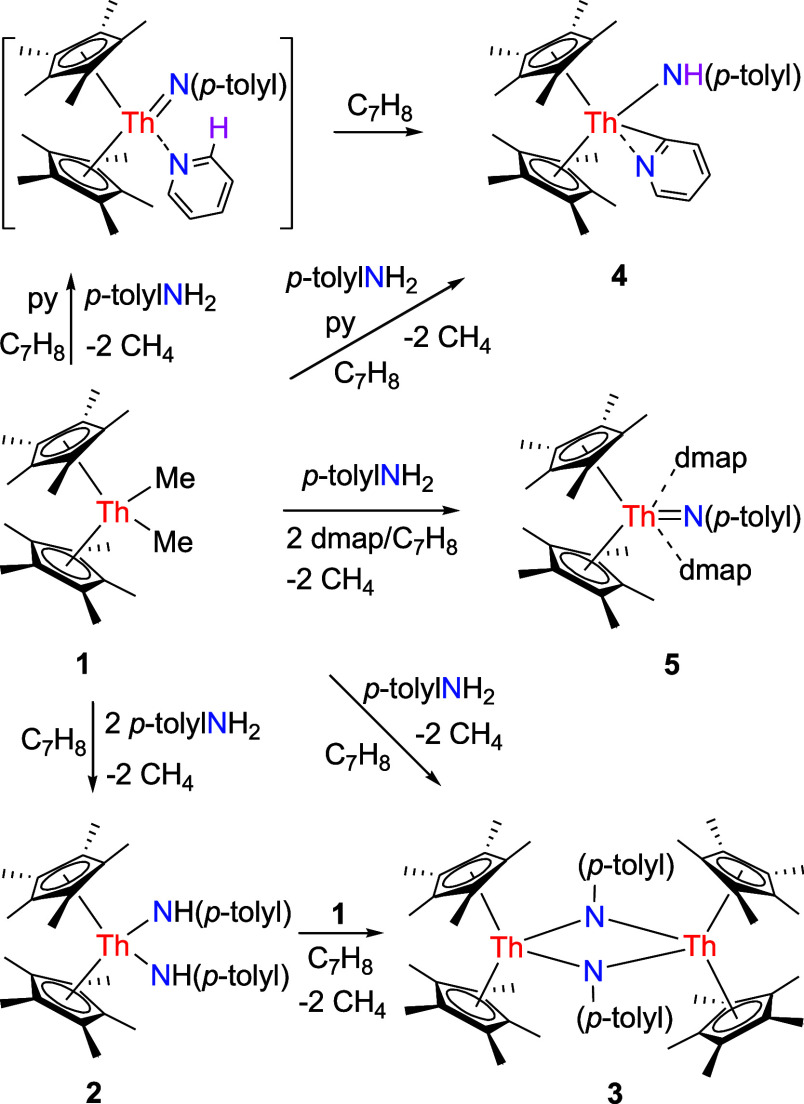
Synthesis of Compounds **2**–**5**

**Figure 2:**
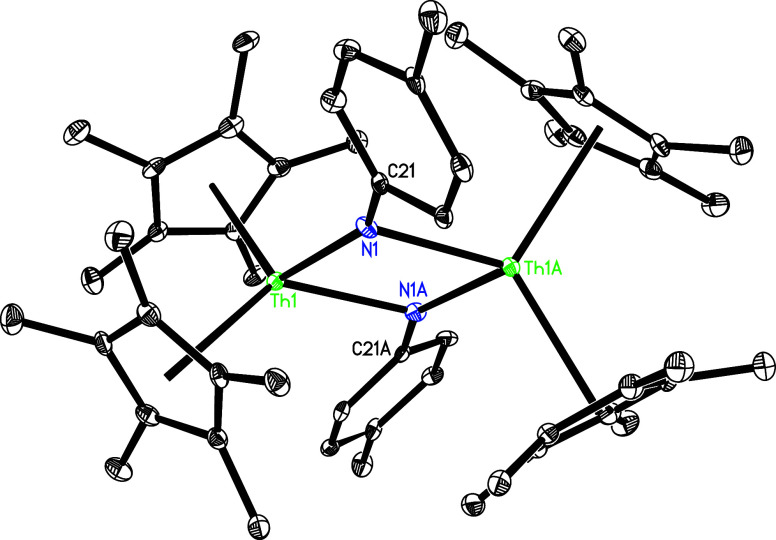
Molecular structure of **3** (thermal ellipsoids drawn at the 35% probability level).

**Figure 3:**
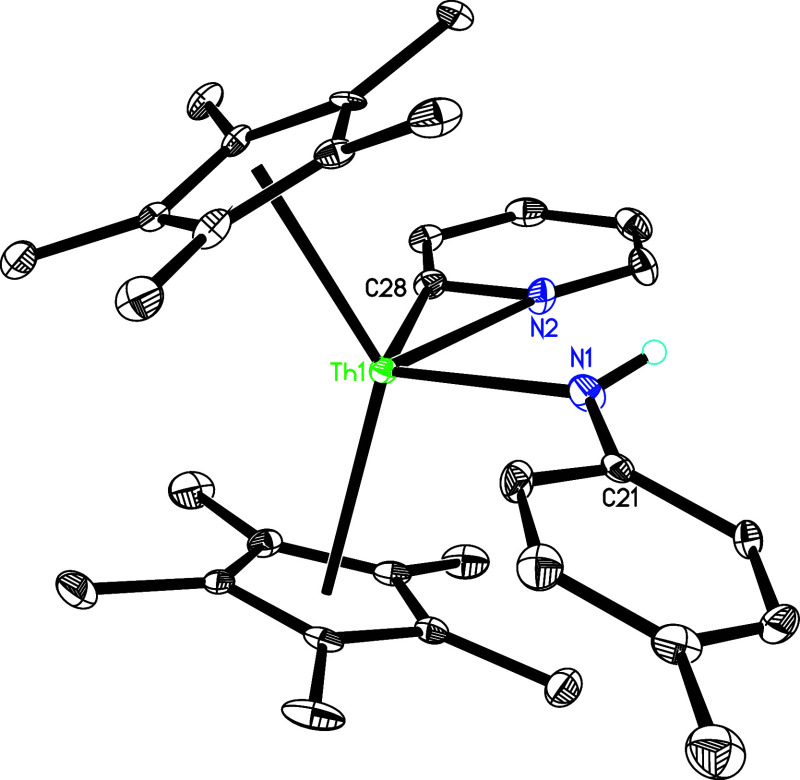
Molecular structure of **4** (thermal ellipsoids drawn at the 35% probability level).

**Figure 4:**
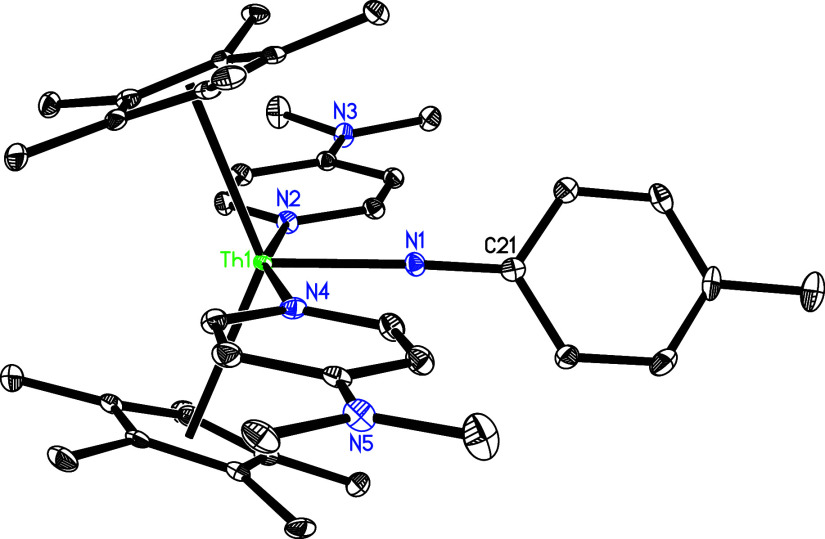
Molecular structure of **5** (thermal ellipsoids drawn at the 35% probability level).

**Figure 5:**
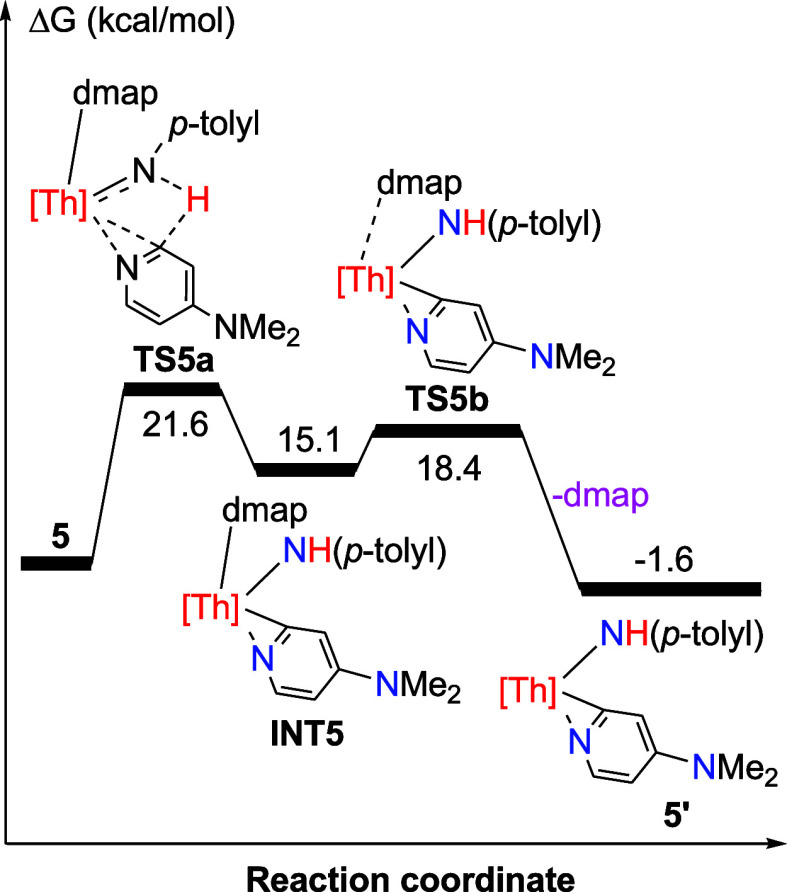
Free energy profile (kcal/mol) for the reaction of **5** ⇌ **5′** + dmap. [Th] = (η^5^-C_5_Me_5_)_2_Th.

**Scheme 2:**
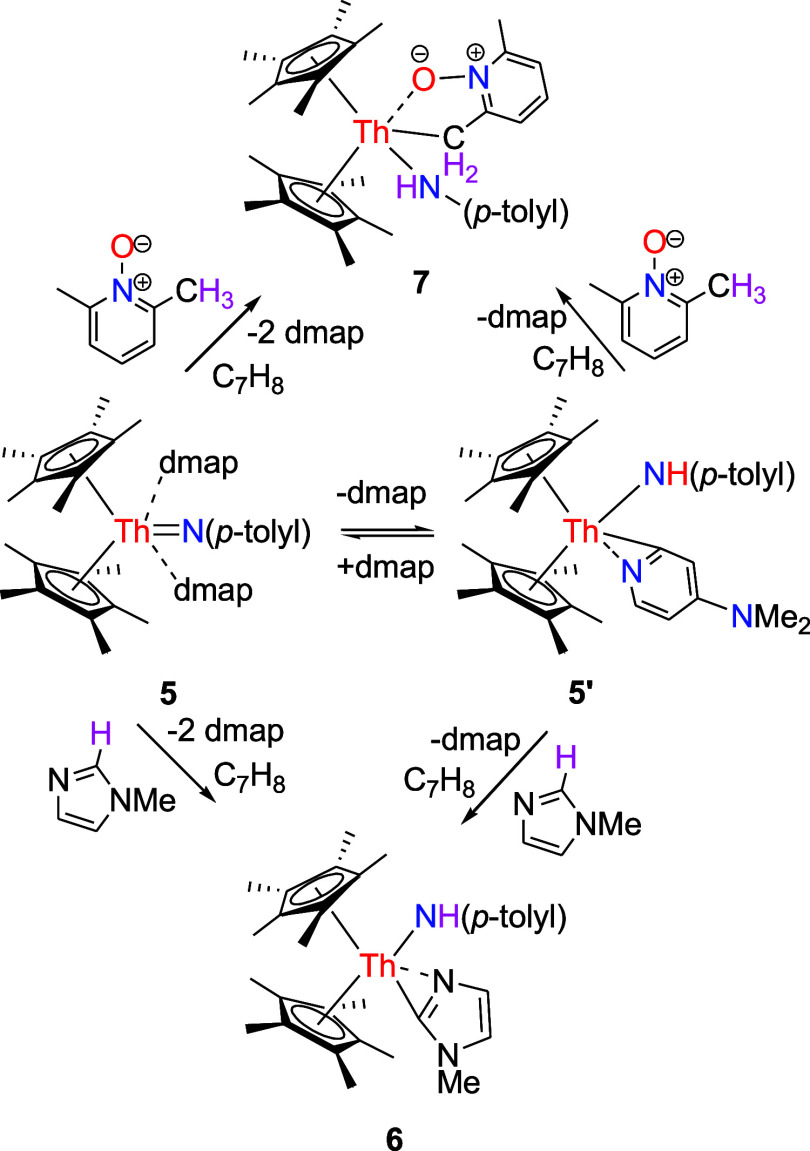
Synthesis of Compounds **6** and **7**

### Reactivity Studies

Next, we shift our focus to the reactivity of complex **5**, juxtaposing the products from its reaction with various small molecules against those isolated from [η^5^-1,2,4-(Me_3_C)_3_C_5_H_2_]_2_Th=N­(*p*-tolyl) (Figure S1),^7a,8^ (η^5^-C_5_Me_5_)_2_Th=N­(mesityl)­(dmap) (Figure S2),^7f,7,8^ [η^5^-1,3-(Me_3_C)_2_C_5_H_3_]_2_Th=N­(dipp)­(dmap) and [η^5^-1,2,4-(Me_3_Si)_3_C_5_H_2_]_2_Th=N­(*p*-tolyl)­(bipy) (Figures S3 and S4).^7y,7,8^ To make comparisons easier for the reader, we have organized the reactions by substance class.

### Reaction with Lewis Bases

In contrast to [η^5^-1,3-(Me_3_C)_2_C_5_H_3_]_2_Th=N­(dipp)­(dmap) (Figure S3),^7z,8^ treatment of complex **5** or **5′** with 1-methylimidazole does not yield a simple 1-methylimidazole adduct. Instead, it produces the amido imidazolyl complex (η^5^-C_5_Me_5_)_2_Th­[NH­(*p*-tolyl)]­(κ^2^-*C*,*N*-1-MeC_3_H_2_N_2_) (**6**) in quantitative conversion with concomitant dmap loss (Scheme 2), a result likely reflecting the reduced steric hindrance of the *p*-tolyl group compared with the 2,6-*^i^* Pr_2_C_6_H_3_ group. The molecular structure of **6** is depicted in Figure 6, with selected bond distances and angles listed in Table 1. The Th–N (1) and Th–N(2) distances are 2.365(7) and 2.448(7) Å, respectively, whereas the Th–C(28) distance is 2.485(7) Å. Furthermore, proton transfer between complex **5** or **5′** and 2,6-Me_2_C_5_H_3_NO leads to the formation of the amido alkyl complex (η^5^-C_5_Me_5_)_2_Th­[NH­(*p*-tolyl)]­(κ^2^-*C*,*O*-2-CH_2_–6-MeC_5_H_3_NO) (**7**) (Scheme 2). The molecular structure of **7** is presented in Figure 7, while key bond distances and angles are summarized in Table 1. The Th–O (1) distance is 2.418(5) Å, whereas the Th–N(2) distance is 2.351(6) Å and Th–C(21) distance is 2.598(8) Å. Furthermore, unlike [η^5^-1,3-(Me_3_C)_2_C_5_H_3_]_2_Th=N­(dipp)­(dmap) (Figure S3),^7z,8^ reacting complex **5** with Me_3_PO does not produce a Me_3_PO adduct. Instead, the reaction yields the bis-amido complex (η^5^-C_5_Me_5_)_2_Th­[NH­(*p*-tolyl)]­[κ^2^-*C*,*N*-N­(*p*-tolyl)­P­(Me_2_)­CH_2_] (**8**) in 38% yield (Scheme 3), likely due to the reduced steric bulk imposed by the *p*-tolyl group compared to that of the 2,6-*^i^* Pr_2_C_6_H_3_ group. We propose that complex **5** initially reacts with Me_3_PO to give a four-membered intermediate with the loss of dmap (Scheme 3). This intermediate then eliminates the oxido complex “(η^5^-C_5_Me_5_)_2_ThO” to generate the organic compound Me_3_PN­(*p*-tolyl), which subsequently reacts with another molecule of **5** via proton transfer to form the product **8** along with dmap release. The molecular structure of **8** is shown in Figure 8, and selected bond distances and angles are listed in Table 1. The Th–N (1) and Th–N(2) distances are 2.489(6) and 2.348(5) Å, respectively, whereas the Th–C(28) is 2.700(7) Å.

**Figure 6:**
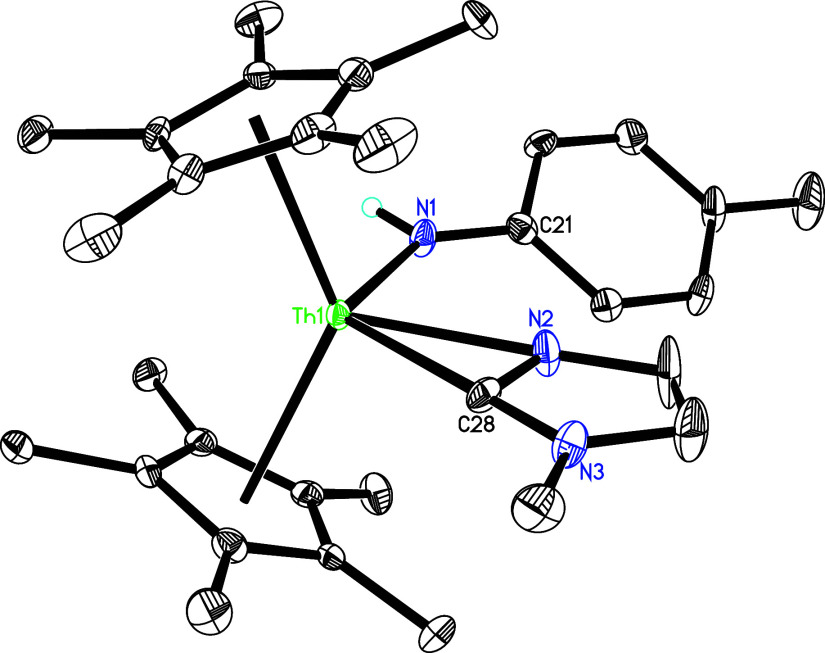
Molecular structure of **6** (thermal ellipsoids drawn at the 35% probability level).

**Figure 7:**
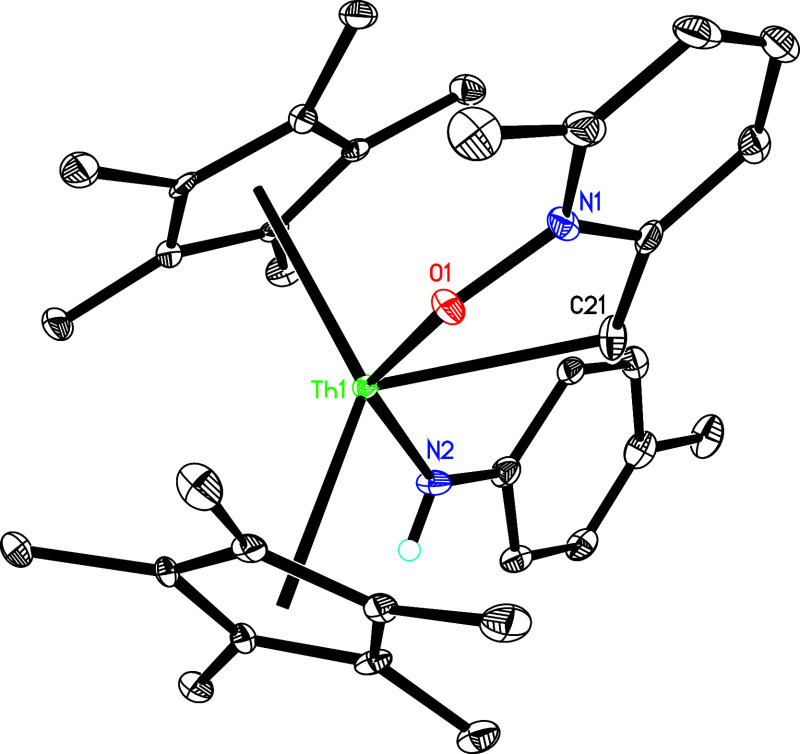
Molecular structure of **7** (thermal ellipsoids drawn at the 35% probability level).

**Scheme 3:**
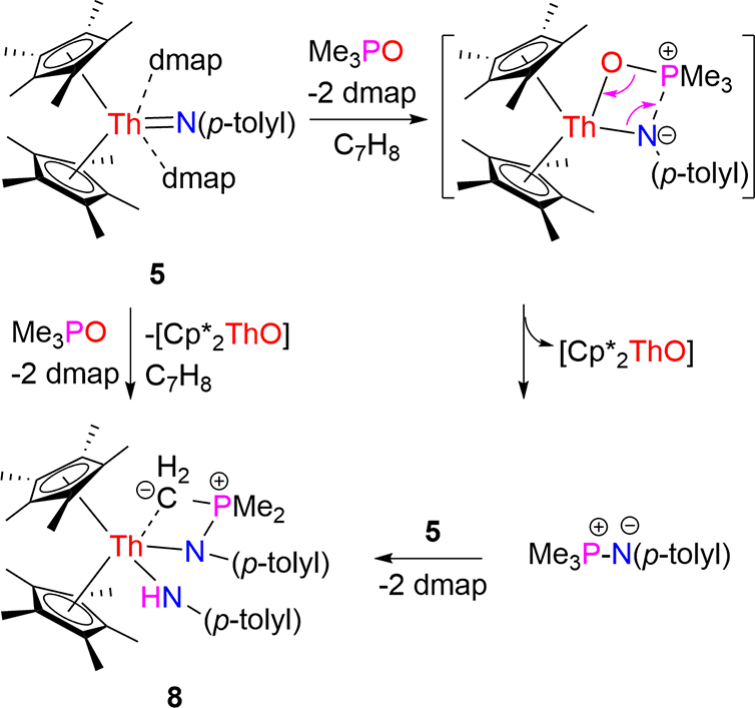
Synthesis of Compound **8**

**Figure 8:**
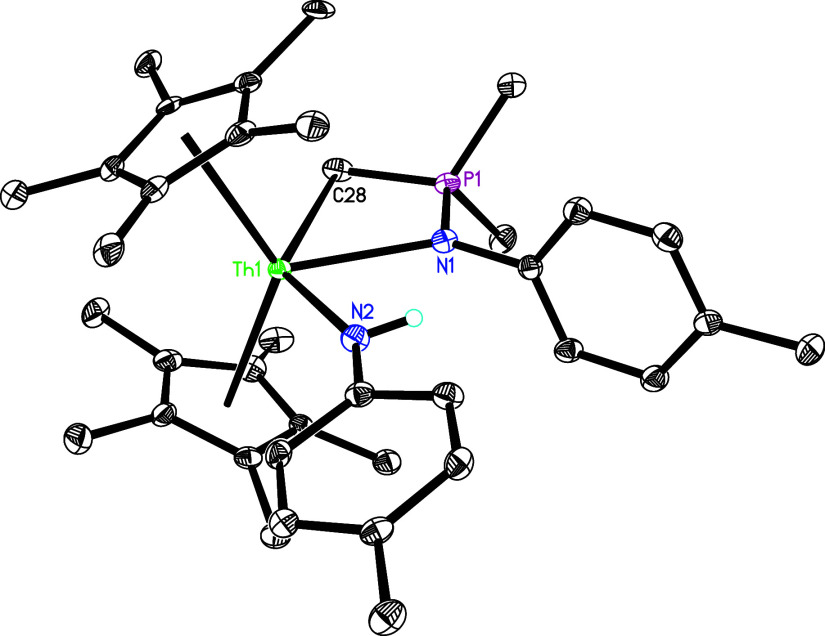
Molecular structure of **8** (thermal ellipsoids drawn at the 35% probability level).

### Reaction with Chalcogen Elements and Metal Halides

Moreover, like the thorium imido complex [η^5^-1,2,4-(Me_3_C)_3_C_5_H_2_]_2_Th=N­(*p*-tolyl) (Figure S1),^7b,8^ complex **5** reacts with 0.25 equiv of elemental sulfur (S_8_) at 40 °C in toluene to form the four-membered complex (η^5^-C_5_Me_5_)_2_Th­[N­(*p*-tolyl)­SS]­(dmap) (**9**) with one dmap loss (Scheme 4). Figure 9 shows the molecular structure of **9**, while Table 1 summarizes the selected bond distances and angles. The relatively long Th–N(2) distance of 2.661(4) Å is indicative of a datively coordinated nitrogen atom, while the Th–N (1) and Th–S (1) distances are 2.402(4) and 2.886 (1) Å, respectively. Nevertheless, in contrast to [η^5^-1,2,4-(Me_3_C)_3_C_5_H_2_]_2_Th­[N­(*p*-tolyl)­SS] derived from [η^5^-1,2,4-(Me_3_C)_3_C_5_H_2_]_2_Th=N­(*p*-tolyl) (Figure S1),^7b,8^ complex **9** undergoes further reaction with 0.25 equiv of elemental sulfur (S_8_) to form the six-membered complex (η^5^-C_5_Me_5_)_2_Th­[N­(*p*-tolyl)­S_4_] (**10**) with concomitant dmap release (Scheme 4), owing to the reduced steric bulk of the C_5_Me_5_ ligand compared to that of the 1,2,4-(Me_3_C)_3_C_5_H_2_ ligand. Moreover, complex **10** can also be directly accessed via the reaction of **5** with 0.5 equiv of sulfur (S_8_) (Scheme 4). This reactivity contrasts with that of [η^5^-1,3-(Me_3_C)_2_C_5_H_3_]_2_Th=N­(dipp)­(dmap), which forms the six-membered complex [η^5^-1,3-(Me_3_C)_2_C_5_H_3_]_2_Th­[SSN­(dipp)­SS] (Figure S3),^7z,8^ likely due to the diminished steric bulk of the *p*-tolyl substituent compared with the 2,6-*^i^* Pr_2_C_6_H_3_ group. The molecular structure of **10** is shown in Figure 10, with selected bond distances and angles detailed in Table 1. The Th–N (1) and Th–S (1) distances are 2.380(11) and 2.819(4) Å, respectively, and the N(1)–Th-S (1) angle is 109.8(3)°. Nevertheless, in contrast to [η^5^-1,2,4-(Me_3_C)_3_C_5_H_2_]_2_Th=N­(*p*-tolyl) forming a four-membered complex [η^5^-1,2,4-(Me_3_C)_3_C_5_H_2_]_2_Th­[N­(*p*-tolyl)­SeSe] with selenium (Figure S1),^7e,8^ when imido complex **5** is treated with 4 equiv of selenium in toluene at 40 °C, the six-membered complex (η^5^-C_5_Me_5_)_2_Th­[N­(*p*-tolyl)­Se_4_] (**11**) forms simultaneously with release of two equivalents of dmap (Scheme 4). This effect likely arises from the lower steric hindrance of the C_5_Me_5_ ligand compared with the 1,2,4-(Me_3_C)_3_C_5_H_2_ ligand. Figure 11 shows the molecular structure of **11**, where Th–N (1) and Th–Se (1) distances are 2.351(8) and 2.927 (1) Å, respectively, and the N(1)–Th-Se (1) angle is 113.3(2)° (Table 1). However, treating **5** with selenium at 15 °C in toluene yields the amido selenido complex (η^5^-C_5_Me_5_)_2_Th­[NH­(*p*-tolyl)]­[κ^2^-*N,Se*-2-Se-4-(Me_2_N)­C_5_H_3_N] (**12**) with one dmap loss (Scheme 4). In this case, selenium inserts into the pyridyl Th-[κ^2^-*C*,*N*-4-(Me_2_N)­C_5_H_3_N] moiety, supporting the notion of an equilibrium between **5** and **5′** + dmap in solution. Figure 12 displays the molecular structure of **12**, and Table 1 provides the selected bond distances and angles. The relatively long Th–N (1) distance of 2.590(4) Å is indicative of a datively coordinated nitrogen atom, while the Th–N(3) distance is 2.348(4) Å, and the Th–Se (1) is 2.959 (1) Å. Similarly, treatment of imido **5** with 1 equiv of tellurium in toluene at 120 °C produces the amido tellurido complex (η^5^-C_5_Me_5_)_2_Th­[NH­(*p*-tolyl)]­[κ^2^-*N,Te*-2-Te-4-(Me_2_N)­C_5_H_3_N] (**13**) with one dmap loss (Scheme 4), again indicating an equilibrium between **5** and **5′** + dmap in solution. Figure 13 presents the molecular structure of **13**, where the Th–N(2) distance of 2.594(5) Å signifies dative nitrogen atom coordination, the Th–N (1) distance is 2.333(5) Å, and the Th–Te (1) distance measures 3.162(2) Å (Table 1).

**Scheme 4:**
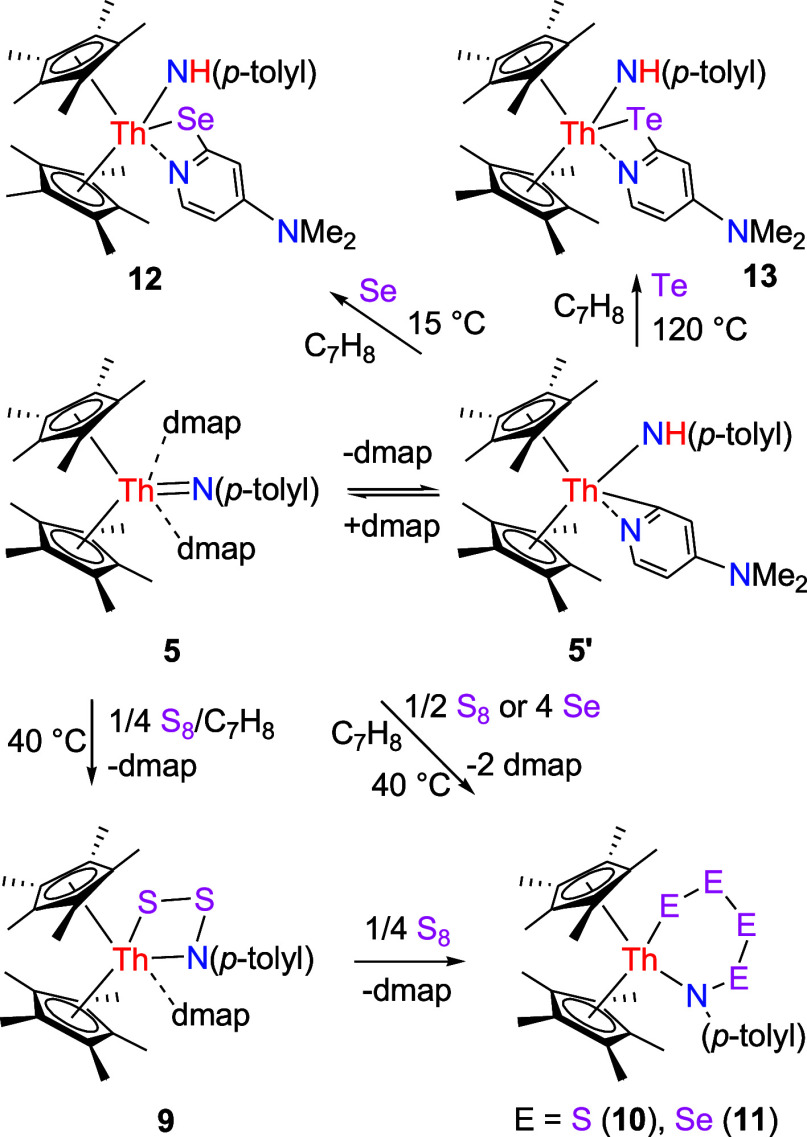
Synthesis of Compounds **9**–**13**

**Figure 9:**
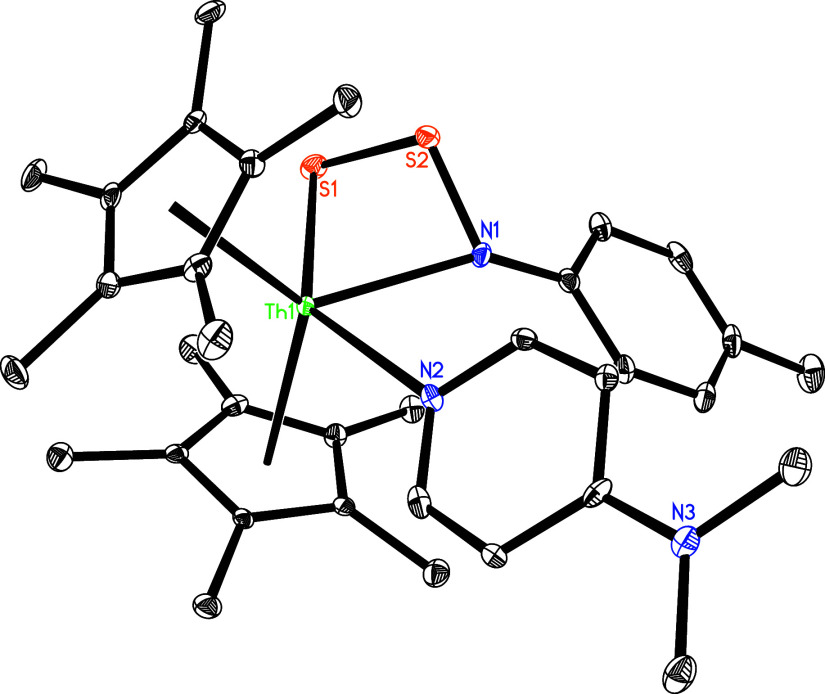
Molecular structure of **9** (thermal ellipsoids drawn at the 35% probability level).

**Figure 10:**
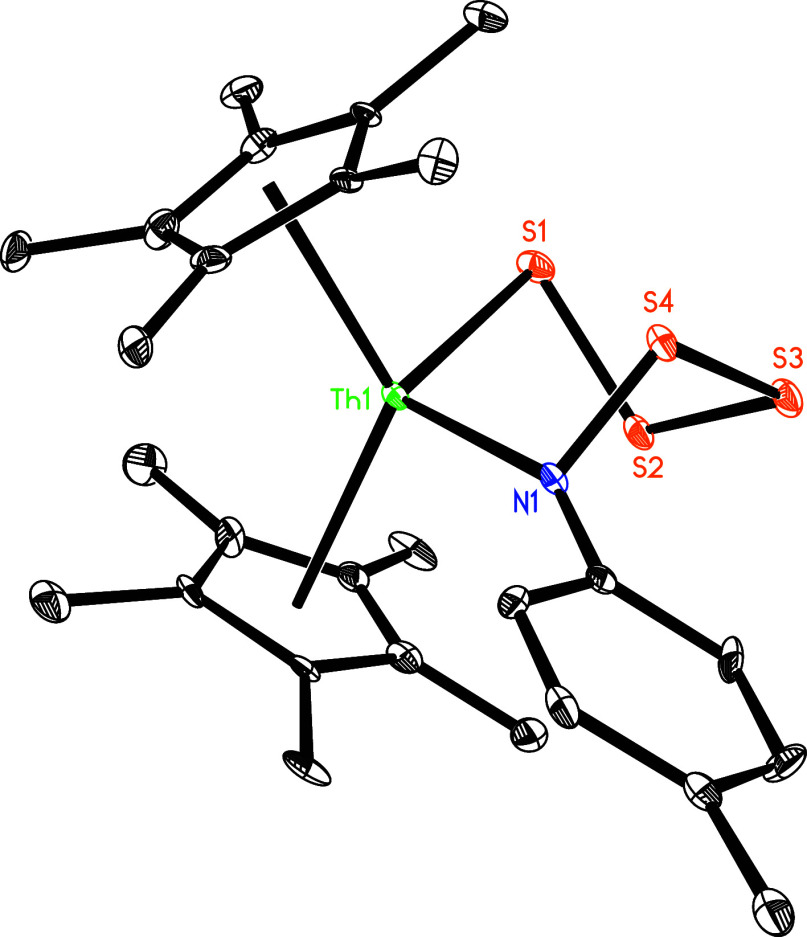
Molecular structure of **10** (thermal ellipsoids drawn at the 35% probability level).

**Figure 11:**
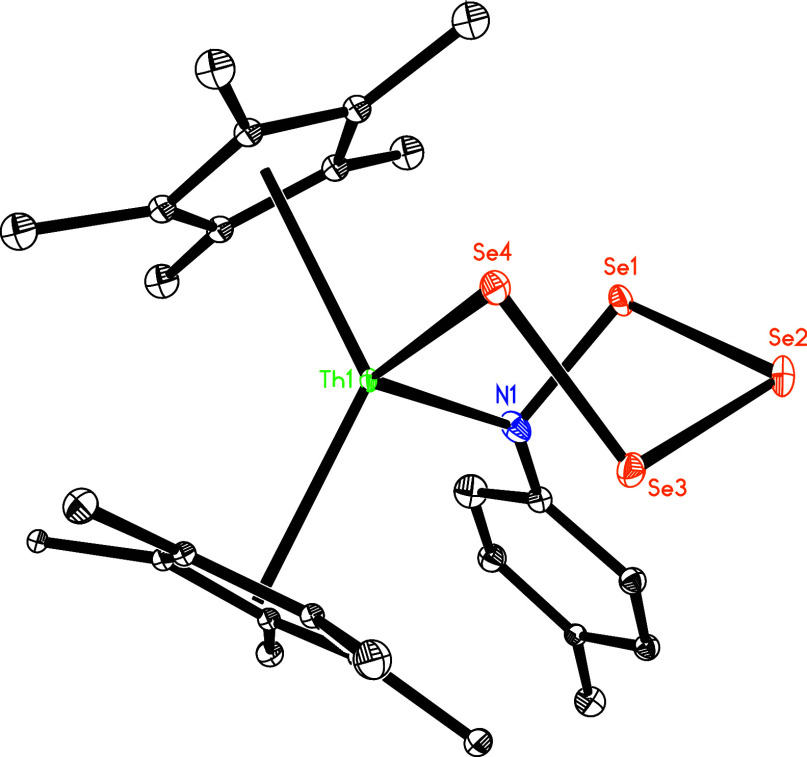
Molecular structure of **11** (thermal ellipsoids drawn at the 35% probability level).

**Figure 12:**
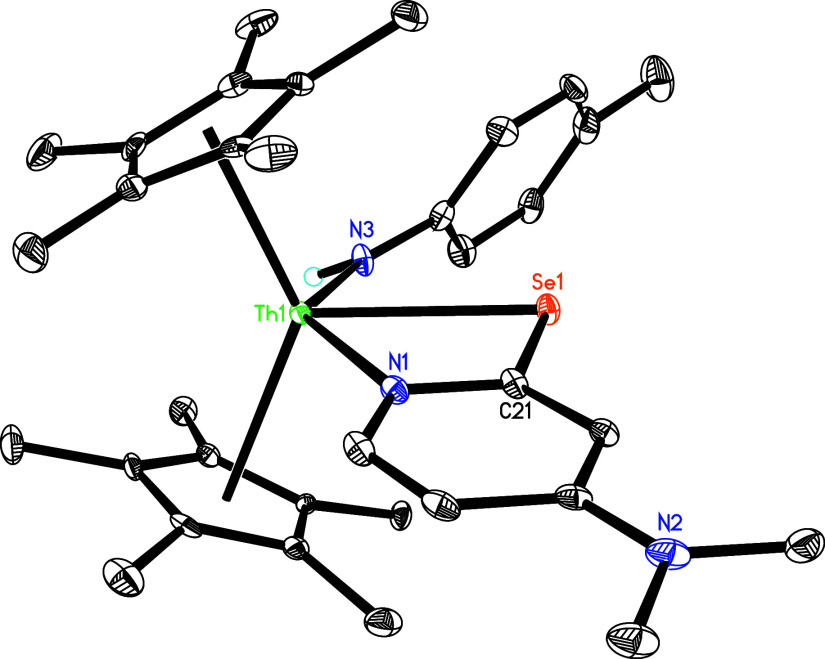
Molecular structure of **12** (thermal ellipsoids drawn at the 35% probability level).

**Figure 13:**
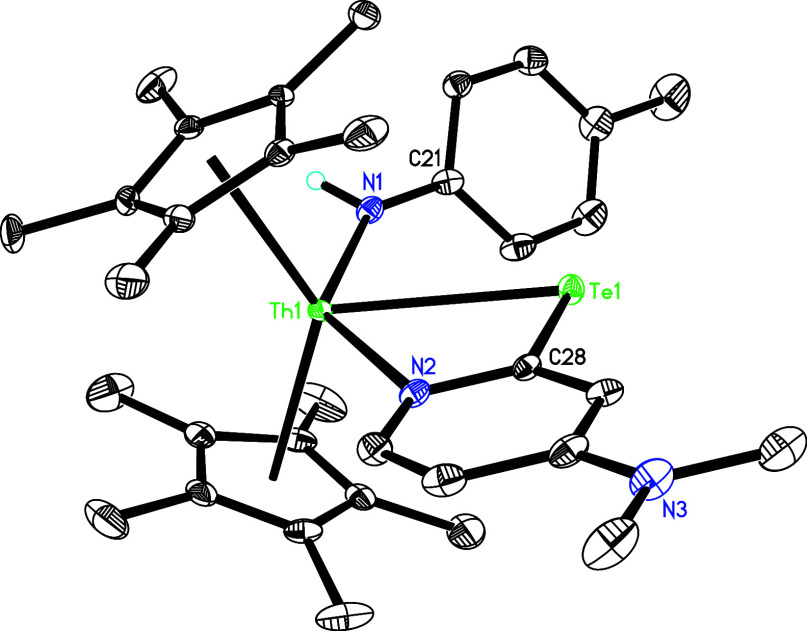
Molecular structure of **13** (thermal ellipsoids drawn at the 35% probability level).

Like the imido compounds (η^5^-C_5_Me_5_)_2_Th=N­(mesityl)­(dmap) and [η^5^-1,3-(Me_3_C)_2_C_5_H_3_]_2_Th=N­(dipp)­(dmap) (Figures S2 and S3),^7f,7,8^ complex **5** readily reacts with metal halides. For instance, treatment of **5** with 2 equiv of AgF yields the difluoride complex (η^5^-C_5_Me_5_)_2_ThF_2_(dmap) (**14**) alongside other unidentified silver species that arise from the loss of nitrene (*p*-tolylN:) and one dmap molecule (Scheme 5). The molecular structure of **14** is shown in Figure 14, with key bond distances and angles summarized in Table 1. In particular, the Th–F (1) and Th–F(2) distances measure 2.182(5) and 2.184(5) Å, respectively, whereas the Th–N (1) bond extends to 2.607(6) Å, a distance that confirms the presence of a dative nitrogen atom coordination. Moreover, in contrast to compounds (η^5^-C_5_Me_5_)_2_Th=N­(mesityl)­(dmap) and [η^5^-1,3-(Me_3_C)_2_C_5_H_3_]_2_Th=N­(dipp)­(dmap), which, upon addition of copper halides, form heterobimetallic compounds (η^5^-C_5_Me_5_)_2_Th­(X)­[N­(mesityl)­Cu­(dmap)] (X = Cl, Br) and [η^5^-1,3-(Me_3_C)_2_C_5_H_3_]_2_Th­(Cl)­[N­(dipp)­Cu­(dmap)] (Figures S2 and S3),^7f,7,8^ respectively, treatment of **5** with CuCl or CuBr results in the isolation of dichloride complex (η^5^-C_5_Me_5_)_2_ThCl_2_(dmap)_2_ (**15**) and dibromide complex (η^5^-C_5_Me_5_)_2_ThBr_2_(dmap) (**16**), respectively. This divergent reactivity is presumably due to the reduced steric hindrance imparted by the *p*-tolyl substituent compared with the mesityl and 2,6-*^i^* Pr_2_C_6_H_3_ groups. The difluoride complex (η^5^-C_5_Me_5_)_2_ThF_2_(dmap) (**14**) and dibromide complex (η^5^-C_5_Me_5_)_2_ThBr_2_(dmap) (**16**) form adducts with one dmap molecule, but complex (η^5^-C_5_Me_5_)_2_ThCl_2_(dmap)_2_ (**15**) coordinates two dmap ligands. This difference likely arises from the varying steric protection around the metal atom offered by the coordinated ligands–in (η^5^-C_5_Me_5_)_2_ThCl_2_, there is sufficient space to accommodate two dmap ligands; by contrast, the shorter Th–F bond lengths or the bulkier bromide ligands impart greater steric shielding at the metal atom, precluding the coordination of two dmap ligands. The molecular structures of **15** and **16** are shown in Figures 15 and 16, respectively, and selected bond distances and angles are provided in Table 1. In complex **15**, the Th–Cl (1) and Th–Cl(2) distances are 2.732 (1) and 2.735 (1) Å, respectively, while the Th–N (1) and Th–N(3) distances are 2.746(4) and 2.737(4) Å, respectively, confirming a dative mode of nitrogen atom coordination. In complex **16**, the Th–Br (1) and Th–Br(2) distances are 2.872 (1) and 2.921 (1) Å, respectively, whereas the Th–N (1) distance is 2.628(5) Å, further indicating that the nitrogen atom is coordinated in a dative fashion.

**Figure 14:**
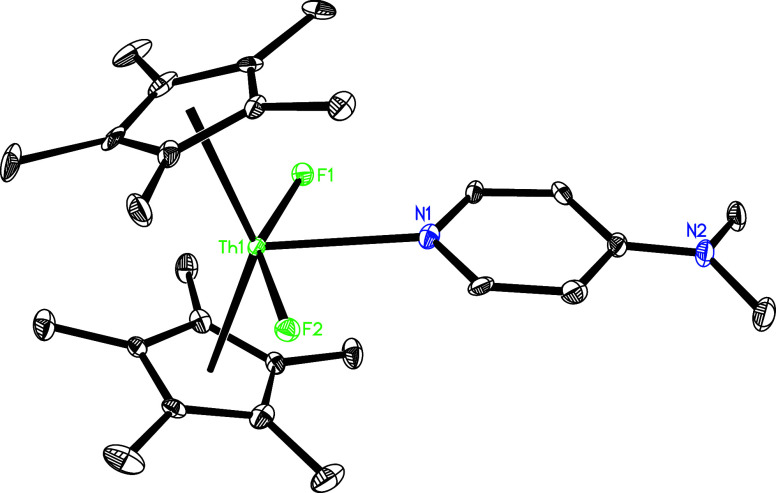
Molecular structure of **14** (thermal ellipsoids drawn at the 35% probability level).

**Scheme 5:**
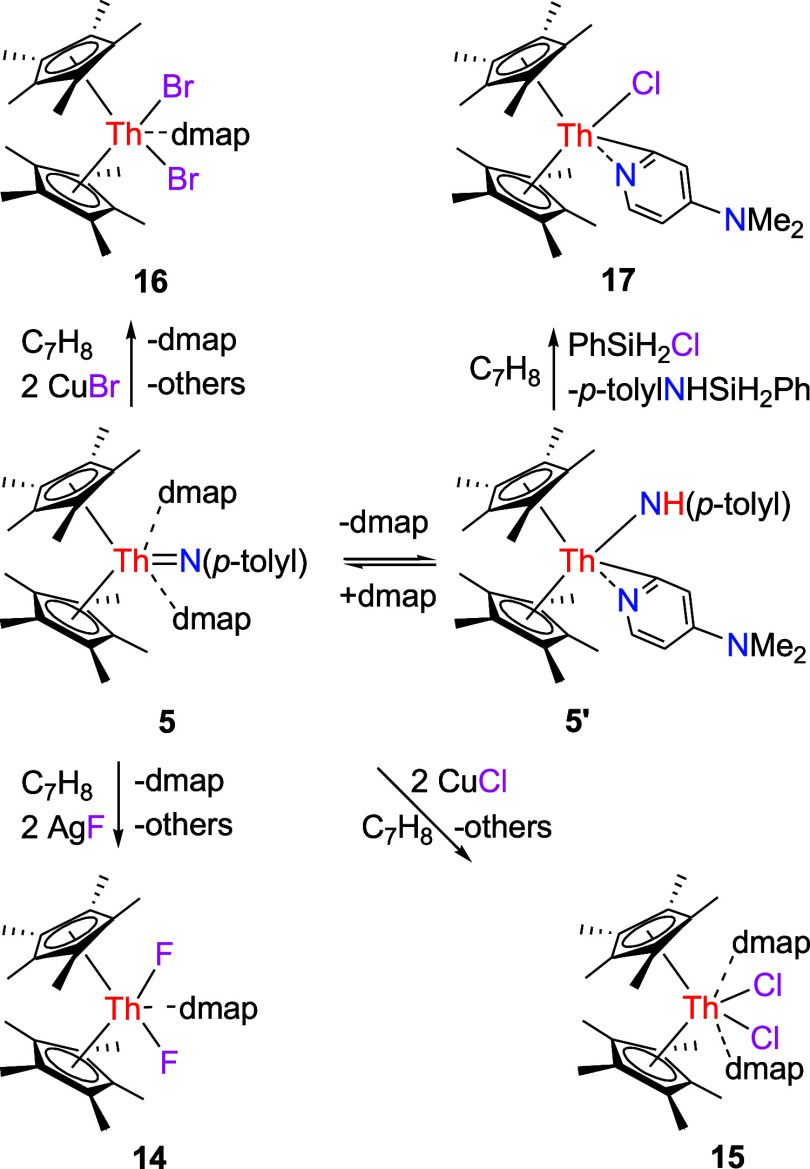
Synthesis of Compounds **14**–**17**

**Figure 15:**
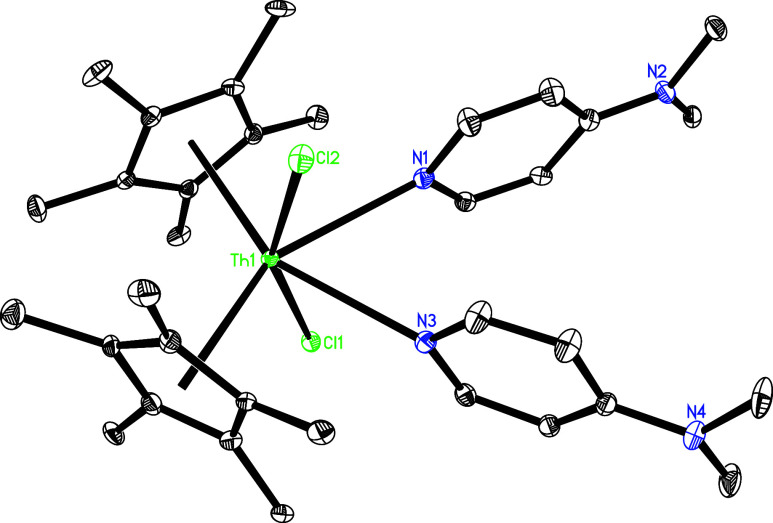
Molecular structure of **15** (thermal ellipsoids drawn at the 35% probability level).

**Figure 16:**
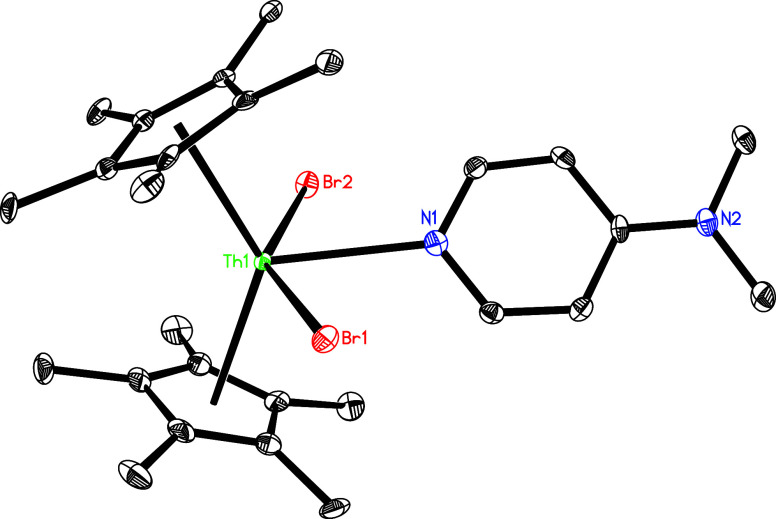
Molecular structure of **16** (thermal ellipsoids drawn at the 35% probability level).

### Reaction with Chlorosilanes and Silanes

Moreover, contrary to the thorium imido complex [η^5^-1,2,4-(Me_3_C)_3_C_5_H_2_]_2_Th=N­(*p*-tolyl) and [η^5^-1,2,4-(Me_3_Si)_3_C_5_H_2_]_2_Th=N­(*p*-tolyl)­(bipy) forming with PhSiH_2_Cl the amido chloride complexes [η^5^-1,2,4-(Me_3_E)_3_C_5_H_2_]_2_Th­(Cl)­[N­(*p*-tolyl)­SiH_2_Ph] (E = C,^7e^ Si^7y^) (Figures S1 and S4),^8^ and (η^5^-C_5_Me_5_)_2_Th=N­(mesityl)­(dmap) and [η^5^-1,3-(Me_3_C)_2_C_5_H_3_]_2_Th=N­(dipp)­(dmap) forming the dichloride complexes (η^5^-C_5_Me_5_)_2_ThCl_2_(dmap) and [η^5^-1,3-(Me_3_C)_2_C_5_H_3_]_2_ThCl_2_(dmap) (Figures S2 and S3),^7g,7,8^ respectively, complex **5** reacts with PhSiH_2_Cl to give the chloro pyridyl complex (η^5^-C_5_Me_5_)_2_Th­(Cl)­[κ^2^-*C*,*N*-4-(Me_2_N)­C_5_H_3_N] (**17**) and *p*-tolylNHSiH_2_Ph in quantitative conversion with the loss of one dmap ligand (Scheme 5), in which the *p*-tolylNH moiety acts a nucleophile, attacking PhSiH_2_Cl. This reaction provides another compelling evidence for the established equilibrium between **5** and **5′** + dmap in the solution. The molecular structure of **17** is shown in Figure 17, and selected bond distances and angles are listed in Table 1. The Th–N (1) and Th–Cl (1) distances are 2.407(8) and 2.678(3) Å, respectively, whereas the Th–C(21) distance is 2.428(10) Å.

**Figure 17:**
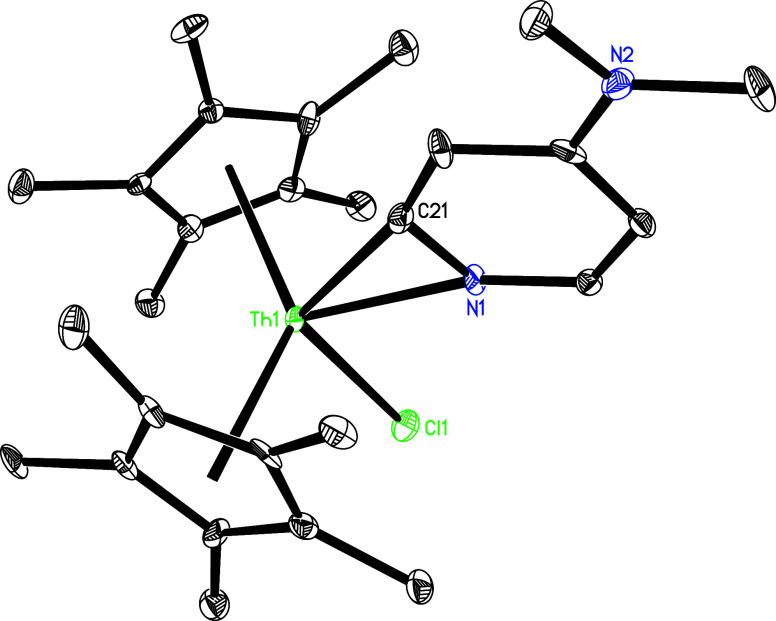
Molecular structure of **17** (thermal ellipsoids drawn at the 35% probability level).

Like the imido compounds [η^5^-1,2,4-(Me_3_C)_3_C_5_H_2_]_2_Th=N­(*p*-tolyl) and (η^5^-C_5_Me_5_)_2_Th=N­(mesityl)­(dmap) (Figures S1 and S2),^7c,7,8^ complex **5** readily reacts with silanes. For example, treatment of complex **5** with 1 equiv of PhSiH_3_ gives the amido phenyl complex (η^5^-C_5_Me_5_)_2_Th­[κ^3^-*C*,*N,N*-(4-Me_2_NC_5_H_3_N)­SiH­(Ph)­N­(4-MeC_6_H_3_)] (**18**) with H_2_ and one dmap release (Scheme 6). Like the reaction observed for thorium imido complex [η^5^-1,2,4-(Me_3_C)_3_C_5_H_2_]_2_Th=N­(*p*-tolyl) with PhSiH_3_ (Figure S1),^7c,8^ we propose that **5** initially reacts with PhSiH_3_ to give an amido hydride intermediate **A** while releasing dmap (Scheme 6; down). Next, this intermediate undergoes C–H activation of the *p*-tolyl group with concurrent H_2_ elimination, yielding an amido phenyl intermediate **B** that subsequently coordinates with the liberated dmap to form a dmap adduct **C**. Finally, this adduct experiences further C–H activation of the dmap ligand along with additional H_2_ release, ultimately delivering product **18** (Scheme 6). Alternatively, once the amido hydride intermediate **A** is generated, it effects C–H bond activation of the liberated dmap with concomitant H_2_ elimination to afford an amido-pyridyl intermediate **D** (Scheme 6; up). Intramolecular nucleophilic attack then delivers amido-hydride intermediate **E**, which undergoes a second C–H bond activation of the *p*-tolyl ring, with further H_2_ release, to furnish product **18** (Scheme 6). This reactivity is contrary to (η^5^-C_5_Me_5_)_2_Th=N­(mesityl)­(dmap) forming an amido alkyl dmap adduct (η^5^-C_5_Me_5_)_2_Th­[κ^2^-*N*, *C*-{N­(2-CH_2_–4,6-Me_2_C_6_H_2_)­(SiH_2_Ph)}]­(dmap) (Figure S2),^7g,8^ attributed to the reduced steric hindrance imposed by the *p*-tolyl group compared with the mesityl group. Similarly, reacting imido complex **5** with 1 equiv of Ph_2_SiH_2_ yields the corresponding amido phenyl complex (η^5^-C_5_Me_5_)_2_Th­[κ^3^-*C*,*N,N*-(4-Me_2_NC_5_H_3_N)­SiPh_2_N­(4-MeC_6_H_3_)] (**19**) with H_2_ and one dmap release (Scheme 6). The molecular structure of **18** is shown in Figure 18, whereas the molecular structure of **19** is provided in the Supporting Information. In complex **18**, the Th–N (1), Th–N(2) and Th–C(21) distances are 2.356(4), 2.630(4) and 2.514(5) Å, respectively, which are comparable to those found in **19** (2.363(3), 2.624(3) and 2.488(4) Å, respectively).

**Scheme 6:**
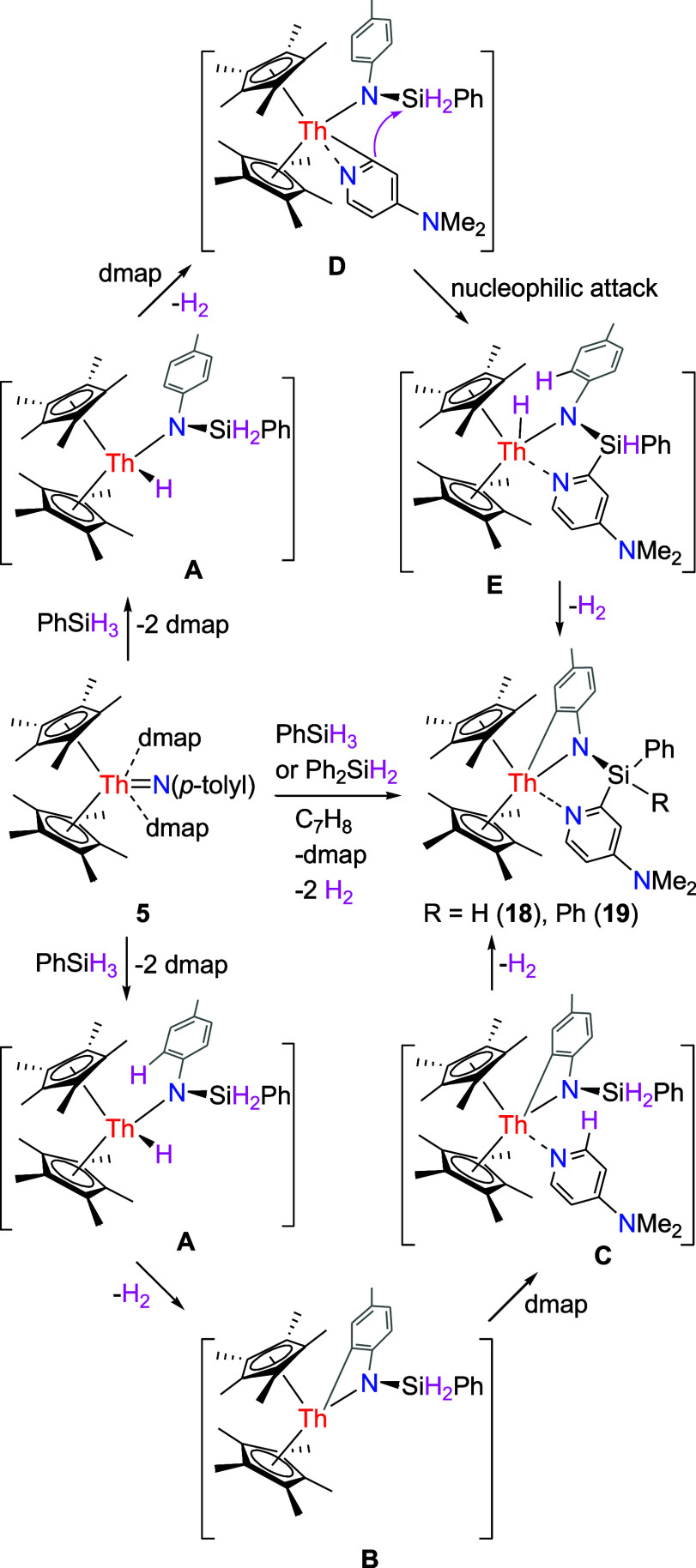
Synthesis of Compounds **18** and **19**

**Figure 18:**
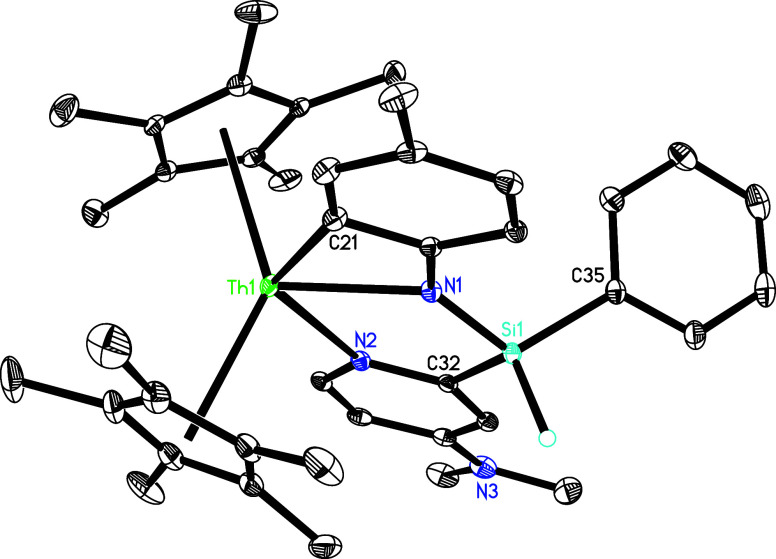
Molecular structure of **18** (thermal ellipsoids drawn at the 35% probability level).

### Reaction with Alkynes

In analogy to [η^5^-1,2,4-(Me_3_C)_3_C_5_H_2_]_2_Th=N­(*p*-tolyl) (Figure S1),^7b,8^ complex **5** reacts with alkynes RC≡CR′. For example, treatment of complex **5** with PhC≡CPh gives a pyridyl amido complex (η^5^-C_5_Me_5_)_2_Th­[N­(*p*-tolyl)­C­(Ph)=CHPh]­[κ^2^-*C*,*N*-4-(Me_2_N)­C_5_H_3_N] (**20**) with the loss of one dmap ligand (Scheme 7). A plausible reaction mechanism begins with a [2 + 2] cycloaddition between **5** and PhC≡CPh. This is followed by deprotonation of an α-H on dmap, ultimately forming the final product **20** (Scheme 7). Nevertheless, imidos (η^5^-C_5_Me_5_)_2_Th=N­(mesityl)­(dmap) and [η^5^-1,3-(Me_3_C)_2_C_5_H_3_]_2_Th=N­(dipp)­(dmap) do not show reactivity with PhC≡CPh,^7g,7z^ whereas complexes [η^5^-1,2,4-(Me_3_C)_3_C_5_H_2_]_2_Th=N­(*p*-tolyl) (Figure S1)^7b,8^ and **5** do. This effect is ascribed to the diminished steric bulk of the *p*-tolyl substituent relative to that of the mesityl and 2,6-*^i^* Pr_2_C_6_H_3_ groups. The molecular structure of **20** is shown in Figure 19, and selected bond distances and angles are listed in Table 1. The Th–N (1) and Th–N(2) distances are 2.425(6) and 2.432(6) Å, respectively, whereas the Th–C(46) distance is 2.455(7) Å.

**Scheme 7:**
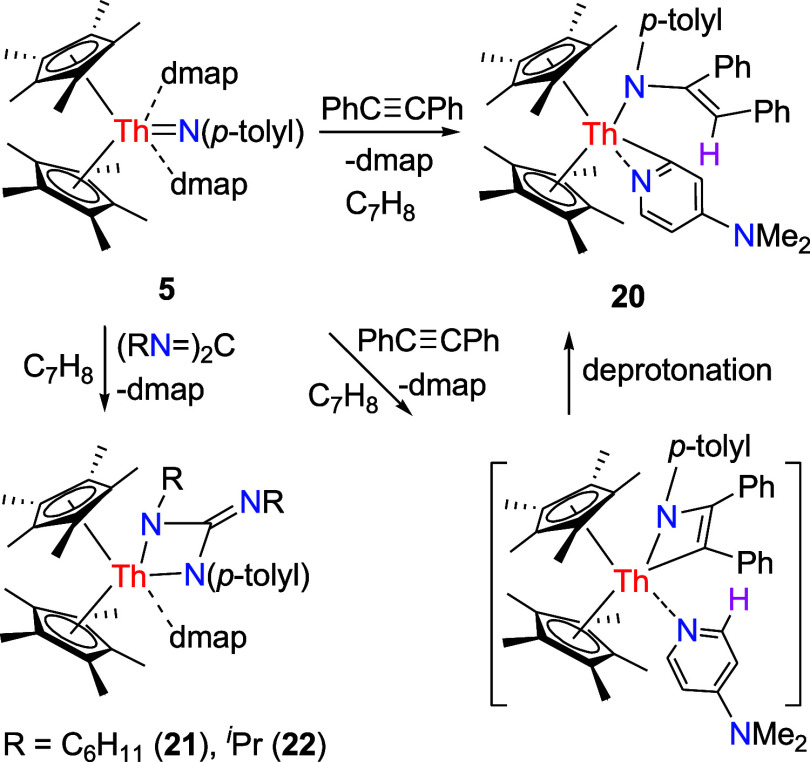
Synthesis of Compounds **20**–**22**

**Figure 19:**
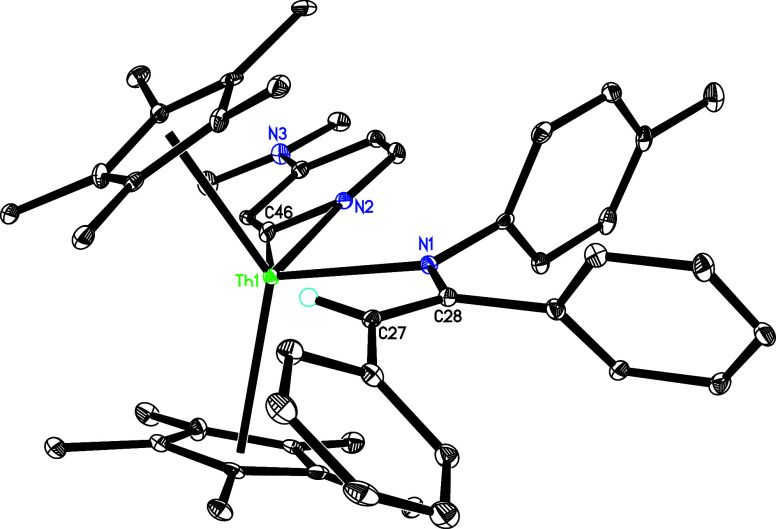
Molecular structure of **20** (thermal ellipsoids drawn at the 35% probability level).

### Reaction with Carbodiimides, Ketones, Isothiocyanates, CS_2_, Esters, and Amidates

In the presence of heterounsaturated organic substrates with polar C-E bonds, the thorium imido complexes [η^5^-1,2,4-(Me_3_C)_3_C_5_H_2_]_2_Th=N­(*p*-tolyl) (Figure S1),^7b,8^ (η^5^-C_5_Me_5_)_2_Th=N­(mesityl)­(dmap) (Figure S2),^7g,8^ [η^5^-1,3-(Me_3_C)_2_C_5_H_3_]_2_Th=N­(dipp)­(dmap) (Figure S3),^7z,8^ [η^5^-1,2,4-(Me_3_Si)_3_C_5_H_2_]_2_Th=N­(*p*-tolyl)­(bipy) (Figure S4),^7y,8^ and **5** immediately react. In this case, although the specific reactivity patterns may vary, the overall reactivity is facilitated by the intrinsic steric properties of the thorium imido complexes and by the steric characteristics of the substrates employed. In analogy to imidos [η^5^-1,2,4-(Me_3_C)_3_C_5_H_2_]_2_Th=N­(*p*-tolyl) and (η^5^-C_5_Me_5_)_2_Th=N­(mesityl)­(dmap) (Figures S1 and S2),^7c,7,8^ **5** reacts with carbodiimides (RN=)_2_C to form the [2 + 2] cycloaddition products (η^5^-C_5_Me_5_)_2_Th­[N­(*p*-tolyl)­C­(=NR)­N­(R)]­(dmap) (R = C_6_H_11_ (**21**), *^i^* Pr (**22**)) besides one molecule of dmap (Scheme 7). This reactivity is contrary to [η^5^-1,3-(Me_3_C)_2_C_5_H_3_]_2_Th=N­(dipp)­(dmap) forming a four-membered metallaheterocycle [η^5^-1,3-(Me_3_C)_2_C_5_H_3_]_2_Th­[N­(C_6_H_11_)­C­(=Ndipp)­N­(C_6_H_11_)] (Figure S3),^7z,8^ in which [1,3]-Th migration occurs for the [2 + 2] addition intermediate,^7z^ a process driven by the greater steric hindrance of the *^i^* Pr_2_C_6_H_3_N moiety compared to mesityl and *p*-tolyl groups. The molecular structure of **21** is shown in Figure 20, whereas the molecular structure of **22** is provided in the Supporting Information. In complex **21**, the Th–N (1), Th–N(3) and Th–N(5) distances are 2.658(4), 2.377(4) and 2.373(4) Å, respectively, which are comparable to those found in **22** (2.613(6), 2.365(5) and 2.344(6) Å, respectively).

**Figure 20:**
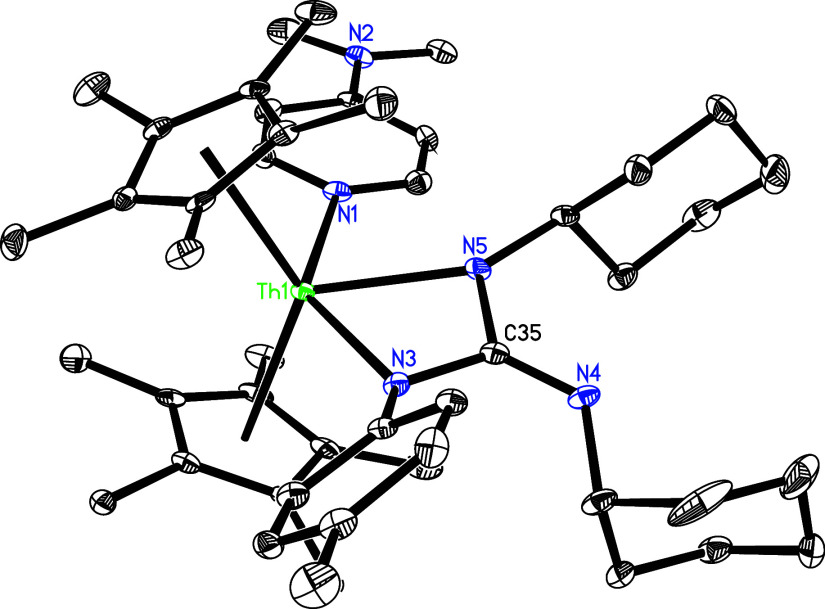
Molecular structure of **21** (thermal ellipsoids drawn at the 35% probability level).

Moreover, complex **5** reacts with ketones. For example, when **5** is treated with 1 equiv of Ph_2_CO, it yields the [2 + 2] cycloaddition product (η^5^-C_5_Me_5_)_2_Th­[N­(*p*-tolyl)­CPh_2_O]­(dmap) (**23**) with a concomitant loss of one dmap (Scheme 8). The disparate reactivity observed–where [η^5^-1,2,4-(Me_3_C)_3_C_5_H_2_]_2_Th=N­(*p*-tolyl) forms a dmap supported terminal oxido product [η^5^-1,2,4-(Me_3_C)_3_C_5_H_2_]_2_Th=O­(dmap) in the presence of dmap (Figure S1),^7a,8^ (η^5^-C_5_Me_5_)_2_Th=N­(mesityl)­(dmap) leads to a four-membered metallaheterocycle (η^5^-C_5_Me_5_)_2_Th­[OCPh_2_O]­(dmap) (Figure S2),^7g,8^ complex **5** gives a [2 + 2] cycloaddition product (η^5^-C_5_Me_5_)_2_Th­[N­(*p*-tolyl)­CPh_2_O]­(dmap) (**23**), and [η^5^-1,3-(Me_3_C)_2_C_5_H_3_]_2_Th=N­(dipp)­(dmap) yields a dimeric oxido complex {[η^5^-1,3-(Me_3_C)_2_C_5_H_3_]_2_Th}_2_(μ-O)_2_ (Figure S3),^7z,8^ –can be ascribed to differences in steric protection of the metal atom imposed by the coordinated ligands. In this context, complex **5** gives the [2 + 2] cycloaddition product (η^5^-C_5_Me_5_)_2_Th­[N­(*p*-tolyl)­CPh_2_O]­(dmap) (**23**). This behavior reflects the lower steric bulk of the C_5_Me_5_ ligand and *p*-tolyl substituent relative to the 1,2,4-(Me_3_C)_3_C_5_H_2_ ligand, mesityl, or 2,6-*^i^* Pr_2_C_6_H_3_ groups. The molecular structure of **23**, depicted in Figure 21 and detailed in Table 1, features a relatively long Th–N (1) distance of 2.608(3) Å, indicative of a datively coordinated nitrogen atom. The Th–N(3) distance is 2.436(3) Å, whereas the Th–O (1) distance is 2.206(3) Å. Nevertheless, complex **23** is unstable and readily eliminates Ph_2_C=N­(*p*-tolyl) to form the monomeric terminal oxido (η^5^-C_5_Me_5_)_2_Th=O­(dmap). This intermediate further reacts with a second molecule of Ph_2_CO to produce the known four-membered metallaheterocycle (η^5^-C_5_Me_5_)_2_Th­[OCPh_2_O]­(dmap) (**24**) (Scheme 8). Complex **24** can also be accessed directly through the reaction of **5** with 2 equiv of Ph_2_CO (Scheme 8). In a similar manner, a cycloaddition–elimination reaction occurs when **5** reacts with Ph_2_C=S, furnishing the monomeric terminal sulfido (η^5^-C_5_Me_5_)_2_Th=S­(dmap), which immediately reacts with a second molecule of Ph_2_CS to yield the four-membered metallaheterocycle (η^5^-C_5_Me_5_)_2_Th­[SCPh_2_S]­(dmap) (**25**) (Scheme 8). The differences in reactivity between [η^5^-1,2,4-(Me_3_C)_3_C_5_H_2_]_2_Th=N­(*p*-tolyl) (Figure S1),^7a,8^ [η^5^-1,2,4-(Me_3_Si)_3_C_5_H_2_]_2_Th=N­(*p*-tolyl)­(bipy) (Figure S4)^7y,8^ and **5** forming the four-membered metallaheterocycle [η^5^-1,2,4-(Me_3_E)_3_C_5_H_2_]_2_Th­(SCPh_2_S) (E = C,^7a^ Si^7y^) and (η^5^-C_5_Me_5_)_2_Th­[SCPh_2_S]­(dmap) (**25**), respectively, and [η^5^-1,3-(Me_3_C)_2_C_5_H_3_]_2_Th=N­(dipp)­(dmap) yielding a dimeric sulfido complex {[η^5^-1,3-(Me_3_C)_2_C_5_H_3_]_2_Th}_2_(μ-S)_2_ (Figure S3),^7z,8^ can also be attributed to the different steric protection of the metal atom exerted by the coordinated ligands, in which [η^5^-1,3-(Me_3_C)_2_C_5_H_3_]_2_Th fragment is not sufficiently sterically protected to prevent dimerization. The molecular structure of **25** is shown in Figure 22, and selected bond distances and angles are listed in Table 1. The Th–S (1) and Th–S(2) distances are 2.766 (1) and 2.764 (1) Å, whereas the Th–N (1) distance of 2.618(4) Å is indicative of a datively coordinated nitrogen atom.

**Figure 21:**
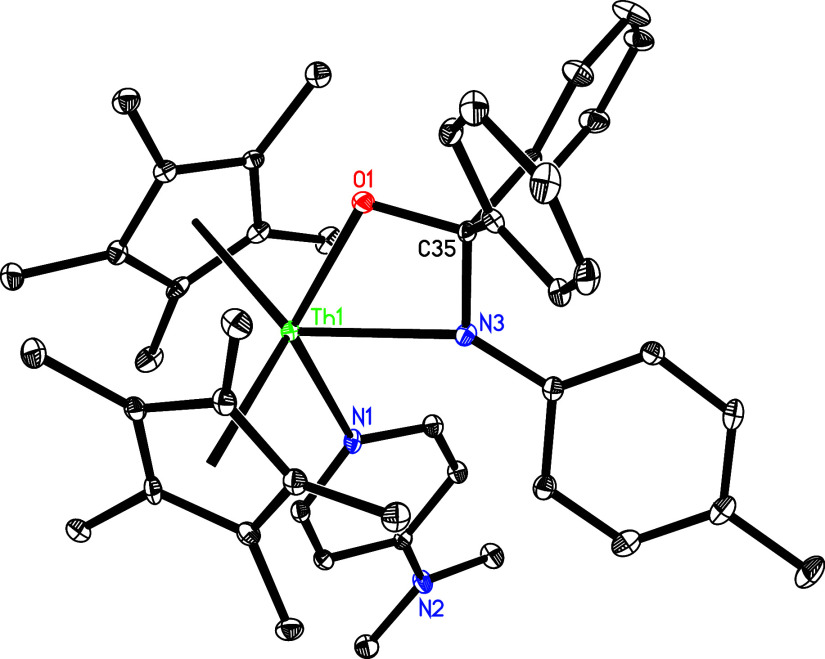
Molecular structure of **23** (thermal ellipsoids drawn at the 35% probability level).

**Scheme 8:**
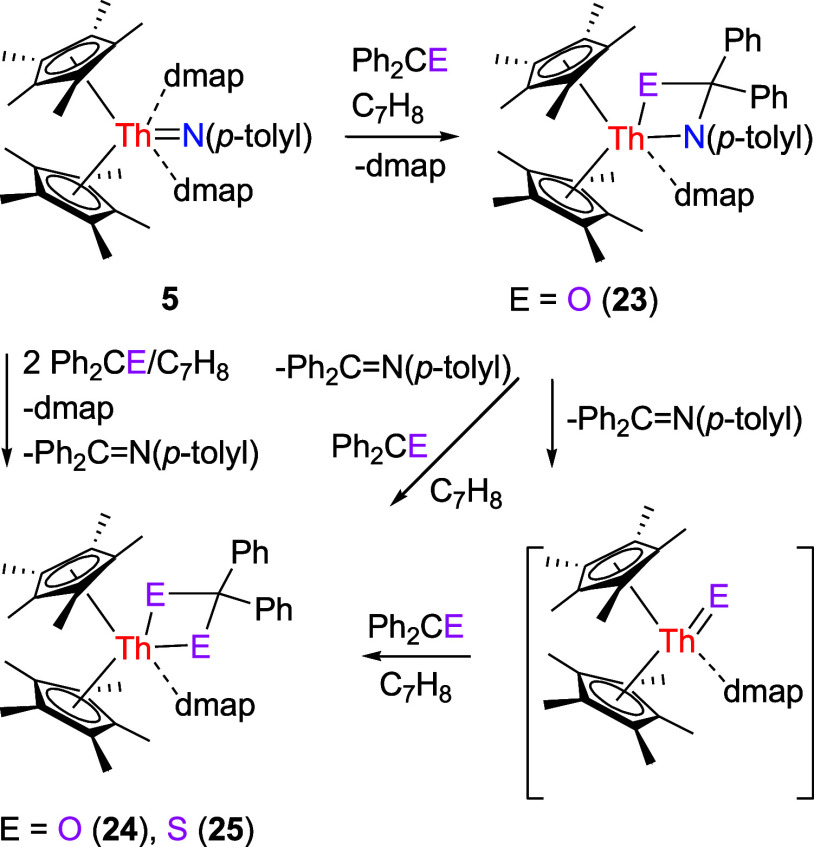
Synthesis of Compounds **23**–**25**

**Figure 22:**
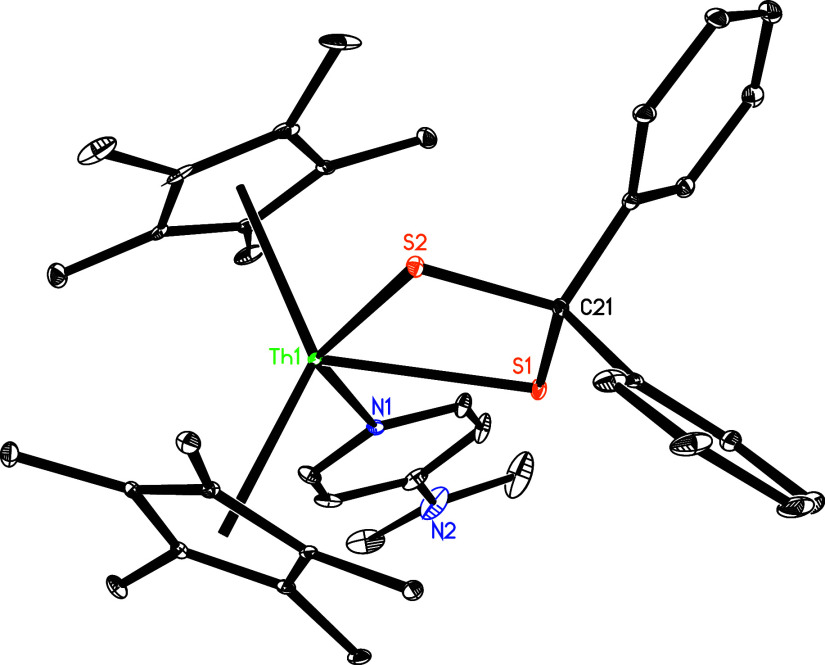
Molecular structure of **25** (thermal ellipsoids drawn at the 35% probability level).

However, the four-membered metallaheterocycle (η^5^-C_5_Me_5_)_2_Th­[SC=N­(*p*-tolyl)­NPh]­(dmap) (**26**) is isolated from the reaction of complex **5** and PhNCS, with one dmap being released (Scheme 9), where no cycloaddition–elimination reaction is observed. By analogy to the reactivity of (η^5^-C_5_Me_5_)_2_Th=N­(mesityl)­(dmap) (Figure S2),^7g,8^ formation of **26** may be explained by an initial [2 + 2] cycloaddition of **5** with PhNCS, followed by a [1,3]-Th migration (Scheme 9). The molecular structure of **26** is shown in Figure 23, with selected bond distances and angles provided in Table 1. The relatively long Th–N(3) distance of 2.633(7) Å is indicative of a datively coordinated nitrogen atom. The Th–N (1) and Th–S (1) distances measure 2.416(14) and 2.780(5) Å, respectively. In a related reaction, **5** reacts with CS_2_ with concomitant dmap loss to initially form a [2 + 2] cycloaddition complex, which then dimerizes to yield [(η^5^-C_5_Me_5_)_2_Th]_2_{μ-[N­(*p*-tolyl)­C­(S)­S]}_2_ (**27**). The molecular structure of **27** is illustrated in Figure 24, and Table 1 lists the selected bond distances and angles. The Th(1)–S (1), Th(1)–S­(1A) and Th(1)–S­(2A) distances are 2.982 (1), 3.039 (1) and 2.889 (1) Å, respectively, whereas the Th(1)–N (1) distance is 2.597(5) Å. Nevertheless, these reactivity patterns are in contrast to those observed for [η^5^-1,2,4-(Me_3_C)_3_C_5_H_2_]_2_Th=N­(*p*-tolyl), (η^5^-C_5_Me_5_)_2_Th=N­(mesityl)­(dmap) and [η^5^-1,3-(Me_3_C)_2_C_5_H_3_]_2_Th=N­(dipp)­(dmap) (Figures S1–S3).^7a,7,8^ Specifically, the thorium imido complex [η^5^-1,2,4-(Me_3_C)_3_C_5_H_2_]_2_Th=N­(*p*-tolyl) reacts with CS_2_ and PhNCS to form the [2 + 2] cycloaddition products [η^5^-1,2,4-(Me_3_C)_3_C_5_H_2_]_2_Th­[N­(*p*-tolyl)­C­(E)-S] (E = S, PhN) (Figure S1),^7a,8^ while (η^5^-C_5_Me_5_)_2_Th=N­(mesityl)­(dmap) affords the four-membered metallaheterocycle (η^5^-C_5_Me_5_)_2_Th­[SC=N­(mesityl)-S]­(dmap) with CS_2_ (Figure S2).^7g,8^ In addition, [η^5^-1,3-(Me_3_C)_2_C_5_H_3_]_2_Th=N­(dipp)­(dmap) gives rise to the dimer {[η^5^-1,3-(Me_3_C)_2_C_5_H_3_]_2_Th}_2_(μ-S)_2_ via the reaction with both CS_2_ and PhNCS (Figure S3).^7z,8^ These differences can also be explained based on the steric arguments outlined above.

**Figure 23:**
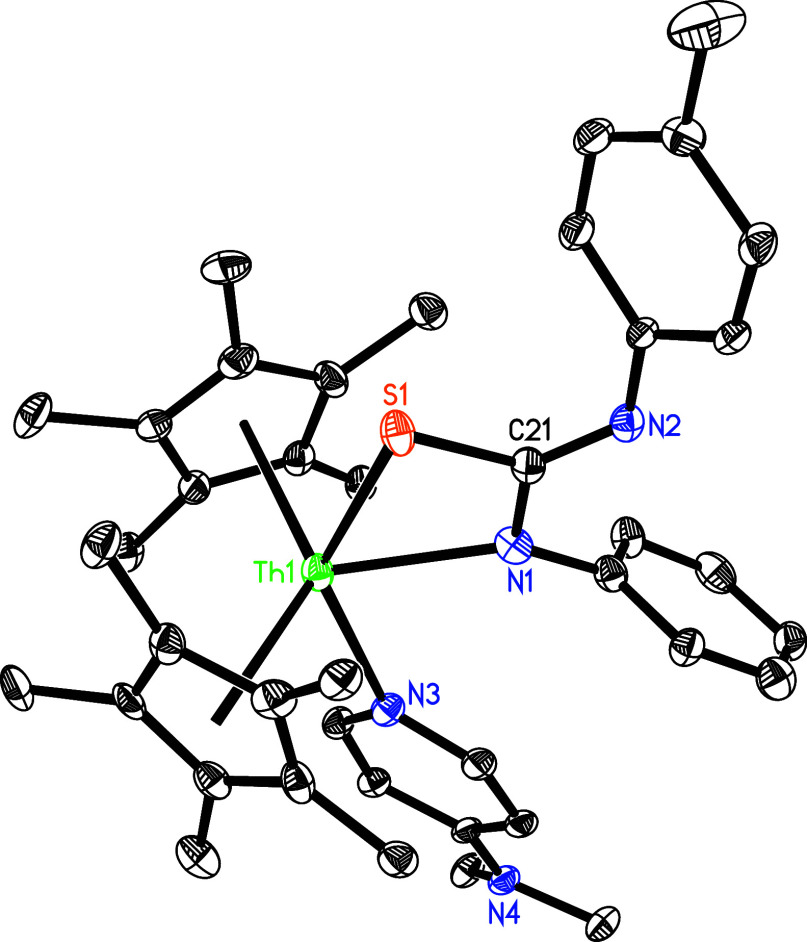
Molecular structure of **26** (thermal ellipsoids drawn at the 35% probability level).

**Scheme 9:**
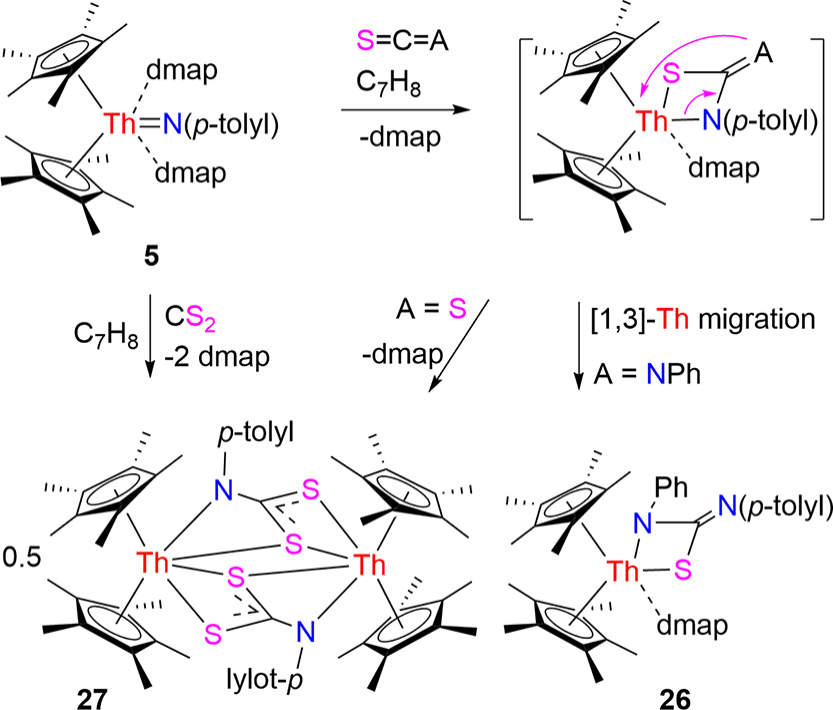
Synthesis of Compounds **26** and **27**

**Figure 24:**
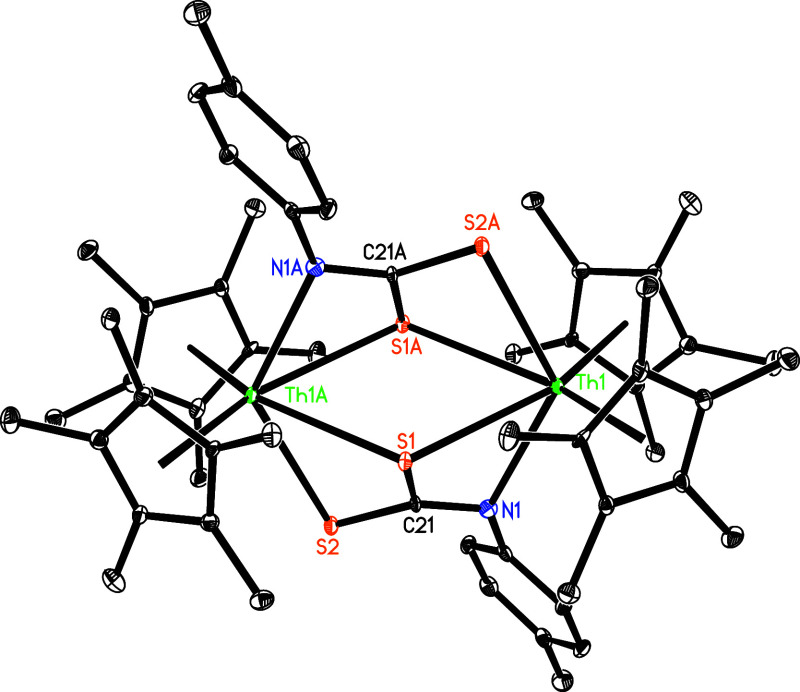
Molecular structure of **27** (thermal ellipsoids drawn at the 35% probability level).

Furthermore, when complex **5** is exposed to esters such as methyl methacrylate (MMA), a cycloaddition–elimination is not observed. Instead, as seen in the reactivity of [η^5^-1,2,4-(Me_3_C)_3_C_5_H_2_]_2_Th=N­(*p*-tolyl) (Figure S1),^7e,8^ the six-membered metallaheterocycle (η^5^-C_5_Me_5_)_2_Th­[N­(*p*-tolyl)­CH_2_C­(Me)=C­(OMe)­O]­(dmap) (**28**) is furnished in quantitative conversion with the loss of one dmap molecule (Scheme 10). In this process, the Th=N­(*p*-tolyl) moiety serves as a nucleophile, undergoing a Michael addition reaction with MMA. This outcome contrasts with the formation of the methoxyl amidate complex [η^5^-1,3-(Me_3_C)_2_C_5_H_3_]_2_Th­(OMe)­[OC­{C­(Me)=CH_2_}­N­(dipp)] from [η^5^-1,3-(Me_3_C)_2_C_5_H_3_]_2_Th=N­(dipp)­(dmap) (Figure S3),^7z,8^ due to the reduced steric bulk of the 1,3-(Me_3_C)_2_C_5_H_3_ ligand compared with 1,2,4-(Me_3_C)_3_C_5_H_2_ and C_5_Me_5_ ligands. The molecular structure of **28** is shown in Figure 25, and selected bond distances and angles are summarized in Table 1. The relatively long Th–N (1) distance of 2.674(10) Å is indicative of a datively coordinated nitrogen atom. The Th–N(3) distance is 2.336(10) Å, whereas the Th–O (1) distance is 2.271(8) Å. Moreover, the reaction of **5** with *rac*-lactide does not yield a [2 + 2] cycloaddition product. Instead, the dimeric complex [(η^5^-C_5_Me_5_)_2_Th]_2_{μ-[OCH­(Me)­C­(=O)­OCH­(Me)­C­(=N-*p*-tolyl)­O]}_2_ (**29**) is formed along with dmap release (Scheme 10). This behavior contrasts with that of (η^5^-C_5_Me_5_)_2_Th=N­(mesityl)­(dmap) and [η^5^-1,3-(Me_3_C)_2_C_5_H_3_]_2_Th=N­(dipp)­(dmap) (Figures S2 and S3),^7g,7,8^ which give the eight-membered metallaheterocycles (η^5^-C_5_Me_5_)_2_Th­[OCH­(Me)­C­(=O)­OCH­(Me)­C­(=Nmesityl)­O] and [η^5^-1,3-(Me_3_C)_2_C_5_H_3_]_2_Th­[OCH­(Me)­C­(=O)­OCH­(Me)­C­(=Ndipp)­O], respectively, again, presumably due to less steric hindrance imposed by the *p*-tolyl group compared with mesityl and 2,6-*^i^* Pr_2_C_6_H_3_ groups. The molecular structure of **29** is presented in Figure 26, with selected bond distances and angles detailed in Table 1. The relatively long Th(1)–O­(3A) distance of 2.695(5) Å is consistent with a datively coordinated oxygen atom, while the Th(1)–O (1) distance of 2.491(5) Å is longer than Th(1)–O­(4A) (2.205(5) Å), likely a consequence of the steric repulsion between the *p*-tolyl group and C_5_Me_5_ ligand. Moreover, the asymmetry in the Th(1)–O (1) (2.491(5) Å) vs Th(1)–N (1) (2.562(6) Å) bond distances suggests that the negative charge is predominantly localized on the O atom of the amidate fragment. Similarly, as observed in the reactivity of [η^5^-1,3-(Me_3_C)_2_C_5_H_3_]_2_Th=N­(dipp)­(dmap) (Figure S3),^7z,8^ treatment of **5** with 2 equiv of the amidate PhCONH­(*p*-tolyl) prompts deprotonation of the N–H moiety to yield the bis-amidate (η^5^-C_5_Me_5_)_2_Th­[OC­(Ph)­N­(*p*-tolyl)]_2_ (**30**) in quantitative conversion, with concomitant release of dmap and *p*-tolylNH_2_ (Scheme 10). The molecular structure of **30** is shown in Figure 27, and selected bond distances and angles are summarized in Table 1. The Th–O bonds (Th–O (1) at 2.462(2) Å and Th–O(2) at 2.452(2) Å) are essentially identical, as are the Th–N bonds (Th–N (1) at 2.557(3) Å and Th–N(2) at 2.565(3) Å). Once again, the discrepancies in the Th–O and Th–N bond lengths reinforce the notion that the negative charge is mainly localized on the O atom of the amidate fragment.

**Scheme 10:**
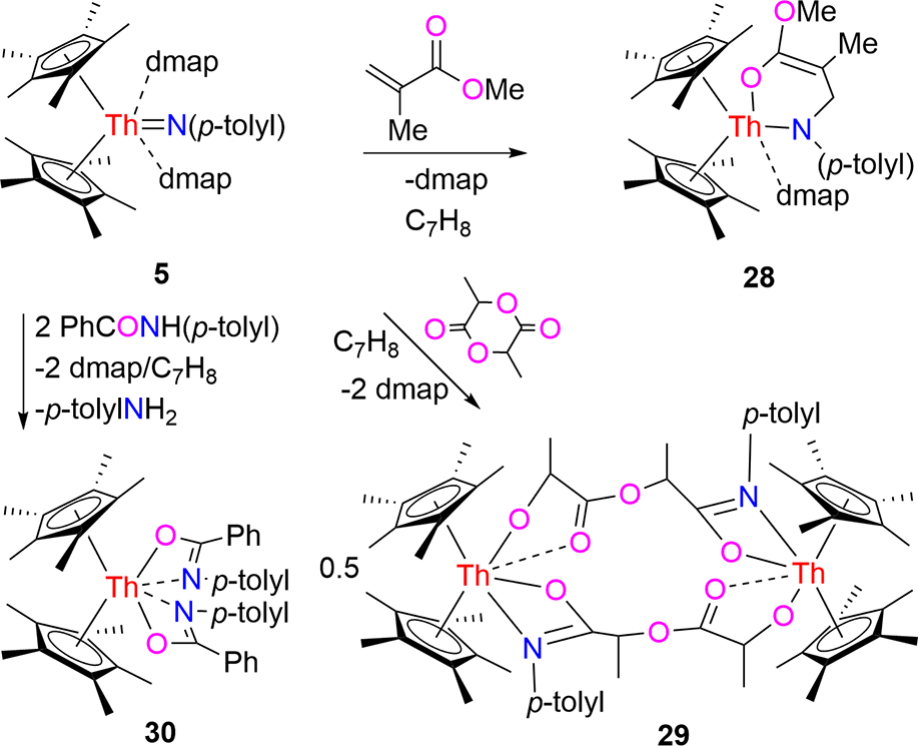
Synthesis of Compounds **28**–**30**

**Figure 25:**
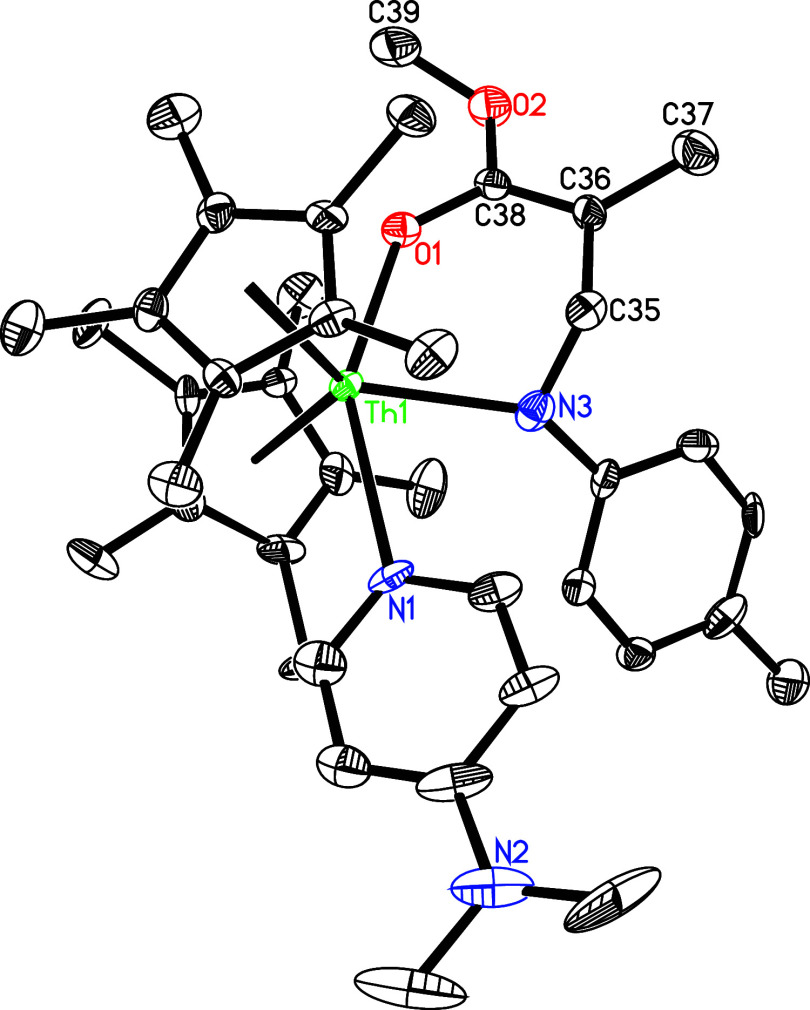
Molecular structure of **28** (thermal ellipsoids drawn at the 35% probability level).

**Figure 26:**
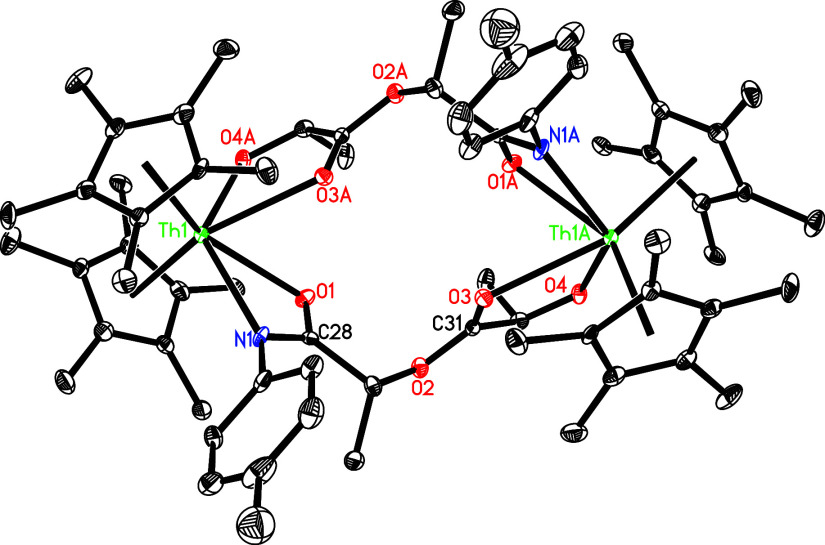
Molecular structure of **29** (thermal ellipsoids drawn at the 35% probability level).

**Figure 27:**
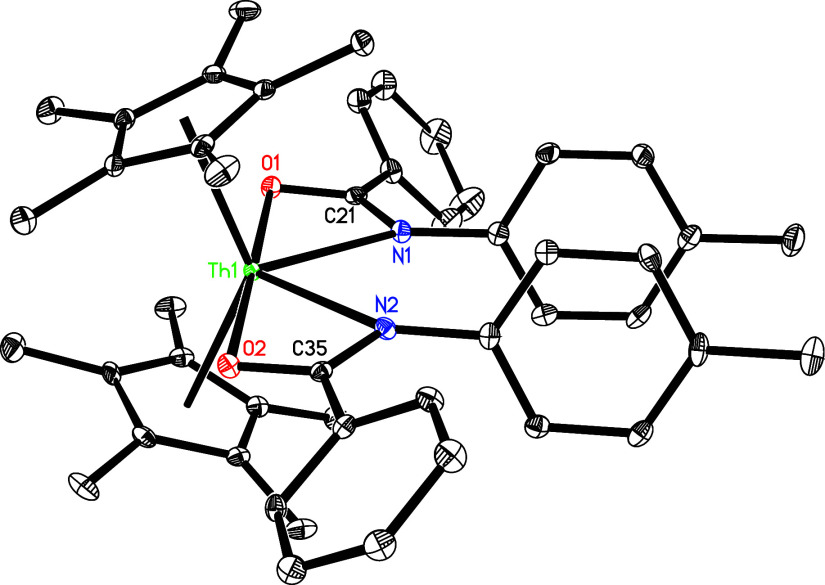
Molecular structure of **30** (thermal ellipsoids drawn at the 35% probability level).

### Reaction with Organic Nitriles and Isonitriles

Moreover, in analogy to the imido complexes [η^5^-1,2,4-(Me_3_C)_3_C_5_H_2_]_2_Th=N­(*p*-tolyl) (Figure S1),^7b,8^ (η^5^-C_5_Me_5_)_2_Th=N­(mesityl)­(dmap) (Figure S2),^7g,8^ [η^5^-1,3-(Me_3_C)_2_C_5_H_3_]_2_Th=N­(dipp)­(dmap) (Figure S3),^7z,8^ and [η^5^-1,2,4-(Me_3_Si)_3_C_5_H_2_]_2_Th=N­(*p*-tolyl)­(bipy) (Figure S4),^7y,8^ complex **5** also react with organic nitriles. For example, contrary to the reaction of PhCN with [η^5^-1,2,4-(Me_3_C)_3_C_5_H_2_]_2_Th=N­(*p*-tolyl), (η^5^-C_5_Me_5_)_2_Th=N­(mesityl)­(dmap) and [η^5^-1,3-(Me_3_C)_2_C_5_H_3_]_2_Th=N­(dipp)­(dmap) forming a [2 + 2] cycloaddition product [η^5^-1,2,4-(Me_3_C)_3_C_5_H_2_]_2_Th­[N­(*p*-tolyl)­C­(Ph)=N], a dmap imido adduct (η^5^-C_5_Me_5_)_2_Th­[=NC­(Ph)=N­(mesityl)]­(dmap) and an amido pyridyl complex [η^5^-1,3-(Me_3_C)_2_C_5_H_3_]_2_Th­[NHC­(Ph)=Ndipp]­[κ^2^-*C*,*N*-4-(Me_2_N)­C_5_H_3_N] (Figures S1–S3),^7b,7,8^ respectively, reaction of **5** with 1 equiv of PhCN yields free dmap and crystals of both [2 + 2] cycloaddition product (η^5^-C_5_Me_5_)_2_Th­[N­(*p*-tolyl)­C­(Ph)=N]­(dmap) (**31a**) and amidinyl pyridyl complex (η^5^-C_5_Me_5_)_2_Th­[η^3^-NHC­(Ph)­N­(*p*-tolyl)]­[κ^2^-*C*,*N*-4-(Me_2_N)­C_5_H_3_N] (**31b**) (Scheme 11). These variations can also be attributed to different degrees of steric hindrance imposed on the thorium atom by the coordinated ligands, as previously described. Although both complexes **31a** and **31b** are isolable in the solid state, only the amidinyl pyridyl complex **31b** is detected in the ^1^H NMR spectrum (recorded in C_7_D_8_ solution between 20 and 100 °C). This observation suggests that the equilibrium is strongly shifted in favor of **31b**. Moreover, DFT investigations indicate that the formation of **31b** proceeds via an α-H transfer from dmap to the Th­[N­(*p*-tolyl)­C­(Ph)=N] moiety through transition state **TS31** (Figure 28). The conversion of **31b** from **31a** is slightly exergonic (Δ*G*(298 K) = −2.1 kcal/mol), but faces a reaction barrier of Δ*G* ^‡^(298 K) = 21.8 kcal/mol, which implies that if an equilibrium between **31a** and **31b** exists in the solution, it is ultimately shifted toward **31b**–consistent with the NMR findings. The molecular structures of **31a** and **31b** are illustrated in Figures 29 and 30, respectively, with selected bond distances and angles provided in Table 1. In complex **31a**, the relatively long Th–N(3) distance of 2.650(5) Å is consistent with a datively coordinated nitrogen atom, whereas the Th–N (1) distance of 2.427(11) Å is longer than Th–N(2) (2.276(10) Å), likely attributed to the increased steric hindrance from the *p*-tolyl group and C_5_Me_5_ ligand. In contrast, complex **31b** exhibits that the Th–N (1), Th–N(2), and Th–N(3) distances are 2.503(4), 2.522(5), and 2.512(4) Å, respectively, with a Th–C(35) distance of 2.479(5) Å. Under similar reaction conditions, contrary to the reactivity of [η^5^-1,2,4-(Me_3_C)_3_C_5_H_2_]_2_Th=N­(*p*-tolyl),^7b^ treatment of **5** with 3 equiv of PhCN yields the eight-membered complex (η^5^-C_5_Me_5_)_2_Th­[η^7^-N­(*p*-tolyl)­C­(Ph)­NC­(Ph)­NC­(Ph)­N] (**32**) with dmap elimination (Scheme 11), ostensibly due to the less steric hindrance of the C_5_Me_5_ ligand compared with the 1,2,4-(Me_3_C)_3_C_5_H_2_ ligand. The molecular structure of **32** is shown in Figure 31, with selected bond distances and angles provided in Table 1. The Th–N (1) distance of 2.647(5) Å is longer than Th–N(2) (2.463(5) Å) and Th–N(4) (2.246(5) Å), again reflecting the steric interference between the *p*-tolyl group and C_5_Me_5_ ligand.

**Scheme 11:**
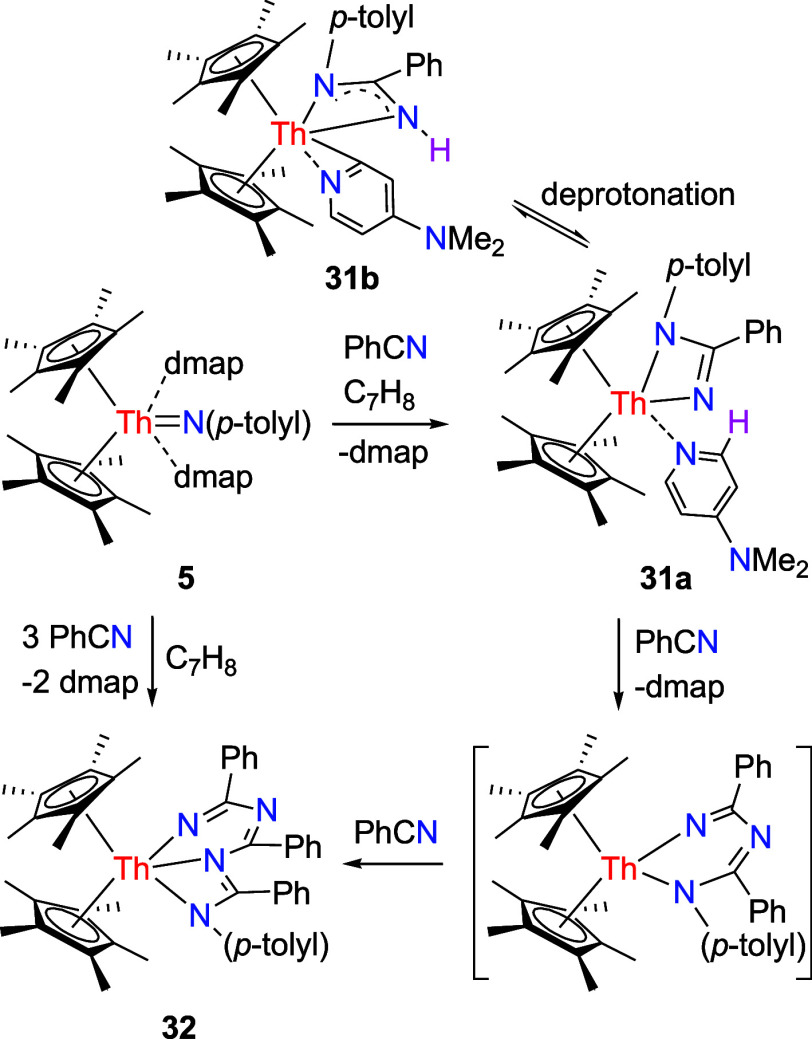
Synthesis of Compounds **31** and **32**

**Figure 28:**
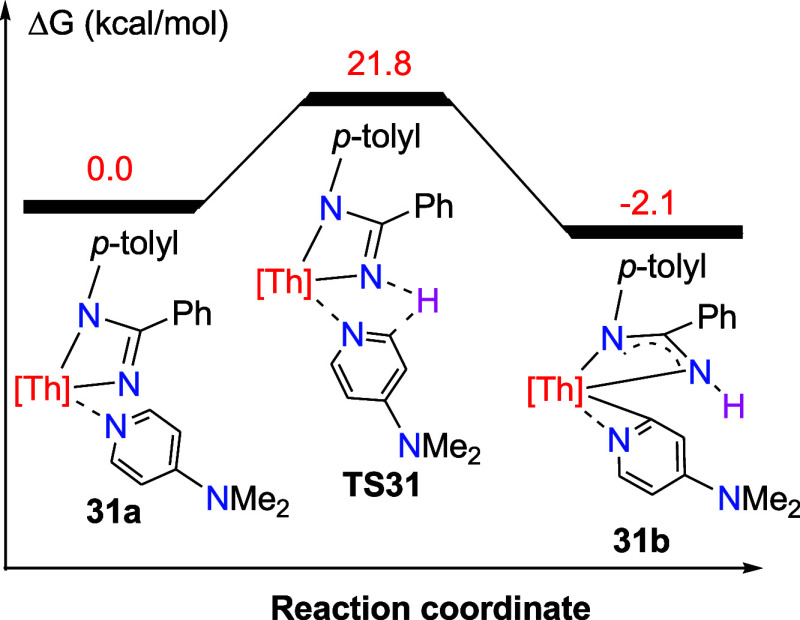
Free energy profile (kcal/mol) for the reaction of **31a** ⇌ **31b**. [Th] = (η^5^-C_5_Me_5_)_2_Th.

**Figure 29:**
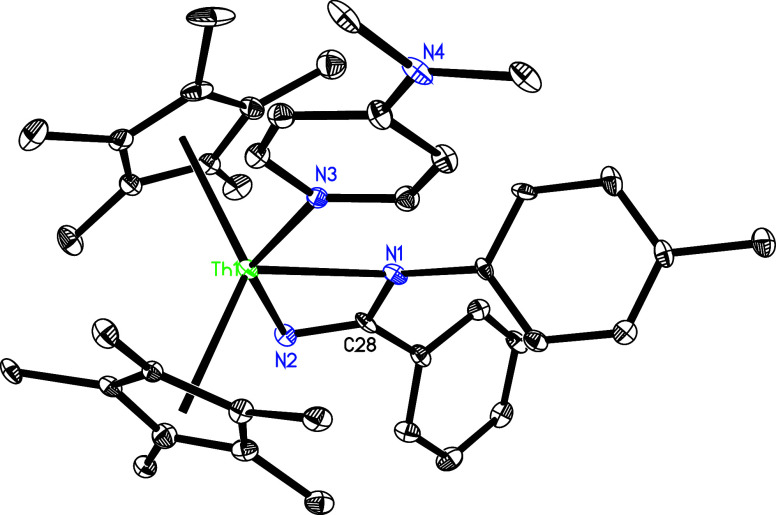
Molecular structure of **31a** (thermal ellipsoids drawn at the 35% probability level).

**Figure 30:**
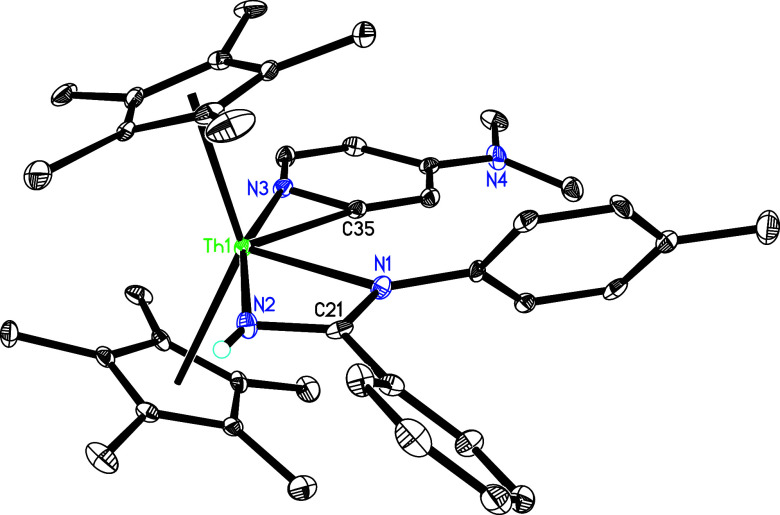
Molecular structure of **31b** (thermal ellipsoids drawn at the 35% probability level).

**Figure 31:**
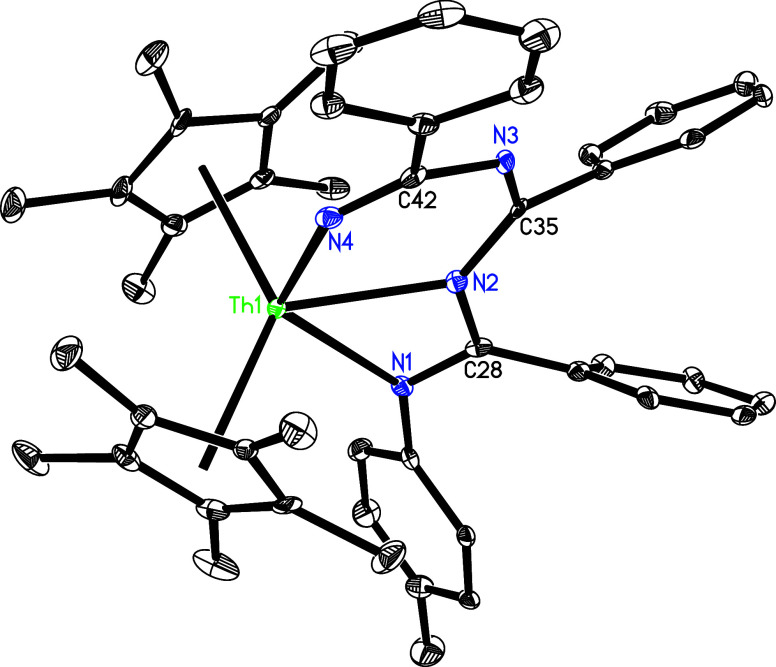
Molecular structure of **32** (thermal ellipsoids drawn at the 35% probability level).

Moreover, the thorium imido complexes [η^5^-1,3-(Me_3_C)_2_C_5_H_3_]_2_Th=N­(dipp)­(dmap) (Figure S3),^7z,8^ [η^5^-1,2,4-(Me_3_Si)_3_C_5_H_2_]_2_Th=N­(*p*-tolyl)­(bipy) (Figure S4)^7y,8^ and **5** exhibit identical reactivity toward benzyl nitrile PhCH_2_CN. In the case of **5**, its reaction with PhCH_2_CN leads to the amidinyl iminato complex (η^5^-C_5_Me_5_)_2_Th­[η^3^-N­(*p*-tolyl)­C­(CH_2_Ph)­NH]­(N=C=CHPh) (**33**) in quantitative conversion with concurrent dmap elimination (Scheme 12). We propose that complex **5** first undergoes a [2 + 2] cycloaddition with PhCH_2_CN, releasing dmap and generating a four-membered intermediate. This intermediate then reacts with a second molecule of PhCH_2_CN via deprotonation of the benzylic C–H bond, ultimately affording complex **33** (Scheme 12). A similar pathway occurs when **5** is exposed to Ph_2_CHCN, yielding the amidinyl iminato complex (η^5^-C_5_Me_5_)_2_Th­[η^3^-N­(*p*-tolyl)­C­(CHPh_2_)­NH]­(N=C=CPh_2_) (**34**) in quantitative conversion with the loss of dmap (Scheme 12). This reactivity is contrary to [η^5^-1,3-(Me_3_C)_2_C_5_H_3_]_2_Th=N­(dipp)­(dmap) giving a mono-Cp amido iminato complex [η^5^-1,3-(Me_3_C)_2_C_5_H_3_]­Th­(NHdipp)­(N=C=CPh_2_)_2_(dmap)_2_ and [η^5^-1,2,4-(Me_3_Si)_3_C_5_H_2_]_2_Th=N­(*p*-tolyl)­(bipy) affording an μ-imido-bridged complex {[η^5^-1,2,4-(Me_3_Si)_3_C_5_H_2_]­Th­(N=N=CPh_2_)­(bipy)}_2_[μ-N­(*p*-tolyl)]_2_ with Ph_2_CHCN (Figures S3 and S4),^7y,7,8^ but similar to (η^5^-C_5_Me_5_)_2_Th=N­(mesityl)­(dmap) forming an iminato complex (η^5^-C_5_Me_5_)_2_Th­[N­(mesityl)­C­(CHPh_2_)­NH]­(N=C=CPh_2_) with Ph_2_CHCN (Figure S2),^7g,8^ presumably due to the steric effect of the C_5_Me_5_ ligand. The molecular structure of **33** is shown in Figure 32, whereas the molecular structure of **34** is provided in the Supporting Information. The Th–N distances observed in **33** (2.466(3), 2.440(3) and 2.430(3) Å) are comparable to those found in **34** (2.461(10), 2.468(11) and 2.411(11) Å).

**Scheme 12:**
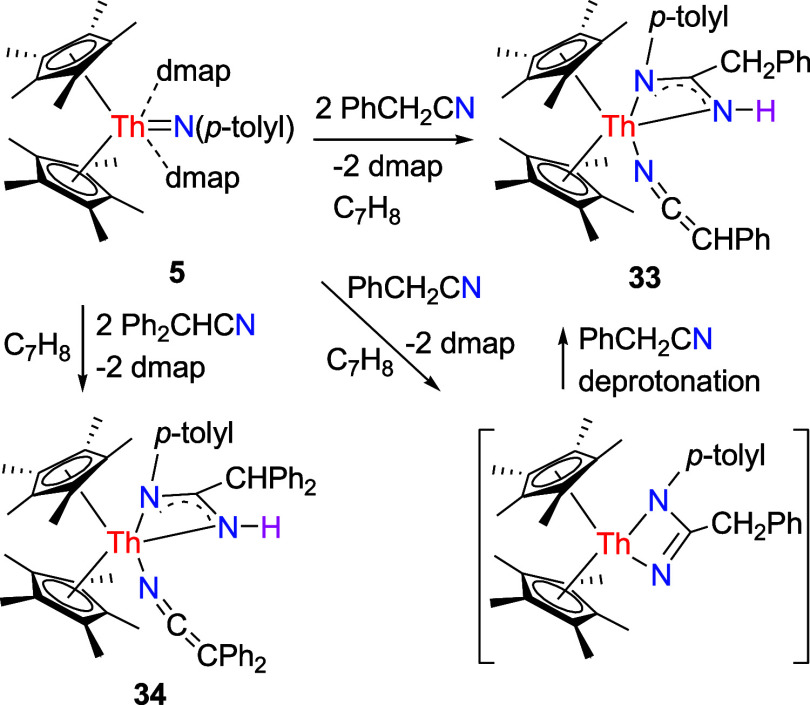
Synthesis of Compounds **33** and **34**

**Figure 32:**
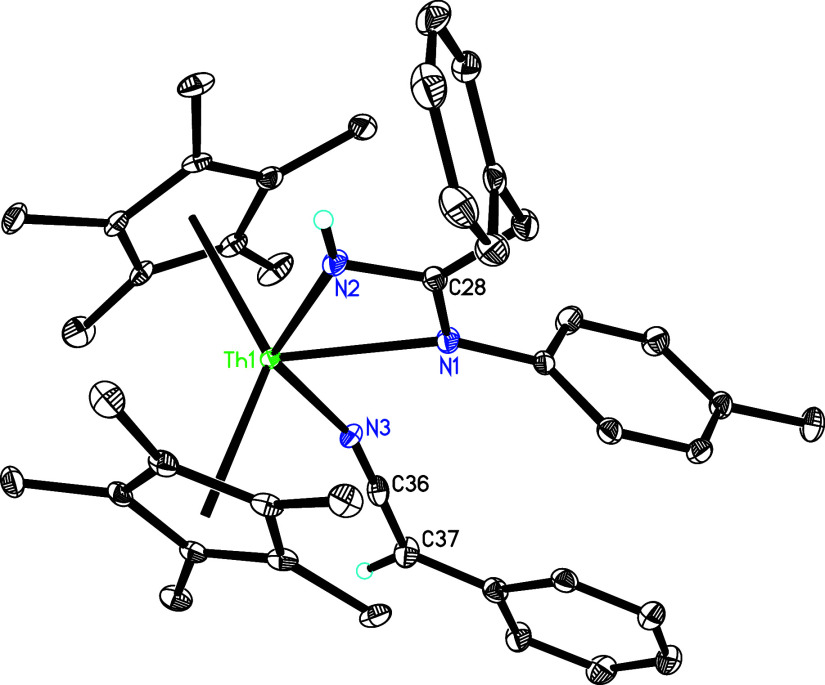
Molecular structure of **33** (thermal ellipsoids drawn at the 35% probability level).

Moreover, analogously to the thorium imido complexes [η^5^-1,2,4-(Me_3_C)_3_C_5_H_2_]_2_Th=N­(*p*-tolyl) (Figure S1),^7h,8^ [η^5^-1,3-(Me_3_C)_2_C_5_H_3_]_2_Th=N­(dipp)­(dmap) (Figure S3),^7z,8^ and [η^5^-1,2,4-(Me_3_Si)_3_C_5_H_2_]_2_Th=N­(*p*-tolyl)­(bipy) (Figure S4),^7y,8^ complex **5** also reacts with organic isonitriles. However, unlike the reaction of 2,6-Me_2_C_6_H_3_NC with [η^5^-1,2,4-(Me_3_C)_3_C_5_H_2_]_2_Th=N­(*p*-tolyl) forming an amido complex [η^5^-1,2,4-(Me_3_C)_3_C_5_H_2_]­[η^5^-1-(2,6-Me_2_C_6_H_3_N=CCH_2_Me_2_C)-3,4-(Me_3_C)_2_C_5_H_2_]­ThNH­(*p*-tolyl) and with [η^5^-1,2,4-(Me_3_Si)_3_C_5_H_2_]_2_Th=N­(*p*-tolyl)­(bipy) affording an eight-membered heterocyclic compound [η^5^-1,2,4-(Me_3_Si)_3_C_5_H_2_]_2_Th­[N­(*p*-tolyl)­C­(=N-2,6-Me_2_C_6_H_3_)­C­(H)=N­(6-MePh-2-CH_2_)] (Figures S1 and S4),^7h,7,8^ exposure of **5** toward 2,6-Me_2_C_6_H_3_NC in toluene at 40 °C yields the amido pyridyl complex (η^5^-C_5_Me_5_)_2_Th­[N­(*p*-tolyl)=CHN­(2,6-Me_2_C_6_H_3_)]­[κ^2^-*C*,*N*-4-(Me_2_N)­C_5_H_3_N] (**35**) with the elimination of one dmap ligand (Scheme 13). No intermediates were observed by NMR spectroscopy; however, drawing on the reactivity observed for the thorium phosphinidene complex [η^5^-1,2,4-(Me_3_C)_3_C_5_H_2_]_2_Th=P­(2,4,6-*^t^* Bu_3_Ph) with 2,6-Me_2_C_6_H_3_NC,^7h^ we propose that complex **5** initially undergoes a [2 + 1] cycloaddition with 2,6-Me_2_C_6_H_3_NC, with the concomitant loss of one dmap, to give a dmap metallaaziridine adduct (Scheme 13). Subsequent deprotonation of dmap yields complex **35**. The molecular structure of **35** is shown in Figure 33, and selected bond distances and angles appear in Table 1. The relatively long Th–N (1) distance of 2.729(5) Å is indicative of a datively coordinated nitrogen atom. While the Th–N(2) and Th–N(3) distances are 2.513(5) and 2.456(5) Å, respectively, the Th–C(37) distance is 2.496(6) Å. In a separate reaction, treatment of complex **5** with 2,6-Me_2_C_6_H_3_NC at 15 °C in toluene affords an amido pyridyl complex (η^5^-C_5_Me_5_)_2_Th­[NH­(*p*-tolyl)]­[κ^2^-*C*,*N*-4-(Me_2_N)-6-(2,6-Me_2_C_6_H_3_N=CH)­C_5_H_2_N] (**36**) with one dmap lost (Scheme 13), further supporting an equilibrium between **5** and **5′** + dmap in solution. We propose that here, 2,6-Me_2_C_6_H_3_NC first inserts into the pyridyl Th-[κ^2^-*C*,*N*-4-(Me_2_N)­C_5_H_3_N] moiety in **5′** to give an amido alkenyl intermediate. This intermediate subsequently undergoes deprotonation of *the p*-tolylNH group to yield an imido complex, which then converts via further deprotonation of the 4-(Me_2_N)-2-(2,6-Me_2_C_6_H_3_N=CH)­C_5_H_3_N moiety to furnish complex **36** (Scheme 13). The molecular structure of **36** is presented in Figure 34, with Table 1 showing that the Th–N (1) and Th–N(2) distances are 2.334(4) and 2.486(4) Å, respectively, and the Th–C(28) bond measures 2.467(5) Å. Furthermore, insertion of isonitriles–specifically Me_3_CNC and C_6_H_11_NC–leads to the isolation of the amido alkenyl complexes (η^5^-C_5_Me_5_)_2_Th­[NH­(*p*-tolyl)]­[κ^2^-*C*,*N*-2-(Me_3_CNC)-4-(Me_2_N)­C_5_H_3_N] (**37**) and (η^5^-C_5_Me_5_)_2_Th­[NH­(*p*-tolyl)]­[κ^2^-*C*,*N*-2-(C_6_H_11_NC)-4-(Me_2_N)­C_5_H_3_N] (**38**), respectively, each formed with the loss of one dmap (Scheme 14). This observation, once again, supports the presence of an equilibrium between **5** and **5′** + dmap in solution. The molecular structure of **37** is shown in Figure 35, whereas that of **38** is available in the Supporting Information. In complex **37**, the Th–N (1), Th–N(2) and Th–C(28) distances are 2.356(4), 2.494(3) and 2.480(4) Å, respectively – values that are comparable to those found in **38** (2.368(4), 2.465(5) and 2.443(5) Å, respectively).

**Figure 33:**
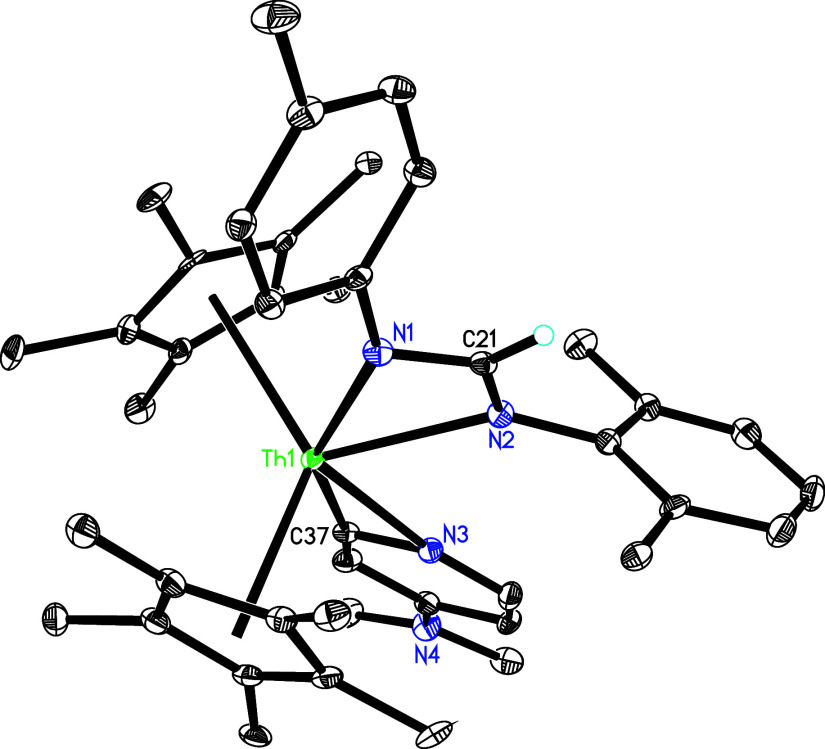
Molecular structure of **35** (thermal ellipsoids drawn at the 35% probability level).

**Scheme 13:**
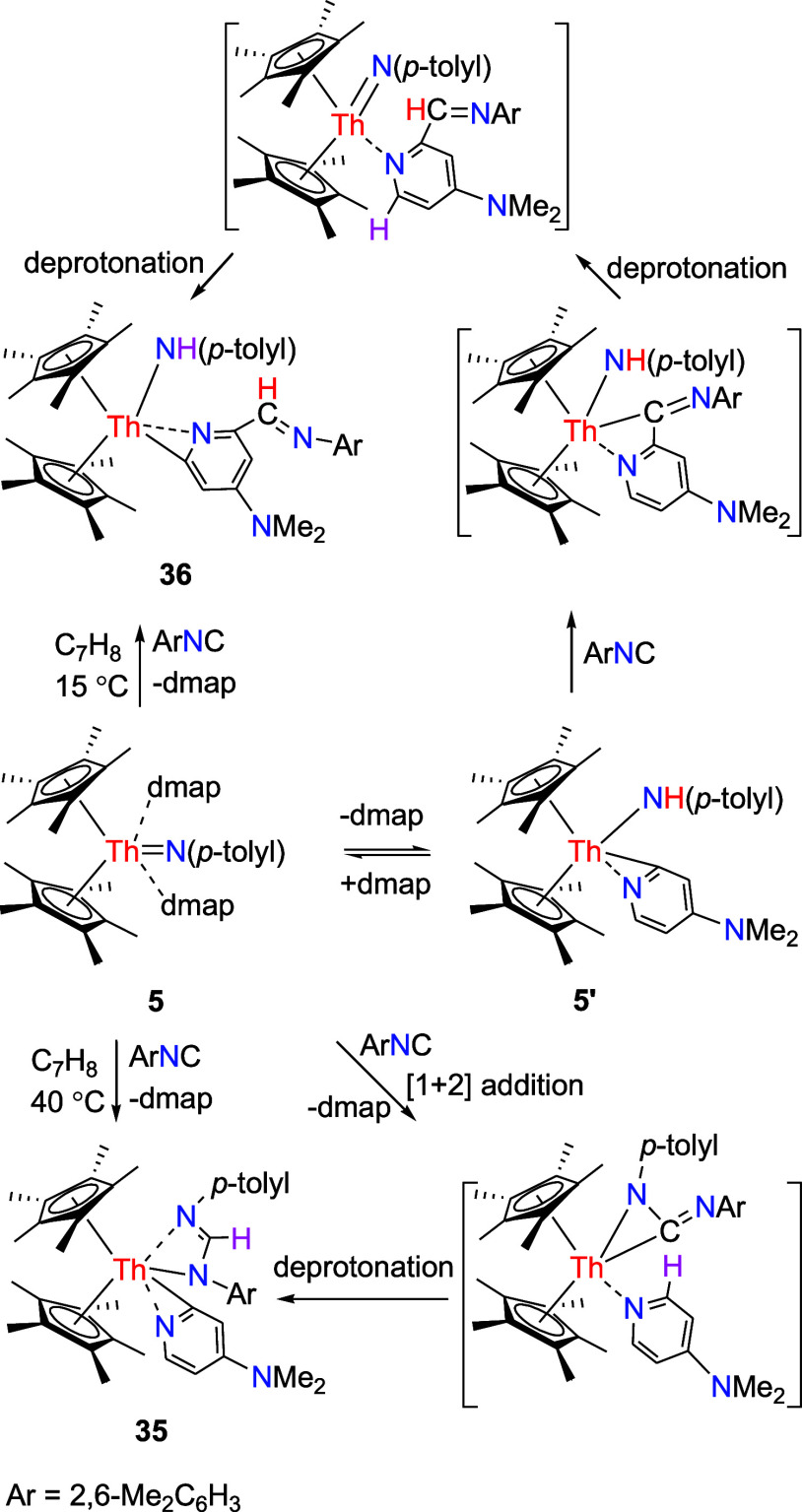
Synthesis of Compounds **35** and **36**

**Figure 34:**
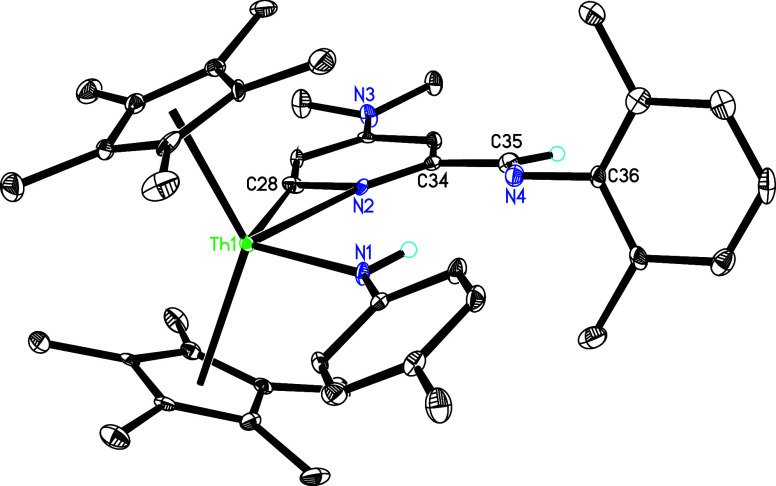
Molecular structure of **36** (thermal ellipsoids drawn at the 35% probability level).

**Figure 35:**
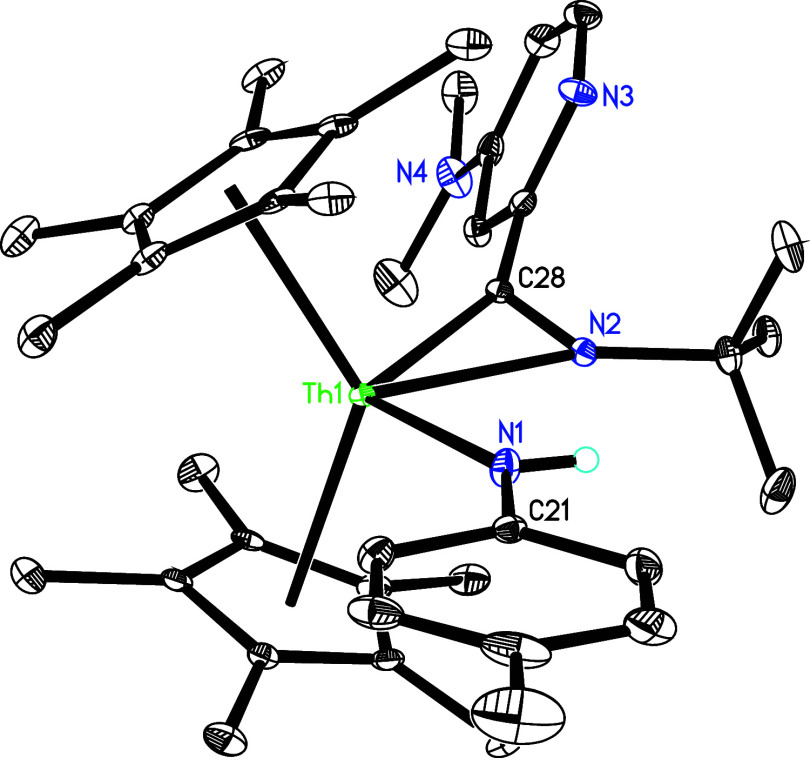
Molecular structure of **37** (thermal ellipsoids drawn at the 35% probability level).

**Scheme 14:**
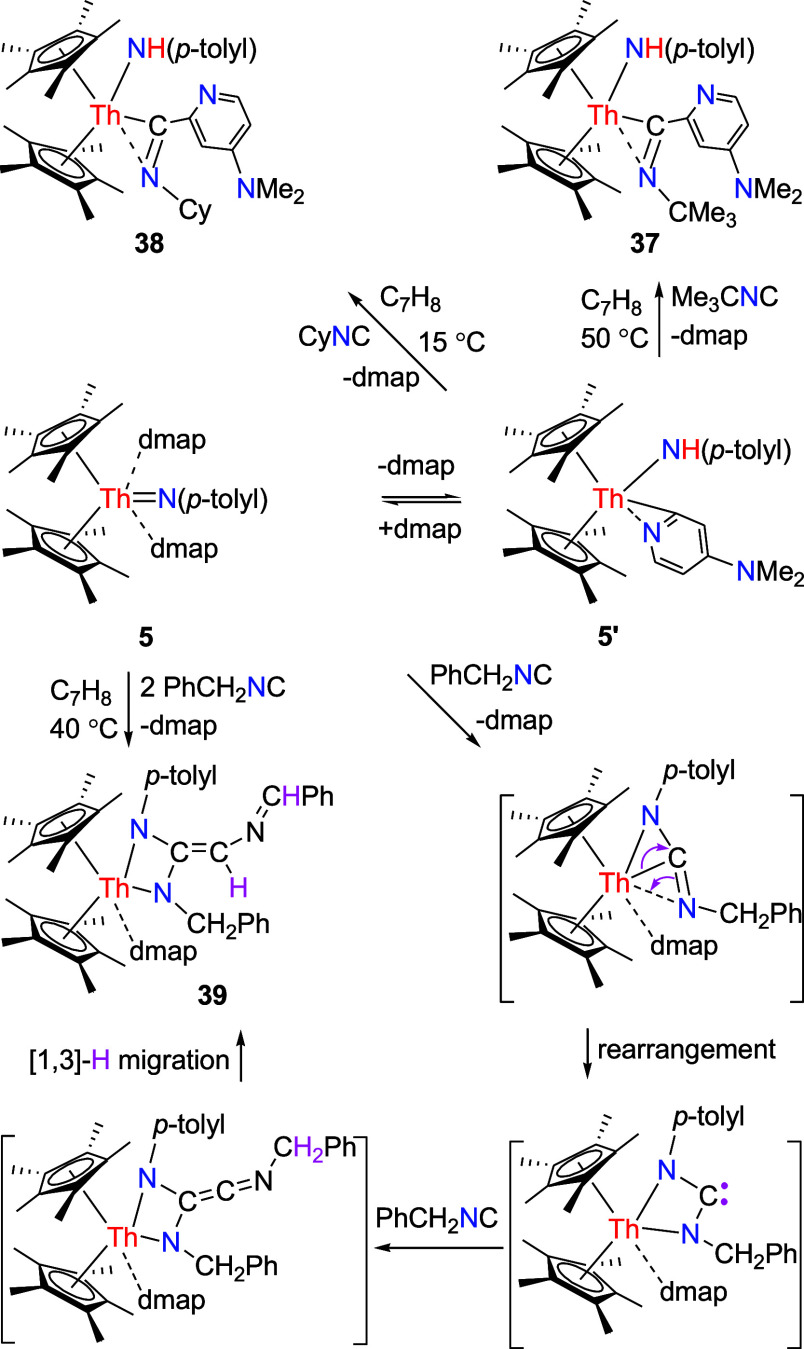
Synthesis of Compounds **37**–**39**

Nevertheless, exposure of complex **5** to 2 equiv of PhCH_2_NC in toluene at 40 °C results in a four-membered heterocyclic complex (η^5^-C_5_Me_5_)_2_Th­[N­(*p*-tolyl)­C­(=CHN=CHPh)­N­(CH_2_Ph)]­(dmap) (**39**) with one dmap being released (Scheme 14). We suggest that **5** initially reacts with PhCH_2_NC via a [2 + 1] cycloaddition with one dmap loss to give a metallaaziridine, which rearranges to a four-membered heterometallacycle (Scheme 14). Within this intermediate, the reactive carbene fragment (RR′C:) subsequently couples with a second molecule of isonitrile PhCH_2_NC to afford the other four-membered heterometallacycle, which then undergoes a [1,3]-H migration to yield the complex **39** (Scheme 14). The molecular structure of **39** is provided in Figure 36, and selected bond distances and angles are detailed in Table 1. The Th–N (1) and Th–N(2) lengths are 2.350(16) and 2.374(17) Å, respectively, while the relatively long Th–N(4) distance of 2.651(8) Å is indicative of a datively coordinated nitrogen atom.

**Figure 36:**
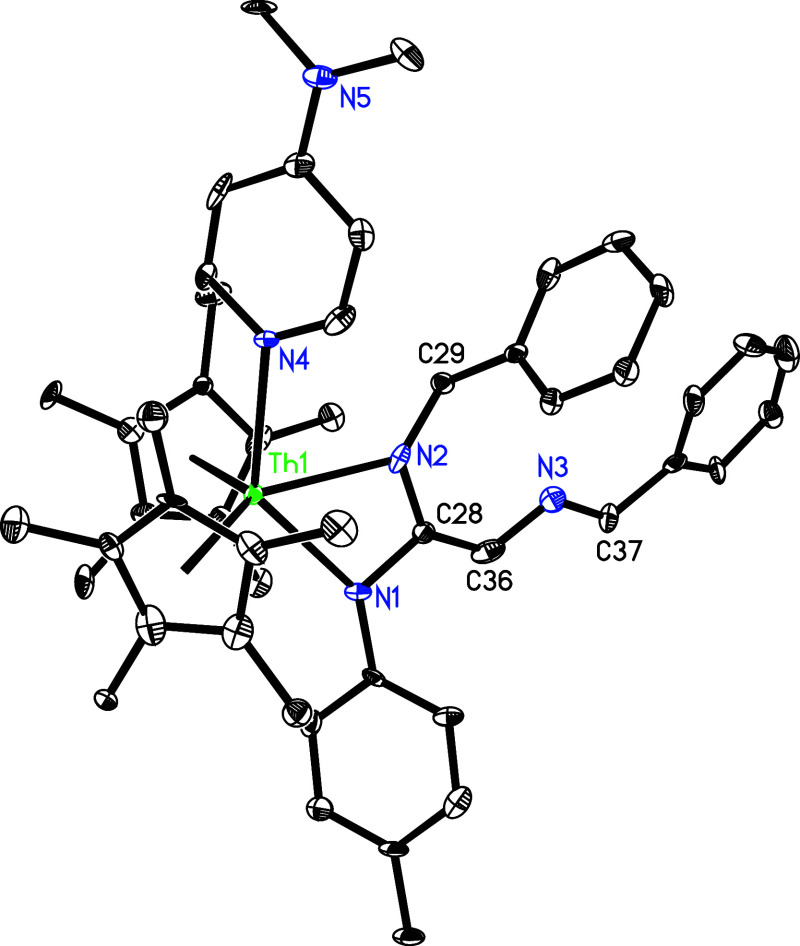
Molecular structure of **39** (thermal ellipsoids drawn at the 35% probability level).

### Reaction with Organic Azides and Diazoalkane Derivatives

Moreover, analogously to the thorium imido complexes [η^5^-1,2,4-(Me_3_C)_3_C_5_H_2_]_2_Th=N­(*p*-tolyl) (Figure S1),^7d,8^ (η^5^-C_5_Me_5_)_2_Th=N­(mesityl)­(dmap) (Figure S2),^7g,8^ and [η^5^-1,3-(Me_3_C)_2_C_5_H_3_]_2_Th=N­(dipp)­(dmap) (Figure S3),^7z,8^ compound **5** also reacts with organic azides. For example, contrary to [η^5^-1,3-(Me_3_C)_2_C_5_H_3_]_2_Th=N­(dipp)­(dmap) affording a bis-amido complex [η^5^-1,3-(Me_3_C)_2_C_5_H_3_]_2_Th­[NH­(*p*-tolyl)]­[2-(dippN_3_)-4-(Me_2_N)­C_5_H_3_N] with *p*-tolylN_3_ (Figure S3),^7z,8^ but like [η^5^-1,2,4-(Me_3_C)_3_C_5_H_2_]_2_Th=N­(*p*-tolyl) and (η^5^-C_5_Me_5_)_2_Th=N­(mesityl)­(dmap) (Figures S1 and S2),^7d,7,8^ exposure of complex **5** toward *p*-tolylN_3_ in toluene at 40 °C eliminates dmap and gives the known tetraazametallacyclopentene (η^5^-C_5_Me_5_)_2_Th­[N­(*p*-tolyl)­N=NN­(*p*-tolyl)] (**40**) (Scheme 15), presumably due to the less steric hindrance introduced by the *p*-tolyl group compared with the 2,6-*^i^* Pr_2_C_6_H_3_ group. However, when this reaction is carried out in toluene at 15 °C, dmap is released and the bis-amido complex (η^5^-C_5_Me_5_)_2_Th­[NH­(*p*-tolyl)]­[κ^2^-*N*,*N*-2-N­(NN-*p*-tolyl)-4-(Me_2_N)­C_5_H_3_N] (**41**) is isolated (Scheme 15), once again, providing support for the equilibrium between **5** and **5′** + dmap in the solution. A similar reactivity is observed when Ph_3_CN_3_ is added to complex **5**, resulting in the bis-amido complex (η^5^-C_5_Me_5_)_2_Th­[NH­(*p*-tolyl)]­[κ^2^-*N*,*N*-2-N­(NNCPh_3_)-4-(Me_2_N)­C_5_H_3_N] (**42**) in quantitative conversion with the loss of one molecule of dmap (Scheme 15). The molecular structure of **41** is shown in Figure 37, while the molecular structure of **42** is provided in the Supporting Information. In complex **41**, the Th–N (1), Th–N(2) and Th–N(4) distances measure 2.344(4), 2.551(4) and 2.496(4) Å, respectively–values that are comparable to those found in **42** (2.304(5), 2.597(4) and 2.494(4) Å, respectively). In contrast, treatment of complex **5** with Me_3_SiN_3_ yields the azido amido complex (η^5^-C_5_Me_5_)_2_Th­(N_3_)­[N­(*p*-tolyl)­SiMe_3_] (**43**) along with dmap in quantitative conversion (Scheme 15), wherein the Th=N­(*p*-tolyl) moiety serves as a nucleophile. This reactivity diverges from that observed with [η^5^-1,3-(Me_3_C)_2_C_5_H_3_]_2_Th=N­(dipp)­(dmap), which forms a bis-azido complex [η^5^-1,3-(Me_3_C)_2_C_5_H_3_]_2_Th­(N_3_)_2_(dmap)_2_ (Figure S3),^7z,8^ presumably due to the increased steric hindrance imparted by the C_5_Me_5_ ligand compared with the 1,3-(Me_3_C)_2_C_5_H_3_ ligand. The molecular structure of **43** is provided in Figure 38, with selected bond distances and angles reported in Table 1. The Th–N (1) distance is 2.318(3) Å, whereas Th–N(4) distance is 2.343(3) Å. Moreover, the N(1)–Th-N(4) angle is 87.6 (1)°.

**Scheme 15:**
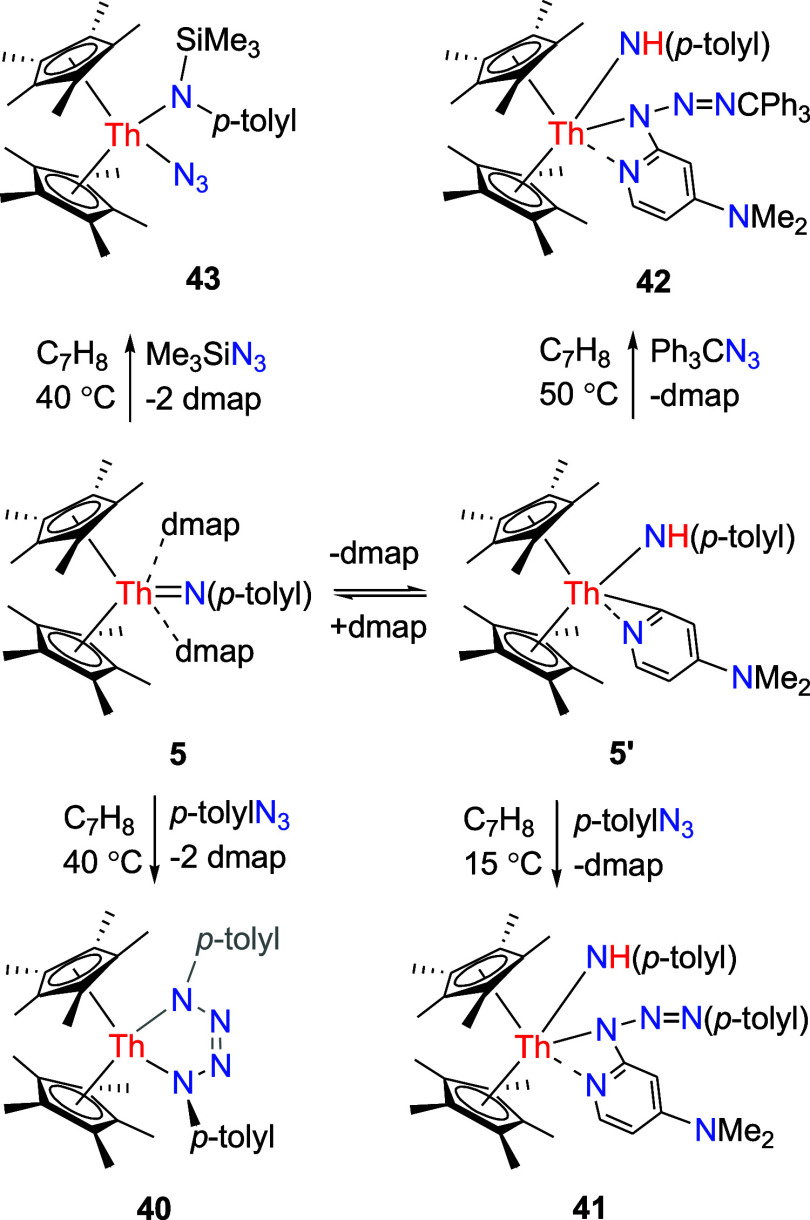
Synthesis of Compounds **40**–**43**

**Figure 37:**
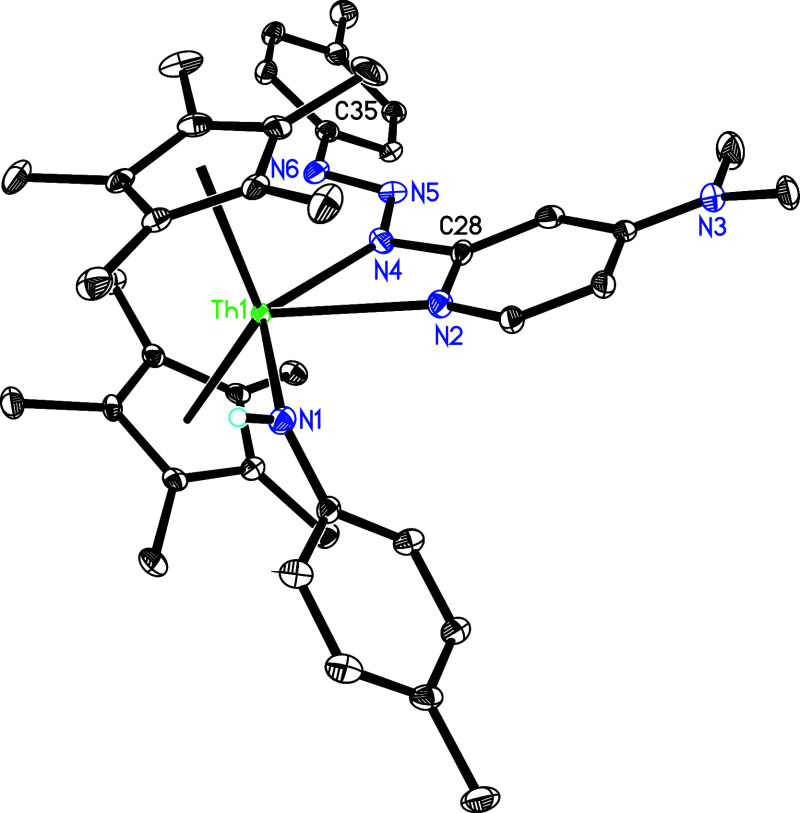
Molecular structure of **41** (thermal ellipsoids drawn at the 35% probability level).

**Figure 38:**
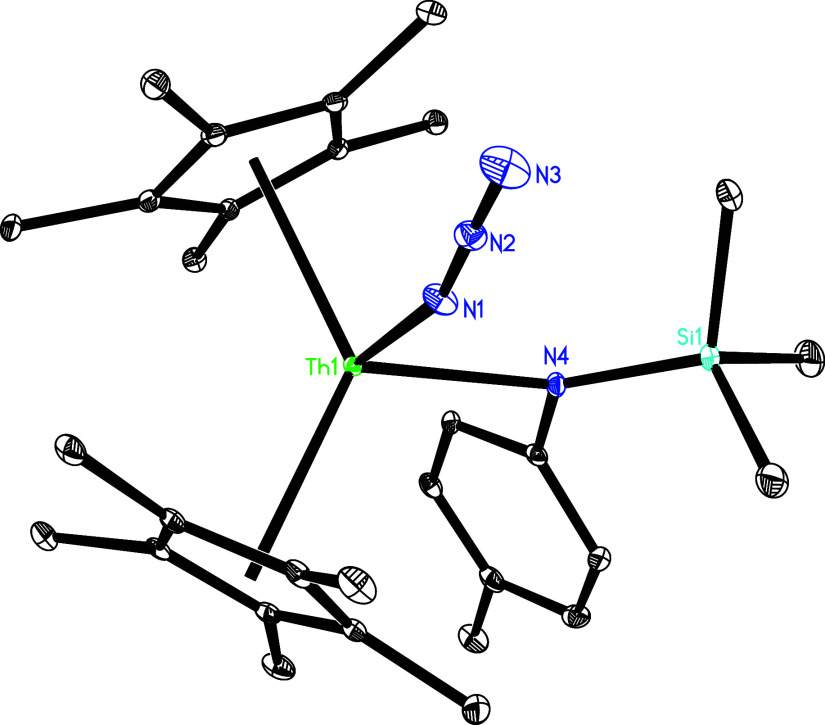
Molecular structure of **43** (thermal ellipsoids drawn at the 35% probability level).

Moreover, analogous to the reactivity observed for the thorium imido complexes [η^5^-1,2,4-(Me_3_C)_3_C_5_H_2_]_2_Th=N­(*p*-tolyl) (Figure S1),^7d,8^ and (η^5^-C_5_Me_5_)_2_Th=N­(mesityl)­(dmap) (Figure S2),^7g,8^ complex **5** also engages with diazoalkanes. However, contrary to [η^5^-1,2,4-(Me_3_C)_3_C_5_H_2_]_2_Th=N­(*p*-tolyl) forming the amido nitrilimido complex [η^5^-1,2,4-(Me_3_C)_3_C_5_H_2_]_2_Th­[NH­(*p*-tolyl)]­(N_2_CSiMe_3_) with Me_3_SiCHN_2_ (Figure S1),^7d,8^ but similar to (η^5^-C_5_Me_5_)_2_Th=N­(mesityl)­(dmap) (Figure S2),^7g,8^ no amido nitrilimido complex is produced. Instead, the known bimetallic compound [(η^5^-C_5_Me_5_)_2_Th]_2_(μ-N=NN=CSiMe_3_)_2_ (**44**) is isolated (Scheme 16), a result that is presumably attributed to the reduced steric bulk of the C_5_Me_5_ ligand compared to the 1,2,4-(Me_3_C)_3_C_5_H_2_ ligand. We propose the following mechanism: Complex **5** initially reacts with Me_3_SiCHN_2_ to generate a three-membered complex when liberating dmap. This intermediate undergoes a [1,3]-C migration to yield *p*-tolyl complex, which immediately experiences either inter- or intramolecular deprotonation of an α-H from the Me_3_SiCHN_3_ group to yield complex **44** and toluene as a byproduct (Scheme 16).

**Scheme 16:**
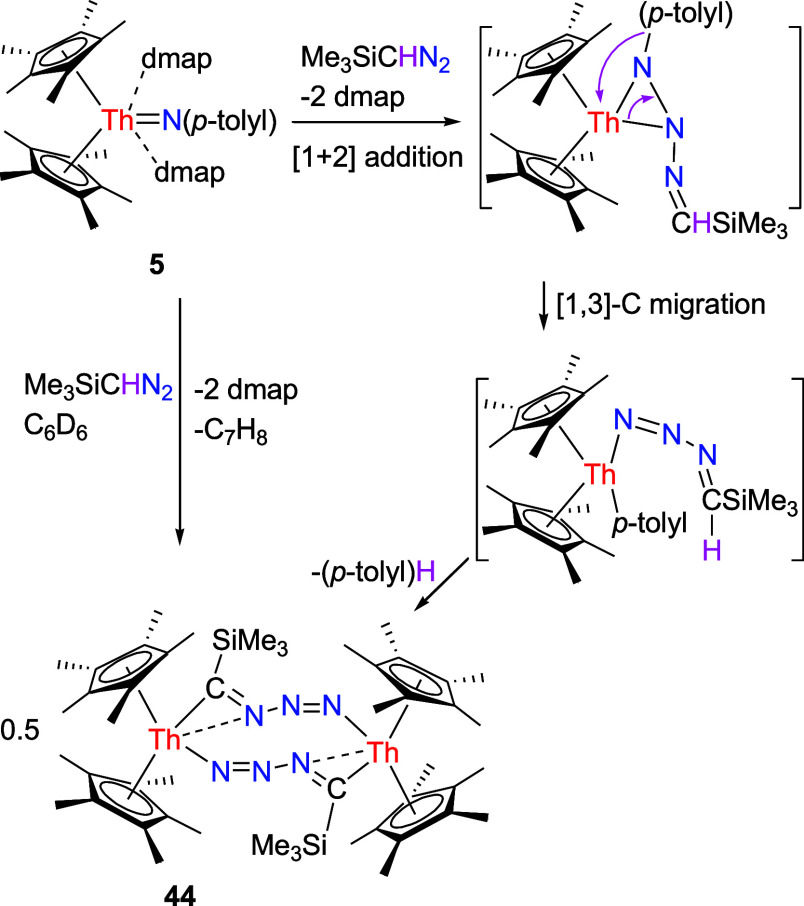
Synthesis of Compound **44**

## Conclusions

In summary, we have synthesized and fully characterized the Lewis base supported thorium imido metallocene, (η^5^-C_5_Me_5_)_2_Th=N­(*p*-tolyl)­(dmap)_2_ (**5**). In toluene solution, an equilibrium is established between **5**, dmap and the amido pyridyl complex (η^5^-C_5_Me_5_)_2_Th­[NH­(*p*-tolyl)]­[κ^2^-*C*,*N*-4-(Me_2_N)­C_5_H_3_N] (**5′**), highlighting the complex’s inherent versatility. Complex **5** engages a broad spectrum of substrates–ranging from 1-methylimidazole, *pyridine-N-oxide derivative* 2,6-Me_2_C_5_H_3_NO, Me_3_PO, elemental sulfur (S_8_) and selenium (Se), metal halides, silanes, and alkynes, to carbodiimides, ketones, thio-ketones, isothiocyanates, CS_2_, esters, amidates, organic nitriles and isonitriles, and organic azides, thus affording a diverse array of products. These include amido imidazolyl complex, amido alkyl complex, and bis-amido complex, as well as heterometallacycles of various ring sizes (four-, five-, six- or eight-membered), dihalido species, bis-amidate compounds, amidinyl iminato complexes, amido phenyl, amido pyridyl, and amido alkenyl derivatives. Moreover, reactions with elemental selenium (Se) and tellurium (Te), chlorosilane PhSiH_2_Cl, organic isonitriles (2,6-Me_2_C_6_H_3_NC, Me_3_CNC and C_6_H_11_NC), and organic azides (*p*-tolylN_3_ and Ph_3_CN_3_) further underscore the remarkable potential of complex **5′** in small molecule activation forming the amido selenido compound (η^5^-C_5_Me_5_)_2_Th­[NH­(*p*-tolyl)]­[κ^2^-*N,Se*-2-Se-4-(Me_2_N)­C_5_H_3_N] (**12**), amido tellurido species (η^5^-C_5_Me_5_)_2_Th­[NH­(*p*-tolyl)]­[κ^2^-*N,Te*-2-Te-4-(Me_2_N)­C_5_H_3_N] (**13**), chloro pyridyl complex (η^5^-C_5_Me_5_)_2_Th­(Cl)­[κ^2^-*C*,*N*-4-(Me_2_N)­C_5_H_3_N] (**17**), amido pyridyl complex (η^5^-C_5_Me_5_)_2_Th­[NH­(*p*-tolyl)]­[κ^2^-*C*,*N*-4-(Me_2_N)-6-(2,6-Me_2_C_6_H_3_N=CH)­C_5_H_2_N] (**36**), amido alkenyl complexes (η^5^-C_5_Me_5_)_2_Th­[NH­(*p*-tolyl)]­[κ^2^-*C*,*N*-2-(Me_3_CNC)-4-(Me_2_N)­C_5_H_3_N] (**37**) and (η^5^-C_5_Me_5_)_2_Th­[NH­(*p*-tolyl)]­[κ^2^-*C*,*N*-2-(C_6_H_11_NC)-4-(Me_2_N)­C_5_H_3_N] (**38**), and bis-amido complexes (η^5^-C_5_Me_5_)_2_Th­[NH­(*p*-tolyl)]­[κ^2^-*N*,*N*-2-N­(NN-*p*-tolyl)-4-(Me_2_N)­C_5_H_3_N] (**41**) and (η^5^-C_5_Me_5_)_2_Th­[NH­(*p*-tolyl)]­[κ^2^-*N*,*N*-2-N­(NNCPh_3_)-4-(Me_2_N)­C_5_H_3_N] (**42**), respectively. Furthermore, while mixing **5** with Me_3_SiN_3_ affords the azido amido complex (η^5^-C_5_Me_5_)_2_Th­(N_3_)­[N­(*p*-tolyl)­SiMe_3_] (**43**), the bimetallic complex [(η^5^-C_5_Me_5_)_2_Th]_2_(μ-N=NN=CSiMe_3_)_2_ (**44**) and toluene are formed when **5** is exposed to Me_3_SiCHN_2_.

Moreover, while these thorium imido metallocenes share similar overall reactivity patterns, subtle variations in the cyclopentadienyl ligand can fine-tune their individual reactivity patterns. For example, imido [η^5^-1,2,4-(Me_3_C)_3_C_5_H_2_]_2_Th=N­(*p*-tolyl) is stable,^7a,7b^ whereas (η^5^-C_5_Me_5_)_2_Th=N­(*p*-tolyl) is not. While the reaction of [η^5^-1,2,4-(Me_3_C)_3_C_5_H_2_]_2_Th=N­(*p*-tolyl) with S_8_ or Se gives four-membered metallaheterocycles [η^5^-1,2,4-(Me_3_C)_3_C_5_H_2_]_2_Th­[N­(*p*-tolyl)­EE] (E = S, Se) (Figure S1),^7b,7,8^ compound **5** yields the six-membered complex (η^5^-C_5_Me_5_)_2_Th­[N­(*p*-tolyl)­E_4_] (E = S (**10**), Se (**11**)). The amido hydrido complex [η^5^-1,2,4-(Me_3_C)_3_C_5_H_2_]_2_Th­(H)­[N­(*p*-tolyl)­SiH_2_Ph] formed from [η^5^-1,2,4-(Me_3_C)_3_C_5_H_2_]_2_Th=N­(*p*-tolyl) and PhSiH_3_ is stable (Figure S1),^7c,8^ whereas a C–H bond activation of the *p*-tolyl ligand occurs for the product derived from **5** and PhSiH_3_. Moreover, while the [2 + 2] cycloaddition product (η^5^-C_5_Me_5_)_2_Th­[N­(*p*-tolyl)­CPh_2_O]­(dmap) (**23**) can be isolated from the reaction of complex **5** with 1 equiv of Ph_2_CO, this is not possible for [η^5^-1,2,4-(Me_3_C)_3_C_5_H_2_]_2_Th=N­(*p*-tolyl).^7a^ Instead, the terminal oxido dmap adduct [η^5^-1,2,4-(Me_3_C)_3_C_5_H_2_]_2_Th=O­(dmap) is generated in the reaction between [η^5^-1,2,4-(Me_3_C)_3_C_5_H_2_]_2_Th=N­(*p*-tolyl) and Ph_2_CO in the presence of dmap (Figure S1).^7a,8^ Furthermore, treatment of [η^5^-1,2,4-(Me_3_C)_3_C_5_H_2_]_2_Th=N­(*p*-tolyl) with PhNCS affords a formal [2 + 2]-cycloaddition product [η^5^-1,2,4-(Me_3_C)_3_C_5_H_2_]_2_Th­[N­(*p*-tolyl)­C­(=NPh)-S] (Figure S1),^7a,8^ whereas (η^5^-C_5_Me_5_)_2_Th=N­(mesityl)­(dmap) (Figure S2)^7g,8^ and compound **5** yield the four-membered metallaheterocycles (η^5^-C_5_Me_5_)_2_Th­[SC=N­(mesityl)­NPh]­(dmap) and (η^5^-C_5_Me_5_)_2_Th­[SC=N­(*p*-tolyl)­NPh]­(dmap) (**26**), respectively. While exposure of [η^5^-1,2,4-(Me_3_C)_3_C_5_H_2_]_2_Th=N­(*p*-tolyl) toward CS_2_ furnishes the formal [2 + 2]-cycloaddition product [η^5^-1,2,4-(Me_3_C)_3_C_5_H_2_]_2_Th­[N­(*p*-tolyl)­C­(=S)-S] (Figure S1),^7a,8^ the imido complexes (η^5^-C_5_Me_5_)_2_Th=N­(mesityl)­(dmap) (Figure S2)^7g,8^ and **5** give either a four-membered metallaheterocycle (η^5^-C_5_Me_5_)_2_Th­[SC=N­(mesityl)-S]­(dmap) or a dimeric complex [(η^5^-C_5_Me_5_)_2_Th]_2_{μ-[N­(*p*-tolyl)­C­(S)­S]}_2_ (**27**). The imido compound [η^5^-1,2,4-(Me_3_C)_3_C_5_H_2_]_2_Th=N­(*p*-tolyl) is an effective catalyst for the trimerization of PhCN,^7b^ whereas **5** reacts with 3 equiv of PhCN to afford an eight-membered complex (η^5^-C_5_Me_5_)_2_Th­[η^7^-N­(*p*-tolyl)­C­(Ph)­NC­(Ph)­NC­(Ph)­N] (**32**). Furthermore, the amido complex [η^5^-1,2,4-(Me_3_C)_3_C_5_H_2_]­[η^5^-1-(2,6-Me_2_C_6_H_3_N=CCH_2_Me_2_C)-3,4-(Me_3_C)_2_C_5_H_2_]­ThNH­(*p*-tolyl) is isolated from the reaction of [η^5^-1,2,4-(Me_3_C)_3_C_5_H_2_]_2_Th=N­(*p*-tolyl) with 2,6-Me_2_C_6_H_3_NC (Figure S1),^7h,8^ but **5** produces an amido pyridyl complex (η^5^-C_5_Me_5_)_2_Th­[N­(*p*-tolyl)=CHN­(2,6-Me_2_C_6_H_3_)]­[κ^2^-*C*,*N*-4-(Me_2_N)­C_5_H_3_N] (**35**). Although the reaction of [η^5^-1,2,4-(Me_3_C)_3_C_5_H_2_]_2_Th=N­(*p*-tolyl) with Me_3_SiCHN_2_ results in the formation of an amido nitrilimido complex [η^5^-1,2,4-(Me_3_C)_3_C_5_H_2_]_2_Th­[NH­(*p*-tolyl)]­(N_2_CSiMe_3_) (Figure S1),^7d,8^ complexes (η^5^-C_5_Me_5_)_2_Th=N­(mesityl)­(dmap) (Figure S2)^7g,8^ and **5** give a bimetallic complex [(η^5^-C_5_Me_5_)_2_Th]_2_(μ-N=NN=CSiMe_3_)_2_ (**44**) concomitant with the elimination of mesitylene and toluene, respectively.

However, the substituents on the imido group also influence the reactivity of these Th imido compounds. For example, (η^5^-C_5_Me_5_)_2_Th=N­(mesityl)­(dmap) forms as a mono-dmap adduct,^7f^ whereas complex **5** crystallizes as an adduct with two coordinated dmap ligands. While in the C_7_D_8_ solution, an equilibrium between imido **5** and amido pyridyl complex **5′** cannot be detected by ^1^H NMR spectroscopy, it is observable for (η^5^-C_5_Me_5_)_2_Th=N­(mesityl)­(dmap).^7f^ Reaction of (η^5^-C_5_Me_5_)_2_Th=N­(mesityl)­(dmap) with CuCl or CuBr produce the heterobimetallic compounds (η^5^-C_5_Me_5_)_2_Th­(X)­[N­(mesityl)­Cu­(dmap)] (X = Cl, Br) (Figure S2),^7f,8^ but **5** yields the dichloride complex (η^5^-C_5_Me_5_)_2_ThCl_2_(dmap)_2_ (**15**) and the dibromide complex (η^5^-C_5_Me_5_)_2_ThBr_2_(dmap) (**16**), respectively. Moreover, while reaction of (η^5^-C_5_Me_5_)_2_Th=N­(mesityl)­(dmap) with PhSiH_3_ forms an amido alkyl dmap adduct (η^5^-C_5_Me_5_)_2_Th­[κ^2^-*N*,*C*-{N­(2-CH_2_–4,6-Me_2_C_6_H_2_)­(SiH_2_Ph)}]­(dmap) (Figure S2),^7g,8^ complex **5** gives an amido phenyl complex (η^5^-C_5_Me_5_)_2_Th­[κ^3^-*C*,*N,N*-(4-Me_2_NC_5_H_3_N)­SiH­(Ph)­N­(4-MeC_6_H_3_)] (**18**). Compounds [η^5^-1,2,4-(Me_3_C)_3_C_5_H_2_]_2_Th=N­(*p*-tolyl) (Figure S1)^7b,8^ and **5** immediately react with PhC≡CPh, but (η^5^-C_5_Me_5_)_2_Th=N­(mesityl)­(dmap) does not.^7g^ In addition, while the [2 + 2] cycloaddition product (η^5^-C_5_Me_5_)_2_Th­[N­(*p*-tolyl)­CPh_2_O]­(dmap) (**23**) is isolated from the reaction of complex **5** with 1 equiv of Ph_2_CO, this is not possible for (η^5^-C_5_Me_5_)_2_Th=N­(mesityl)­(dmap) (Figure S2).^7g,8^ Exposure of (η^5^-C_5_Me_5_)_2_Th=N­(mesityl)­(dmap) toward CS_2_ results in a four-membered metallaheterocycle (η^5^-C_5_Me_5_)_2_Th­[SC=N­(mesityl)-S]­(dmap) (Figure S2)^7g,8^ but complex **5** affords the dimeric complex [(η^5^-C_5_Me_5_)_2_Th]_2_{μ-[N­(*p*-tolyl)­C­(S)­S]}_2_ (**27**). Moreover, while reaction of (η^5^-C_5_Me_5_)_2_Th=N­(mesityl)­(dmap) with *rac*-lactide gives an eight-membered metallaheterocycle (η^5^-C_5_Me_5_)_2_Th­[OCH­(Me)­C­(=O)­OCH­(Me)­C­(=Nmesityl)­O] (Figure S2),^7g,8^ complex **5** yields a dimeric product [(η^5^-C_5_Me_5_)_2_Th]_2_{μ-[OCH­(Me)­C­(=O)­OCH­(Me)­C­(=N-*p*-tolyl)­O]}_2_ (**29**). Reaction of (η^5^-C_5_Me_5_)_2_Th=N­(mesityl)­(dmap) with PhCN affords a dmap imido adduct (η^5^-C_5_Me_5_)_2_Th­[=NC­(Ph)=N­(mesityl)]­(dmap) (Figure S2),^7g,8^ whereas [η^5^-1,2,4-(Me_3_C)_3_C_5_H_2_]_2_Th=N­(*p*-tolyl) forms a [2 + 2] cycloaddition product [η^5^-1,2,4-(Me_3_C)_3_C_5_H_2_]_2_Th­[N­(*p*-tolyl)­C­(Ph)=N] (Figure S1),^7b,8^ and complex **5** gives a mixture of a [2 + 2] cycloaddition product (η^5^-C_5_Me_5_)_2_Th­[N­(*p*-tolyl)­C­(Ph)=N]­(dmap) (**31a**) and amidinyl pyridyl complex (η^5^-C_5_Me_5_)_2_Th­[η^3^-NHC­(Ph)­N­(*p*-tolyl)]­[κ^2^-*C*,*N*-4-(Me_2_N)­C_5_H_3_N] (**31b**). Although the tetraazametallacyclopentenes [η^5^-1,2,4-(Me_3_C)_3_C_5_H_2_]_2_Th­[N­(*p*-tolyl)­N=NN­(*p*-tolyl)] (Figure S1)^7d,8^ and (η^5^-C_5_Me_5_)_2_Th­[N­(*p*-tolyl)­N=NN­(*p*-tolyl)] (**40**) are isolated as stable products from the reactions of [η^5^-1,2,4-(Me_3_C)_3_C_5_H_2_]_2_Th=N­(*p*-tolyl) and complex **5** with *p*-tolylN_3_, respectively, NN cleavage and mesitylN_3_ elimination are encountered in the reaction of (η^5^-C_5_Me_5_)_2_Th=N­(mesityl)­(dmap) with *p*-tolylN_3_ (Figure S2).^7g,8^ Further exploration of actinide imido complexes is ongoing, and the results will be reported in due course.

## Experimental Section

### General Procedures

All reactions and product manipulations were conducted under an atmosphere of dry dinitrogen using standard Schlenk or cannula techniques or in a glovebox, ensuring rigid exclusion of air and moisture. Organic solvents were freshly distilled from sodium benzophenone ketyl immediately before use. (η^5^-C_5_Me_5_)_2_ThMe_2_ (**1**)^7f,9^ was prepared according to the literature method, and all other chemicals were purchased from Aldrich Chemical Co. and Beijing Chemical Co. and used as received unless stated otherwise. Infrared spectra were recorded in KBr pellets on an Avatar 360 Fourier transform spectrometer. ^1^H and ^13^C­{^1^H} NMR spectra were recorded on a Bruker AV 400 (at 400 and 100 MHz, respectively), or a JEOL AV 400 (at 400 and 100 MHz, respectively), or a JEOL 600 (at 600 and 151 MHz, respectively). Chemical shifts are reported in δ units and referenced to the residual protons of the deuterated solvents, which served as internal standards, for proton and carbon chemical shifts. ^31^P­{^1^H} NMR spectra were recorded on a JEOL AV 400 at 162 MHz. ^29^Si­{^1^H}, ^77^Se­{^1^H}, and ^125^Te­{^1^H} NMR spectra were recorded on a JEOL 600 at 119.2, 114, and 189 MHz, respectively. Phosphorus, silicon, selenium, and tellurium chemical shifts were referenced to external 85% H_3_PO_4_ (0.00 ppm), Me_4_Si (0.00 ppm), Me_2_Se (0.00 ppm), and Me_2_Te (0.00 ppm), respectively. Melting points were determined on an X-6 melting point apparatus and were uncorrected. Elemental analyses were performed on a Vario EL elemental analyzer.

*Caution*: Natural thorium (primary isotope ^232^Th) is a weak α-emitter (4.012 MeV) with a half-life of 1.41 × 10^10^ years. Therefore, manipulations and reactions should be carried out in monitored fume hoods or in an inert-atmosphere drybox in a laboratory equipped with α- and β-counting equipment. Moreover, all organic reactants used in this work are standard or commercially available reagents, and there is no special safety consideration.

### Preparation of (η^5^-C_5_Me_5_)_2_Th­(NH-*p*-tolyl)_2_ (**2**)

#### Method A

A toluene (10 mL) solution of *p*-toluidine (0.43 g, 4.0 mmol) was added to a stirred toluene (20 mL) solution of (η^5^-C_5_Me_5_)_2_ThMe_2_ (**1**; 1.06 g, 2.0 mmol) at room temperature. The resulting mixture was stirred overnight at room temperature. Followed by removal of the solvent, the residue was extracted with benzene (3 × 10 mL) and filtered. The volume of the combined filtrates was reduced to 10 mL, and colorless crystals of **2** developed when this solution was kept at 10 °C for 1 day. The crystals of **2** were then collected by filtration, rapidly washed with cold *n*-hexane (5 mL), and dried under vacuum at room temperature overnight. Yield: 1.34 g (94%). M.p.: 188–190 °C (dec.). ^1^H NMR (C_6_D_6_): δ 6.92 (d, *J* = 6.8 Hz, 4H, phenyl), 6.71 (d, *J* = 7.0 Hz, 4H, phenyl), 5.17 (s, 2H, N*H*), 2.20 (s, 6H, tolylC*H*_3_), 1.95 (s, 30H, CpC*H*_3_) ppm. ^13^C­{^1^H} NMR (C_6_D_6_): δ 151.6 (phenyl *C*), 129.9 (phenyl *C*), 126.0 (phenyl *C*), 124.8 (phenyl *C*), 118.0 (ring *C*), 20.7 (tolyl*C*H_3_), 11.3 (Cp*C*H_3_) ppm. IR (KBr, cm^–1^): ν 3315 (m), 2965 (s), 2917 (s), 1605 (s), 1503 (s), 1404 (s), 1383 (s), 1262 (s), 1108 (s), 1021 (s), 812 (s). Anal. Calcd for C_34_H_46_N_2_Th: C, 57.13; H, 6.49; N, 3.92. Found: C, 57.16; H, 6.52; N, 3.90.

#### Method B

##### NMR Scale

A C_6_D_6_ (0.3 mL) solution of *p*-toluidine (4.3 mg, 0.04 mmol) was slowly added to a J. Young NMR tube charged with (η^5^-C_5_Me_5_)_2_ThMe_2_ (**1**; 10.6 mg, 0.02 mmol) and C_6_D_6_ (0.2 mL). Resonances of **2** and that of methane were observed by ^1^H NMR spectroscopy (100% conversion) when this solution was kept at room temperature overnight.

### Preparation of [(η^5^-C_5_Me_5_)_2_Th]_2_[μ-N­(*p*-tolyl)]_2_·C_6_H_6_ (3·C_6_H_6_)

#### Method A

A toluene (10 mL) solution of (η^5^-C_5_Me_5_)_2_Th­(NH-*p*-tolyl)_2_ (**2**; 0.72 g, 1.0 mmol) was added to a stirred toluene (20 mL) solution of (η^5^-C_5_Me_5_)_2_ThMe_2_ (**1**; 0.53 g, 1.0 mmol) at room temperature. The mixture was then stirred at 80 °C overnight and subsequently cooled to room temperature. After removal of the solvent under reduced pressure, the resulting residue was extracted with benzene (3 × 10 mL) and filtered. The combined filtrate was concentrated to 10 mL, and yellow crystals of **3**·C_6_H_6_ formed when this solution was maintained at 10 °C for 1 day. Crystals of **3**·C_6_H_6_ were isolated by filtration, rapidly washed with cold *n*-hexane (5 mL), and dried under vacuum at room temperature overnight. Yield: 1.14 g (88%). M.p.: 178–180 °C (dec.). ^1^H NMR (C_6_D_6_): δ 7.19 (d, *J* = 7.4 Hz, 4H, phenyl), 7.15 (s, 6H, C_6_ *H*_6_), 6.54 (d, *J* = 7.4 Hz, 4H, phenyl), 2.36 (s, 6H, tolylC*H*_3_), 2.10 (s, 60H, CpC*H*_3_) ppm. ^13^C­{^1^H} NMR (C_6_D_6_): δ 150.0 (phenyl *C*), 131.9 (phenyl *C*), 128.5 (*C*_6_H_6_), 127.2 (phenyl *C*), 125.9 (phenyl *C*), 119.4 (ring *C*), 20.7 (tolyl*C*H_3_), 13.3 (Cp*C*H_3_) ppm. IR (KBr, cm^–1^): ν 2961 (s), 2906 (s), 2856 (s), 1598 (m), 1435 (s), 1384 (s), 1259 (s), 1089 (s), 1019 (s), 804 (s). Anal. Calcd for C_60_H_80_N_2_Th_2_: C, 55.72; H, 6.23; N, 2.17. Found: C, 55.74; H, 6.21; N, 2.15.

#### Method B

##### NMR Scale

A C_6_D_6_ (0.3 mL) solutionof *p*-toluidine (2.1 mg, 0.02 mmol) was slowly addedto a J. Young NMR tube charged with (η^5^-C_5_Me_5_)_2_ThMe_2_ (**1**; 10.6mg, 0.02 mmol) and C_6_D_6_ (0.2 mL). Resonancesof **3** and that of methane were observed by ^1^H NMR spectroscopy (100% conversion) when this solution was keptat 80 °C overnight.

### Preparation of (η^5^-C_5_Me_5_)_2_Th­[NH­(*p*-tolyl)]­(κ^2^-*C*,*N*-C_5_H_4_N) (**4**)

#### Method A

A toluene (10 mL) solution of *p*-toluidine (0.21 g, 2.0 mmol) and pyridine (0.16 g, 2.0 mmol) was added to a stirred toluene (20 mL) solution of (η^5^-C_5_Me_5_)_2_ThMe_2_ (**1**; 1.06 g, 2.0 mmol) at room temperature. The reaction mixture was then stirred at 80 °C overnight. After cooling, the solvent was removed under reduced pressure, and the resulting residue was extracted with benzene (3 × 10 mL) and filtered. The volume of the combined filtrate was reduced to 10 mL, and colorless crystals of **4** formed when this solution was kept at 10 °C for 1 day. These crystals were isolated by filtration, rapidly washed with cold *n*-hexane (5 mL), and subsequently dried under vacuum at room temperature overnight. Yield: 1.24 g (90%). M.p.: 130–132 °C (dec.). ^1^H NMR (C_6_D_6_): δ 8.27 (d, *J* = 5.2 Hz, 1H, py), 7.78 (d, *J* = 7.3 Hz, 1H, py), 7.13 (m, 3H, py and phenyl), 6.81 (d, *J* = 8.4 Hz, 2H, phenyl), 6.59 (m, 1H, py), 4.62 (s, 1H, N*H*), 2.38 (s, 3H, tolylC*H*_3_), 1.89 (s, 30H, CpC*H*_3_) ppm. ^13^C­{^1^H} NMR (C_6_D_6_): δ 238.2 (Th*C*), 155.2 (aryl *C*), 145.0 (aryl *C*), 136.1 (aryl *C*), 131.0 (aryl *C*), 129.6 (aryl *C*), 124.0 (aryl *C*), 123.3 (aryl *C*), 122.8 (aryl *C*), 118.0 (ring *C*), 20.8 (tolyl*C*H_3_), 11.2 (Cp*C*H_3_) ppm. IR (KBr, cm^–1^): ν 2921 (s), 2856 (s), 1585 (s), 1440 (s), 1384 (s), 1260 (s), 1092 (s), 1020 (s), 800 (s). Anal. Calcd for C_32_H_42_N_2_Th: C, 55.97; H, 6.16; N, 4.08. Found: C, 55.96; H, 6.14; N, 4.11.

#### Method B

##### NMR Scale

A C_6_D_6_ (0.3 mL) solution of *p*-toluidine (2.1 mg, 0.02 mmol) and pyridine (1.6 mg, 0.02 mmol) was slowly added to a J. Young NMR tube charged with (η^5^-C_5_Me_5_)_2_ThMe_2_ (**1**; 10.6 mg, 0.02 mmol) and C_6_D_6_ (0.2 mL). Resonances of **4** and that of methane were observed by ^1^H NMR spectroscopy (100% conversion) when this solution was kept at 80 °C overnight.

### Preparation of (η^5^-C_5_Me_5_)_2_Th=N­(*p*-tolyl)­(dmap)_2_ (**5**)

A toluene (10 mL) solution of *p*-toluidine (0.21 g, 2.0 mmol) and dmap (0.50 g, 4.1 mmol) was added to a stirred toluene (20 mL) solution of (η^5^-C_5_Me_5_)_2_ThMe_2_ (**1**; 1.06 g, 2.0 mmol) at room temperature. The reaction mixture was stirred overnight at ambient temperature. After removal of the solvent under reduced pressure, the residue was extracted with benzene (3 × 10 mL) and filtered. The filtrate was concentrated to 10 mL, and yellow crystals of complex **5** formed upon storage at 10 °C for 1 day. The crystals were then isolated by filtration, rapidly washed with 5 mL of cold *n*-hexane, and dried under vacuum at room temperature overnight. Yield: 1.47 g (86%). M.p.: 138–140 °C (dec.). ^1^H NMR (C_6_D_6_): δ 8.46 (d, *J* = 3.8 Hz, 2H, dmap), 8.15 (d, *J* = 6.0 Hz, 1H, py), 7.17 (m, 3H, phenyl and py), 6.91 (d, *J* = 8.0 Hz, 2H, phenyl), 6.09 (d, *J* = 5.4 Hz, 2H, dmap), 6.05 (m, 1H, py), 4.62 (s, 1H, N*H*), 2.40 (s, 3H, tolylC*H*_3_), 2.34 (s, 6H, N­(C*H*_3_)_2_), 2.21 (s, 6H, (C*H*_3_)_2_N, dmap), 2.01 (s, 30H, CpC*H*_3_) ppm. ^13^C­{^1^H} NMR (C_6_D_6_): δ 234.2 (Th*C*), 155.9 (aryl *C*), 154.8 (aryl *C*), 154.3 (dmap *C*), 150.4 (dmap *C*), 143.9 (aryl *C*), 129.6 (aryl *C*), 122.8 (aryl *C*), 122.4 (ring *C*), 118.2 (aryl *C*), 110.9 (aryl *C*), 108.7 (aryl *C*), 106.7 (dmap *C*), 38.6 (N*C*H_3_, dmap), 38.2 (N*C*H_3_), 20.9 (tolyl*C*H_3_), 11.3 (Cp*C*H_3_) ppm. IR (KBr, cm^–1^): ν 2907 (s), 1608 (s), 1581 (s), 1502 (s), 1481 (s), 1383 (s), 1267 (s), 1227 (s), 1001 (s), 805 (s). Anal. Calcd for C_41_H_57_N_5_Th: C, 57.80; H, 6.74; N, 8.22. Found: C, 57.78; H, 6.76; N, 8.21. NMR spectroscopy only showed the presence of the resonances of (η^5^-C_5_Me_5_)_2_Th­[NH­(*p*-tolyl)]­[κ^2^-*C*,*N*-4-(Me_2_N)­C_5_H_3_N] (**5′**) and dmap in C_6_D_6_ solution, indicating that an equilibrium between **5** and **5′** + dmap may exist in the solution.

### Preparation of (η^5^-C_5_Me_5_)_2_Th­[NH­(*p*-tolyl)]­(κ^2^-*C*,*N*-1-MeC_3_H_2_N_2_) (**6**)

#### Method A

A toluene (5 mL) solution of 1-methylimidazole (21 mg, 0.25 mmol) was added to a toluene (10 mL) solution of (η^5^-C_5_Me_5_)_2_Th=N­(*p*-tolyl)­(dmap)_2_ (**5**; 213 mg, 0.25 mmol) with stirring at room temperature. After this solution was stirred at room temperature overnight, the solvent was removed under reduced pressure. The residue was extracted with benzene (3 × 10 mL) and then filtered. The volume of the combined filtrate was reduced to 5 mL, and colorless crystals of complex **6** formed upon storage at 10 °C for 2 days. These crystals were isolated by filtration, rapidly washed with cooled *n*-hexane (2 mL), and dried under vacuum at room temperature overnight. Yield: 159 mg (92%). M.p.: 60–62 °C (dec.). ^1^H NMR (C_6_D_6_): δ 7.13 (d, *J* = 8.0 Hz, 2H, aryl), 6.95 (m, 3H, aryl), 6.89 (s, 1H, aryl), 4.55 (s, 1H, N*H*), 3.21 (s, 3H, NC*H*_3_), 2.37 (s, 3H, tolylC*H*_3_), 1.97 (s, 30H, CpC*H*_3_) ppm. ^13^C­{^1^H} NMR (C_6_D_6_): δ 216.8 (Th*C*), 155.2 (aryl *C*), 129.8 (aryl *C*), 129.6 (aryl *C*), 123.6 (aryl *C*), 123.5 (aryl *C*), 123.2 (aryl *C*), 118.4 (ring *C*), 35.7 (N*C*H_3_), 20.9 (tolyl*C*H_3_), 11.4 (Cp*C*H_3_) ppm. IR (KBr, cm^–1^): ν 2964 (s), 2911 (s), 1606 (s), 1502 (s), 1397 (s), 1264 (s), 1100 (s), 1021 (s), 805 (s). Anal. Calcd for C_31_H_43_N_3_Th: C, 53.98; H, 6.28; N, 6.09. Found: C, 53.96; H, 6.26; N, 6.12.

#### Method B

##### NMR Scale

A C_6_D_6_ (0.3 mL) solution of 1-methylimidazole (1.7 mg, 0.02 mmol) was slowly added to a J. Young NMR tube charged with (η^5^-C_5_Me_5_)_2_Th=N­(*p*-tolyl)­(dmap)_2_ (**5**; 17.0 mg, 0.02 mmol) and C_6_D_6_ (0.2 mL). Resonances of **6** and those of dmap were observed by ^1^H NMR spectroscopy (100% conversion) when this solution was kept at room temperature overnight.

### Preparation of (η^5^-C_5_Me_5_)_2_Th­[NH­(*p*-tolyl)]­(κ^2^-*C*,*O*-2-CH_2_–6-MeC_5_H_3_NO) (**7**)

#### Method A

This compound was prepared as colorless crystals by reacting (η^5^-C_5_Me_5_)_2_Th=N­(*p*-tolyl)­(dmap)_2_ (**5**; 213 mg, 0.25 mmol) with 2,6-Me_2_C_5_H_3_NO (31 mg, 0.25 mmol) in toluene (15 mL) at room temperature. Recrystallization from a benzene solution following a procedure analogous to that used in the synthesis of complex **6**. The product was isolated by filtration, rapidly washed with cooled *n*-hexane (2 mL), and dried at room temperature under vacuum overnight. Yield: 157 mg (86%). M.p.: 116–118 °C (dec.). ^1^H NMR (C_6_D_6_): δ 7.69 (m, 1H, py), 6.99 (d, *J* = 8.0 Hz, 2H, phenyl), 6.59 (d, *J* = 2.2 Hz, 1H, py), 6.49 (d, *J* = 8.0 Hz, 2H, phenyl), 5.37 (dd, *J* = 6.6 and 2.6 Hz, 1H, py), 4.96 (s, 1H, N*H*), 2.28 (s, 2H, C*H*_2_), 2.15 (s, 30H, CpC*H*_3_), 2.08 (s, 3H, C*H*_3_), 1.96 (s, 3H, C*H*_3_) ppm. ^13^C­{^1^H} NMR (C_6_D_6_): δ 161.7 (aryl *C*), 154.5 (aryl *C*), 148.2 (aryl *C*), 129.6 (aryl *C*), 124.2 (aryl *C*), 124.0 (aryl *C*), 119.5 (aryl *C*), 118.1 (ring *C*), 113.4 (aryl *C*), 102.4 (aryl *C*), 38.0 (Th*C*H_2_), 27.2 (py*C*H_3_), 20.8 (tolyl*C*H_3_), 12.1 (Cp*C*H_3_) ppm. IR (KBr, cm^–1^): ν 2902 (s), 2856 (s), 1594 (s), 1503 (s), 1433 (s), 1374 (s), 1264 (s), 1137 (s), 1075 (s), 1001 (s), 803 (s). Anal. Calcd for C_34_H_46_N_2_OTh: C, 55.88; H, 6.34; N, 3.83. Found: C, 55.86; H, 7.36; N, 3.81.

#### Method B

##### NMR Scale

A C_6_D_6_ (0.3 mL) solution of 2,6-Me_2_C_5_H_3_NO (2.5 mg, 0.02 mmol) was slowly added to a J. Young NMR tube charged with (η^5^-C_5_Me_5_)_2_Th=N­(*p*-tolyl)­(dmap)_2_ (**5**; 17.0 mg, 0.02 mmol) and C_6_D_6_ (0.2 mL). Resonances of **7** and those of dmap were observed by ^1^H NMR spectroscopy (100% conversion) when this solution was kept at room temperature overnight.

### Preparation of (η^5^-C_5_Me_5_)_2_Th­[NH­(*p*-tolyl)]­[κ^2^-*C*,*N*-N­(*p*-tolyl)­P­(Me_2_)­CH_2_] (**8**)

#### Method A

This compound was prepared as colorless crystals from the reaction of (η^5^-C_5_Me_5_)_2_Th=N­(*p*-tolyl)­(dmap)_2_ (**5**; 213 mg, 0.25 mmol) and Me_3_PO (23 mg, 0.25 mmol) in toluene (15 mL) at room temperature. Recrystallization from a benzene solution following a procedure analogous to that used in the synthesis of complex **6**. The product was isolated by filtration, rapidly washed with cooled *n*-hexane (2 mL), and dried at room temperature under vacuum overnight. Yield: 75 mg (38%; based on Th). M.p.: 116–118 °C (dec.). ^1^H NMR (C_6_D_6_): δ 6.97 (d, *J* = 7.6 Hz, 2H, phenyl), 6.93 (d, *J* = 8.0 Hz, 2H, phenyl), 6.81 (d, *J* = 7.6 Hz, 2H, phenyl), 6.16 (d, *J* = 8.0 Hz, 2H, phenyl), 4.27 (s, 1H, N*H*), 2.19 (s, 3H, tolylC*H*_3_), 2.15 (s, 3H, tolylC*H*_3_), 2.14 (s, 30H, CpC*H*_3_), 1.03 (d, *J*_P–H_ = 11.2 Hz, 6H, P­(C*H*_3_)_2_), 0.33 (d, *J*_P–H_ = 9.5 Hz, 2H, C*H*_2_) ppm. ^13^C­{^1^H} NMR (C_6_D_6_): δ 146.0 (P*C*H_2_), 132.9 (phenyl *C*), 129.9 (phenyl *C*), 129.6 (phenyl *C*), 128.8 (phenyl *C*), 128.5 (phenyl *C*), 127.7 (phenyl *C*), 125.1 (ring *C*), 122.9 (phenyl *C*), 119.1 (phenyl *C*), 20.7 (d, *J*_P–C_ = 46.2 Hz, P­(*C*H_3_)_2_), 19.1 (tolyl*C*H_3_), 18.7 (tolyl*C*H_3_), 12.5 (Cp*C*H_3_) ppm. ^31^P­{^1^H} NMR (C_6_D_6_): δ 25.4 ppm. IR (KBr, cm^–1^): ν 2924 (s), 1603 (s), 1380 (s), 1223 (s), 1105 (s), 991 (s), 947 (s), 806 (s). Anal. Calcd for C_37_H_53_N_2_PTh: C, 56.34; H, 6.77; N, 3.55. Found: C, 56.36; H, 6.76; N, 3.57. After isolation of the colorless crystals of **8**, the solvent of the mother liquid was removed. ^1^H NMR spectroscopy showed the presence of the resonances of **8**, dmap, and other unidentified compounds in the residue.

#### Method B

##### NMR Scale

A C_6_D_6_ (0.3 mL) solution of Me_3_PN­(*p*-tolyl) (3.6 mg, 0.02 mmol) was slowly added to a J. Young NMR tube charged with (η^5^-C_5_Me_5_)_2_Th=N­(*p*-tolyl)­(dmap)_2_ (**5**; 17.0 mg, 0.02 mmol) and C_6_D_6_ (0.2 mL). Resonances of **8** and those of dmap were observed by ^1^H NMR spectroscopy (100% conversion) when this solution was kept at room temperature overnight.

### Preparation of (η^5^-C_5_Me_5_)_2_Th­[N­(*p*-tolyl)­SS]­(dmap) (**9**)

#### Method A

Solid S_8_ (16 mg, 0.0625 mmol) was added to a toluene (15 mL) solution of (η^5^-C_5_Me_5_)_2_Th=N­(*p*-tolyl)­(dmap)_2_ (**5**; 213 mg, 0.25 mmol) with stirring at room temperature. After this solution was stirred at 40 °C for 2 days, the solvent was removed. The residue was extracted with benzene (3 × 10 mL) and filtered. The volume of the filtrate was reduced to 5 mL, and colorless microcrystals of **9** formed when this solution was kept at 10 °C for 2 days. Microcrystals of **9** were isolated by filtration, rapidly washed with cooled *n*-hexane (2 mL), and dried at room temperature under vacuum overnight. Yield: 163 mg (82%). M.p.: 120–122 °C (dec.). ^1^H NMR (C_6_D_6_): δ 8.40 (s, 2H, py), 7.68 (d, *J* = 8.2 Hz, 2H, phenyl), 7.03 (d, *J* = 8.2 Hz, 2H, phenyl), 6.03 (s, 2H, py), 2.16 (s, 6H, N­(C*H*_3_)_2_), 2.09 (s, 30H, CpC*H*_3_), 1.99 (s, 3H, tolylC*H*_3_) ppm. ^13^C­{^1^H} NMR (C_6_D_6_): δ 154.5 (py *C*), 150.3 (py *C*), 129.9 (phenyl *C*), 125.9 (phenyl *C*), 124.0 (phenyl *C*), 123.8 (phenyl *C*), 118.2 (ring *C*), 106.9 (py *C*), 38.1 (N*C*H_3_), 20.8 (tolyl*C*H_3_), 11.9 (Cp*C*H_3_) ppm. IR (KBr, cm^–1^): ν 2903 (s), 2854 (s), 1597 (s), 1436 (s), 1377 (s), 1088 (s), 1018 (s), 801(s). Anal. Calcd for C_34_H_47_N_3_S_2_Th: C, 51.44; H, 5.97; N, 5.29. Found: C, 51.46; H, 5.96; N, 6.32. Colorless crystals of **9**·0.5C_6_H_6_·0.5C_7_H_8_ suitable for X-ray structural analysis were isolated from a mixture of benzene and toluene (4:1) solution.

#### Method B

##### NMR Scale

S_8_ (1.3 mg, 0.0051 mmol) was slowly added to a J. Young NMR tube charged with (η^5^-C_5_Me_5_)_2_Th=N­(*p*-tolyl)­(dmap)_2_ (**5**; 17.0 mg, 0.02 mmol) and C_6_D_6_ (0.5 mL). Resonances of **9** and those of dmap were observed by ^1^H NMR spectroscopy (100% conversion) when this solution was kept at 40 °C for 2 days.

### Preparation of (η^5^-C_5_Me_5_)_2_Th­[N­(*p*-tolyl)­S_4_] (**10**)

#### Method A

This compound was prepared as yellow crystals from the reaction of (η^5^-C_5_Me_5_)_2_Th=N­(*p*-tolyl)­(dmap)_2_ (**5**; 213 mg, 0.25 mmol) and S_8_ (32 mg, 0.125 mmol) in toluene (15 mL) at 40 °C and recrystallization from a benzene solution by a similar procedure as that in the synthesis of **9**. The product was isolated by filtration, rapidly washed with cooled *n*-hexane (2 mL), and dried at room temperature under vacuum overnight. Yield: 147 mg (80%). M.p.: 160–162 °C (dec.). ^1^H NMR (C_6_D_6_): δ 7.26 (d, *J* = 8.2 Hz, 2H, phenyl), 7.04 (d, *J* = 8.2 Hz, 2H, phenyl), 2.19 (s, 3H, tolylC*H*_3_), 2.12 (s, 15H, CpC*H*_3_), 1.94 (s, 15H, CpC*H*_3_) ppm. ^13^C­{^1^H} NMR (C_6_D_6_): δ 152.2 (phenyl *C*), 132.9 (phenyl *C*), 129.4 (phenyl *C*), 126.2 (ring *C*), 125.8 (ring *C*), 122.7 (phenyl *C*), 20.9 (tolyl*C*H_3_), 12.1 (Cp*C*H_3_), 12.0 (Cp*C*H_3_) ppm. IR (KBr, cm^–1^): ν 2962 (s), 2908 (s), 2856 (s), 1614 (s), 1535 (s), 1508 (s), 1442 (s), 1381 (s), 1228 (s), 1004 (s), 804 (s). Anal. Calcd for C_27_H_37_NS_4_Th: C, 44.07; H, 5.07; N, 1.90. Found: C, 44.06; H, 5.09; N, 1.87.

#### Method B

##### NMR Scale

S_8_ (2.6 mg, 0.01 mmol) was slowly added to a J. Young NMR tube charged with (η^5^-C_5_Me_5_)_2_Th=N­(*p*-tolyl)­(dmap)_2_ (**5**; 17.0 mg, 0.02 mmol) and C_6_D_6_ (0.5 mL). Resonances of **10** and those of dmap were observed by ^1^H NMR spectroscopy (100% conversion) when this solution was kept at 40 °C for 2 days.

#### Method C

##### NMR Scale

S_8_ (1.3 mg, 0.0051 mmol) was slowly added to a J. Young NMR tube charged with (η^5^-C_5_Me_5_)_2_Th­[N­(*p*-tolyl)­SS]­(dmap) (**9**; 17.6 mg, 0.02 mmol) and C_6_D_6_ (0.5 mL). Resonances of **10** and those of dmap were observed by ^1^H NMR spectroscopy (100% conversion) when this solution was kept at 40 °C for 2 days.

### Preparation of (η^5^-C_5_Me_5_)_2_Th­[N­(*p*-tolyl)­Se_4_] (**11**)

#### Method A

This compound was prepared as orange crystals from the reaction of (η^5^-C_5_Me_5_)_2_Th=N­(*p*-tolyl)­(dmap)_2_ (**5**; 213 mg, 0.25 mmol) and Se (79 mg, 1.00 mmol) in toluene (15 mL) at 40 °C and recrystallization from a benzene solution by a similar procedure as that in the synthesis of **9**. The product was isolated by filtration, rapidly washed with cooled *n*-hexane (2 mL), and dried at room temperature under vacuum overnight. Yield: 194 mg (84%). M.p.: 250–252 °C (dec.). ^1^H NMR (C_6_D_6_): δ 7.22 (d, *J* = 8.2 Hz, 2H, phenyl), 7.02 (d, *J* = 8.2 Hz, 2H, phenyl), 2.18 (s, 3H, tolylC*H*_3_), 2.14 (s, 15H, CpC*H*_3_), 1.94 (s, 15H, CpC*H*_3_) ppm. ^13^C­{^1^H} NMR (C_6_D_6_): δ 156.6 (phenyl *C*), 133.0 (phenyl *C*), 129.1 (phenyl *C*), 126.4 (ring *C*), 125.7 (ring *C*), 123.4 (phenyl *C*), 20.9 (tolyl*C*H_3_), 12.7 (Cp*C*H_3_), 12.3 (Cp*C*H_3_) ppm. ^77^Se­{^1^H} NMR (C_6_D_6_): δ 603.2, 417.3, 385.1, 265.8 ppm. IR (KBr, cm^–1^): ν 2962 (s), 2908 (s), 2854 (s), 1604 (s), 1519 (s), 1504 (s), 1442 (s), 1381 (s), 1226 (s), 1003 (s), 802 (s). Anal. Calcd for C_27_H_37_NSe_4_Th: C, 35.12; H, 4.04; N, 1.52. Found: C, 35.15; H, 4.02; N, 1.54.

#### Method B

##### NMR Scale

Se (6.3 mg, 0.08 mmol) was slowly added to a J. Young NMR tube charged with (η^5^-C_5_Me_5_)_2_Th=N­(*p*-tolyl)­(dmap)_2_ (**5**; 17.0 mg, 0.02 mmol) and C_6_D_6_ (0.5 mL). Resonances of **11** and those of dmap were observed by ^1^H NMR spectroscopy (100% conversion) when this solution was kept at 40 °C for 2 days.

### Preparation of (η^5^-C_5_Me_5_)_2_Th­[NH­(*p*-tolyl)]­[κ^2^-*N,Se*-2-Se-4-(Me_2_N)­C_5_H_3_N] (**12**)

#### Method A

This compound was prepared as colorless crystals from the reaction of (η^5^-C_5_Me_5_)_2_Th=N­(*p*-tolyl)­(dmap)_2_ (**5**; 213 mg, 0.25 mmol) and Se (20 mg, 0.25 mmol) in toluene (15 mL) at 15 °C and recrystallization from a benzene solution by a similar procedure as that in the synthesis of **9**. The product was isolated by filtration, rapidly washed with cooled *n*-hexane (2 mL), and dried at room temperature under vacuum overnight. Yield: 162 mg (80%). M.p.: 218–220 °C (dec.). ^1^H NMR (C_6_D_6_): δ 7.70 (d, *J* = 6.8 Hz, 1H, py), 6.99 (d, *J* = 8.0 Hz, 2H, phenyl), 6.60 (d, *J* = 2.5 Hz, 1H, py), 6.49 (d, *J* = 8.0 Hz, 2H, phenyl), 5.37 (dd, *J* = 6.7 and 2.5 Hz, 1H, py), 4.96 (s, 1H, N*H*), 2.28 (s, 3H, tolylC*H*_3_), 2.15 (s, 30H, CpC*H*_3_), 1.96 (s, 6H, N­(C*H*_3_)_2_) ppm. ^13^C­{^1^H} NMR (C_6_D_6_): δ 161.7 (py *C*), 154.5 (py *C*), 148.2 (py *C*), 129.6 (phenyl *C*), 129.3 (phenyl *C*), 129.1 (phenyl *C*), 124.2 (phenyl *C*), 118.1 (ring *C*), 113.4 (py *C*), 102.3 (py *C*), 38.0 (N*C*H_3_), 20.8 (tolyl*C*H_3_), 12.1 (Cp*C*H_3_) ppm. ^77^Se­{^1^H} NMR (C_6_D_6_): δ 461.1 ppm. IR (KBr, cm^–1^): ν 2902 (s), 1594 (s), 1432 (s), 1384 (s), 1263 (s), 1075 (s), 1001 (s), 800 (s). Anal. Calcd for C_34_H_47_N_3_SeTh: C, 50.49; H, 5.86; N, 5.20. Found: C, 50.47; H, 5.88; N, 5.22.

#### Method B

##### NMR Scale

Se (1.6 mg, 0.02 mmol) was slowly added to a J. Young NMR tube charged with (η^5^-C_5_Me_5_)_2_Th=N­(*p*-tolyl)­(dmap)_2_ (**5**; 17.0 mg, 0.02 mmol) and C_6_D_6_ (0.5 mL). Resonances of **12** and those of dmap were observed by ^1^H NMR spectroscopy (100% conversion) when this solution was kept at 15 °C for 2 weeks.

### Preparation of (η^5^-C_5_Me_5_)_2_Th­[NH­(*p*-tolyl)]­[κ^2^-*N,Te*-2-Te-4-(Me_2_N)­C_5_H_3_N] (**13**)

#### Method A

This compound was prepared as colorless crystals from the reaction of (η^5^-C_5_Me_5_)_2_Th=N­(*p*-tolyl)­(dmap)_2_ (**5**; 213 mg, 0.25 mmol) and Te (32 mg, 0.25 mmol) in toluene (15 mL) at reflux and recrystallization from a benzene solution by a similar procedure as that in the synthesis of **9**. The product was isolated by filtration, rapidly washed with cooled *n*-hexane (2 mL), and dried at room temperature under vacuum overnight. Yield: 180 mg (84%). M.p.: 180–182 °C (dec.). ^1^H NMR (C_6_D_6_): δ 7.85 (d, *J* = 6.7 Hz, 1H, py), 6.98 (d, *J* = 8.0 Hz, 2H, phenyl), 6.85 (d, *J* = 2.7 Hz, 1H, py), 6.46 (d, *J* = 8.0 Hz, 2H, phenyl), 5.37 (dd, *J* = 6.7 and 2.7 Hz, 1H, py), 5.01 (s, 1H, N*H*), 2.26 (s, 3H, tolylC*H*_3_), 2.18 (s, 30H, CpC*H*_3_), 1.88 (s, 6H, N­(C*H*_3_)_2_) ppm. ^13^C­{^1^H} NMR (C_6_D_6_): δ 155.6 (py *C*), 154.4 (py *C*), 153.3 (py *C*), 149.6 (phenyl *C*), 145.3 (phenyl *C*), 129.7 (phenyl *C*), 124.7 (phenyl *C*), 119.8 (ring *C*), 118.2 (py *C*), 103.3 (py *C*), 37.9 (N*C*H_3_), 20.8 (tolyl*C*H_3_), 12.8 (Cp*C*H_3_) ppm. ^125^Te­{^1^H} NMR (C_6_D_6_): δ 818.1 ppm. IR (KBr, cm^–1^): ν 2901 (s), 2858 (s), 1589 (s), 1502 (s), 1438 (s), 1374 (s), 1262 (s), 1133 (s), 1069 (s), 998 (s), 811 (s). Anal. Calcd for C_34_H_47_N_3_TeTh: C, 47.63; H, 5.53; N, 4.90. Found: C, 47.65; H, 5.51; N, 4.92.

#### Method B

##### NMR Scale

Te (2.6 mg, 0.02 mmol) was slowly added to a J. Young NMR tube charged with (η^5^-C_5_Me_5_)_2_Th=N­(*p*-tolyl)­(dmap)_2_ (**5**; 17.0 mg, 0.02 mmol) and C_6_D_6_ (0.5 mL). Resonances of **13** and those of dmap were observed by ^1^H NMR spectroscopy (100% conversion) when this solution was kept at 120 °C overnight.

### Preparation of (η^5^-C_5_Me_5_)_2_ThF_2_(dmap) (**14**)

#### Method A

This compound was prepared as colorless crystals from the reaction of (η^5^-C_5_Me_5_)_2_Th=N­(*p*-tolyl)­(dmap)_2_ (**5**; 213 mg, 0.25 mmol) and AgF (64 mg, 0.50 mmol) in refluxing toluene (15 mL) and recrystallization from a benzene solution by a similar procedure as that in the synthesis of **9**. The product was isolated by filtration, rapidly washed with cooled *n*-hexane (2 mL), and dried at room temperature under vacuum overnight. Yield: 146 mg (88%). M.p.: 120–122 °C (dec.). ^1^H NMR (C_6_D_6_): δ 8.84 (s, 2H, py), 5.86 (d, *J* = 5.8 Hz, 2H, py), 2.18 (s, 30H, CpC*H*_3_), 1.99 (s, 6H, NC*H*_3_) ppm. ^13^C­{^1^H} NMR (C_6_D_6_): δ 150.8 (py *C*), 122.1 (ring *C*), 106.2 (py *C*), 38.0 (N*C*H_3_), 10.9 (Cp*C*H_3_) ppm; one carbon of dmap was not observed. IR (KBr, cm^–1^): ν 2962 (s), 2922 (s), 1606 (s), 1523 (s), 1396 (s), 1261 (s), 1091 (s), 1024 (s), 802 (s). Anal. Calcd for C_27_H_40_N_2_F_2_Th: C, 48.94; H, 6.08; N, 4.23. Found: C, 48.95; H, 6.11; N, 4.22.

#### Method B

##### NMR Scale

AgF (5.1 mg, 0.04 mmol) was slowly added to a J. Young NMR tube charged with (η^5^-C_5_Me_5_)_2_Th=N­(*p*-tolyl)­(dmap)_2_ (**5**; 17.0 mg, 0.02 mmol) and C_6_D_6_ (0.5 mL). Resonances of **14** and those of dmap and other unidentified compounds were observed by ^1^H NMR spectroscopy (100% conversion) when this solution was kept at 120 °C overnight.

### Preparation of (η^5^-C_5_Me_5_)_2_ThCl_2_(dmap)_2_ (**15**)

#### Method A

This compound was prepared as colorless crystals from the reaction of (η^5^-C_5_Me_5_)_2_Th=N­(*p*-tolyl)­(dmap)_2_ (**5**; 213 mg, 0.25 mmol) and CuCl (50 mg, 0.50 mmol) in toluene (15 mL) at room temperature and recrystallization from a benzene solution by a similar procedure as that in the synthesis of **9**. The product was isolated by filtration, rapidly washed with cooled *n*-hexane (2 mL), and dried at room temperature under vacuum overnight. Yield: 172 mg (84%). M.p.: 166–168 °C (dec.). ^1^H NMR (C_6_D_6_): δ 8.66 (d, *J* = 6.5 Hz, 4H, py), 5.92 (d, *J* = 6.2 Hz, 4H, py), 2.30 (s, 30H, CpC*H*_3_), 2.11 (s, 12H, N­(C*H*_3_)_2_) ppm. ^13^C­{^1^H} NMR (C_6_D_6_): δ 154.1 (py *C*), 151.0 (py *C*), 126.2 (ring *C*), 106.2 (py *C*), 38.1 (N*C*H_3_), 12.6 (Cp*C*H_3_) ppm. IR (KBr, cm^–1^): ν 2964 (s), 2933 (s), 2887 (s), 1605 (s), 1527 (s), 1380 (s), 1222 (s), 803 (s). Anal. Calcd for C_34_H_50_N_4_Cl_2_Th: C, 49.94; H, 6.16; N, 6.85. Found: C, 49.95; H, 6.13; N, 6.82.

#### Method B

##### NMR Scale

CuCl (4.0 mg, 0.04 mmol) was slowly added to a J. Young NMR tube charged with (η^5^-C_5_Me_5_)_2_Th=N­(*p*-tolyl)­(dmap)_2_ (**5**; 17.0 mg, 0.02 mmol) and C_6_D_6_ (0.5 mL). Resonances of **15** and those of dmap and other unidentified compounds were observed by ^1^H NMR spectroscopy (100% conversion) when this solution was kept at room temperature overnight.

### Preparation of (η^5^-C_5_Me_5_)_2_ThBr_2_(dmap)·2C_6_H_6_ (16·2C_6_H_6_)

#### Method A

This compound was prepared as colorless crystals from the reaction of (η^5^-C_5_Me_5_)_2_Th=N­(*p*-tolyl)­(dmap)_2_ (**5**; 213 mg, 0.25 mmol) and CuBr (72 mg, 0.50 mmol) in toluene (15 mL) at room temperature and recrystallization from a benzene solution by a similar procedure as that in the synthesis of **9**. The product was isolated by filtration, rapidly washed with cooled *n*-hexane (2 mL), and dried at room temperature under vacuum overnight. Yield: 202 mg (86%). M.p.: 146–148 °C (dec.). ^1^H NMR (C_6_D_6_): δ 8.55 (s, 2H, py), 7.15 (s, 12H, C_6_ *H*_6_), 5.79 (s, 2H, py), 2.31 (s, 30H, CpC*H*_3_), 2.03 (s, 6H, N­(C*H*_3_)_2_) ppm. ^13^C­{^1^H} NMR (C_6_D_6_): δ 154.2 (py *C*), 150.4 (py *C*), 128.5 (*C*_6_H_6_),127.6 (ring *C*), 105.8 (py *C*), 38.1 (N*C*H_3_), 13.2 (Cp*C*H_3_) ppm. IR (KBr, cm^–1^): ν 2908 (s), 2859 (s), 1616 (s), 1535 (s), 1442 (s), 1384 (s), 1230 (s), 1004 (s), 809 (s). Anal. Calcd for C_39_H_52_N_2_Br_2_Th: C, 49.80; H, 5.57; N, 2.98. Found: C, 49.83; H, 5.55; N, 3.01.

#### Method B

##### NMR Scale

CuBr (5.8 mg, 0.04 mmol) was slowly added to a J. Young NMR tube charged with (η^5^-C_5_Me_5_)_2_Th=N­(*p*-tolyl)­(dmap)_2_ (**5**; 17.0 mg, 0.02 mmol) and C_6_D_6_ (0.5 mL). Resonances of **16** and those of dmap and other unidentified compounds were observed by ^1^H NMR spectroscopy (100% conversion) when this solution was kept at room temperature overnight.

### Preparation of (η^5^-C_5_Me_5_)_2_Th­(Cl)­[κ^2^-*C*,*N*-4-(Me_2_N)­C_5_H_3_N] (**17**)

#### Method A

This compound was prepared as colorless crystals from the reaction of (η^5^-C_5_Me_5_)_2_Th=N­(*p*-tolyl)­(dmap)_2_ (**5**; 213 mg, 0.25 mmol) and PhSiH_2_Cl (36 mg, 0.25 mmol) in toluene (15 mL) at room temperature and recrystallization from a benzene solution by a similar procedure as that in the synthesis of **6**. The product was isolated by filtration, rapidly washed with cooled *n*-hexane (2 mL), and dried at room temperature under vacuum overnight. Yield: 132 mg (80%). M.p.: 208–210 °C (dec.). ^1^H NMR (C_6_D_6_): δ 8.11 (d, *J* = 6.5 Hz, 1H, py), 7.03 (d, *J* = 2.3 Hz, 1H, py), 5.99 (dd, *J* = 6.2 and 2.6 Hz, 1H, py), 2.30 (s, 6H, N­(C*H*_3_)_2_), 2.06 (s, 30H, CpC*H*_3_) ppm. ^13^C­{^1^H} NMR (C_6_D_6_): δ 233.0 (Th*C*), 154.9 (py *C*), 143.7 (py *C*), 123.6 (ring *C*), 110.4 (py *C*), 109.1 (py *C*), 38.7 (N*C*H_3_), 11.4 (Cp*C*H_3_) ppm. IR (KBr, cm^–1^): ν 2964 (s), 2932 (s), 1617 (s), 1578 (s), 1431 (s), 1377 (s), 1259 (s), 1019 (s), 989 (s), 803 (s). Anal. Calcd for C_27_H_39_N_2_ClTh: C, 49.20; H, 5.96; N, 4.25. Found: C, 49.22; H, 5.94; N, 4.27.

#### Method B

##### NMR Scale

A C_6_D_6_ (0.3 mL) solution of PhSiH_2_Cl (2.9 mg, 0.02 mmol) was slowly added to a J. Young NMR tube charged with (η^5^-C_5_Me_5_)_2_Th=N­(*p*-tolyl)­(dmap)_2_ (**5**; 17.0 mg, 0.02 mmol) and C_6_D_6_ (0.2 mL). Resonances of **17** and those of dmap and *p*-tolylNHSiH_2_Ph^10^ were observed by ^1^H NMR spectroscopy (100% conversion) when this solution was kept at room temperature overnight.

### Preparation of (η^5^-C_5_Me_5_)_2_Th­[κ^3^-*C*,*N,N*-(4-Me_2_NC_5_H_3_N)­SiH­(Ph)­N­(4-MeC_6_H_3_)] (**18**)

#### Method A

This compound was prepared as yellow crystals from the reaction of (η^5^-C_5_Me_5_)_2_Th=N­(*p*-tolyl)­(dmap)_2_ (**5**; 213 mg, 0.25 mmol) and PhSiH_3_ (27 mg, 0.25 mmol) in toluene (15 mL) at room temperature and recrystallization from a benzene solution by a similar procedure as that in the synthesis of **6**. The product was isolated by filtration, rapidly washed with cooled *n*-hexane (2 mL), and dried at room temperature under vacuum overnight. Yield: 175 mg (84%). M.p.: 286–288 °C (dec.). ^1^H NMR (C_6_D_6_): δ 8.06 (m, 4H, phenyl), 7.31 (t, *J* = 7.3 Hz, 2H, phenyl), 7.23 (m, 1H, py), 6.98 (m, 1H, phenyl), 6.70 (d, *J* = 2.9 Hz, 1H, py), 6.64 (d, *J* = 7.5 Hz, 1H, phenyl), 6.42 (s, 1H, Si*H*), 6.06 (dd, *J* = 6.4 and 3.0 Hz, 1H, py), 2.58 (s, 3H, tolylC*H*_3_), 2.04 (s, 15H, CpC*H*_3_), 1.99 (s, 6H, N­(C*H*_3_)_2_), 1.97 (s, 15H, CpC*H*_3_) ppm. ^13^C­{^1^H} NMR (C_6_D_6_): δ 192.0 (Th*C*), 168.6 (aryl *C*), 153.4 (aryl *C*), 152.9 (aryl *C*), 147.2 (aryl *C*), 139.0 (aryl *C*), 136.2 (aryl *C*), 135.2 (aryl *C*), 130.0 (aryl *C*), 129.3 (aryl *C*), 128.4 (aryl *C*), 127.0 (aryl *C*), 122.3 (ring *C*), 115.9 (aryl *C*), 115.0 (aryl *C*), 104.7 (aryl *C*), 37.9 (N*C*H_3_), 22.2 (tolyl*C*H_3_), 11.9 (Cp*C*H_3_), 11.6 (Cp*C*H_3_) ppm. ^29^Si­{^1^H} NMR (C_6_D_6_): δ −27.9 ppm. IR (KBr, cm^–1^): ν 2903 (s), 2856 (s), 2066 (s, Si–H), 1598 (s), 1536 (s), 1427 (s), 1378 (s), 1262 (s), 1217 (s), 1001 (s), 968 (s), 818 (s). Anal. Calcd for C_40_H_51_N_3_SiTh: C, 57.61; H, 6.61; N, 5.04. Found: C, 57.62; H, 6.64; N, 5.01.

#### Method B

##### NMR Scale

A C_6_D_6_ (0.3 mL) solution of PhSiH_3_ (2.2 mg, 0.02 mmol) was slowly added to a J. Young NMR tube charged with (η^5^-C_5_Me_5_)_2_Th=N­(*p*-tolyl)­(dmap)_2_ (**5**; 17.0 mg, 0.02 mmol) and C_6_D_6_ (0.2 mL). Resonances of **18** and those of dmap and H_2_ were observed by ^1^H NMR spectroscopy (100% conversion) when this solution was kept at room temperature overnight.

### Preparation of (η^5^-C_5_Me_5_)_2_Th­[κ^3^-*C*,*N,N*-(4-Me_2_NC_5_H_3_N)­SiPh_2_N­(4-MeC_6_H_3_)]·C_6_H_6_ (19·C_6_H_6_)

#### Method A

This compound was prepared as yellow crystals from the reaction of (η^5^-C_5_Me_5_)_2_Th=N­(*p*-tolyl)­(dmap)_2_ (**5**; 213 mg, 0.25 mmol) and Ph_2_SiH_2_ (46 mg, 0.25 mmol) in toluene (15 mL) at 50 °C and recrystallization from a benzene solution by a similar procedure as that in the synthesis of **6**. The product was isolated by filtration, rapidly washed with cooled *n*-hexane (2 mL), and dried at room temperature under vacuum overnight. Yield: 203 mg (82%). M.p.: 210–212 °C (dec.). ^1^H NMR (C_6_D_6_): δ 8.11 (d, *J* = 6.3 Hz, 1H, py), 8.07 (s, 1H, phenyl), 8.03 (d, *J* = 6.5 Hz, 4H, phenyl), 7.23 (m, 6H, phenyl), 7.15 (s, 6H, C_6_ *H*_6_), 6.99 (d, *J* = 8.0 Hz, 1H, phenyl), 6.90 (m, 2H, phenyl and py), 6.11 (d, 1H, *J* = 3.6 Hz, py), 2.58 (s, 3H, tolylC*H*_3_), 2.04 (s, 6H, N­(C*H*_3_)_2_), 1.97 (s, 30H, CpC*H*_3_) ppm. ^13^C­{^1^H} NMR (C_6_D_6_): δ 192.3 (Th*C*), 169.1 (aryl *C*), 153.5 (aryl *C*), 153.3 (aryl *C*), 147.2 (aryl *C*), 139.8 (aryl *C*), 136.0 (aryl *C*), 129.5 (aryl *C*), 129.3 (aryl *C*), 128.53 (aryl *C*), 128.50 (*C*_6_H_6_), 126.6 (aryl *C*), 125.6 (aryl *C*), 122.4 (ring *C*), 116.7 (aryl *C*), 115.9 (aryl *C*), 104.8 (aryl *C*), 38.0 (N*C*H_3_), 22.2 (tolyl*C*H_3_), 11.9 (Cp*C*H_3_) ppm. ^29^Si­{^1^H} NMR (C_6_D_6_): δ −21.6 ppm. IR (KBr, cm^–1^): ν 2924 (s), 2855 (s), 1597 (s), 1426 (s), 1378 (s), 1263 (s), 1214 (s), 1105 (s), 1002 (s), 808 (s). Anal. Calcd for C_52_H_61_N_3_SiTh: C, 63.20; H, 6.22; N, 4.25. Found: C, 63.22; H, 6.24; N, 4.22.

#### Method B

##### NMR Scale

A C_6_D_6_ (0.3 mL) solution of Ph_2_SiH_2_ (3.7 mg, 0.02 mmol) was slowly added to a J. Young NMR tube charged with (η^5^-C_5_Me_5_)_2_Th=N­(*p*-tolyl)­(dmap)_2_ (**5**; 17.0 mg, 0.02 mmol) and C_6_D_6_ (0.2 mL). Resonances of **19** and those of dmap and H_2_ were observed by ^1^H NMR spectroscopy (100% conversion) when this solution was kept at 50 °C overnight.

### Preparation of (η^5^-C_5_Me_5_)_2_Th­[N­(*p*-tolyl)­C­(Ph)=CHPh]­[κ^2^-*C*,*N*-4-(Me_2_N)­C_5_H_3_N] (**20**)

#### Method A

This compound was prepared as yellow crystals from the reaction of (η^5^-C_5_Me_5_)_2_Th=N­(*p*-tolyl)­(dmap)_2_ (**5**; 213 mg, 0.25 mmol) and PhC≡CPh (45 mg, 0.25 mmol) in toluene (15 mL) at 80 °C and recrystallization from a benzene solution by a similar procedure as that in the synthesis of **6**. The product was isolated by filtration, rapidly washed with cooled *n*-hexane (2 mL), and dried at room temperature under vacuum overnight. Yield: 186 mg (82%). M.p.: 180–182 °C (dec.). ^1^H NMR (C_6_D_6_): δ 7.83 (d, *J* = 7.0 Hz, 1H, py), 7.35 (d, *J* = 7.0 Hz, 2H, phenyl), 7.27 (d, *J* = 7.3 Hz, 2H, phenyl), 7.00 (m, 3H, phenyl), 6.88 (m, 3H, phenyl), 6.77 (s, 4H, phenyl), 6.46 (d, *J* = 5.9 Hz, 1H, py), 6.06 (s, 1H, C*H*=C), 5.89 (d, *J* = 4.0 Hz, 1H, py), 2.31 (s, 6H, N­(C*H*_3_)_2_), 2.22 (s, 3H, tolylC*H*_3_), 2.16 (s, 30H, CpC*H*_3_) ppm. ^13^C­{^1^H} NMR (C_6_D_6_): δ 232.4 (Th*C*), 157.6 (aryl *C*), 154.1 (aryl *C*), 151.9 (aryl *C*), 143.6 (aryl *C*), 141.6 (aryl *C*), 141.5 (aryl *C*), 131.9 (aryl *C*), 130.7 (aryl *C*), 130.1 (aryl *C*), 129.3 (aryl *C*), 128.6 (aryl *C*), 126.7 (aryl *C*), 123.6 (ring *C*), 122.3 (aryl *C*), 120.4 (aryl *C*), 117.9 (aryl *C*), 109.1 (aryl *C*), 108.0 (*C*=CH), 98.5 (C=*C*H), 38.6 (N*C*H_3_), 20.8 (tolyl*C*H_3_), 12.2 (Cp*C*H_3_) ppm. IR (KBr, cm^–1^): ν 2903 (s), 2851 (s), 1578 (s), 1500 (s), 1430 (s), 1364 (s), 1257 (s), 1098 (s), 993 (s), 806 (s). Anal. Calcd for C_48_H_57_N_3_Th: C, 63.49; H, 6.33; N, 4.63. Found: C, 63.52; H, 6.34; N, 4.62.

#### Method B

##### NMR Scale

A C_6_D_6_ (0.3 mL) solution of PhC≡CPh (3.6 mg, 0.02 mmol) was slowly added to a J. Young NMR tube charged with (η^5^-C_5_Me_5_)_2_Th=N­(*p*-tolyl)­(dmap)_2_ (**5**; 17.0 mg, 0.02 mmol) and C_6_D_6_ (0.2 mL). Resonances of **20** and those of dmap were observed by ^1^H NMR spectroscopy (100% conversion) when this solution was kept at 80 °C overnight.

### Preparation of (η^5^-C_5_Me_5_)_2_Th­[N­(*p*-tolyl)­C­(=NC_6_H_11_)­N­(C_6_H_11_)]­(dmap) (**21**)

#### Method A

This compound was prepared as colorless crystals from the reaction of (η^5^-C_5_Me_5_)_2_Th=N­(*p*-tolyl)­(dmap)_2_ (**5**; 213 mg, 0.25 mmol) and DCC (52 mg, 0.25 mmol) in toluene (15 mL) at room temperature and recrystallization from a benzene solution by a similar procedure as that in the synthesis of **6**. The product was isolated by filtration, rapidly washed with cooled *n*-hexane (2 mL), and dried at room temperature under vacuum overnight. Yield: 201 mg (86%). M.p.: 158–160 °C (dec.). ^1^H NMR (C_6_D_6_): δ 8.46 (d, *J* = 6.4 Hz, 2H, py), 7.13 (d, *J* = 8.2 Hz, 2H, phenyl), 7.03 (d, *J* = 8.2 Hz, 2H, phenyl), 6.11 (d, *J* = 6.4 Hz, 2H, py), 4.04 (m, 1H, NC*H*), 3.70 (m, 1H, NC*H*), 2.46 (m, 2H, Cy), 2.30 (s, 3H, tolylC*H*_3_), 2.20 (s, 6H, N­(C*H*_3_)_2_), 2.06 (m, 2H, *C*H_2_), 1.96 (s, 30H, CpC*H*_3_), 1.87 (m, 4H, *C*H_2_), 1.75 (m, 4H, *C*H_2_), 1.52 (m, 4H, *C*H_2_), 1.34 (m, 4H, *C*H_2_) ppm. ^13^C­{^1^H} NMR (C_6_D_6_): δ 154.1 (py *C*), 150.5 (py *C*), 147.3 (phenyl *C*), 146.7 (phenyl *C*), 129.4 (phenyl *C*), 127.3 (phenyl *C*), 126.4 (ring *C*), 120.4 (*C*=N), 106.8 (py *C*), 55.7 (N*C*H), 54.8 (N*C*H), 38.2 (N*C*H_3_), 36.7 (Cy *C*), 34.9 (Cy *C*), 27.3 (Cy *C*), 27.1 (Cy *C*), 26.6 (Cy *C*), 25.5 (Cy *C*), 20.9 (tolyl*C*H_3_), 11.4 (Cp*C*H_3_) ppm. IR (KBr, cm^–1^): ν 2925 (s), 2852 (s), 1605 (s), 1551 (s), 1506 (s), 1446 (s), 1276 (s), 1230 (s), 1002 (s), 808 (s). Anal. Calcd for C_47_H_69_N_5_Th: C, 60.30; H, 7.43; N, 7.48. Found: C, 60.32; H, 7.44; N, 7.51.

#### Method B

##### NMR Scale

A C_6_D_6_ (0.3 mL) solution of DCC (4.1 mg, 0.02 mmol) was slowly added to a J. Young NMR tube charged with (η^5^-C_5_Me_5_)_2_Th=N­(*p*-tolyl)­(dmap)_2_ (**5**; 17.0 mg, 0.02 mmol) and C_6_D_6_ (0.2 mL). Resonances of **21** and those of dmap were observed by ^1^H NMR spectroscopy (100% conversion) when this solution was kept at room temperature overnight.

### Preparation of (η^5^-C_5_Me_5_)_2_Th­[N­(*p*-tolyl)­C­(=N*^i^* Pr)­N­(*^i^* Pr)]­(dmap) (**22**)

#### Method A

This compound was prepared as colorless crystals from the reaction of (η^5^-C_5_Me_5_)_2_Th=N­(*p*-tolyl)­(dmap)_2_ (**5**; 213 mg, 0.25 mmol) and DIC (32 mg, 0.25 mmol) in toluene (15 mL) at room temperature and recrystallization from a benzene solution by a similar procedure as that in the synthesis of **6**. The product was isolated by filtration, rapidly washed with cooled *n*-hexane (2 mL), and dried at room temperature under vacuum overnight. Yield: 176 mg (82%). M.p.: 114–116 °C (dec.). ^1^H NMR (C_6_D_6_): δ 8.46 (d, *J* = 5.5 Hz, 2H, py), 7.13 (d, *J* = 7.7 Hz, 2H, phenyl), 7.05 (d, *J* = 7.7 Hz, 2H, phenyl), 6.10 (d, *J* = 5.3 Hz, 2H, py), 4.43 (s, 1H, NC*H*), 4.09 (m, 1H, NC*H*), 2.31 (s, 3H, tolylC*H*_3_), 2.19 (s, 6H, N­(C*H*_3_)_2_), 1.94 (s, 30H, CpC*H*_3_), 1.60 (d, *J* = 6.1 Hz, 6H, CH­(C*H*_3_)_2_), 1.41 (d, *J* = 5.9 Hz, 6H, CH­(C*H*_3_)_2_) ppm. ^13^C­{^1^H} NMR (C_6_D_6_): δ 154.1 (py *C*), 150.5 (py *C*), 137.5 (phenyl *C*), 134.4 (phenyl *C*), 126.1 (ring *C*), 123.6 (phenyl *C*), 122.4 (phenyl *C*), 118.8 (*C*=N), 106.8 (py *C*), 51.8 (N*C*H), 46.7 (N*C*H), 38.3 (N*C*H_3_), 26.5 (CH­(*C*H_3_)_2_), 20.9 (tolyl*C*H_3_), 11.7 (CH­(*C*H_3_)_2_), 11.2 (Cp*C*H_3_) ppm. IR (KBr, cm^–1^): *v* 2963 (s), 2919 (s), 2857 (s), 1600 (s), 1541 (s), 1505 (s), 1383 (s), 1262 (s), 1228 (s), 987 (s), 804 (s). Anal. Calcd for C_41_H_61_N_5_Th: C, 57.53; H, 7.18; N, 8.18. Found: C, 57.52; H, 7.15; N, 8.21.

#### Method B

##### NMR Scale

A C_6_D_6_ (0.3 mL) solution of DIC (2.5 mg, 0.02 mmol) was slowly added to a J. Young NMR tube charged with (η^5^-C_5_Me_5_)_2_Th=N­(*p*-tolyl)­(dmap)_2_ (**5**; 17.0 mg, 0.02 mmol) and C_6_D_6_ (0.2 mL). Resonances of **22** and those of dmap were observed by ^1^H NMR spectroscopy (100% conversion) when this solution was kept at room temperature overnight.

### Preparation of (η^5^-C_5_Me_5_)_2_Th­[N­(*p*-tolyl)­CPh_2_O]­(dmap) (**23**)

#### Method A

This compound was prepared as colorless crystals from the reaction of (η^5^-C_5_Me_5_)_2_Th=N­(*p*-tolyl)­(dmap)_2_ (**5**; 213 mg, 0.25 mmol) and Ph_2_C=O (46 mg, 0.25 mmol) in toluene (15 mL) at room temperature and recrystallization from a benzene solution by a similar procedure as that in the synthesis of **6**. The product was isolated by filtration, rapidly washed with cooled *n*-hexane (2 mL), and dried at room temperature under vacuum overnight. Yield: 187 mg (82%). M.p.: 62–64 °C (dec.). ^1^H NMR (C_6_D_6_): δ 8.84 (s, 1H, py), 8.44 (s, 1H, py), 8.01 (d, *J* = 7.5 Hz, 2H, phenyl), 7.90 (d, *J* = 7.5 Hz, 2H, phenyl), 7.21 (m, 6H, phenyl), 7.10 (m, 4H, phenyl), 6.08 (s, 1H, py), 5.95 (s, 1H, py), 2.35 (s, 3H, tolylC*H*_3_), 2.03 (s, 30H, CpC*H*_3_), 2.01 (s, 6H, N­(C*H*_3_)_2_) ppm. ^13^C­{^1^H} NMR (C_6_D_6_): δ 156.2 (phenyl *C*), 154.1 (py *C*), 150.6 (py *C*), 138.3 (phenyl *C*), 132.0 (phenyl *C*), 130.1 (phenyl *C*), 129.9 (phenyl *C*), 126.9 (phenyl *C*), 124.8 (phenyl *C*), 123.4 (ring *C*), 121.4 (phenyl *C*), 106.8 (py *C*), 92.1 (*C*O), 38.3 (N*C*H_3_), 20.7 (tolyl*C*H_3_), 11.8 (Cp*C*H_3_) ppm. IR (KBr, cm^–1^): ν 2904 (s), 1614 (s), 1537 (s), 1445 (s), 1378 (s), 1226 (s), 1021 (s), 1002 (s), 807 (s). Anal. Calcd for C_47_H_57_N_3_OTh: C, 61.90; H, 6.30; N, 4.61. Found: C, 61.92; H, 6.32; N, 4.59.

#### Method B

##### NMR Scale

A C_6_D_6_ (0.3 mL) solution of Ph_2_C=O (3.6 mg, 0.02 mmol) was slowly added to a J. Young NMR tube charged with (η^5^-C_5_Me_5_)_2_Th=N­(*p*-tolyl)­(dmap)_2_ (**5**; 17.0 mg, 0.02 mmol) and C_6_D_6_ (0.2 mL). Resonances of **23** and those of dmap were observed by ^1^H NMR spectroscopy (100% conversion) when this solution was kept at room temperature overnight.

### Preparation of (η^5^-C_5_Me_5_)_2_Th­[OCPh_2_O]­(dmap)·C_6_H_6_ (24·C_6_H_6_)

#### Method A

This compound was prepared as colorless crystals from the reaction of (η^5^-C_5_Me_5_)_2_Th=N­(*p*-tolyl)­(dmap)_2_ (**5**; 213 mg, 0.25 mmol) and Ph_2_C=O (91 mg, 0.50 mmol) in toluene (15 mL) at 80 °C and recrystallization from a benzene solution by a similar procedure as that in the synthesis of **6**. The product was isolated by filtration, rapidly washed with cooled *n*-hexane (2 mL), and dried at room temperature under vacuum overnight. Yield: 189 mg (84%). ^1^H NMR (C_6_D_6_): δ 9.00 (br s, 2H, py), 8.19 (d, *J* = 7.7 Hz, 4H, phenyl), 7.30 (t, *J* = 7.4 Hz, 4H, phenyl), 7.15 (s, 6H, C_6_ *H*_6_), 7.06 (t, *J* = 6.9 Hz, 2H, phenyl), 6.07 (d, *J* = 6.0 Hz, 2H, py), 2.08 (s, 6H, N­(C*H*_3_)_2_), 1.93 (s, 30H, CpC*H*_3_) ppm. These spectroscopic data agreed with those reported in the literature.^7g^ Furthermore, this complex was also characterized by X-ray diffraction analysis, and its molecular structure is shown in the Supporting Information (Figure S7).

#### Method B

##### NMR Scale

A C_6_D_6_ (0.3 mL) solution of Ph_2_C=O (7.3 mg, 0.04 mmol) was slowly added to a J. Young NMR tube charged with (η^5^-C_5_Me_5_)_2_Th=N­(*p*-tolyl)­(dmap)_2_ (**5**; 17.0 mg, 0.02 mmol) and C_6_D_6_ (0.2 mL). Resonances of **24** and those of dmap and (*p*-tolyl)­N=CPh_2_ ^7a^ were observed by ^1^H NMR spectroscopy (100% conversion) when this solution was kept at 80 °C overnight.

### Preparation of (η^5^-C_5_Me_5_)_2_Th­[SCPh_2_S]­(dmap) (**25**)

#### Method A

This compound was prepared as colorless crystals from the reaction of (η^5^-C_5_Me_5_)_2_Th=N­(*p*-tolyl)­(dmap)_2_ (**5**; 213 mg, 0.25 mmol) and Ph_2_C=S (99 mg, 0.50 mmol) in toluene (15 mL) at 50 °C and recrystallization from a benzene solution by a similar procedure as that in the synthesis of **6**. The product was isolated by filtration, rapidly washed with cooled *n*-hexane (2 mL), and dried at room temperature under vacuum overnight. Yield: 184 mg (86%). M.p.: 128–130 °C (dec.). ^1^H NMR (C_6_D_6_): δ 8.74 (d, *J* = 7.8 Hz, 4H, phenyl), 8.60 (br s, 2H, py), 7.27 (t, *J* = 7.6 Hz, 4H, phenyl), 7.05 (m, 2H, phenyl), 5.99 (br s, 2H, py), 2.11 (s, 6H, N­(C*H*_3_)_2_), 2.00 (s, 30H, CpC*H*_3_) ppm. ^13^C­{^1^H} NMR (C_6_D_6_): δ 156.8 (py *C*), 131.7 (py *C*), 129.8 (phenyl *C*), 129.0 (phenyl *C*), 126.9 (phenyl *C*), 125.1 (phenyl *C*), 125.0 (ring *C*), 106.6 (py *C*), 58.4 (*C*S_2_), 38.1 (N*C*H_3_), 12.5 (Cp*C*H_3_) ppm. IR (KBr, cm^–1^): ν 2962 (s), 2923 (s), 1607 (s), 1441 (s), 1384 (s), 1260 (s), 1089 (s), 1019 (s), 800 (s). Anal. Calcd for C_40_H_50_N_2_S_2_Th: C, 56.19; H, 5.89; N, 3.28. Found: C, 56.22; H, 5.91; N, 3.26.

#### Method B

##### NMR Scale

A C_6_D_6_ (0.3 mL) solution of Ph_2_C=S (7.9 mg, 0.04 mmol) was slowly added to a J. Young NMR tube charged with (η^5^-C_5_Me_5_)_2_Th=N­(*p*-tolyl)­(dmap)_2_ (**5**; 17.0 mg, 0.02 mmol) and C_6_D_6_ (0.2 mL). Resonances of **25** and those of dmap and (*p*-tolyl)­N=CPh_2_ were observed by ^1^H NMR spectroscopy (100% conversion) when this solution was kept at 50 °C overnight.

### Preparation of (η^5^-C_5_Me_5_)_2_Th­[SC=N­(*p*-tolyl)­NPh]­(dmap) (**26**)

#### Method A

This compound was prepared as colorless crystals from the reaction of (η^5^-C_5_Me_5_)_2_Th=N­(*p*-tolyl)­(dmap)_2_ (**5**; 213 mg, 0.25 mmol) and PhNCS (34 mg, 0.25 mmol) in toluene (15 mL) at room temperature and recrystallization from a benzene solution by a similar procedure as that in the synthesis of **6**. The product was isolated by filtration, rapidly washed with cooled *n*-hexane (2 mL), and dried at room temperature under vacuum overnight. Yield: 177 mg (82%). M.p.: 240–242 °C (dec.). ^1^H NMR (C_6_D_6_): δ 8.10 (br s, 2H, py), 7.54 (d, *J* = 7.8 Hz, 2H, tolyl), 7.43 (m, 3H, phenyl), 7.22 (d, *J* = 7.8 Hz, 2H, tolyl), 7.02 (m, 2H, phenyl), 5.87 (s, 2H, py), 2.11 (s, 3H, tolylC*H*_3_), 2.08 (s, 6H, N­(C*H*_3_)_2_), 1.98 (s, 30H, CpC*H*_3_) ppm. ^13^C­{^1^H} NMR (C_6_D_6_): δ 154.4 (py *C*), 149.9 (py *C*), 137.8 (phenyl *C*), 129.3 (phenyl *C*), 125.8 (ring *C*), 124.6 (phenyl *C*), 124.5 (phenyl *C*), 124.4 (phenyl *C*), 124.3 (phenyl *C*), 120.7 (phenyl *C*), 120.6 (phenyl *C*), 115.4 (*C*=N), 106.7 (py *C*), 38.1 (N*C*H_3_), 21.4 (tolyl*C*H_3_), 12.3 (Cp*C*H_3_) ppm. Anal. Calcd for C_41_H_52_N_4_STh: C, 56.93; H, 6.06; N, 6.48. Found: C, 56.92; H, 6.08; N, 6.46.

#### Method B

##### NMR Scale

A C_6_D_6_ (0.3 mL) solution of PhNCS (2.7 mg, 0.02 mmol) was slowly added to a J. Young NMR tube charged with (η^5^-C_5_Me_5_)_2_Th=N­(*p*-tolyl)­(dmap)_2_ (**5**; 17.0 mg, 0.02 mmol) and C_6_D_6_ (0.2 mL). Resonances of **26** and those of dmap were observed by ^1^H NMR spectroscopy (100% conversion) when this solution was kept at room temperature overnight.

### Preparation of [(η^5^-C_5_Me_5_)_2_Th]_2_{μ-[N­(*p*-tolyl)­C­(S)­S]}_2_·C_6_H_6_ (27·C_6_H_6_)

#### Method A

This compound was prepared as colorless crystals from the reaction of (η^5^-C_5_Me_5_)_2_Th=N­(*p*-tolyl)­(dmap)_2_ (**5**; 213 mg, 0.25 mmol) and CS_2_ (19 mg, 0.25 mmol) in toluene (15 mL) at room temperature and recrystallization from a benzene solution by a similar procedure as that in the synthesis of **6**. The product was isolated by filtration, rapidly washed with cooled *n*-hexane (2 mL), and dried at room temperature under vacuum overnight. Yield: 160 mg (84%). M.p.: > 300 °C (dec.). ^1^H NMR (C_6_D_6_): δ 7.15 (s, 6H, C_6_ *H*_6_), 7.08 (d, *J* = 6.9 Hz, 4H, phenyl), 6.98 (d, *J* = 6.9 Hz, 4H, phenyl), 2.12 (s, 6H, tolylC*H*_3_), 2.06 (s, 30H, CpC*H*_3_), 1.26 (s, 30H, CpC*H*_3_) ppm. ^13^C­{^1^H} NMR (C_6_D_6_): δ 204.1 (*C*S_2_), 137.9 (phenyl *C*), 129.3 (phenyl *C*), 128.6 (phenyl *C*), 128.5 (*C*_6_H_6_), 128.2 (phenyl *C*), 125.6 (ring *C*), 20.9 (tolyl*C*H_3_), 11.7 (Cp*C*H_3_) ppm. IR (KBr, cm^–1^): ν 2962 (s), 1590 (m), 1436 (m), 1385 (s), 1260 (s), 1091 (s), 1019 (s), 983 (s), 799 (s). Anal. Calcd for C_68_H_86_N_2_S_4_Th_2_: C, 53.60; H, 5.69; N, 1.84. Found: C, 53.62; H, 5.71; N, 1.86.

#### Method B

##### NMR Scale

A C_6_D_6_ (0.3 mL) solution of CS_2_ (1.6 mg, 0.02 mmol) was slowly added to a J. Young NMR tube charged with (η^5^-C_5_Me_5_)_2_Th=N­(*p*-tolyl)­(dmap)_2_ (**5**; 17.0 mg, 0.02 mmol) and C_6_D_6_ (0.2 mL). Resonances of **27** and those of dmap were observed by ^1^H NMR spectroscopy (100% conversion) when this solution was kept at room temperature overnight.

### Preparation of (η^5^-C_5_Me_5_)_2_Th­[N­(*p*-tolyl)­CH_2_C­(Me)=C­(OMe)­O]­(dmap) (**28**)

#### Method A

This compound was prepared as colorless crystals from the reaction of (η^5^-C_5_Me_5_)_2_Th=N­(*p*-tolyl)­(dmap)_2_ (**5**; 213 mg, 0.25 mmol) and MMA (36 mg, 0.25 mmol) in toluene (15 mL) at room temperature and recrystallization from a benzene solution by a similar procedure as that in the synthesis of **6**. The product was isolated by filtration, rapidly washed with cooled *n*-hexane (2 mL), and dried at room temperature under vacuum overnight. Yield: 170 mg (82%). M.p.: 76–78 °C (dec.). ^1^H NMR (C_6_D_6_): δ 8.45 (d, *J* = 5.4 Hz, 2H, py), 7.13 (d, 2H, *J* = 8.1 Hz, phenyl), 6.57 (d, 2H, *J* = 8.1 Hz, phenyl), 6.10 (d, *J* = 5.8 Hz, 2H, py), 3.96 (s, 2H, C*H*_2_), 3.70 (s, 3H, OC*H*_3_), 2.26 (s, 3H, C*H*_3_), 2.22 (s, 6H, N­(C*H*_3_)_2_), 2.08 (s, 3H, tolylC*H*_3_), 1.93 (s, 30H, CpC*H*_3_) ppm. ^13^C­{^1^H} NMR (C_6_D_6_): δ 154.1 (py *C*), 153.6 (phenyl *C*), 150.6 (py *C*), 149.7 (phenyl *C*), 133.3 (phenyl *C*), 128.6 (phenyl *C*), 125.6 (phenyl *C*), 125.1 (ring *C*), 108.6 (*C*O), 106.9 (py *C*), 81.9 (*C*=CO), 53.4 (O*C*H_3_), 53.1 (*C*H_2_), 38.3 (N*C*H_3_), 20.6 (tolyl*C*H_3_), 15.7 (*C*H_3_), 11.0 (Cp*C*H_3_) ppm. IR (KBr, cm^–1^): ν 2902 (s), 2856 (s), 1655 (s), 1623 (s), 1545 (s), 1435 (s), 1387 (s), 1261 (s), 1246 (s), 1143 (s), 1000 (s), 807 (s). Anal. Calcd for C_39_H_55_N_3_O_2_Th: C, 56.44; H, 6.68; N, 5.06. Found: C, 56.42; H, 6.71; N, 5.04.

#### Method B

##### NMR Scale

A C_6_D_6_ (0.3 mL) solution of MMA (2.9 mg, 0.02 mmol) was slowly added to a J. Young NMR tube charged with (η^5^-C_5_Me_5_)_2_Th=N­(*p*-tolyl)­(dmap)_2_ (**5**; 17.0 mg, 0.02 mmol) and C_6_D_6_ (0.2 mL). Resonances of **28** and those of dmap were observed by ^1^H NMR spectroscopy (100% conversion) when this solution was kept at room temperature overnight.

### Preparation of [(η^5^-C_5_Me_5_)_2_Th]_2_{μ-[OCH­(Me)­C­(=O)­OCH­(Me)­C­(=N-*p*-tolyl)­O]}_2_ (**29**)

#### Method A

This compound was prepared as colorless crystals from the reaction of (η^5^-C_5_Me_5_)_2_Th=N­(*p*-tolyl)­(dmap)_2_ (**5**; 213 mg, 0.25 mmol) and *rac*-lactide (36 mg, 0.25 mmol) in toluene (15 mL) at room temperature and recrystallization from a benzene solution by a similar procedure as that in the synthesis of **6**. The product was isolated by filtration, rapidly washed with cooled *n*-hexane (2 mL), and dried at room temperature under vacuum overnight. Yield: 150 mg (80%). M.p.: 110–112 °C (dec.). ^1^H NMR (C_6_D_6_): δ 7.56 (d, *J* = 8.0 Hz, 4H, phenyl), 7.21 (d, *J* = 8.0 Hz, 4H, phenyl), 5.71 (m, 2H, OC*H*), 4.90 (m, 2H, OC*H*), 2.27 (s, 12H, C*H*_3_), 1.96 (s, 30H, CpC*H*_3_), 1.93 (s, 6H, tolylC*H*_3_), 1.81 (s, 30H, CpC*H*_3_) ppm. ^13^C­{^1^H} NMR (C_6_D_6_): δ 175.0 (*C*O_2_), 163.8 (*C*ON), 146.8 (phenyl *C*), 132.1 (phenyl *C*), 129.2 (phenyl *C*), 124.8 (ring *C*), 124.5 (ring *C*), 123.1 (phenyl *C*), 80.3 (O*C*H), 73.5 (O*C*H), 30.2 (*C*H_3_), 23.9 (*C*H_3_), 20.5 (tolyl*C*H_3_), 11.3 (Cp*C*H_3_), 10.8 (Cp*C*H_3_) ppm. IR (KBr, cm^–1^): ν 2917 (s), 1676 (s), 1632 (s), 1526 (s), 1404 (s), 1384 (s), 1251 (s), 1151 (s), 1031 (s), 934 (s), 809 (s). Anal. Calcd for C_66_H_90_N_2_O_8_Th_2_: C, 52.72; H, 6.03; N, 1.86. Found: C, 52.74; H, 6.01; N, 1.84.

#### Method B

##### NMR Scale

A C_6_D_6_ (0.3 mL) solution of *rac*-lactide (2.9 mg, 0.02 mmol) was slowly added to a J. Young NMR tube charged with (η^5^-C_5_Me_5_)_2_Th=N­(*p*-tolyl)­(dmap)_2_ (**5**; 17.0 mg, 0.02 mmol) and C_6_D_6_ (0.2 mL). Resonances of **29** and those of dmap were observed by ^1^H NMR spectroscopy (100% conversion) when this solution was kept at room temperature overnight.

### Preparation of (η^5^-C_5_Me_5_)_2_Th­[OC­(Ph)­N­(*p*-tolyl)]_2_ (**30**)

#### Method A

This compound was prepared as colorless crystals from the reaction of (η^5^-C_5_Me_5_)_2_Th=N­(*p*-tolyl)­(dmap)_2_ (**5**; 213 mg, 0.25 mmol) and PhCONH­(*p*-tolyl) (106 mg, 0.50 mmol) in toluene (15 mL) at room temperature and recrystallization from a benzene solution by a similar procedure as that in the synthesis of **6**. The product was isolated by filtration, rapidly washed with cooled *n*-hexane (2 mL), and dried at room temperature under vacuum overnight. Yield: 194 mg (84%). M.p.: 170–172 °C (dec.). ^1^H NMR (C_6_D_6_): δ 7.64 (d, *J* = 6.4 Hz, 4H, phenyl), 6.95 (m, 6H, phenyl), 6.47 (s, 8H, phenyl), 2.23 (s, 30H, CpC*H*_3_), 2.08 (s, 6H, tolylC*H*_3_) ppm. ^13^C­{^1^H} NMR (C_6_D_6_): δ 174.3 (*C*O), 145.2 (phenyl *C*), 136.7 (phenyl *C*), 132.1 (phenyl *C*), 130.4 (phenyl *C*), 130.0 (phenyl *C*), 129.1 (phenyl *C*), 128.5 (phenyl *C*), 125.0 (phenyl *C*), 123.7 (ring *C*), 20.9 (tolyl*C*H_3_), 12.1 (Cp*C*H_3_) ppm. IR (KBr, cm^–1^): ν 2921 (s), 1608 (m), 1508 (s), 1465 (s), 1367 (s), 1265 (s), 1098 (s), 1064 (s), 823 (s). Anal. Calcd for C_48_H_54_N_2_O_2_Th: C, 62.46; H, 5.90; N, 3.04. Found: C, 62.44; H, 5.91; N, 3.06.

#### Method B

##### NMR Scale

A C_6_D_6_ (0.3 mL) solution of PhCONH­(*p*-tolyl) (8.2 mg, 0.04 mmol) was slowly added to a J. Young NMR tube charged with (η^5^-C_5_Me_5_)_2_Th=N­(*p*-tolyl)­(dmap)_2_ (**5**; 17.0 mg, 0.02 mmol) and C_6_D_6_ (0.2 mL). Resonances of **30** and those of dmap and *p*-toluidine were observed by ^1^H NMR spectroscopy (100% conversion) when this solution was kept at room temperature overnight.

### Preparation of (η^5^-C_5_Me_5_)_2_Th­[η^3^-NHC­(Ph)­N­(*p*-tolyl)]­[κ^2^-*C*,*N*-4-(Me_2_N)­C_5_H_3_N] (**31b**)

#### Method A

This compound was prepared as colorless microcrystals from the reaction of (η^5^-C_5_Me_5_)_2_Th=N­(*p*-tolyl)­(dmap)_2_ (**5**; 213 mg, 0.25 mmol) and PhCN (26 mg, 0.25 mmol) in toluene (15 mL) at 80 °C and recrystallization from a benzene solution by a similar procedure as that in the synthesis of **6**. The product was isolated by filtration, rapidly washed with cooled *n*-hexane (2 mL), and dried at room temperature under vacuum overnight. Yield: 171 mg (82%). M.p.: 158–160 °C (dec.). ^1^H NMR (C_6_D_6_): δ 8.03 (d, *J* = 4.8 Hz, 1H, py), 7.47 (d, *J* = 7.0 Hz, 2H, phenyl), 6.97 (m, 7H, phenyl), 6.59 (s, 1H, py), 6.09 (s, 1H, py), 6.03 (s, 1H, N*H*), 2.38 (s, 6H, N­(C*H*_3_)_2_), 2.16 (s, 3H, tolylC*H*_3_), 2.03 (s, 30H, CpC*H*_3_) ppm. ^13^C­{^1^H} NMR (C_6_D_6_): δ 172.7 (Th*C*), 154.2 (aryl *C*), 150.6 (aryl *C*), 149.3 (aryl *C*), 142.7 (aryl *C*), 139.5 (aryl *C*), 130.3 (aryl *C*), 129.9 (aryl *C*), 128.9 (aryl *C*), 126.5 (aryl *C*), 123.9 (aryl *C*), 120.8 (ring *C*), 111.5 (aryl *C*), 106.8 (aryl *C*), 106.6 (*C*=N), 38.6 (N*C*H_3_), 38.3 (N*C*H_3_), 20.8 (tolyl*C*H_3_), 11.8 (Cp*C*H_3_), 11.7 (Cp*C*H_3_) ppm. IR (KBr, cm^–1^): ν 3395 (m), 2963 (s), 1604 (s), 1523 (s), 1447 (s), 1369 (s), 1260 (s), 1098 (s), 1018 (s), 800 (s). Anal. Calcd for C_41_H_52_N_4_Th: C, 59.12; H, 6.29; N, 6.73. Found: C, 59.14; H, 6.31; N, 6.71. While colorless crystals of (η^5^-C_5_Me_5_)_2_Th­[N­(*p*-tolyl)­C­(Ph)=N]­(dmap)·3C_6_H_6_ (**31a**·3C_6_H_6_) and **31b** suitable for X-ray structural analysis were isolated from a mixture of benzene and *n*-hexane (4:1) solution, NMR spectroscopy only showed the presence of the resonances of **31b** in C_6_D_6_ solution, indicating an equilibrium between **31a** and **31b** may exist in the solution.

#### Method B

##### NMR Scale

A C_6_D_6_ (0.3 mL) solution of PhCN (2.1 mg, 0.02 mmol) was slowly added to a J. Young NMR tube charged with (η^5^-C_5_Me_5_)_2_Th=N­(*p*-tolyl)­(dmap)_2_ (**5**; 17.0 mg, 0.02 mmol) and C_6_D_6_ (0.2 mL). Resonances of **31b** and those of dmap were observed by ^1^H NMR spectroscopy (100% conversion) when this solution was kept at 80 °C overnight.

### Preparation of (η^5^-C_5_Me_5_)_2_Th­[η^7^-N­(*p*-tolyl)­C­(Ph)­NC­(Ph)­NC­(Ph)­N]·C_6_H_6_ (32·C_6_H_6_)

#### Method A

This compound was prepared as red crystals from the reaction of (η^5^-C_5_Me_5_)_2_Th=N­(*p*-tolyl)­(dmap)_2_ (**5**; 213 mg, 0.25 mmol) and PhCN (78 mg, 0.75 mmol) in toluene (15 mL) at 80 °C and recrystallization from a benzene solution by a similar procedure as that in the synthesis of **6**. The product was isolated by filtration, rapidly washed with cooled *n*-hexane (2 mL), and dried at room temperature under vacuum overnight. Yield: 209 mg (84%). M.p.: 120–122 °C (dec.). ^1^H NMR (C_6_D_6_): δ 8.94 (d, *J* = 7.2 Hz, 2H, phenyl), 7.60 (d, *J* = 7.3 Hz, 2H, phenyl), 7.51 (t, *J* = 7.6 Hz, 2H, phenyl), 7.32 (t, *J* = 7.3 Hz, 1H, phenyl), 7.15 (s, 6H, C_6_ *H*_6_), 6.83 (m, 6H, phenyl), 6.79 (m, 3H, phenyl), 6.63 (m, 3H, phenyl), 2.12 (s, 30H, CpC*H*_3_), 2.03 (s, 3H, tolylC*H*_3_) ppm. ^13^C­{^1^H} NMR (C_6_D_6_): δ 174.5 (ThN=*C*), 158.5 (phenyl *C*), 156.3 (phenyl *C*), 150.6 (phenyl *C*), 143.3 (phenyl *C*), 141.8 (phenyl *C*), 141.6 (phenyl *C*), 134.9 (phenyl *C*), 133.7 (phenyl *C*), 129.87 (phenyl *C*), 129.86 (phenyl *C*), 129.82 (phenyl *C*), 129.4 (phenyl *C*), 129.3 (phenyl *C*), 129.2 (phenyl *C*), 128.5 (*C*_6_H_6_), 128.1 (phenyl *C*), 127.9 (phenyl *C*), 127.3 (*C*=N), 125.7 (*C*=N), 123.9 (ring *C*), 20.7 (tolyl*C*H_3_), 11.7 (Cp*C*H_3_) ppm. IR (KBr, cm^–1^): ν 2915 (m), 1589 (m), 1521 (s), 1446 (m), 1367 (s), 1027 (m), 743 (s). Anal. Calcd for C_54_H_58_N_4_Th: C, 65.18; H, 5.87; N, 5.63. Found: C, 65.15; H, 5.89; N, 5.61.

#### Method B

##### NMR Scale

A C_6_D_6_ (0.3 mL) solution of PhCN (6.2 mg, 0.06 mmol) was slowly added to a J. Young NMR tube charged with (η^5^-C_5_Me_5_)_2_Th=N­(*p*-tolyl)­(dmap)_2_ (**5**; 17.0 mg, 0.02 mmol) and C_6_D_6_ (0.2 mL). Resonances of **32** and those of dmap were observed by ^1^H NMR spectroscopy (100% conversion) when this solution was kept at 80 °C overnight.

### Preparation of (η^5^-C_5_Me_5_)_2_Th­[η^3^-N­(*p*-tolyl)­C­(CH_2_Ph)­NH]­(N=C=CHPh) (**33**)

#### Method A

This compound was prepared as yellow crystals from the reaction of (η^5^-C_5_Me_5_)_2_Th=N­(*p*-tolyl)­(dmap)_2_ (**5**; 213 mg, 0.25 mmol) and PhCH_2_CN (59 mg, 0.50 mmol) in toluene (15 mL) at room temperature and recrystallization from a benzene solution by a similar procedure as that in the synthesis of **6**. The product was isolated by filtration, rapidly washed with cooled *n*-hexane (2 mL), and dried at room temperature under vacuum overnight. Yield: 173 mg (82%). M.p.: 96–98 °C (dec.). ^1^H NMR (C_6_D_6_): δ 7.23 (t, *J* = 7.4 Hz, 1H, phenyl), 7.12 (d, *J* = 7.2 Hz, 2H, phenyl), 7.04 (m, 7H, phenyl), 6.95 (d, *J* = 7.8 Hz, 2H, phenyl), 6.89 (d, *J* = 7.0 Hz, 2H, phenyl), 5.41 (s, 1H, C=C*H*), 3.46 (s, 1H, N*H*), 3.40 (s, 2H, C*H*_2_), 2.15 (s, 3H, tolylC*H*_3_), 1.92 (s, 30H, CpC*H*_3_) ppm. ^13^C­{^1^H} NMR (C_6_D_6_): δ 175.1­(*C*=NH), 162.9 (*C*=N), 144.1 (phenyl *C*), 142.9 (phenyl *C*), 135.0 (phenyl *C*), 133.5 (phenyl *C*), 130.3 (phenyl *C*), 129.9 (phenyl *C*), 129.1 (phenyl *C*), 128.6 (phenyl *C*), 127.6 (phenyl *C*), 125.2 (phenyl *C*), 124.1 (ring *C*), 122.4 (phenyl *C*), 122.3 (phenyl *C*), 118.4 (H*C*=C), 41.2 (*C*H_2_), 20.9 (tolyl*C*H_3_), 11.2 (Cp*C*H_3_) ppm. IR (KBr, cm^–1^): ν 3383 (s, NH), 2908 (s), 2860 (s), 2049 (s, C=C=N), 1591 (s), 1506 (s), 1296 (s), 1268 (s), 989 (s), 807(s). Anal. Calcd for C_43_H_51_N_3_Th: C, 61.34; H, 6.11; N, 4.99. Found: C, 61.35; H, 6.09; N, 4.97.

#### Method B

##### NMR Scale

A C_6_D_6_ (0.3 mL) solution of PhCH_2_CN (4.7 mg, 0.04 mmol) was slowly added to a J. Young NMR tube charged with (η^5^-C_5_Me_5_)_2_Th=N­(*p*-tolyl)­(dmap)_2_ (**5**; 17.0 mg, 0.02 mmol) and C_6_D_6_ (0.2 mL). Resonances of **33** and those of dmap were observed by ^1^H NMR spectroscopy (100% conversion) when this solution was kept at room temperature overnight.

### Preparation of (η^5^-C_5_Me_5_)_2_Th­[η^3^-N­(*p*-tolyl)­C­(CHPh_2_)­NH]­(N=C=CPh_2_) (**34**)

#### Method A

This compound was prepared as yellow crystals from the reaction of (η^5^-C_5_Me_5_)_2_Th=N­(*p*-tolyl)­(dmap)_2_ (**5**; 213 mg, 0.25 mmol) and Ph_2_CHCN (97 mg, 0.50 mmol) in toluene (15 mL) at room temperature and recrystallization from a benzene solution by a similar procedure as that in the synthesis of **6**. The product was isolated by filtration, rapidly washed with cooled *n*-hexane (2 mL), and dried at room temperature under vacuum overnight. Yield: 214 mg (86%). M.p.: 180–182 °C (dec.). ^1^H NMR (C_6_D_6_): δ 7.36 (d, *J* = 7.9 Hz, 4H, phenyl), 7.16 (d, *J* = 8.0 Hz, 4H, phenyl), 7.07 (m, 5H, phenyl), 7.03 (t, *J* = 7.2 Hz, 4H, phenyl), 6.94 (m, 5H, phenyl), 6.80 (d, *J* = 7.2 Hz, 2H, phenyl), 5.74 (s, 1H, N*H*), 5.21 (s, 1H, C*H*), 2.13 (s, 3H, tolylC*H*_3_), 1.94 (s, 30H, CpC*H*_3_) ppm. ^13^C­{^1^H} NMR (C_6_D_6_): δ 176.3 (*C*=NH), 161.6 (*C*=N), 150.5 (phenyl *C*), 144.0 (phenyl *C*), 140.7 (phenyl *C*), 139.5 (phenyl *C*), 134.1 (phenyl *C*), 129.7 (phenyl *C*), 129.6 (phenyl *C*), 129.2 (phenyl *C*), 128.9 (phenyl *C*), 127.6 (phenyl *C*), 126.5 (phenyl *C*), 125.8 (phenyl *C*), 124.8 (ring *C*), 121.6 (Ph_2_ *C*=C), 42.5 (*C*H), 20.9 (tolyl*C*H_3_), 11.5 (Cp*C*H_3_) ppm. IR (KBr, cm^–1^): ν 2970 (s), 2939 (s), 2027 (s), 1635 (s), 1566 (s), 1381 (s), 1265 (s), 1018 (s), 802 (s). Anal. Calcd for C_55_H_59_N_3_Th: C, 66.45; H, 5.98; N, 4.23. Found: C, 66.43; H, 6.01; N, 4.25.

#### Method B

##### NMR Scale

A C_6_D_6_ (0.3 mL) solution of Ph_2_CHCN (7.7 mg, 0.04 mmol) was slowly added to a J. Young NMR tube charged with (η^5^-C_5_Me_5_)_2_Th=N­(*p*-tolyl)­(dmap)_2_ (**5**; 17.0 mg, 0.02 mmol) and C_6_D_6_ (0.2 mL). Resonances of **34** and those of dmap were observed by ^1^H NMR spectroscopy (100% conversion) when this solution was kept at room temperature overnight.

### Preparation of (η^5^-C_5_Me_5_)_2_Th­[N­(*p*-tolyl)=CHN­(2,6-Me_2_C_6_H_3_)]­[κ^2^-*C*,*N*-4-(Me_2_N)­C_5_H_3_N]·0.5C_6_H_6_ (35·0.5C_6_H_6_)

#### Method A

This compound was prepared as yellow crystals from the reaction of (η^5^-C_5_Me_5_)_2_Th=N­(*p*-tolyl)­(dmap)_2_ (**5**; 213 mg, 0.25 mmol) and 2,6-Me_2_C_6_H_3_NC (33 mg, 0.25 mmol) in toluene (15 mL) at 40 °C and recrystallization from a benzene solution by a similar procedure as that in the synthesis of **6**. The product was isolated by filtration, rapidly washed with cooled *n*-hexane (2 mL), and dried at room temperature under vacuum overnight. Yield: 185 mg (82%). M.p.: 228–230 °C (dec.). ^1^H NMR (C_6_D_6_): δ 8.73 (s, 1H, C*H*=N), 7.15 (s, 3H, C_6_ *H*_6_), 7.12–6.98 (m, 7H, phenyl and py), 6.91 (d, *J* = 8.0 Hz, 1H, phenyl), 6.53 (d, *J* = 8.0 Hz, 1H, py), 5.94 (d, *J* = 3.8 Hz, 1H, py), 2.35 (s, 6H, N­(C*H*_3_)_2_), 2.28 (s, 3H, tolylC*H*_3_), 2.17 (s, 6H, arylC*H*_3_), 2.07 (s, 30H, CpC*H*_3_) ppm. ^13^C­{^1^H} NMR (C_6_D_6_): δ 239.3 (Th*C*), 166.5 (*C*H=N), 162.9 (aryl *C*), 154.2 (aryl *C*), 150.6 (aryl *C*), 150.3 (aryl *C*), 143.3 (aryl *C*), 132.6 (aryl *C*), 131.1 (aryl *C*), 129.8 (aryl *C*), 129.3 (aryl *C*), 128.5 (*C*_6_H_6_), 125.6 (aryl *C*), 123.0 (ring *C*), 117.7 (aryl *C*), 107.4 (aryl *C*), 38.6 (N*C*H_3_), 20.9 (tolyl*C*H_3_), 18.5 (aryl*C*H_3_), 12.4 (Cp*C*H_3_) ppm. IR (KBr, cm^–1^): ν 2913 (s), 2855 (s), 1672 (s), 1600 (s), 1536 (s), 1506 (s), 1433 (s), 1377 (s), 1305 (s), 1193 (s), 993 (s), 806 (s). Anal. Calcd for C_46_H_59_N_4_Th: C, 61.39; H, 6.61; N, 6.23. Found: C, 61.41; H, 6.59; N, 6.25.

#### Method B

##### NMR Scale

A C_6_D_6_ (0.3 mL) solution of 2,6-Me_2_C_6_H_3_NC (2.6 mg, 0.02 mmol) was slowly added to a J. Young NMR tube charged with (η^5^-C_5_Me_5_)_2_Th=N­(*p*-tolyl)­(dmap)_2_ (**5**; 17.0 mg, 0.02 mmol) and C_6_D_6_ (0.2 mL). Resonances of **35** and those of dmap were observed by ^1^H NMR spectroscopy (100% conversion) when this solution was kept at 40 °C overnight.

### Preparation of (η^5^-C_5_Me_5_)_2_Th­[NH­(*p*-tolyl)]­[κ^2^-*C*,*N*-4-(Me_2_N)-6-(2,6-Me_2_C_6_H_3_N=CH)­C_5_H_2_N] (**36**)

#### Method A

This compound was prepared as yellow crystals from the reaction of (η^5^-C_5_Me_5_)_2_Th=N­(*p*-tolyl)­(dmap)_2_ (**5**; 213 mg, 0.25 mmol) and 2,6-Me_2_C_6_H_3_NC (33 mg, 0.25 mmol) in toluene (15 mL) at 15 °C and recrystallization from a benzene solution by a similar procedure as that in the synthesis of **6**. The product was isolated by filtration, rapidly washed with cooled *n*-hexane (2 mL), and dried at room temperature under vacuum overnight. Yield: 181 mg (84%). M.p.: 214–216 °C (dec.). ^1^H NMR (C_6_D_6_): δ 7.91 (s, 1H, C*H*=N), 7.24 (d, *J* = 1.8 Hz, 1H, py), 7.06 (m, 2H, aryl), 6.99 (m, 2H, aryl), 6.91 (d, *J* = 7.8 Hz, 2H, phenyl), 6.53 (d, *J* = 7.8 Hz, 2H, phenyl), 6.48 (s, 1H, N*H*), 2.42 (s, 6H, N­(C*H*_3_)_2_), 2.23 (s, 6H, arylC*H*_3_), 2.22 (s, 3H, tolylC*H*_3_), 2.04 (s, 30H, CpC*H*_3_) ppm. ^13^C­{^1^H} NMR (C_6_D_6_): δ 239.3 (Th*C*), 162.9 (*C*H=N), 154.9 (aryl *C*), 154.6 (aryl *C*), 151.2 (aryl *C*), 150.9 (aryl *C*), 129.3 (aryl *C*), 129.2 (aryl *C*), 127.0 (aryl *C*), 125.6 (aryl *C*), 124.3 (aryl *C*), 122.6 (ring *C*), 117.7 (aryl *C*), 112.0 (aryl *C*), 110.3 (aryl *C*), 38.7 (N*C*H_3_), 20.8 (tolyl*C*H_3_), 18.5 (aryl*C*H_3_), 11.5 (Cp*C*H_3_) ppm. IR (KBr, cm^–1^): ν 3219 (m), 1918 (s), 1647 (s), 1599 (s), 1541 (s), 1507 (s), 1432 (s), 1382 (s), 1262 (s), 1089 (s), 1007 (s), 811 (s). Anal. Calcd for C_43_H_56_N_4_Th: C, 59.99; H, 6.56; N, 6.51. Found: C, 60.01; H, 6.54; N, 6.53.

#### Method B

##### NMR Scale

A C_6_D_6_ (0.3 mL) solution of 2,6-Me_2_C_6_H_3_NC (2.6 mg, 0.02 mmol) was slowly added to a J. Young NMR tube charged with (η^5^-C_5_Me_5_)_2_Th=N­(*p*-tolyl)­(dmap)_2_ (**5**; 17.0 mg, 0.02 mmol) and C_6_D_6_ (0.2 mL). Resonances of **36** and those of dmap were observed by ^1^H NMR spectroscopy (100% conversion) when this solution was kept at 15 °C for 1 week.

### Preparation of (η^5^-C_5_Me_5_)_2_Th­[NH­(*p*-tolyl)]­[κ^2^-*C*,*N*-2-(Me_3_CNC)-4-(Me_2_N)­C_5_H_3_N] (**37**)

#### Method A

This compound was prepared as colorless crystals from the reaction of (η^5^-C_5_Me_5_)_2_Th=N­(*p*-tolyl)­(dmap)_2_ (**5**; 213 mg, 0.25 mmol) and Me_3_CNC (21 mg, 0.25 mmol) in toluene (15 mL) at 50 °C and recrystallization from a benzene solution by a similar procedure as that in the synthesis of **6**. The product was isolated by filtration, rapidly washed with cooled *n*-hexane (2 mL), and dried at room temperature under vacuum overnight. Yield: 167 mg (82%). M.p.: 160–162 °C (dec.). ^1^H NMR (C_6_D_6_): δ 8.38 (d, *J* = 5.9 Hz, 1H, py), 7.16 (d, *J* = 8.2 Hz, 2H, phenyl), 6.85 (d, *J* = 8.2 Hz, 2H, phenyl), 6.51 (d, *J* = 2.5 Hz, 1H, py), 5.93 (m, 1H, py), 5.37 (s, 1H, N*H*), 2.42 (s, 6H, N­(C*H*_3_)_2_), 2.36 (s, 3H, tolylC*H*_3_), 2.16 (s, 30H, CpC*H*_3_), 1.47 (s, 9H, C­(C*H*_3_)_3_) ppm. ^13^C­{^1^H} NMR (C_6_D_6_): δ 271.5 (Th*C*), 166.1 (py *C*), 153.6 (py *C*), 150.6 (py *C*), 149.3 (phenyl *C*), 129.4 (phenyl *C*), 123.4 (ring *C*), 118.3 (phenyl *C*), 106.8 (phenyl *C*), 104.2 (py *C*), 102.8 (py *C*), 64.4 (*C*(CH_3_)), 38.5 (N*C*H_3_), 31.8 (C­(*C*H_3_)_3_), 20.9 (tolyl*C*H_3_), 11.8 (Cp*C*H_3_) ppm. IR (KBr, cm^–1^): ν 2962 (s), 2908 (s), 2862 (s), 1604 (s), 1512 (s), 1442 (s), 1373 (s), 1226 (s), 1003 (s), 810 (s). Anal. Calcd for C_39_H_56_N_4_Th: C, 57.62; H, 6.94; N, 6.89. Found: C, 57.61; H, 6.96; N, 6.91.

#### Method B

##### NMR Scale

A C_6_D_6_ (0.3 mL) solution of Me_3_CNC (1.7 mg, 0.02 mmol) was slowly added to a J. Young NMR tube charged with (η^5^-C_5_Me_5_)_2_Th=N­(*p*-tolyl)­(dmap)_2_ (**5**; 17.0 mg, 0.02 mmol) and C_6_D_6_ (0.2 mL). Resonances of **37** and those of dmap were observed by ^1^H NMR spectroscopy (100% conversion) when this solution was kept at 50 °C overnight.

### Preparation of (η^5^-C_5_Me_5_)_2_Th­[NH­(*p*-tolyl)]­[κ^2^-*C*,*N*-2-(C_6_H_11_NC)-4-(Me_2_N)­C_5_H_3_N] (**38**)

#### Method A

This compound was prepared as colorless crystals from the reaction of (η^5^-C_5_Me_5_)_2_Th=N­(*p*-tolyl)­(dmap)_2_ (**5**; 213 mg, 0.25 mmol) and C_6_H_11_NC (28 mg, 0.25 mmol) in toluene (15 mL) at 15 °C and recrystallization from a benzene solution by a similar procedure as that in the synthesis of **6**. The product was isolated by filtration, rapidly washed with cooled *n*-hexane (2 mL), and dried at room temperature under vacuum overnight. Yield: 164 mg (78%). M.p.: 178–180 °C (dec.). ^1^H NMR (C_6_D_6_): δ 7.19 (d, *J* = 8.1 Hz, 2H, phenyl), 7.154 (s, 1H, py), 6.91 (d, *J* = 8.1 Hz, 2H, phenyl), 6.70 (d, *J* = 2.6 Hz, 1H, py), 5.98 (m, 1H, py), 5.85 (s, 1H, N*H*), 4.60 (m, 1H, C*H*), 2.43 (s, 6H, N­(C*H*_3_)_2_), 2,38 (s, 3H, tolylC*H*_3_), 2.18 (s, 30H, CpC*H*_3_), 1.96 (m, 2H, C*H*_2_), 1.67 (m, 2H, C*H*_2_), 1.50 (m, 2H, C*H*_2_), 1.26 (m, 2H, C*H*_2_), 1.13 (m, 2H, C*H*_2_) ppm. ^13^C­{^1^H} NMR (C_6_D_6_): δ 269.9 (Th*C*), 162.1 (py *C*), 154.2 (py *C*), 154.1 (py *C*), 150.6 (phenyl *C*), 129.5 (phenyl *C*), 128.3 (phenyl *C*), 123.4 (ring *C*), 118.2 (phenyl *C*), 106.2 (py *C*), 105.0 (py *C*), 63.7 (Cy *C*), 38.5 (N*C*H_3_), 35.1 (Cy *C*), 26.1 (Cy *C*), 25.6 (Cy *C*), 20.9 (tolyl*C*H_3_), 11.9 (Cp*C*H_3_) ppm. IR (KBr, cm^–1^): ν 2928 (s), 1599 (s), 1539 (s), 1518 (s), 1446 (s), 1381 (s), 1226 (s), 987 (s), 804 (s). Anal. Calcd for C_41_H_58_N_4_Th: C, 58.70; H, 6.97; N, 6.68. Found: C, 58.71; H, 6.94; N, 6.71.

#### Method B

##### NMR Scale

A C_6_D_6_ (0.3 mL) solution of C_6_H_11_NC (2.2 mg, 0.02 mmol) was slowly added to a J. Young NMR tube charged with (η^5^-C_5_Me_5_)_2_Th=N­(*p*-tolyl)­(dmap)_2_ (**5**; 17.0 mg, 0.02 mmol) and C_6_D_6_ (0.2 mL). Resonances of **38** and those of dmap were observed by ^1^H NMR spectroscopy (100% conversion) when this solution was kept at 15 °C for 1 week.

### Preparation of (η^5^-C_5_Me_5_)_2_Th­[N­(*p*-tolyl)­C­(=CHN=CHPh)­N­(CH_2_Ph)]­(dmap) (**39**)

A benzene (5 mL) solution of PhCH_2_NC (59 mg, 0.50 mmol) was added to a benzene (10 mL) solution of (η^5^-C_5_Me_5_)_2_Th=N­(*p*-tolyl)­(dmap)_2_ (**5**; 213 mg, 0.25 mmol) without stirring at room temperature. After the solution was stored at 40 °C overnight without stirring, orange crystals were isolated from the solution by filtration. The product was washed with *n*-hexane (2 mL) and dried at room temperature under vacuum overnight, and the product was identified as **39** by X-ray diffraction analysis. Yield: 202 mg (84%). M.p.: 120–122 °C (dec.). IR (KBr, cm^–1^): ν 2908 (s), 2862 (s), 1597 (s), 1496 (s), 1427 (s), 1288 (s), 810(s). Anal. Calcd for C_50_H_61_N_5_Th: C, 62.29; H, 6.38; N, 7.26. Found: C, 62.27; H, 6.35; N, 7.28. This compound was insoluble in common (deuterated) solvents such as pyridine, THF, toluene, and CD_2_Cl_2_, which prevented its characterization by NMR spectroscopy.

### Preparation of (η^5^-C_5_Me_5_)_2_Th­[N­(*p*-tolyl)­N=NN­(*p*-tolyl)] (**40**)

#### Method A

This compound was prepared as orange microcrystals from the reaction of (η^5^-C_5_Me_5_)_2_Th=N­(*p*-tolyl)­(dmap)_2_ (**5**; 213 mg, 0.25 mmol) and *p*-tolylN_3_ (34 mg, 0.25 mmol) in toluene (15 mL) at 40 °C and recrystallization from a benzene solution by a similar procedure as that in the synthesis of **6**. The product was isolated by filtration, rapidly washed with cooled *n*-hexane (2 mL), and dried at room temperature under vacuum overnight. Yield: 152 mg (82%). ^1^H NMR (C_6_D_6_): δ 7.12 (d, *J* = 7.2 Hz, 4H, phenyl), 7.04 (d, *J* = 7.2 Hz, 4H, phenyl), 2.25 (s, 6H, tolylC*H*_3_), 1.88 (s, 30H, CpC*H*_3_) ppm. These spectroscopic data agreed with those reported in the literature.^4f,7g^

#### Method B

##### NMR Scale

A C_6_D_6_ (0.3 mL) solution of *p*-tolylN_3_ (2.7 mg, 0.02 mmol) was slowly added to a J. Young NMR tube charged with (η^5^-C_5_Me_5_)_2_Th=N­(*p*-tolyl)­(dmap)_2_ (**5**; 17.0 mg, 0.02 mmol) and C_6_D_6_ (0.2 mL). Resonances of **40** and those of dmap were observed by ^1^H NMR spectroscopy (100% conversion) when this solution was kept at 40 °C overnight.

### Preparation of (η^5^-C_5_Me_5_)_2_Th­[NH­(*p*-tolyl)]­[κ^2^-*N*,*N*-2-N­(NN-*p*-tolyl)-4-(Me_2_N)­C_5_H_3_N] (**41**)

#### Method A

This compound was prepared as yellow crystals from the reaction of (η^5^-C_5_Me_5_)_2_Th=N­(*p*-tolyl)­(dmap)_2_ (**5**; 213 mg, 0.25 mmol) and *p*-tolylN_3_ (34 mg, 0.25 mmol) in toluene (15 mL) at 15 °C and recrystallization from a benzene solution by a similar procedure as that in the synthesis of **6**. The product was isolated by filtration, rapidly washed with cooled *n*-hexane (2 mL), and dried at room temperature under vacuum overnight. Yield: 168 mg (78%). M.p.: 160–162 °C (dec.). ^1^H NMR (C_6_D_6_): δ 8.00 (d, *J* = 8.2 Hz, 2H, phenyl), 7.69 (d, *J* = 6.6 Hz, 1H, py), 7.26 (d, *J* = 7.9 Hz, 2H, phenyl), 7.15 (d, *J* = 7.9 Hz, 2H, phenyl), 6.81 (d, *J* = 8.2 Hz, 2H, phenyl), 6.74 (d, *J* = 2.5 Hz, 1H, py), 5.36 (dd, *J* = 6.6 and 2.5 Hz, 1H, py), 5.16 (s, 1H, N*H*), 2.38 (s, 3H, tolylC*H*_3_), 2.22 (s, 3H, tolylC*H*_3_), 2.19 (s, 6H, N­(C*H*_3_)_2_), 2.15 (s, 30H, CpC*H*_3_) ppm. ^13^C­{^1^H} NMR (C_6_D_6_): δ 168.1 (py *C*), 157.1 (py *C*), 154.9 (py *C*), 147.0 (phenyl *C*), 135.8 (phenyl *C*), 130.0 (phenyl *C*), 129.5 (phenyl *C*), 128.5 (phenyl *C*), 124.9 (ring *C*), 123.9 (phenyl *C*), 121.4 (phenyl *C*), 118.9 (phenyl *C*), 100.1 (py *C*), 87.1 (py *C*), 38.4 (N*C*H_3_), 21.1 (tolyl*C*H_3_), 20.8 (tolyl*C*H_3_), 11.9 (Cp*C*H_3_) ppm. IR (KBr, cm^–1^): ν 2918 (s), 2856 (s), 1602 (s), 1531 (s), 1452 (s), 1384 (s),1369 (s), 1302 (s), 1278 (s), 1195 (s), 1151 (s), 993 (s), 823 (s). Anal. Calcd for C_41_H_54_N_6_Th: C, 57.06; H, 6.31; N, 9.74. Found: C, 57.04; H, 6.34; N, 9.73.

#### Method B

##### NMR Scale

A C_6_D_6_ (0.3 mL) solution of *p*-tolylN_3_ (2.7 mg, 0.02 mmol) was slowly added to a J. Young NMR tube charged with (η^5^-C_5_Me_5_)_2_Th=N­(*p*-tolyl)­(dmap)_2_ (**5**; 17.0 mg, 0.02 mmol) and C_6_D_6_ (0.2 mL). Resonances of **41** and those of dmap were observed by ^1^H NMR spectroscopy (100% conversion) when this solution was kept at 15 °C for 1 week.

### Preparation of (η^5^-C_5_Me_5_)_2_Th­[NH­(*p*-tolyl)]­[κ^2^-*N*,*N*-2-N­(NNCPh_3_)-4-(Me_2_N)­C_5_H_3_N] (**42**)

#### Method A

This compound was prepared as colorless crystals from the reaction of (η^5^-C_5_Me_5_)_2_Th=N­(*p*-tolyl)­(dmap)_2_ (**5**; 213 mg, 0.25 mmol) and Ph_3_CN_3_ (72 mg, 0.25 mmol) in toluene (15 mL) at 50 °C and recrystallization from a benzene solution by a similar procedure as that in the synthesis of **6**. The product was isolated by filtration, rapidly washed with cooled *n*-hexane (2 mL), and dried at room temperature under vacuum overnight. Yield: 208 mg (82%). M.p.: 152–154 °C (dec.). ^1^H NMR (C_6_D_6_): δ 7.63 (d, *J* = 6.5 Hz, 1H, py), 7.57 (d, *J* = 7.6 Hz, 6H, phenyl), 7.46 (s, 1H, N*H*), 7.18 (m, 6H, phenyl), 7.06 (m, 3H, phenyl), 6.91 (d, *J* = 7.8 Hz, 2H, phenyl), 6.49 (d, *J* = 7.8 Hz, 2H, phenyl), 6.05 (d, *J* = 2.0 Hz, 1H, py), 5.80 (m, 1H, py), 2.29 (s, 6H, N­(C*H*_3_)_2_), 2.27 (s, 3H, tolylC*H*_3_), 2.05 (s, 30H, CpC*H*_3_) ppm. ^13^C­{^1^H} NMR (C_6_D_6_): δ 168.0 (py *C*), 156.3 (py *C*), 153.6 (py *C*), 146.5 (phenyl *C*), 145.0 (phenyl *C*), 131.2 (phenyl *C*), 128.9 (phenyl *C*), 128.5 (phenyl *C*), 126.8 (phenyl *C*), 124.5 (ring *C*), 123.8 (phenyl *C*), 120.2 (phenyl *C*), 100.1 (py *C*), 88.1 (py *C*), 82.0 (N*C*Ph_3_), 38.7 (N*C*H_3_), 20.8 (tolyl*C*H_3_), 11.8 (Cp*C*H_3_) ppm. IR (KBr, cm^–1^): ν 3164 (m), 2906 (s), 2856 (s), 1604 (s), 1503 (s), 1433 (s), 1376 (s), 1266 (s), 1162 (s), 999 (s), 750 (s). Anal. Calcd for C_53_H_62_N_6_Th: C, 62.71; H, 6.16; N, 8.28. Found: C, 62.73; H, 6.14; N, 8.31.

#### Method B

##### NMR Scale

A C_6_D_6_ (0.3 mL) solution of Ph_3_CN_3_ (5.7 mg, 0.02 mmol) was slowly added to a J. Young NMR tube charged with (η^5^-C_5_Me_5_)_2_Th=N­(*p*-tolyl)­(dmap)_2_ (**5**; 17.0 mg, 0.02 mmol) and C_6_D_6_ (0.2 mL). Resonances of **42** and those of dmap were observed by ^1^H NMR spectroscopy (100% conversion) when this solution was kept at 50 °C overnight.

### Preparation of (η^5^-C_5_Me_5_)_2_Th­(N_3_)­[N­(*p*-tolyl)­SiMe_3_] (**43**)

#### Method A

This compound was prepared as colorless crystals from the reaction of (η^5^-C_5_Me_5_)_2_Th=N­(*p*-tolyl)­(dmap)_2_ (**5**; 213 mg, 0.25 mmol) and Me_3_SiN_3_ (29 mg, 0.25 mmol) in toluene (15 mL) at 40 °C and recrystallization from a benzene solution by a similar procedure as that in the synthesis of **6**. The product was isolated by filtration, rapidly washed with cooled *n*-hexane (2 mL), and dried at room temperature under vacuum overnight. Yield: 151 mg (84%). M.p.: 182–184 °C (dec.). ^1^H NMR (C_6_D_6_): δ 7.00 (d, *J* = 7.8 Hz, 2H, phenyl), 6.80 (d, *J* = 8.0 Hz, 2H, phenyl), 2.16 (s, 3H, tolylC*H*_3_), 1.93 (s, 30H, CpC*H*_3_), 0.39 (s, 9H, SiC*H*_3_) ppm. ^13^C­{^1^H} NMR (C_6_D_6_): δ 148.7 (phenyl *C*), 133.2 (phenyl *C*), 129.9 (phenyl *C*), 126.3 (phenyl *C*), 118.9 (ring *C*), 20.5 (tolyl*C*H_3_), 11.2 (Cp*C*H_3_), 3.4 (Si*C*H_3_) ppm. ^29^Si­{^1^H} NMR (C_6_D_6_): δ −1.76 ppm. IR (KBr, cm^–1^): ν 2912 (s), 2092 (s), 1611 (s), 1516 (s), 1441 (s), 1383 (s), 1242 (s), 1080 (s), 919 (s), 833 (s). Anal. Calcd for C_30_H_46_N_4_SiTh: C, 49.85; H, 6.41; N, 7.75. Found: C, 49.83; H, 6.44; N, 7.73.

#### Method B

##### NMR Scale

A C_6_D_6_ (0.3 mL) solution of Me_3_SiN_3_ (2.3 mg, 0.02 mmol) was slowly added to a J. Young NMR tube charged with (η^5^-C_5_Me_5_)_2_Th=N­(*p*-tolyl)­(dmap)_2_ (**5**; 17.0 mg, 0.02 mmol) and C_6_D_6_ (0.2 mL). Resonances of **43** and those of dmap were observed by ^1^H NMR spectroscopy (100% conversion) when this solution was kept at 40 °C overnight.

### Preparation of [(η^5^-C_5_Me_5_)_2_Th]_2_(μ-N=NN=CSiMe_3_)_2_ (**44**)

#### Method A

This compound was prepared as colorless crystals from the reaction of (η^5^-C_5_Me_5_)_2_Th=N­(*p*-tolyl)­(dmap)_2_ (**5**; 213 mg, 0.25 mmol) and Me_3_SiCHN_2_ (29 mg, 0.25 mmol) in toluene (15 mL) at room temperature and recrystallization from a benzene solution by a similar procedure as that in the synthesis of **6**. The product was isolated by filtration, rapidly washed with cooled *n*-hexane (2 mL), and dried at room temperature under vacuum overnight. Yield: 120 mg (76%). ^1^H NMR (C_6_D_6_): δ 2.13 (s, 60H, CpC*H*_3_), 0.52 (s, 18H, SiC*H*_3_) ppm. These spectroscopic data agreed with those reported in the literature.^7g^ Furthermore, this complex was also characterized by X-ray diffraction analysis, and its molecular structure is shown in the Supporting Information (Figure S11).

#### Method B

##### NMR Scale

A C_6_D_6_ (0.3 mL) solution of Me_3_SiCHN_2_ (2.3 mg, 0.02 mmol) was slowly added to a J. Young NMR tube charged with (η^5^-C_5_Me_5_)_2_Th=N­(*p*-tolyl)­(dmap)_2_ (**5**; 17.0 mg, 0.02 mmol) and C_6_D_6_ (0.2 mL). Resonances of **44** and those of dmap and toluene were observed by ^1^H NMR spectroscopy (100% conversion) when this solution was kept at room temperature for 2 days.

### X-ray Crystallography

Single-crystal X-ray diffraction measurements were carried out on a Bruker Smart APEX II CCD diffractometer or on a Rigaku Saturn CCD diffractometer using Mο Kα radiation (λ = 0.71073 Å) or Cu Kα radiation (λ = 1.54184 Å). An empirical absorption correction was applied using the SADABS program.^11^ All structures were solved by direct methods and refined by full-matrix least-squares on *F* ^2^ using the SHELXL program package.^12^ All the hydrogen atoms were geometrically fixed using the riding model. The crystal data and experimental data for **2**–**39** and **41**–**44** are summarized in the Supporting Information. Selected bond lengths and angles are listed in Table 1.

### Computational Methods

Calculations were performed with the Gaussian 09 program (G09),^13^ employing the B3PW91 functional, plus a polarizable continuum model (PCM) (denoted as B3PW91-PCM), with a standard 6–31G­(d) basis set for the elements C, H and N and a quasi-relativistic 5f-in-valence effective-core potential (ECP60MWB) treatment with 60 electrons in the core region for Th and the corresponding optimized segmented ((14s13p10d8f6g)/[10s9p5d4f3g]) basis set for the valence shells of Th,^14^ to fully optimize the structures of reactants, transition state(s), intermediates, and products, and to also account for the experimental reaction conditions using toluene as a solvent (dielectric constant ε = 2.379). All stationary points were subsequently characterized by vibrational analyses, from which their respective zero-point (vibrational) energy (ZPE) was extracted and used in the relative energy determinations. In addition, frequency calculations were also performed to ensure that the determined structures for reactants, intermediates, products, and transition states resided at minima and first-order saddle points, respectively, on their potential energy hypersurfaces.

## Supplementary Material





## Notes

Cartesian coordinates of all stationary points optimized at the B3PW91-PCM level (XYZ)
Selected reactivity of the thorium imido complexes, molecular structures, crystal parameters, computational details, and NMR spectra (PDF)
