# Supplementary material for: Synthesis and Reactivity of Lewis-Base-Supported Terminal Thorium Imido Metallocene, (η5‑C5Me5)2Th=N(p‑tolyl)(dmap)2
Source: Inorg Chem. 2025 Jun 10;64(24):12132–64. doi: 10.1021/acs.inorgchem.5c01481 (PMC12188576; doi:10.1021/acs.inorgchem.5c01481)
Supplement: Supplementary file 2 [file ic5c01481_si_002.pdf]

Supporting Information for

Synthesis and Reactivity of Lewis-Base-Supported Terminal Thorium Imido Metallocene, $(\eta^5\text{-}C_5Me_5)_2Th{=}N(p\text{-tolyl})(dmap)_2$

Yi Heng,[†] Enwei Zhou,[†] Dongwei Wang,[†] Wanjian Ding,[†] Guohua Hou,[†] Guofu Zi,*[,†] and Marc D. Walter*[,‡]

[†]Department of Chemistry, Beijing Normal University, Beijing 100875, China

[‡]Institut für Anorganische und Analytische Chemie, Technische Universität Braunschweig, Hagenring 30, 38106 Braunschweig, Germany

*Corresponding authors. E-mail: gzi@bnu.edu.cn (G.Z.), mwalter@tu-bs.de (M.D.W.)

Table of contents

1. **Figures** **S2**
2. **Crystallographic details** **S8**
3. **Computational details** **S17**
4. **NMR spectra** **S41**
5. **References** **S84**

1. Figures

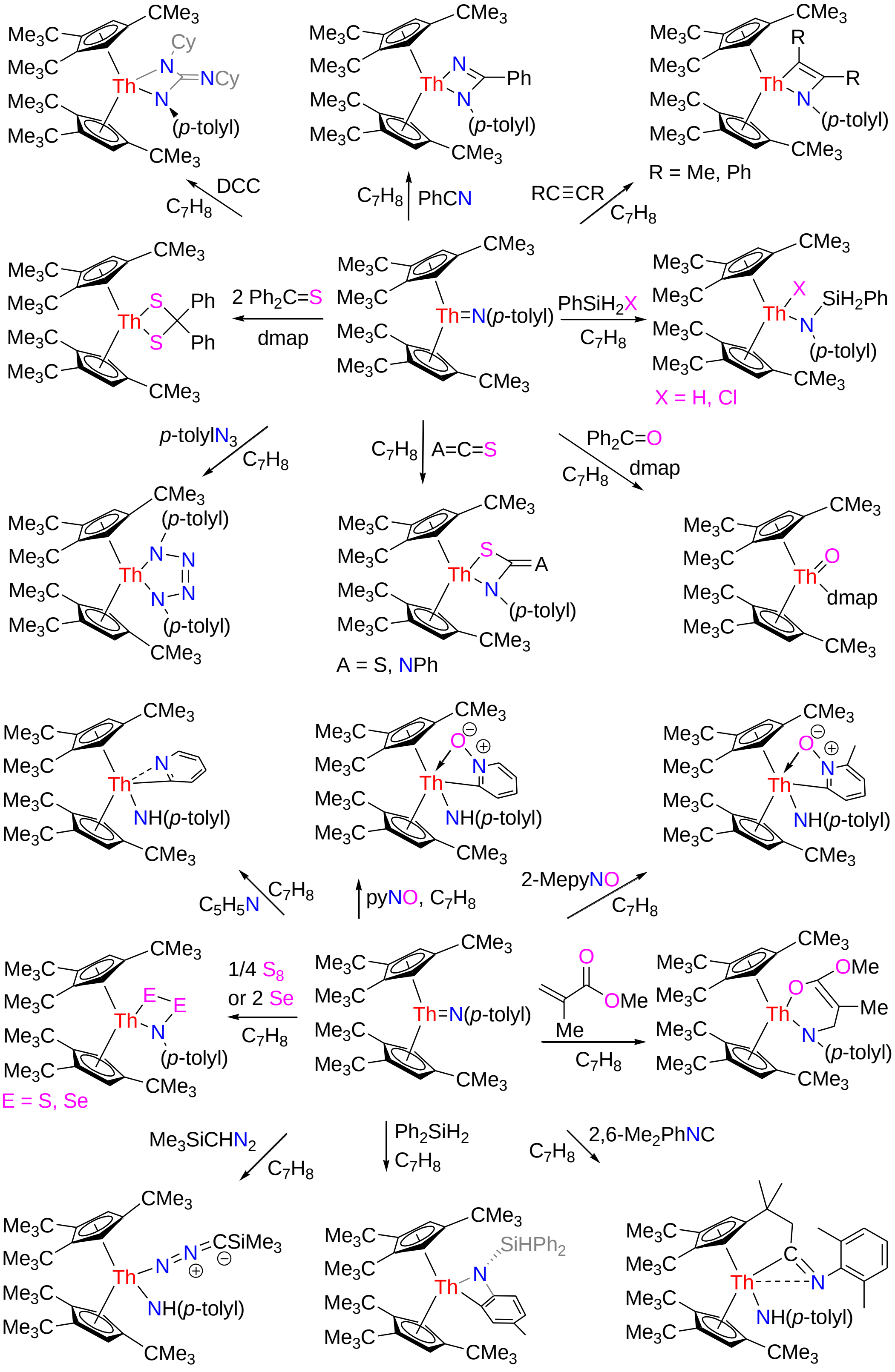

Figure S1. Selected examples concerning the reactivity of [η^5-1,2,4-$(Me_3C)_3C_5H_2]_2Th=N(p$-tolyl).

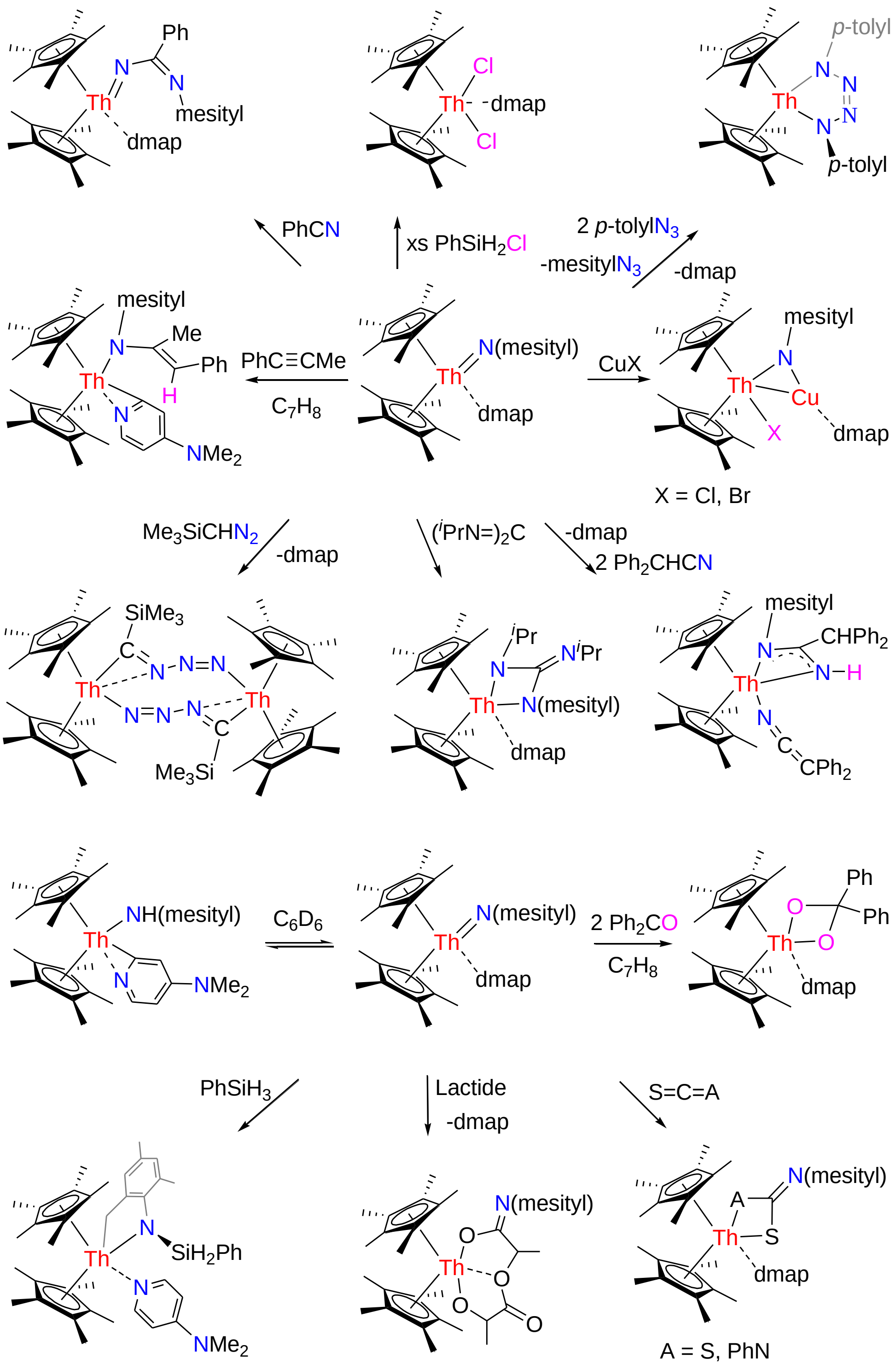

Figure S2. Selected examples concerning the reactivity of (η^5-C_5Me_5)$_2$Th=N(mesityl)(dmap).

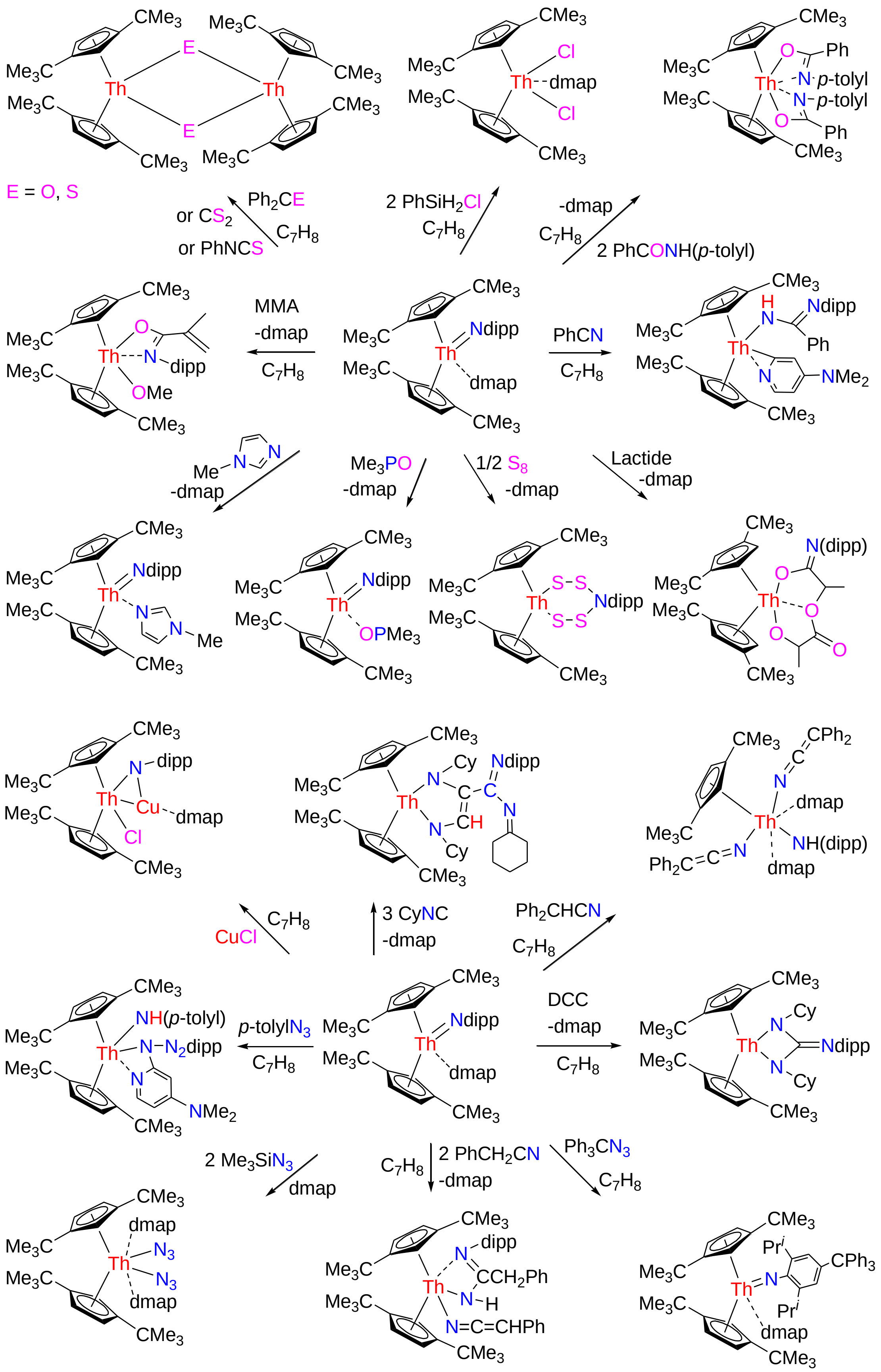

Figure S3. Selected examples concerning the reactivity of [η^5-1,3-(Me$_3$C)$_2$C$_5$H$_3$]$_2$Th=N(dipp)(dmap).

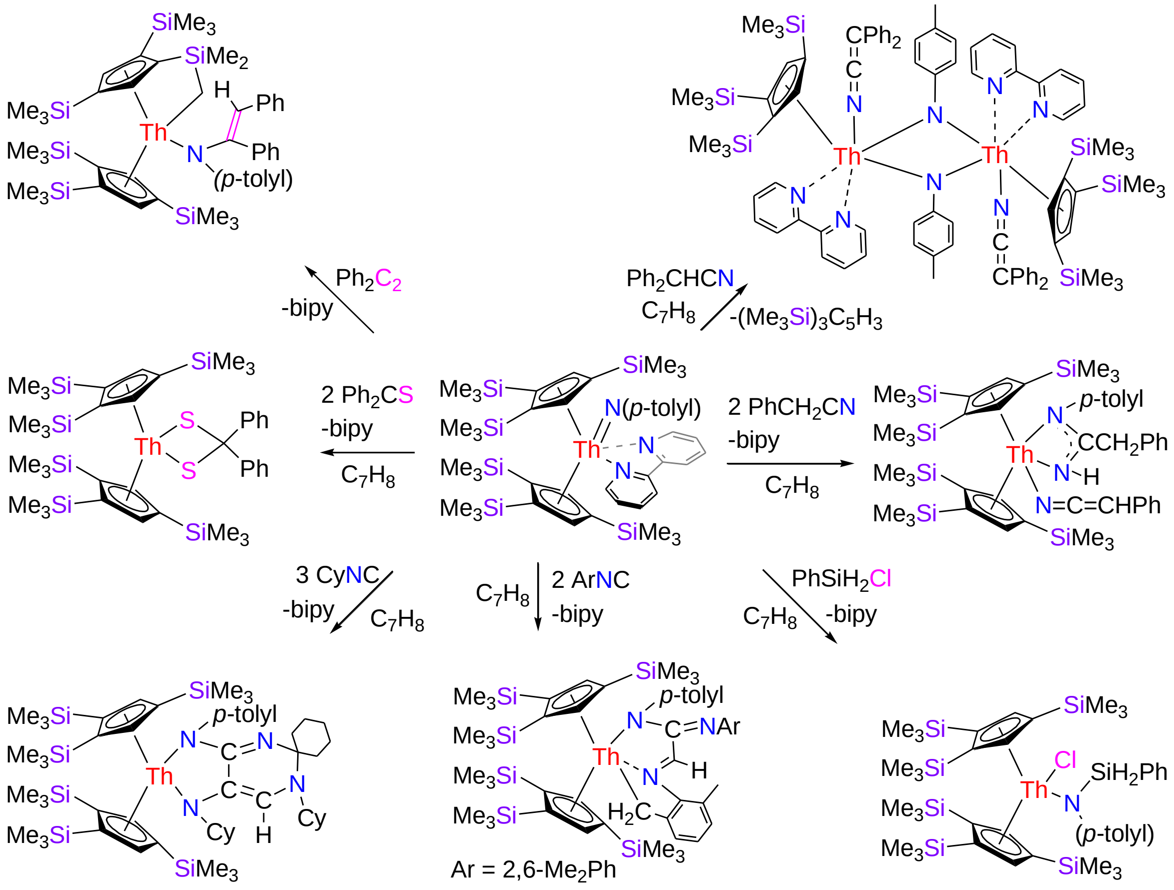

Figure S4. Selected examples concerning the reactivity of [η^5-1,2,4-$(Me_3Si)_3C_5H_2]_2Th{=}N$(*p*-tolyl)(bipy).

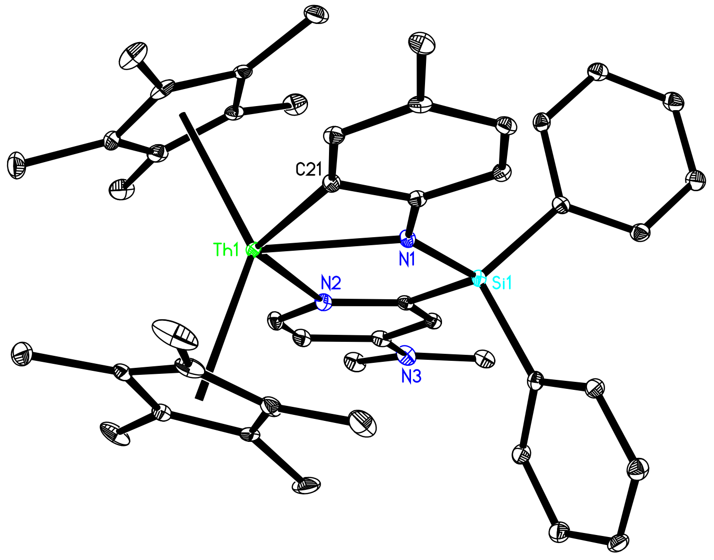

Figure S5. Molecular structure of (η^5-$C_5Me_5)_2Th[\kappa^3$-*C*,*N*,*N*-(4-$Me_2NC_5H_3N)SiPh_2N(4$-$MeC_6H_3)]$ (**19**) (thermal ellipsoids drawn at the 35% probability level).

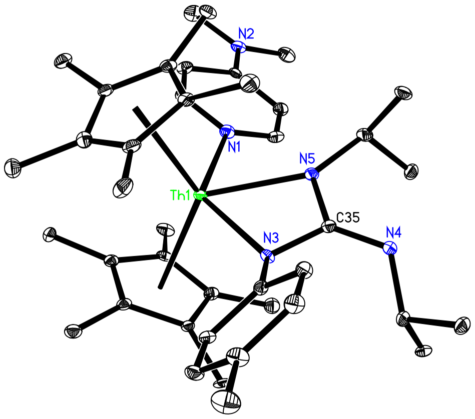

Figure S6. Molecular structure of (η^5-C_5Me_5)$_2$Th[N(*p*-tolyl)C(=N^{i}Pr)N(^{i}Pr)](dmap) (**22**) (thermal ellipsoids drawn at the 35% probability level).

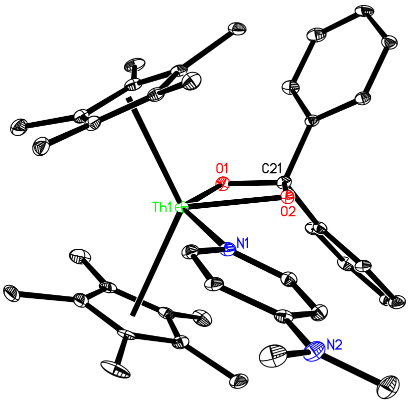

Figure S7. Molecular structure of (η^5-C_5Me_5)$_2$Th[$OCPh_2O$](dmap) (**24**) (thermal ellipsoids drawn at the 35% probability level). Selected bond lengths (Å) and angles (°): Th(1)-C(Cp) (av.) 2.877(12), Th(1)-C(Cp) (range) 2.826(5) to 2.923(5), Th(1)-Cp (cent) 2.609(5) and 2.615(5), Th(1)-N(1) 2.615(4), Th(1)-O(1) 2.248(3), Th(1)-O(2) 2.253(3), Cp(cent)-Th(1)-Cp(cent) 130.7(2), O(1)-Th(1)-O(2) 60.9(1).

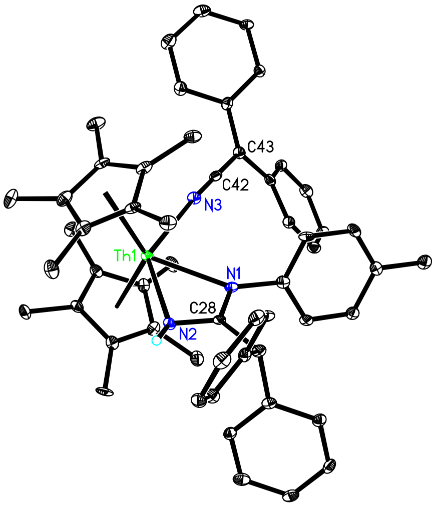

Figure S8. Molecular structure of (η^5-C_5Me_5)$_2$Th[η^3-N(*p*-tolyl)C($CHPh_2$)NH](N=C=CPh_2) (**34**) (thermal ellipsoids drawn at the 35% probability level).

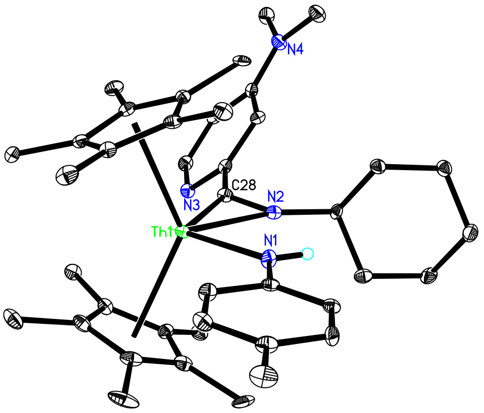

Figure S9. Molecular structure of (η^5-C_5Me_5)$_2$Th[NH(*p*-tolyl)][κ^2-*C*,*N*-2-(C_6H_{11}NC)-4-(Me_2N)C_5H_3N] (**38**) (thermal ellipsoids drawn at the 35% probability level).

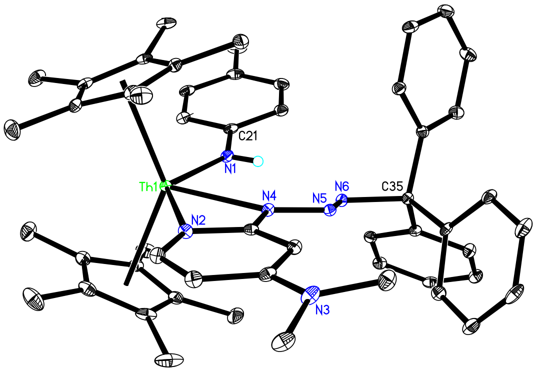

Figure S10. Molecular structure of (η^5-C_5Me_5)$_2$Th[NH(*p*-tolyl)][κ^2-*N*,*N*-2-N(NNCPh$_3$)-4-(Me_2N)C_5H_3N] (**42**) (thermal ellipsoids drawn at the 35% probability level).

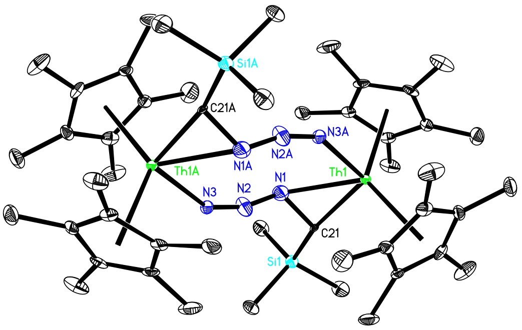

Figure S11. Molecular structure of [(η^5-C_5Me_5)$_2$Th]$_2$(μ-N=NN=CSiMe$_3$)$_2$ (**44**) (thermal ellipsoids drawn at the 35% probability level). Selected bond lengths (Å) and angles (°): Th(1)-C(Cp) (av.) 2.822(6), Th(1)-C(Cp) (range) 2.802(8) to 2.857(9), Th(1)-Cp (cent) 2.550(8) and 2.557(8), Th(1)-N(1) 2.440(9), Th(1)-N(3A) 2.456(7), Th(1)-C(21) 2.291(7), Cp(cent)-Th(1)-Cp(cent) 131.0(3), N(1)-Th(1)-N(3A) 77.8(3), N(1)-Th(1)-C(21) 35.7(3).

2. Crystallographic details

Table S1. Crystal Data and Experimental Parameters for Compounds 2-6

Compound	**2**	**3** C_6H_6	**4**	**5**	**6**
Formula	$C_{34}H_{46}N_2Th$	$C_{60}H_{80}N_2Th_2$	$C_{32}H_{42}N_2Th$	$C_{41}H_{57}N_5Th$	$C_{31}H_{43}N_3Th$
Fw	714.77	1293.34	686.71	851.95	689.72
crystal system	orthorhombic,	monoclinic	monoclinic	triclinic	monoclinic
space group	*Pbcn*	*C*2/*c*	$P2_1/c$	$P\bar{1}$	$P2_1/c$
a (Å)	10.111(1)	17.880(1)	14.443(12)	9.467(1)	10.748(12)
b (Å)	16.573(1)	11.276(1)	13.623(11)	12.143(1)	15.206(16)
c (Å)	18.216(1)	25.970(1)	14.486(12)	17.336(1)	18.54(2)
α (deg)	90	90	90	96.23(1)	90
β (deg)	90	95.06(1)	91.80(1)	105.67(1)	92.04(2)
γ (deg)	90	90	90	93.44(1)	90
V (Å^3)	3052.47(12)	5215.7(2)	2845(4)	1899.10(6)	3029(6)
temperature (K)	99.99(10)	100.01(19)	100(2)	100.00(10)	100(2)
Z	4	4	4	2	4
D_{calc} (g/cm^3)	1.555	1.647	1.603	1.490	1.513
μ(Mo/Kα)$_{calc}$ (cm^{-1})	15.913	18.544	5.263	12.910	4.945
size (mm)	0.15 × 0.15 × 0.15	0.10 × 0.05 × 0.05	0.40 × 0.30 × 0.15	0.20 × 0.15 × 0.15	0.20 × 0.20 × 0.20
F(000)	1416	2536	1352	856	1360
2θ range (deg)	9.71 to 143.91	6.83 to 152.83	4.11 to 54.99	7.36 to 153.09	3.46 to 54.69
no. of reflns, collected	7658	18754	18354	24397	19077
no of obsd reflns	2941	5257	6491	7639	6769
no of variables	174	300	327	439	327
abscorr (T_{max}, T_{min})	1.00, 0.22	1.00, 0.61	0.75, 0.43	1.00, 0.31	0.75, 0.43
R	0.028	0.043	0.043	0.030	0.052
R_w	0.075	0.128	0.074	0.077	0.127
R_{all}	0.033	0.045	0.075	0.031	0.067
Gof	1.08	1.07	0.96	1.06	0.99
CCDC	2433970	2433975	2433992	2433984	2433986

Table S2. Crystal Data and Experimental Parameters for Compounds 7-11

Compound	**7**	**8**	**9** $\cdot 0.5C_6H_6 \cdot 0.5C_7H_8$	**10**	**11**
Formula	$C_{34}H_{46}N_2OTh$	$C_{37}H_{53}N_2PTh$	$C_{40.5}H_{54}N_3S_2Th$	$C_{27}H_{37}NS_4Th$	$C_{27}H_{37}NSe_4Th$
Fw	730.77	788.82	879.02	735.85	923.45
crystal system	orthorhombic	monoclinic	triclinic	monoclinic	triclinic
space group	$P2_12_12_1$	$P2_1/n$	$P\bar{1}$	$P2_1$	$P\bar{1}$
a (Å)	10.299(5)	10.404(1)	10.284(2)	10.171(1)	9.334(1)
b (Å)	10.715(6)	17.198(1)	11.432(2)	13.275(1)	11.624(1)
c (Å)	28.044(14)	19.058(1)	16.683(3)	10.371(1)	14.090(1)
α (deg)	90	90	79.14(1)	90	86.67(1)
β (deg)	90	94.01(1)	80.70(1)	94.08(1)	85.86(1)
γ (deg)	90	90	78.75(1)	90	74.66(1)
V (Å^3)	3095(3)	3401.76(13)	1873.3(6)	1396.68(9)	1469.24(12)
temperature (K)	100(2)	100.01(10)	100(2)	100.00(10)	100.00(10)
Z	4	4	2	2	2
D_{calc} (g/cm^3)	1.568	1.540	1.558	1.750	2.087
μ(Mo/Kα)$_{calc}$ (cm^{-1})	4.846	14.767	4.124	5.654	10.045
size (mm)	0.20 × 0.20 × 0.18	0.20 × 0.10 × 0.10	0.25 × 0.20 × 0.20	0.20 × 0.20 × 0.20	0.15 × 0.15 × 0.15
F(000)	1448	1576	880	720	864
2θ range (deg)	4.07 to 54.97	6.93 to 152.23	3.68 to 55.32	7.29 to 59.39	6.97 to 59.43
no. of reflns, collected	20984	24819	12813	7285	14404
no of obsd reflns	7085	6832	8655	5006	6914
no of variables	359	388	479	291	231
abscorr (T_{max}, T_{min})	0.75, 0.62	1.00, 0.40	0.75, 0.63	1.00, 0.83	1.00, 0.58
R	0.036	0.048	0.037	0.049	0.065
R_w	0.066	0.133	0.075	0.098	0.120
R_{all}	0.042	0.052	0.049	0.058	0.096
Gof	0.93	1.10	1.04	1.03	1.00
CCDC	2433987	2434012	2433976	2433971	2433978

Table S3. Crystal Data and Experimental Parameters for Compounds 12-16

Compound	**12**	**13**	**14**	**15**	**16** ·$2C_6H_6$
Formula	$C_{34}H_{47}N_3SeTh$	$C_{34}H_{47}N_3TeTh$	$C_{27}H_{40}N_2F_2Th$	$C_{34}H_{50}Cl_2N_4Th$	$C_{39}H_{52}Br_2N_2Th$
Fw	808.74	857.38	662.65	817.72	940.68
crystal system	orthorhombic	orthorhombic	orthorhombic	monoclinic	triclinic
space group	*Pbca*	*Pbca*	$P2_12_12_1$	$P2_1/n$	$P\bar{1}$
a (Å)	16.941(8)	17.046(10)	10.045(4)	17.919(1)	9.674(1)
b (Å)	18.481(8)	18.638(11)	13.872(5)	9.838(1)	12.865(1)
c (Å)	20.561(10)	20.611(12)	18.745(7)	19.123(1)	16.166(1)
α (deg)	90	90	90	90	67.95(1)
β (deg)	90	90	90	96.63(1)	84.38(1)
γ (deg)	90	90	90	90	86.49(1)
V (Å^3)	6437(5)	6548(7)	2612.0(17)	3348.61(10)	1855.24(5)
temperature (K)	100(2)	100(2)	100(2)	100.00(10)	100.01(10)
Z	8	8	4	4	2
D_{calc} (g/cm^3)	1.669	1.739	1.685	1.622	1.684
μ(Mo/Kα)$_{calc}$ (cm^{-1})	5.790	5.454	5.739	16.038	15.684
size (mm)	0.20 × 0.15 × 0.15	0.20 × 0.20 × 0.20	0.20 × 0.20 × 0.20	0.05 × 0.05 × 0.04	0.15 × 0.15 × 0.15
F(000)	3168	3312	1296	1624	920
2θ range (deg)	3.82 to 55.06	3.79 to 55.41	3.65 to 55.08	7.19 to 152.23	7.42 to 153.22
no. of reflns, collected	41086	42465	17793	25623	22716
no of obsd reflns	7370	7602	5977	6722	7501
no of variables	365	365	302	576	397
abscorr (T_{max}, T_{min})	0.75, 0.47	0.75, 0.50	0.75, 0.58	1.00, 0.72	1.00, 0.68
R	0.034	0.040	0.036	0.032	0.043
R_w	0.064	0.091	0.070	0.077	0.112
R_{all}	0.063	0.067	0.041	0.039	0.044
Gof	1.01	1.02	1.02	1.06	1.06
CCDC	2433997	2434005	2434011	2433990	2433991

Table S4. Crystal Data and Experimental Parameters for Compounds 17-21

Compound	**17**	**18**	**19** C_6H_6	**20**	**21**
Formula	$C_{27}H_{39}N_2ClTh$	$C_{40}H_{51}N_3SiTh$	$C_{52}H_{61}N_3SiTh$	$C_{48}H_{57}N_3Th$	$C_{47}H_{69}N_5Th$
Fw	659.09	833.96	988.16	908.00	936.11
crystal system	triclinic	monoclinic	monoclinic	orthorhombic	monoclinic
space group	$P\bar{1}$	$P2_1/n$	$P2_1/c$	*Pbca*	$P2_1/n$
a (Å)	8.708(8)	11.760(2)	10.501(1)	10.537(1)	9.953(3)
b (Å)	9.759(9)	25.140(3)	26.366(1)	17.357(1)	18.492(6)
c (Å)	15.591(14)	12.903(2)	16.998(1)	44.603(1)	23.387(7)
α (deg)	88.80(1)	90	90	90	90
β (deg)	77.60(1)	101.60(1)	107.91(1)	90	94.20(1)
γ (deg)	79.61(1)	90	90	90	90
V (Å^3)	1273(2)	3736.9(8)	4477.99(9)	8157.8(4)	4293(2)
temperature (K)	100(2)	180(2)	99.9(6)	100.00(10)	100(2)
Z	2	4	4	8	4
D_{calc} (g/cm^3)	1.720	1.482	1.466	1.479	1.448
μ(Mo/Kα)$_{calc}$ (cm^{-1})	5.980	4.053	11.273	12.049	3.511
size (mm)	0.40 × 0.36 × 0.30	0.30 × 0.25 × 0.20	0.15 × 0.10 × 0.10	0.08 × 0.07 × 0.05	0.30 × 0.28 × 0.25
F(000)	644	1664	1992	3648	1904
2θ range (deg)	4.24 to 50.49	3.24 to 50.50	6.70 to 152.55	7.93 to 152.17	3.49 to 55.19
no. of reflns, collected	6566	21242	42571	27498	29102
no of obsd reflns	4364	6760	9119	8031	9885
no of variables	292	505	527	483	491
abscorr (T_{max}, T_{min})	0.75, 0.45	0.75, 0.57	1.00, 0.32	1.00, 0.64	0.75, 0.58
R	0.050	0.034	0.029	0.051	0.039
R_w	0.139	0.065	0.077	0.123	0.073
R_{all}	0.055	0.051	0.030	0.061	0.060
Gof	0.99	1.07	1.07	1.19	0.99
CCDC	2433973	2433989	2433999	2434010	2433993

Table S5. Crystal Data and Experimental Parameters for Compounds 22-26

Compound	**22**	**23**	**24** $\cdot C_6H_6$	**25**	**26**
Formula	$C_{41}H_{61}N_5Th$	$C_{47}H_{57}N_3OTh$	$C_{46}H_{56}N_2O_2Th$	$C_{40}H_{50}N_2S_2Th$	$C_{41}H_{52}N_4STh$
Fw	855.98	911.99	900.96	854.98	864.96
crystal system	monoclinic	monoclinic	monoclinic	monoclinic	monoclinic
space group	$P2_1/n$	$P2_1/n$	$P2_1/c$	$P2_1/c$	$P2_1/c$
a (Å)	11.241(1)	14.628(4)	14.390(1)	26.891(8)	10.470(1)
b (Å)	18.858(1)	17.421(5)	19.601(1)	19.375(6)	20.073(1)
c (Å)	18.081(1)	19.901(6)	15.902(1)	16.476(5)	17.986(1)
α (deg)	90	90	90	90	90
β (deg)	94.25(1)	90.56(1)	115.80(1)	103.37(1)	95.35(1)
γ (deg)	90	90	90	90	90
V (Å^3)	3822.19(8)	5071(2)	4037.82(17)	8352(4)	3763.2(3)
temperature (K)	100.00(10)	100(2)	100.00(10)	100.15	100.00(10)
Z	4	4	4	8	4
D_{calc} (g/cm^3)	1.488	1.195	1.482	1.360	1.527
μ(Mo/Kα)$_{calc}$ (cm^{-1})	12.829	2.971	12.197	3.697	13.540
size (mm)	0.10 × 0.10 × 0.05	0.20 × 0.18 × 0.15	0.10 × 0.10 × 0.10	0.18 × 0.15 × 0.12	0.15 × 0.15 × 0.10
F(000)	1728	1832	1808	3408	1728
2θ range (deg)	6.78 to 152.57	3.64 to 55.04	6.82 to 154.14	3.11 to 55.09	6.61 to 151.66
no. of reflns, collected	28037	33575	30801	56970	22923
no of obsd reflns	7685	11624	8100	19223	7273
no of variables	441	482	472	811	383
abscorr (T_{max}, T_{min})	1.00, 0.66	0.75, 0.64	1.00, 0.48	0.75, 0.62	1.00, 0.55
R	0.063	0.036	0.041	0.039	0.090
R_w	0.170	0.079	0.114	0.089	0.228
R_{all}	0.069	0.051	0.042	0.053	0.121
Gof	1.04	1.02	1.12	0.99	1.10
CCDC	2434008	2434001	2433995	2434003	2433988

Table S6. Crystal Data and Experimental Parameters for Compounds 27-31a

Compound	**27** $\cdot$2C_6H_6	**28**	**29**	**30**	**31a** $\cdot$3C_6H_6
Formula	$C_{68}H_{86}N_2S_4Th_2$	$C_{39}H_{55}N_3O_2Th$	$C_{66}H_{90}N_2O_8Th_2$	$C_{48}H_{54}N_2O_2Th$	$C_{59}H_{70}N_4Th$
Fw	1523.70	829.90	1503.47	922.97	1067.23
crystal system	triclinic	monoclinic	monoclinic	triclinic	orthorhombic
space group	$P\bar{1}$	$P2_1/c$	$C2/c$	$P\bar{1}$	$P2_12_12_1$
a (Å)	9.956(1)	14.952(6)	27.76(2)	10.248(3)	9.699(1)
b (Å)	11.495(1)	13.485(5)	14.648(9)	11.039(3)	19.645(1)
c (Å)	14.623(1)	17.890(7)	17.657(11)	18.780(5)	26.819(1)
α (deg)	104.43(1)	90	90	104.47(1)	90
β (deg)	98.98(1)	91.54(1)	101.40(2)	93.80(1)	90
γ (deg)	106.38(1)	90	90	91.68(1)	90
V (Å^3)	1508.18(13)	3606(2)	7038(8)	2050.3(10)	5110.22(18)
temperature (K)	110.00(12)	100(2)	100(2)	100(2)	100.00(10)
Z	1	4	4	2	4
D_{calc} (g/cm^3)	1.678	1.529	1.419	1.495	1.387
μ(Mo/Kα)$_{calc}$ (cm^{-1})	17.398	4.172	4.269	3.676	9.709
size (mm)	0.20 × 0.10 × 0.10	0.20 × 0.20 × 0.15	0.20 × 0.20 × 0.15	0.30 × 0.28 × 0.25	0.15 × 0.15 × 0.15
F(000)	752	1664	2976	924	2168
2θ range (deg)	8.43 to 143.88	3.78 to 50.49	3.16 to 55.56	3.81 to 54.69	6.59 to 152.11
no. of reflns, collected	10599	15898	23309	13714	21639
no of obsd reflns	5756	6501	8200	9166	9201
no of variables	354	420	365	490	500
abscorr (T_{max}, T_{min})	1.00, 0.47	0.75, 0.59	0.75, 0.47	0.75, 0.59	1.00, 0.61
R	0.033	0.065	0.056	0.029	0.060
R_w	0.078	0.157	0.134	0.064	0.155
R_{all}	0.038	0.095	0.074	0.034	0.065
Gof	1.03	1.00	0.97	1.01	1.03
CCDC	2433974	2433980	2433994	2433972	2434002

Table S7. Crystal Data and Experimental Parameters for Compounds 31b-35

Compound	**31b**	**32** C_6H_6	**33**	**34**	**35** $0.5C_6H_6$
Formula	$C_{41}H_{52}N_4Th$	$C_{54}H_{58}N_4Th$	$C_{43}H_{51}N_3Th$	$C_{55}H_{59}N_3Th$	$C_{46}H_{59}N_4Th$
Fw	832.90	995.08	841.90	994.09	900.01
crystal system	triclinic	triclinic	monoclinic	orthorhombic	monoclinic
space group	$P\bar{1}$	$P\bar{1}$	$P2_1/n$	$P2_12_12_1$	$C2/c$
a (Å)	9.782(1)	10.264(1)	11.144(1)	10.020(1)	16.078(1)
b (Å)	9.831(1)	10.687(1)	28.363(1)	17.464(1)	12.186(1)
c (Å)	20.228(3)	21.778(3)	11.858(1)	25.501(1)	42.237(1)
α (deg)	92.34(1)	101.79(1)	90	90	90
β (deg)	102.15(1)	101.55(1)	90.32(1)	90	102.04(1)
γ (deg)	103.83(1)	95.27(1)	90	90	90
V (Å^3)	1838.1(5)	2269.0(5)	3748.01(6)	4462.3(2)	8092.7(2)
temperature (K)	100(2)	100(2)	100.00(10)	100.01(10)	99.8(4)
Z	2	2	4	4	8
D_{calc} (g/cm^3)	1.505	1.456	1.492	1.480	1.477
μ(Mo/Kα)$_{calc}$ (cm^{-1})	4.089	3.326	13.065	11.071	12.146
size (mm)	0.20 × 0.18 × 0.15	0.20 × 0.18 × 0.15	0.20 × 0.10 × 0.01	0.15 × 0.15 × 0.15	0.20 × 0.15 × 0.15
F(000)	832	1000	1680	2000	3624
2θ range (deg)	4.14 to 55.15	3.92 to 50.50	8.08 to 152.88	6.93 to 153.27	8.56 to 152.85
no. of reflns, collected	12668	13046	52093	33780	26962
no of obsd reflns	8470	8184	7776	8935	8062
no of variables	432	543	439	547	475
abscorr (T_{max}, T_{min})	0.75, 0.56	0.75, 0.66	1.00, 0.72	1.00, 0.23	1.00, 0.52
R	0.043	0.044	0.029	0.053	0.047
R_w	0.074	0.100	0.072	0.130	0.108
R_{all}	0.058	0.055	0.032	0.058	0.047
Gof	1.02	1.01	1.07	1.12	1.23
CCDC	2433985	2434007	2433998	2434004	2434009

Table S8. Crystal Data and Experimental Parameters for Compounds 36-39

Compound	**36**	**37**	**38**	**39**
Formula	$C_{43}H_{56}N_4Th$	$C_{39}H_{56}N_4Th$	$C_{41}H_{58}N_4Th$	$C_{50}H_{61}N_5Th$
Fw	860.95	812.91	838.95	964.07
crystal system	monoclinic	monoclinic	triclinic	monoclinic
space group	$P2_1/c$	$P2_1/n$	$P\bar{1}$	$P2_1$
a (Å)	10.504(3)	15.104(1)	9.855(1)	10.527(1)
b (Å)	17.395(4)	14.405(1)	12.670(1)	38.048(1)
c (Å)	21.057(5)	18.668(1)	15,853(1)	10.763(1)
α (deg)	90	90	104.59(1)	90
β (deg)	90.01(1)	112.44(1)	99.56(1)	99.33(1)
γ (deg)	90	90	95.90(1)	90
V (Å^3)	3847.4(16)	3754.3(3)	1867.18(4)	4253.77(7)
temperature (K)	100(2)	100.01(10)	100.00(10)	100.00(10)
Z	4	4	2	4
D_{calc} (g/cm^3)	1.486	1.438	1.492	1.505
μ(Mo/Kα)$_{calc}$ (cm^{-1})	3.910	4.002	13.112	11.604
size (mm)	0.10 × 0.10 × 0.10	0.20 × 0.20 × 0.20	0.10 × 0.10 × 0.10	0.15 × 0.10 × 0.10
F(000)	1728	1632	844	1944
2θ range (deg)	3.04 to 55.20	7.02 to 59.83	7.29 to 153.08	8.33 to 152.81
no. of reflns, collected	26086	21745	24357	32203
no of obsd reflns	8929	8979	7528	14564
no of variables	452	417	433	901
abscorr (T_{max}, T_{min})	0.75, 0.66	1.00, 0.80	1.00, 0.76	1.00, 0.65
R	0.041	0.041	0.041	0.064
R_w	0.063	0.065	0.090	0.164
R_{all}	0.075	0.057	0.047	0.070
Gof	0.97	1.02	1.07	1.03
CCDC	2434000	2433981	2433983	2433996

Table S9. Crystal Data and Experimental Parameters for Compounds 41-44

Compound	**41**	**42**	**43**	**44**
Formula	$C_{41}H_{54}N_6Th$	$C_{53}H_{62}N_6Th$	$C_{30}H_{46}N_4SiTh$	$C_{48}H_{78}N_6Si_2Th_2$
Fw	862.94	1015.12	722.84	1259.42
crystal system	monoclinic	triclinic	orthorhombic	triclinic
space group	$P2_1/c$	$P\bar{1}$	*Pbca*	$P\bar{1}$
a (Å)	19.758(1)	10.423(1)	18.247(1)	10.650(3)
b (Å)	9.600(1)	11.278(1)	17.708(1)	13.885(3)
c (Å)	24.015(1)	20.642(1)	18.845(1)	18.552(5)
α (deg)	90	80.37(1)	90	96.57(1)
β (deg)	103.87(1)	81.13(1)	90	90.57(1)
γ (deg)	90	77.88(1)	90	106.22(1)
V (Å^3)	4422.41(12)	2320.83(17)	6088.99(12)	2614.3(11)
temperature (K)	100.00(10)	110.01(10)	100.00(10)	100.15
Z	4	2	8	2
D_{calc} (g/cm^3)	1.296	1.453	1.577	1.600
μ(Mo/Kα)$_{calc}$ (cm^{-1})	11.103	10.672	16.343	5.764
size (mm)	0.20 × 0.20 × 0.20	0.20 × 0.20 × 0.20	0.15 × 0.15 × 0.15	0.15 × 0.10 × 0.10
F(000)	1728	1024	2864	1232
2θ range (deg)	7.58 to 144.09	8.10 to 144.39	8.39 to 144.10	3.08 to 61.37
no. of reflns, collected	17803	16672	15970	20591
no of obsd reflns	8498	8870	5848	14235
no of variables	451	557	339	556
abscorr (T_{max}, T_{min})	1.00, 0.74	1.00, 0.41	1.00, 0.70	0.75, 0.66
R	0.034	0.039	0.024	0.059
R_w	0.083	0.098	0.055	0.122
R_{all}	0.037	0.040	0.029	0.108
Gof	1.02	1.09	1.06	0.97
CCDC	2433982	2433979	2433977	2434006

3. Computational Studies

All calculations were carried out with the Gaussian 09 suit (G09),[1] employing the B3PW91 functional in conjunction with a polarizable continuum model (PCM) (denoted as B3PW91-PCM), with standard 6-31G(d) basis set for C, H and N elements, and a quasi-relativistic 5f-in-valence effective-core potential (ECP60MWB) treatment with 60 electrons in the core region for Th and the corresponding optimized segmented ((14s13p10d8f6g)/[10s9p5d4f3g]) basis set for the valence shells of Th,[2-4] to fully optimize the structures of reactants, complexes, transition state, intermediates, and products, and also to mimic the experimental toluene-solvent conditions (dielectric constant ε = 2.379). All stationary points were subsequently verified by vibrational analyses, from which their respective zero-point (vibrational) energy (ZPE) were extracted and used in the relative energy determinations; in addition, frequency calculations were also performed to ensure that the reactant, complex, intermediate, product and transition state structures residing at minima and first-order saddle points, respectively, on their potential energy surfaces.

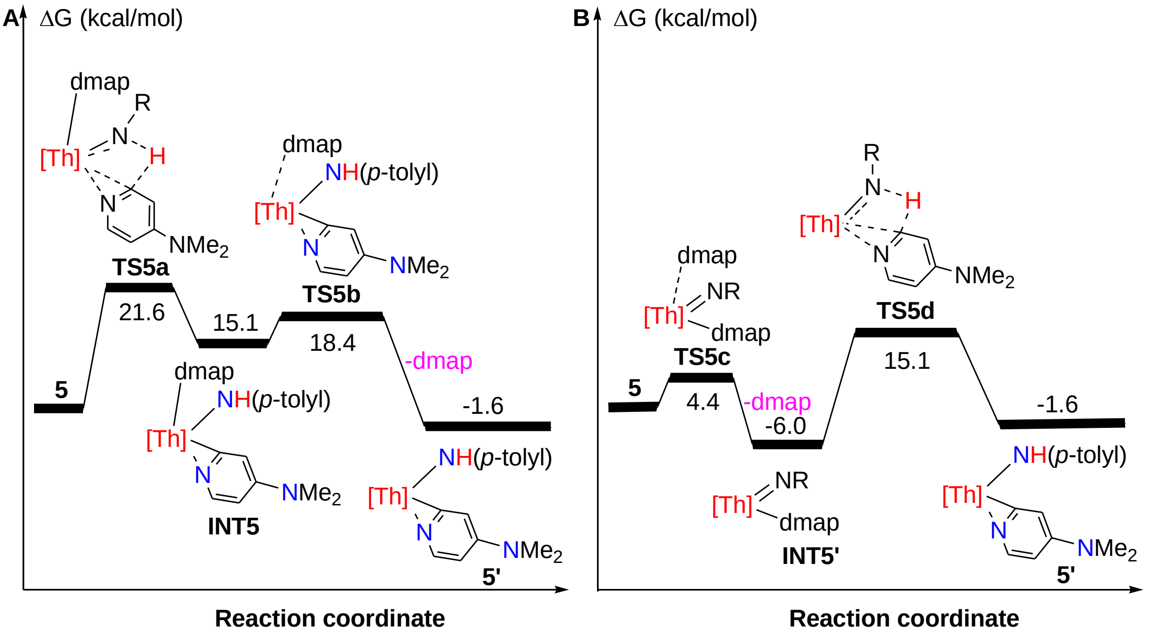

Figure S12. Free energy profile (kcal/mol) for the reaction of **5** ⇌ **5′** + dmap. [Th] = (η^5-C_5Me_5)$_2$Th. R = *p*-tolyl.

For the formation of **5′**, DFT investigations indicate that an *α*-H atom from dmap is transferred to the imido Th=N(*p*-tolyl) mioiety through the transition state **TS5a** to give **INT5** (Figure S12; **A**). In a subsequent step the dmap ligand dissociates from **INT5** *via* the transition state **TS5b** yielding product **5′**. The overall formation of the **5′** + dmap from **5** is energetically slightly exergonic (ΔG(298 K) = -1.6 kcal/mol) and entails an overall reaction barrier of $\Delta G^{\ddagger}$(298 K) = 21.6 kcal/mol. These energetic parameters indicate that an equilibrium between **5** and **5′** + dmap may be established in the solution, with the equilibrium favoring the dissociated species, **5′** + dmap, in agreement with the NMR spectroscopic observations. Alternatively, the pathway may commence with dmap dissociation from **5** *via* transition state **TS5c** to give the imido intermediate **INT5′** (Figure S12; **B**). In a subsequent an *α*-H transfer from dmap to the imido Th=N(*p*-tolyl) moiety occurs *via* transition state **TS5d** to give **5′**. The formation of the **INT5′** + dmap from **5** is energetically favorable (ΔG(298 K) = -6.0 kcal/mol), while the subsequent *α*-H transfer to from **5′** from **INT5′** is energetically unfavorable (ΔG(298 K) = 4.4 kcal/mol). This suggests that an equilibrium may exists between **INT5′** and **5′** exist in the solution, but intermediate **INT5′** is not observed in the ^{1}H NMR spectrum.

For the formation of **31b**, DFT investigations propose that an *α*-H atom is transferred from dmap to the Th[N(*p*-tolyl)C(Ph)=N] mioiety *via* transition state **TS31** to give **31b** (Figure S13). The conversion of **31a** to **31b** is slightly exergonic

(ΔG(298 K) = -2.1 kcal/mol) with an associated reaction barrier of ΔG‡(298 K) = 21.8 kcal/mol, suggesting that an equilibrium is attainable in solution, but favoring **31b**, consistent with the experimental NMR data.

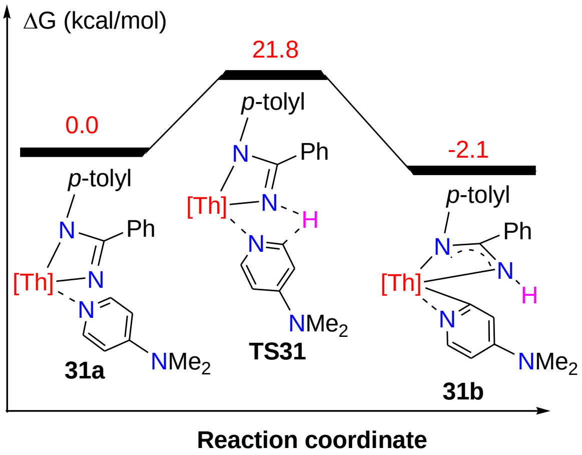

Figure S13. Free energy profile (kcal/mol) for the reaction of **31a** ⇌ **31b**. [Th] = (η^5-C_5Me_5)$_2$Th.

Table S10. The optimized Cartesian Coordinates (in Å) of stationary points for **5** ⇌ **5′** + dmap, obtained with B3PW91-PCM method.

Species	Cartesian coordinates				
**5**	C	3.377591	3.785980	13.392598	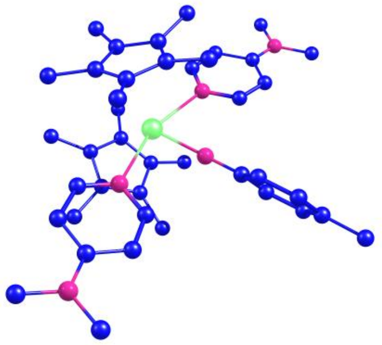 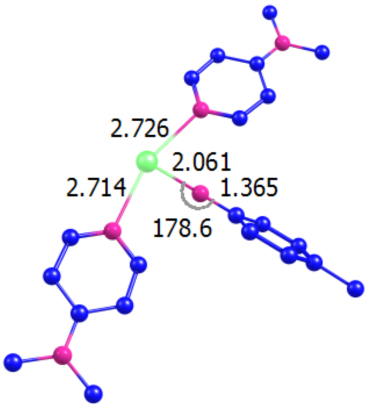
	C	3.088800	4.539050	12.218833	
	C	1.739251	4.982063	12.299400	
	C	1.200897	4.530017	13.534535	
	C	2.214130	3.786828	14.211713	
	C	4.724534	3.249464	13.786684	
	H	4.645281	2.299297	14.324466	
	H	5.265288	3.953767	14.437686	
	H	5.359675	3.069487	12.912616	
	C	4.112997	5.042722	11.244158	
	H	4.583871	5.961647	11.625357	
	H	3.680150	5.293865	10.270128	
	H	4.916357	4.320803	11.068738	
	C	1.067970	5.948737	11.366096	
	H	-0.007522	5.757517	11.267167	
	H	1.498583	5.917394	10.358803	
	H	1.168602	6.987387	11.717912	
	C	-0.101935	5.008929	14.111836	
	H	-0.052529	6.083940	14.341202	
	H	-0.347111	4.498623	15.048086	
	H	-0.952826	4.880174	13.429161	
	C	2.169642	3.278050	15.624456	
	H	2.698049	2.323947	15.728931	
	H	1.146447	3.121951	15.979283	
	H	2.649520	3.987632	16.315491	
	C	-0.095250	2.111729	9.608092	
	C	-1.042560	2.037005	10.667277	
	C	-0.975617	0.729286	11.225548	
	C	0.035195	0.007580	10.534454	
	C	0.568060	0.851531	9.521397	
	C	-0.004474	3.258902	8.639375	
	H	-0.967555	3.422777	8.134334	
	H	0.731891	3.069109	7.851779	
	H	0.258435	4.213628	9.114432	
	C	-2.061717	3.095848	10.979993	
	H	-2.533535	2.939099	11.956459	
	H	-2.874224	3.107711	10.237050	

	H -1.629996 4.104398 10.982776<br>C -1.978389 0.092289 12.142762<br>H -2.720239 -0.473844 11.559418<br>H -2.533771 0.825132 12.735417<br>H -1.516299 -0.612641 12.842024<br>C 0.321391 -1.456957 10.702275<br>H 1.303517 -1.724451 10.301196<br>H -0.423968 -2.075060 10.177602<br>H 0.310529 -1.762052 11.754492<br>C 1.474863 0.375382 8.421876<br>H 2.349430 -0.164476 8.801957<br>H 1.850003 1.198584 7.807348<br>H 0.940227 -0.310851 7.748783<br>C 3.561095 -0.629176 13.151422<br>C 4.080713 -0.708462 14.473453<br>H 3.841929 0.095059 15.167703<br>C 4.871257 -1.772656 14.895316<br>H 5.239413 -1.782420 15.921808<br>C 5.208846 -2.829495 14.039961<br>C 6.092869 -3.963134 14.493591<br>H 7.158939 -3.749302 14.326794<br>H 5.865698 -4.890627 13.954374<br>H 5.972666 -4.164280 15.564919<br>C 4.706394 -2.768240 12.733038<br>H 4.939244 -3.574930 12.037028<br>C 3.913218 -1.712233 12.298684<br>H 3.530485 -1.703661 11.280359<br>C 4.667992 1.294476 10.385158<br>H 4.651862 0.688124 11.284080<br>C 5.750880 1.249540 9.526272<br>H 6.580164 0.599396 9.778146<br>C 5.754020 2.031717 8.347705<br>C 4.605122 2.832744 8.142909<br>H 4.507002 3.472887 7.274202<br>C 3.581194 2.807486 9.070493<br>H 2.705899 3.429408 8.908324<br>C 7.943289 1.166358 7.722474<br>H 8.442808 1.434447 8.662567<br>H 8.662977 1.286728 6.911809<br>H 7.661006 0.106450 7.771638<br>C 6.743315 2.826306 6.266805<br>H 5.895726 2.549252 5.625679<br>H 7.660546 2.677280 5.695895<br>H 6.662230 3.895410 6.503951<br>C -0.990242 1.797841 14.655814<br>H -1.338379 2.644739 14.073386<br>C -1.696006 1.421533 15.782817<br>H -2.577903 1.986971 16.059615<br>C -1.262306 0.305673 16.538641<br>C -0.106405 -0.348817 16.052187<br>H 0.305143 -1.217175 16.552424<br>C 0.529977 0.120726 14.917391<br>H 1.423061 -0.368576 14.544461<br>C -3.101733 0.594827 18.109667<br>H -2.882403 1.646317 18.337326<br>H -3.472906 0.124374 19.020992<br>H -3.904175 0.563297 17.360381<br>C -1.440573 -1.270604 18.387960<br>H -1.450031 -2.174116 17.764072<br>H -2.091298 -1.445459 19.245656<br>H -0.419234 -1.120003 18.761452<br>N 2.780354 0.407540 12.729758<br>N 3.574318 2.058084 10.187447<br>N 6.793404 2.014808 7.468475<br>N 0.118596 1.187527 14.203620<br>N -1.917590 -0.110627 17.657099<br>Th 1.599279 1.952557 12.045989	
**TS5a**	C 3.850215 3.240079 14.060450<br>C 3.951967 3.736535 12.728049	

C	2.738098	4.407278	12.416548
C	1.897149	4.356771	13.564246
C	2.588356	3.641591	14.583912
C	4.952727	2.613730	14.867567
H	4.570674	1.865436	15.570437
H	5.489871	3.369141	15.460958
H	5.695316	2.119025	14.233467
C	5.216137	3.825287	11.925127
H	5.760599	4.748073	12.175774
H	5.028278	3.852122	10.846826
H	5.895825	2.990246	12.119308
C	2.488821	5.249820	11.198510
H	1.431347	5.269124	10.911439
H	3.061082	4.904787	10.330142
H	2.783992	6.295990	11.372341
C	0.631256	5.149954	13.726296
H	0.855049	6.223312	13.816380
H	0.080534	4.863097	14.626535
H	-0.050147	5.045641	12.873166
C	2.193538	3.505487	16.025670
H	2.360502	2.492077	16.406539
H	1.136561	3.734197	16.186118
H	2.780184	4.193934	16.651670
C	0.865933	1.525801	9.992836
C	-0.231311	1.873018	10.834072
C	-0.580469	0.723940	11.598907
C	0.320742	-0.322147	11.260326
C	1.199499	0.161435	10.251132
C	1.367234	2.383444	8.862025
H	0.551123	2.623343	8.166074
H	2.134964	1.871714	8.272414
H	1.786749	3.342111	9.190571
C	-1.020741	3.147330	10.737663
H	-1.547548	3.383044	11.668842
H	-1.786994	3.082310	9.950267
H	-0.388734	4.007201	10.490210
C	-1.840106	0.523180	12.389655
H	-2.576857	-0.026272	11.785137
H	-2.306904	1.468723	12.678875
H	-1.673515	-0.054385	13.303897
C	0.203686	-1.743756	11.727728
H	1.162935	-2.267770	11.701001
H	-0.500999	-2.309186	11.098614
H	-0.161063	-1.799676	12.758277
C	2.095723	-0.723998	9.432901
H	2.716250	-1.379422	10.051851
H	2.769005	-0.160025	8.781879
H	1.494288	-1.375833	8.782985
C	3.158739	-1.572749	13.979056
C	3.267491	-2.123707	15.280066
H	2.897438	-1.540584	16.119383
C	3.831850	-3.374659	15.496930
H	3.893995	-3.755618	16.516313
C	4.331550	-4.154503	14.444246
C	4.917715	-5.521979	14.685815
H	5.454689	-5.568587	15.640872
H	5.621571	-5.802726	13.893610
H	4.141993	-6.300456	14.717373
C	4.235283	-3.615100	13.157444
H	4.612527	-4.187729	12.310160
C	3.662771	-2.367009	12.924754
H	3.601748	-1.984732	11.909806
C	5.282086	0.250993	12.127273
H	5.043902	-0.047819	13.141443
C	6.537343	-0.001612	11.607038
H	7.265322	-0.498441	12.236821
C	6.840667	0.370585	10.275514
C	5.797374	1.017096	9.569562
H	5.928338	1.359613	8.550187

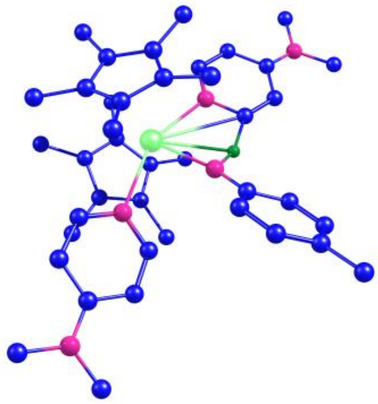

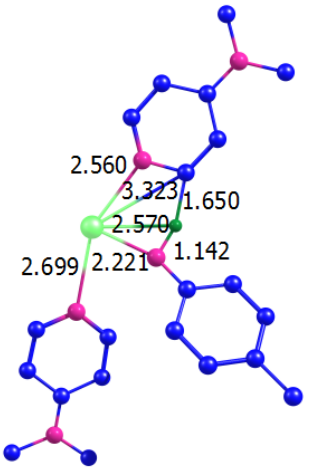

	C	4.584345	1.234015	10.192430	
	H	3.797012	1.752733	9.654940	
	C	9.100311	-0.515894	10.485365	
	H	9.383946	0.082106	11.361551	
	H	9.984411	-0.638141	9.858594	
	H	8.789131	-1.510083	10.830209	
	C	8.317672	0.548981	8.347512	
	H	7.625688	0.073750	7.640224	
	H	9.331874	0.257241	8.072615	
	H	8.235142	1.638325	8.234653	
	C	-0.779580	2.180722	14.840316	
	H	-1.027984	2.904943	14.066968	
	C	-1.587263	2.087448	15.953566	
	H	-2.452852	2.735150	16.032936	
	C	-1.266767	1.132835	16.951624	
	C	-0.119440	0.345958	16.709190	
	H	0.167800	-0.407654	17.438315	
	C	0.681342	0.475425	15.568772	
	H	1.943211	-0.111548	14.682491	
	C	-3.265743	1.748347	18.217342	
	H	-3.080021	2.828986	18.171774	
	H	-3.712134	1.531680	19.189635	
	H	-4.001824	1.494710	17.439580	
	C	-1.744086	-0.067071	19.020385	
	H	-1.842063	-1.063547	18.565246	
	H	-2.439540	-0.003734	19.859478	
	H	-0.726522	0.024231	19.419728	
	N	2.603047	-0.321478	13.774174	
	N	4.286955	0.858661	11.449808	
	N	8.054288	0.129081	9.711737	
	N	0.311018	1.417515	14.629492	
	N	-2.034844	0.996636	18.079430	
	Th	1.987549	1.521172	12.697896	
**INT5**	C	4.363411	2.412373	14.768059	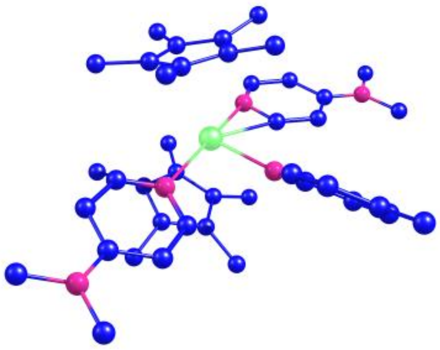 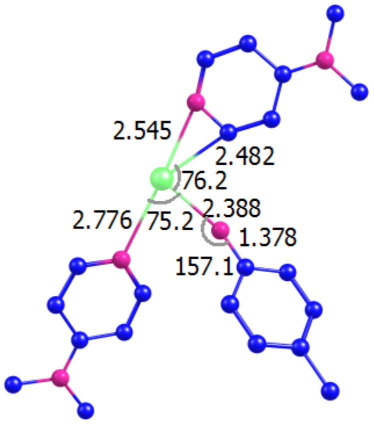
	C	4.932563	2.569144	13.475875	
	C	4.056390	3.387099	12.706606	
	C	2.979664	3.787636	13.544934	
	C	3.167646	3.185755	14.821743	
	C	5.020462	1.732026	15.935340	
	H	4.286544	1.378950	16.667569	
	H	5.702931	2.412174	16.467391	
	H	5.609015	0.862569	15.624045	
	C	6.351092	2.242329	13.118454	
	H	7.014335	3.061760	13.434960	
	H	6.498475	2.112612	12.042046	
	H	6.709905	1.334239	13.611783	
	C	4.328114	3.930937	11.332580	
	H	3.403566	4.104572	10.768268	
	H	4.956788	3.258614	10.737578	
	H	4.853404	4.897440	11.375955	
	C	1.963092	4.830647	13.181177	
	H	2.444105	5.807643	13.026033	
	H	1.220394	4.958459	13.972632	
	H	1.417525	4.597383	12.257671	
	C	2.413262	3.512716	16.077790	
	H	2.405001	2.674474	16.781618	
	H	1.370312	3.776859	15.880345	
	H	2.875361	4.366631	16.596212	
	C	1.736074	0.511849	10.253991	
	C	0.583236	1.097203	10.852914	
	C	0.001123	0.132752	11.721152	
	C	0.816187	-1.030120	11.696055	
	C	1.875903	-0.809573	10.770019	
	C	2.474194	1.120532	9.091641	
	H	1.780823	1.344372	8.268791	
	H	3.227916	0.437758	8.686672	
	H	2.979025	2.066254	9.332723	
	C	-0.004777	2.421035	10.458021	
	H	-0.649458	2.830792	11.241051	

	H -0.618431 2.332827 9.548316<br>H 0.766974 3.170105 10.241667<br>C -1.356416 0.200219 12.356520<br>H -2.066473 -0.433087 11.804414<br>H -1.758727 1.216425 12.352924<br>H -1.352748 -0.146541 13.395516<br>C 0.458906 -2.335233 12.346588<br>H 1.337142 -2.950312 12.560105<br>H -0.209326 -2.927477 11.702346<br>H -0.062117 -2.180772 13.297150<br>C 2.760214 -1.894994 10.227099<br>H 3.100589 -2.582650 11.007281<br>H 3.652308 -1.507438 9.727675<br>H 2.214130 -2.500144 9.487894<br>C 3.105552 -2.147355 14.968900<br>C 2.951785 -2.574831 16.313237<br>H 2.623911 -1.849102 17.057572<br>C 3.203776 -3.882958 16.703470<br>H 3.064397 -4.155544 17.749560<br>C 3.631018 -4.858316 15.790638<br>C 3.914203 -6.273677 16.224622<br>H 4.814665 -6.341504 16.851441<br>H 4.070253 -6.929688 15.360830<br>H 3.087314 -6.693798 16.811763<br>C 3.788044 -4.447083 14.463537<br>H 4.111878 -5.172454 13.717397<br>C 3.537826 -3.138675 14.058114<br>H 3.669147 -2.868369 13.015405<br>C 5.569183 -0.948905 12.965092<br>H 5.187889 -1.119282 13.965190<br>C 6.780868 -1.496385 12.582629<br>H 7.322575 -2.101040 13.300137<br>C 7.282827 -1.264850 11.281288<br>C 6.480540 -0.438758 10.458235<br>H 6.780311 -0.177836 9.450145<br>C 5.283366 0.051966 10.943984<br>H 4.679827 0.689479 10.306453<br>C 9.255062 -2.620752 11.737922<br>H 9.581673 -2.065081 12.627101<br>H 10.144403 -2.963856 11.207832<br>H 8.695577 -3.505025 12.069212<br>C 8.938611 -1.501342 9.510457<br>H 8.229296 -1.845401 8.746282<br>H 9.885095 -2.018165 9.347332<br>H 9.109825 -0.426249 9.364681<br>C -0.699350 2.963533 14.139532<br>H -0.900236 3.742902 13.405998<br>C -1.538255 2.811299 15.229192<br>H -2.387995 3.475586 15.335502<br>C -1.276647 1.784136 16.172420<br>C -0.140567 0.977220 15.923570<br>H 0.109143 0.168065 16.603716<br>C 0.669779 1.203837 14.813481<br>H 2.456616 -0.363710 15.394588<br>C -3.260632 2.419537 17.449532<br>H -2.997929 3.482210 17.539250<br>H -3.763715 2.122059 18.370999<br>H -3.977007 2.312632 16.622369<br>C -1.817113 0.501383 18.174683<br>H -1.867085 -0.474875 17.672547<br>H -2.560921 0.511081 18.973085<br>H -0.825084 0.596287 18.635809<br>N 2.853857 -0.847610 14.586901<br>N 4.789597 -0.190416 12.170963<br>N 8.461025 -1.792452 10.849074<br>N 0.372335 2.185959 13.935615<br>N -2.084966 1.593954 17.260276<br>Th 2.435325 0.961058 13.085433	
**TS5b**	C 4.239777 2.571806 14.391723	

C	4.525451	2.787086	13.015336
C	3.504682	3.626892	12.481933
C	2.616932	3.966609	13.536515
C	3.059288	3.303360	14.716781
C	5.129188	1.892458	15.394284
H	4.561948	1.284214	16.108105
H	5.698610	2.628594	15.981112
H	5.854927	1.232791	14.910708
C	5.827842	2.515008	12.321509
H	6.469221	3.408437	12.372807
H	5.700202	2.278511	11.260392
H	6.383563	1.690755	12.774445
C	3.510906	4.249309	11.114712
H	2.503584	4.528206	10.785687
H	3.935018	3.583944	10.354607
H	4.113012	5.170890	11.099093
C	1.543400	5.010371	13.441752
H	1.984081	6.009630	13.310925
H	0.931682	5.043019	14.346843
H	0.868135	4.847987	12.592473
C	2.545331	3.526861	16.110445
H	2.692670	2.646609	16.744963
H	1.476234	3.759607	16.125620
H	3.070829	4.364842	16.593524
C	1.300861	0.639258	10.374374
C	0.176669	1.301521	10.946283
C	-0.450754	0.392643	11.845733
C	0.293414	-0.816761	11.846899
C	1.363777	-0.675777	10.920055
C	2.139853	1.139189	9.232511
H	1.788334	0.727542	8.274323
H	3.192319	0.850016	9.328406
H	2.108032	2.229552	9.140149
C	-0.357968	2.648063	10.549232
H	-0.768687	3.196630	11.403990
H	-1.167320	2.556223	9.809910
H	0.412586	3.280421	10.094807
C	-1.793720	0.564014	12.492386
H	-2.547211	-0.046621	11.973524
H	-2.135091	1.601542	12.450931
H	-1.798676	0.258492	13.544579
C	-0.116037	-2.069329	12.566346
H	0.736643	-2.716240	12.790235
H	-0.832081	-2.654393	11.969522
H	-0.602602	-1.839825	13.520541
C	2.211327	-1.792859	10.381745
H	2.037278	-2.720732	10.933599
H	3.283589	-1.584060	10.423052
H	1.956288	-1.990685	9.330109
C	2.836796	-2.065502	14.733544
C	2.665936	-2.606098	16.031266
H	2.357081	-1.942951	16.839539
C	2.886223	-3.951334	16.301549
H	2.739874	-4.314981	17.318507
C	3.291079	-4.847395	15.303619
C	3.492466	-6.312903	15.592069
H	3.896144	-6.475493	16.598772
H	4.186893	-6.770066	14.877598
H	2.550734	-6.877275	15.530641
C	3.473754	-4.317918	14.020877
H	3.797657	-4.977079	13.215631
C	3.258611	-2.972817	13.735968
H	3.425362	-2.600304	12.731484
C	5.605391	-0.892356	13.063497
H	5.066784	-0.879184	14.006294
C	6.906237	-1.368362	13.016806
H	7.364614	-1.722033	13.932968
C	7.599092	-1.400083	11.784927
C	6.879213	-0.917585	10.667153

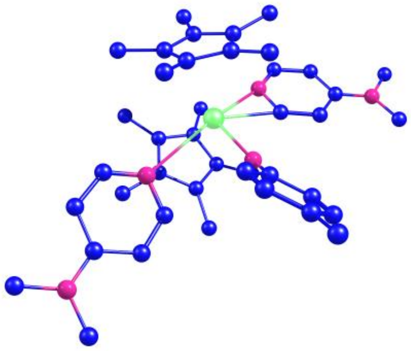

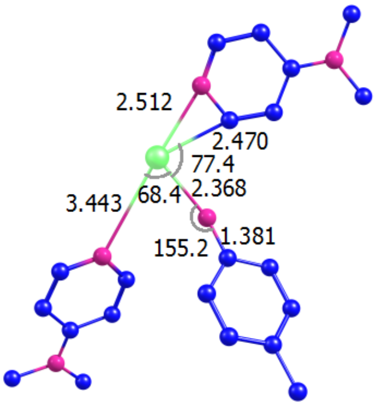

	H 7.316655 -0.897037 9.675730<br>C 5.583833 -0.458206 10.838905<br>H 5.044912 -0.085206 9.969966<br>C 9.574289 -2.340422 12.860130<br>H 9.689488 -1.546465 13.610671<br>H 10.570323 -2.682332 12.575059<br>H 9.048507 -3.182542 13.329278<br>C 9.546732 -1.863129 10.394307<br>H 9.013350 -2.479730 9.658337<br>H 10.550664 -2.273599 10.511570<br>H 9.641367 -0.846949 9.987445<br>C -0.968147 3.084310 14.419864<br>H -1.234134 3.859339 13.703374<br>C -1.719972 2.920192 15.568474<br>H -2.568662 3.572740 15.737106<br>C -1.378220 1.895278 16.489206<br>C -0.255434 1.100849 16.155507<br>H 0.053529 0.292618 16.811664<br>C 0.467403 1.337262 14.989689<br>H 2.259991 -0.303236 15.327581<br>C -3.291679 2.480347 17.892138<br>H -3.060925 3.552282 17.945916<br>H -3.714962 2.183041 18.852874<br>H -4.061419 2.332918 17.120877<br>C -1.766103 0.590204 18.510575<br>H -1.860859 -0.381424 18.005495<br>H -2.441403 0.595452 19.367627<br>H -0.740107 0.679025 18.890020<br>N 2.614296 -0.728454 14.466994<br>N 4.920516 -0.429182 12.004746<br>N 8.877033 -1.867182 11.679921<br>N 0.095245 2.317898 14.136355<br>N -2.101713 1.694137 17.632297<br>Th 2.055077 1.128731 13.108456	
**dmap**	C 4.500801 1.160223 10.334085<br>H 4.457916 0.475577 11.181559<br>C 5.478537 0.970867 9.368033<br>H 6.175205 0.147268 9.476617<br>C 5.543913 1.853065 8.265288<br>C 4.573408 2.880841 8.243037<br>H 4.536948 3.606916 7.438880<br>C 3.646137 2.963609 9.271920<br>H 2.901379 3.759440 9.247445<br>C 7.443839 0.635627 7.339565<br>H 8.054692 0.682835 8.251175<br>H 8.115976 0.704522 6.482879<br>H 6.952479 -0.347025 7.307056<br>C 6.490323 2.633593 6.156967<br>H 5.554209 2.580272 5.583514<br>H 7.311219 2.373461 5.487005<br>H 6.635053 3.673685 6.479401<br>N 3.579761 2.132668 10.320291<br>N 6.486212 1.721206 7.282585	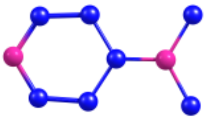
**TS5c**	C 3.587310 4.060594 12.933738<br>C 3.057858 4.816553 11.845165<br>C 1.712055 5.153966 12.154071<br>C 1.407313 4.611025 13.431014<br>C 2.569522 3.943693 13.919565<br>C 5.013909 3.610837 13.079079<br>H 5.091974 2.673803 13.640803<br>H 5.622777 4.359880 13.607631<br>H 5.487693 3.443126 12.105702<br>C 3.863578 5.416642 10.729438<br>H 4.294336 6.380565 11.040118<br>H 3.260731 5.619769 9.836338<br>H 4.700192 4.779774 10.426052<br>C 0.833322 6.091211 11.377148<br>H -0.230395 5.853201 11.495188<br>H 1.055197 6.078407 10.304396	

H	0.960131	7.131477	11.714387
C	0.164268	4.899792	14.221921
H	0.188080	5.919322	14.635993
H	0.058905	4.210454	15.063380
H	-0.749222	4.830140	13.617572
C	2.742457	3.390952	15.305238
H	3.620711	2.741882	15.367797
H	1.875427	2.806973	15.629547
H	2.886167	4.200159	16.037297
C	-0.214116	2.404178	9.602783
C	-0.984256	2.074741	10.753653
C	-0.682997	0.725496	11.112011
C	0.280927	0.232449	10.193464
C	0.565643	1.262903	9.256355
C	-0.346883	3.655264	8.780549
H	-1.066187	3.520888	7.958504
H	0.599941	3.959263	8.316454
H	-0.701731	4.502924	9.375099
C	-2.051406	2.931559	11.374138
H	-2.192211	2.702279	12.435673
H	-3.025481	2.782784	10.884381
H	-1.821833	4.000816	11.297970
C	-1.408586	-0.128791	12.112100
H	-2.101172	-0.813285	11.599389
H	-1.998430	0.466770	12.812812
H	-0.725730	-0.746089	12.705739
C	0.745217	-1.192404	10.104321
H	1.720571	-1.275690	9.612385
H	0.039695	-1.804753	9.521142
H	0.835461	-1.653802	11.092493
C	1.313486	1.060710	7.969991
H	2.223899	0.465917	8.100597
H	1.602754	2.007013	7.502213
H	0.685331	0.528175	7.240118
C	3.377917	-0.471801	13.287951
C	3.855250	-0.445231	14.626114
H	3.647500	0.436615	15.227514
C	4.562351	-1.507940	15.179243
H	4.900764	-1.435072	16.213298
C	4.852589	-2.666216	14.446955
C	5.646330	-3.800943	15.042575
H	6.717650	-3.725789	14.805007
H	5.304997	-4.773579	14.667595
H	5.562166	-3.818621	16.135561
C	4.389667	-2.708986	13.124998
H	4.586447	-3.596112	12.521916
C	3.678901	-1.655285	12.560139
H	3.322600	-1.729293	11.535467
C	4.705696	1.126600	10.331861
H	4.599172	0.538207	11.236454
C	5.800733	0.955411	9.507280
H	6.547230	0.222895	9.789398
C	5.927728	1.725738	8.326714
C	4.879787	2.645880	8.080503
H	4.878652	3.285499	7.206081
C	3.828724	2.738233	8.970929
H	3.028801	3.445936	8.775159
C	8.041356	0.641353	7.786369
H	8.531000	0.872955	8.741145
H	8.796335	0.683221	7.000509
H	7.657191	-0.385829	7.834471
C	7.068046	2.406369	6.284902
H	6.211919	2.232472	5.620080
H	7.975851	2.148508	5.738354
H	7.109115	3.477069	6.524872
C	-1.372745	1.853897	15.230264
H	-1.795552	2.760815	14.802845
C	-2.047049	1.226897	16.265546
H	-2.977964	1.654648	16.619877

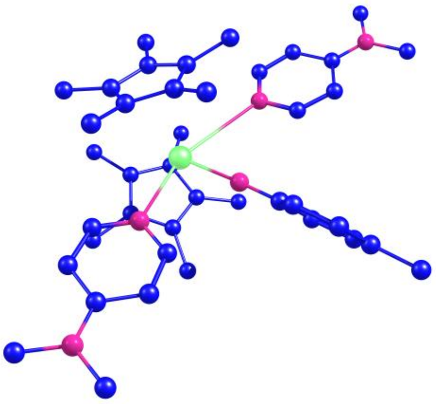

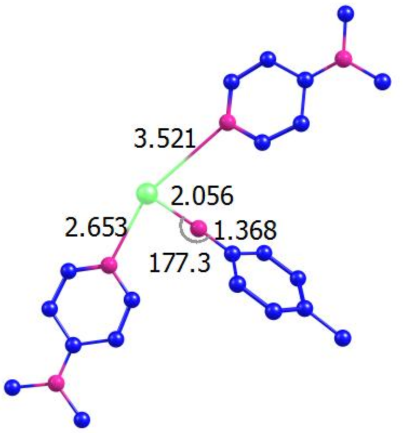

	C	-1.508497	0.048812	16.832758	
	C	-0.289959	-0.398398	16.272640	
	H	0.205608	-1.292217	16.633541	
	C	0.292904	0.311698	15.234257	
	H	1.225503	-0.035542	14.797400	
	C	-3.385977	-0.120484	18.378463	
	H	-3.285943	0.894100	18.787390	
	H	-3.720312	-0.774990	19.184705	
	H	-4.169676	-0.104504	17.608387	
	C	-1.542818	-1.825951	18.391342	
	H	-1.463793	-2.611813	17.627798	
	H	-2.173675	-2.201418	19.198337	
	H	-0.540601	-1.644642	18.801978	
	N	2.684748	0.568939	12.734141	
	N	3.707816	2.005134	10.093117	
	N	6.986096	1.592008	7.483440	
	N	-0.216953	1.432132	14.695906	
	N	-2.127248	-0.612327	17.855117	
	Th	1.720824	2.152253	11.845074	
**INT5′**	C	3.431836	3.569393	13.662483	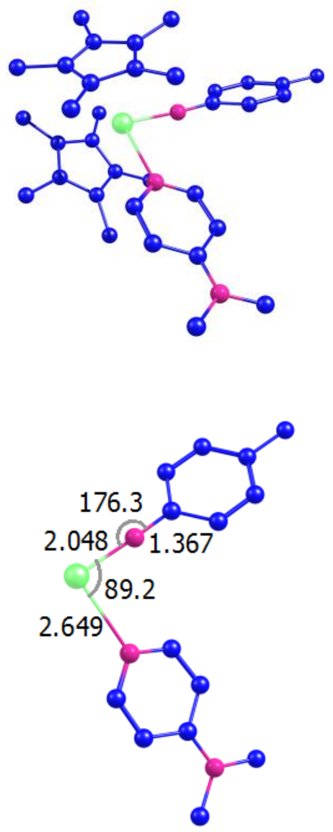
	C	3.383201	4.417571	12.518999	
	C	2.049048	4.906209	12.384082	
	C	1.271739	4.351629	13.435639	
	C	2.124374	3.515898	14.221025	
	C	4.665221	2.934965	14.238616	
	H	4.456586	1.950937	14.669455	
	H	5.105780	3.560346	15.029362	
	H	5.437736	2.787556	13.476387	
	C	4.568727	4.881170	11.720491	
	H	4.997564	5.801013	12.144857	
	H	4.311674	5.101491	10.678455	
	H	5.369675	4.134562	11.704009	
	C	1.589691	5.926285	11.380338	
	H	0.516594	5.848940	11.169743	
	H	2.119614	5.833221	10.424203	
	H	1.767242	6.952565	11.734680	
	C	-0.138204	4.726284	13.793396	
	H	-0.153898	5.528433	14.546072	
	H	-0.697729	3.886294	14.221717	
	H	-0.702318	5.089806	12.928974	
	C	1.737047	2.825383	15.499167	
	H	2.380720	1.963676	15.703102	
	H	0.701691	2.463130	15.481006	
	H	1.818284	3.501593	16.363258	
	C	-0.045872	1.670270	9.677245	
	C	-0.825903	2.014661	10.812820	
	C	-0.724406	0.946026	11.753998	
	C	0.099607	-0.067957	11.185936	
	C	0.533282	0.385982	9.909634	
	C	-0.004437	2.447603	8.392056	
	H	-0.902267	2.258754	7.785045	
	H	0.856196	2.174486	7.772412	
	H	0.033083	3.532679	8.556316	
	C	-1.742313	3.201490	10.902784	
	H	-2.067719	3.390970	11.929895	
	H	-2.651831	3.044425	10.304106	
	H	-1.281137	4.125478	10.530947	
	C	-1.475477	0.827369	13.051839	
	H	-2.411820	0.264943	12.924120	
	H	-1.748289	1.805316	13.464067	
	H	-0.894978	0.298684	13.817402	
	C	0.352199	-1.424773	11.775284	
	H	1.319307	-1.831442	11.466960	
	H	-0.424794	-2.140783	11.467575	
	H	0.356227	-1.400066	12.869881	
	C	1.338941	-0.415453	8.926395	
	H	2.077346	-1.047905	9.430342	
	H	1.882985	0.222538	8.221547	
	H	0.697928	-1.080476	8.329101	

	C	3.651698	-0.687260	13.002577	
	C	3.707562	-1.002531	14.385719	
	H	3.200819	-0.340502	15.085143	
	C	4.373891	-2.126990	14.858436	
	H	4.382117	-2.323901	15.930773	
	C	5.031552	-3.015768	13.996373	
	C	5.778353	-4.215581	14.520408	
	H	6.800925	-3.960748	14.835193	
	H	5.863641	-4.998729	13.757916	
	H	5.277711	-4.654710	15.391925	
	C	4.975935	-2.725105	12.627977	
	H	5.460158	-3.400184	11.921695	
	C	4.309551	-1.604514	12.141501	
	H	4.268798	-1.424487	11.068551	
	C	4.808604	1.550961	9.903124	
	H	4.896595	0.891248	10.761272	
	C	5.827987	1.642146	8.976487	
	H	6.717714	1.042950	9.126556	
	C	5.696709	2.504864	7.860476	
	C	4.482202	3.232550	7.784859	
	H	4.282405	3.920283	6.972176	
	C	3.527176	3.070405	8.767192	
	H	2.597887	3.630869	8.703399	
	C	7.903451	1.870670	7.047689	
	H	8.447753	2.132168	7.964452	
	H	8.547176	2.093252	6.196253	
	H	7.710468	0.790539	7.053868	
	C	6.495995	3.545807	5.809414	
	H	5.624163	3.276631	5.199465	
	H	7.378801	3.504741	5.170778	
	H	6.374245	4.580971	6.154101	
	N	3.005314	0.418330	12.525703	
	N	3.653848	2.247179	9.825907	
	N	6.670391	2.629207	6.922549	
	Th	1.923963	2.006614	11.816996	
**TS5d**	C	3.835745	3.954404	12.765978	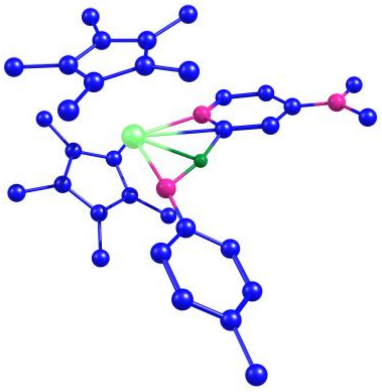 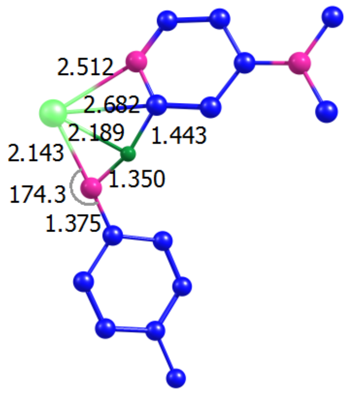
	C	3.409113	4.836846	11.730016	
	C	2.027662	5.118394	11.927852	
	C	1.595481	4.404993	13.082168	
	C	2.714655	3.682731	13.597085	
	C	5.246088	3.499571	13.010866	
	H	5.281642	2.489560	13.431302	
	H	5.763562	4.169423	13.713266	
	H	5.830508	3.485827	12.085196	
	C	4.296432	5.508457	10.721521	
	H	4.750654	6.418191	11.141077	
	H	3.741343	5.812110	9.827584	
	H	5.115527	4.860368	10.392512	
	C	1.219479	6.100906	11.128018	
	H	0.146344	5.993999	11.314022	
	H	1.376806	5.993016	10.046703	
	H	1.485637	7.137734	11.379695	
	C	0.277463	4.559813	13.786312	
	H	0.332782	5.352028	14.547511	
	H	-0.026013	3.645724	14.308316	
	H	-0.531420	4.834728	13.102267	
	C	2.731840	2.898172	14.878811	
	H	3.561180	2.185046	14.905124	
	H	1.806186	2.330235	15.034517	
	H	2.844224	3.560553	15.749974	
	C	0.071229	1.879149	9.264929	
	C	-0.637929	2.273147	10.432962	
	C	-0.494043	1.236009	11.403211	
	C	0.291703	0.197809	10.826578	
	C	0.649044	0.597942	9.508584	
	C	0.070621	2.619139	7.958069	
	H	-0.853334	2.428175	7.392642	
	H	0.904470	2.313449	7.318528	
	H	0.135782	3.706422	8.091534	

	C -1.547008 3.465060 10.526726<br>H -1.753736 3.745172 11.564057<br>H -2.518759 3.255668 10.055558<br>H -1.137533 4.347339 10.021559<br>C -1.186084 1.155524 12.735607<br>H -2.080187 0.517999 12.679829<br>H -1.516508 2.137449 13.088275<br>H -0.545023 0.724340 13.514644<br>C 0.568681 -1.138404 11.451263<br>H 1.529400 -1.550322 11.130637<br>H -0.212561 -1.866632 11.186835<br>H 0.597248 -1.079635 12.544225<br>C 1.392927 -0.245202 8.511212<br>H 2.117772 -0.904858 8.999699<br>H 1.940559 0.363468 7.783896<br>H 0.705242 -0.886902 7.941357<br>C 4.048650 -0.485023 12.199466<br>C 3.971409 -0.964457 13.529549<br>H 3.370592 -0.406892 14.244807<br>C 4.641358 -2.113788 13.932207<br>H 4.551258 -2.442058 14.967674<br>C 5.429887 -2.859386 13.044781<br>C 6.126161 -4.122301 13.483425<br>H 6.564859 -4.017253 14.483329<br>H 6.932962 -4.394348 12.793429<br>H 5.435852 -4.976859 13.526449<br>C 5.515936 -2.392499 11.728305<br>H 6.118394 -2.944827 11.007065<br>C 4.845763 -1.246248 11.310332<br>H 4.927603 -0.922336 10.274291<br>C 4.422187 2.148969 9.734419<br>H 4.080410 1.292453 10.843810<br>C 5.699110 2.050704 9.198979<br>H 6.464359 1.536120 9.770083<br>C 5.997977 2.600408 7.927351<br>C 4.928145 3.247733 7.252946<br>H 5.066402 3.705328 6.280652<br>C 3.685390 3.308653 7.847185<br>H 2.862317 3.812627 7.345766<br>C 8.314221 1.865175 8.102649<br>H 8.506411 2.356253 9.065347<br>H 9.228482 1.913961 7.509944<br>H 8.087659 0.808324 8.294193<br>C 7.499212 3.089115 6.066761<br>H 6.859639 2.638053 5.296921<br>H 8.537733 2.899367 5.793219<br>H 7.339364 4.175638 6.057445<br>N 3.381887 0.650304 11.803446<br>N 3.421121 2.772752 9.052276<br>N 7.240034 2.518939 7.376177<br>Th 2.166903 2.300874 11.177395	
**5′**	C 4.655045 3.062244 12.927459<br>C 4.245669 4.076947 12.017139<br>C 2.890980 4.414718 12.309822<br>C 2.468132 3.615110 13.407857<br>C 3.555074 2.769616 13.782255<br>C 6.043992 2.506591 13.053192<br>H 6.041210 1.474649 13.415380<br>H 6.645429 3.104160 13.754099<br>H 6.569323 2.511145 12.092377<br>C 5.141355 4.795068 11.049414<br>H 5.677149 5.616334 11.547577<br>H 4.578548 5.231934 10.218746<br>H 5.895031 4.130620 10.615045<br>C 2.099353 5.520350 11.671839<br>H 1.021479 5.319702 11.688366<br>H 2.388789 5.678323 10.627785<br>H 2.250228 6.476189 12.194784<br>C 1.195994 3.790121 14.186690	

H	1.331554	4.531756	14.987761
H	0.871028	2.862989	14.671883
H	0.368492	4.143840	13.563988
C	3.589153	1.875466	14.990164
H	4.330467	1.077390	14.888131
H	2.620218	1.399936	15.186104
H	3.846746	2.445127	15.895217
C	0.237020	1.419516	9.916505
C	-0.075628	1.222857	11.288185
C	0.535297	0.003861	11.710877
C	1.214880	-0.557678	10.592948
C	1.043453	0.324884	9.486384
C	-0.325543	2.507333	9.047892
H	-1.362004	2.283859	8.755454
H	0.250700	2.627563	8.126150
H	-0.344190	3.480328	9.554566
C	-1.066039	2.029917	12.077277
H	-0.910437	1.938948	13.156692
H	-2.093197	1.691584	11.874457
H	-1.032845	3.096535	11.825749
C	0.326806	-0.651375	13.048200
H	-0.685332	-1.073600	13.128823
H	0.439078	0.047716	13.887392
H	1.029440	-1.474489	13.208799
C	1.856312	-1.913420	10.532129
H	2.656119	-1.950325	9.786371
H	1.118439	-2.681988	10.258063
H	2.299963	-2.207957	11.487459
C	1.496581	0.059589	8.078155
H	2.436995	-0.500883	8.051784
H	1.657503	0.986383	7.517940
H	0.752134	-0.531223	7.524425
C	4.831129	-0.988685	12.246454
C	4.157197	-1.397839	13.418930
H	3.256095	-0.869921	13.719385
C	4.612208	-2.463395	14.189073
H	4.052481	-2.743136	15.081081
C	5.761765	-3.185570	13.848586
C	6.264520	-4.321625	14.701782
H	7.024838	-3.989655	15.423256
H	6.724132	-5.109795	14.093577
H	5.452297	-4.778539	15.278657
C	6.435884	-2.786096	12.687071
H	7.332982	-3.323157	12.380301
C	5.991827	-1.723773	11.908472
H	6.542573	-1.450920	11.008606
C	4.073841	2.225407	9.158517
H	4.982529	0.196718	10.675622
C	4.987653	2.287085	8.110101
H	5.836707	1.610423	8.113015
C	4.832013	3.218915	7.055253
C	3.699206	4.075186	7.125000
H	3.503206	4.818909	6.361569
C	2.825896	3.963235	8.186918
H	1.953121	4.609990	8.253698
C	6.880764	2.437140	5.990722
H	7.541865	2.609936	6.851146
H	7.449741	2.631917	5.080597
H	6.590944	1.378638	5.988259
C	5.549831	4.313988	4.992734
H	4.601216	4.189061	4.454064
H	6.359701	4.225387	4.267293
H	5.578730	5.330277	5.409363
N	4.370125	0.059922	11.480086
N	3.007010	3.067539	9.171171
N	5.718122	3.304282	6.020432
Th	2.771183	1.759358	11.228863

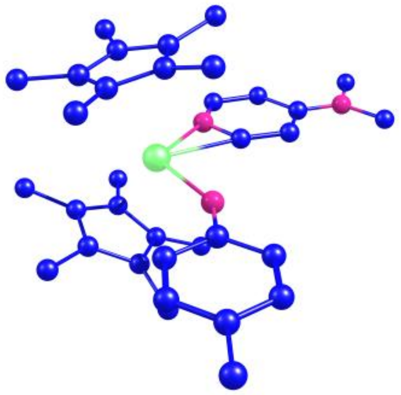

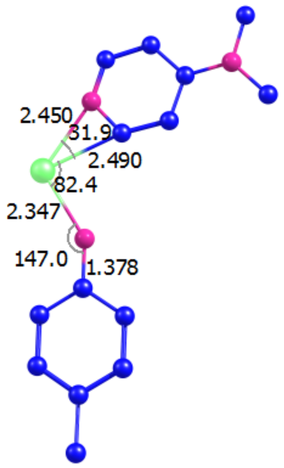

Table S11. Frequencies of the stationary points optimized for **5** ⇌ **5′** + dmap.

Species	Frequencies (cm^{-1})
**5**	15 18 20 23 27 31 34 42 45 51 52 61 63 66 68 85 88 91 92 95 97<br>101 105 109 113 118 121 122 127 131 142 142 147 151 152 158 165 174 178 187 190<br>192 194 197 200 205 208 217 220 226 241 255 259 261 264 271 274 282 287 287 293<br>294 296 302 303 327 353 357 362 370 379 394 399 426 432 434 485 488 500 537 549<br>550 551 551 552 553 556 557 557 563 563 608 609 630 632 633 635 655 676 678 721<br>751 752 778 781 796 818 820 825 825 826 826 827 836 839 841 931 941 944 970 974<br>977 979 981 983 985 988 1008 1015 1018 1019 1021 1022 1044 1045 1051 1054 1057 1058 1060 1064<br>1065 1065 1065 1087 1088 1090 1090 1092 1093 1095 1099 1125 1128 1132 1143 1147<br>1148 1149 1151 1152 1193 1193 1194 1195 1198 1211<br>1215 1247 1257 1259 1288 1291 1303 1343 1351 1363 1365 1381 1384 1422 1423 1424 1426 1427 1429 1431<br>1432 1434 1434 1436 1439 1442 1450 1452 1455 1456 1459 1461 1464 1474 1475 1490 1492 1492 1495<br>1497 1498 1500 1501 1501 1502 1504 1504 1504 1505 1505 1508 1509 1511 1512 1512 1513 1514 1515<br>1517 1520 1524 1529 1532 1533 1534 1535 1536 1537 1546 1547 1547 1548 1553 1558 1582 1584 1594<br>1597 1599 1667 1681 1683 3018 3019 3021 3022 3024 3026 3028 3028 3029 3030 3033 3034 3034 3042 3042<br>3071 3078 3080 3082 3090 3092 3092 3096 3096 3098 3099 3101 3102 3102 3103 3118 3121 3122 3128<br>3137 3140 3141 3141 3142 3143 3143 3157 3158 3169 3174 3181 3183 3189 3197 3226 3233 3240 3247 3250<br>3251 3254 3261
**TS5a**	-111 16 22 25 28 30 35 40 48 51 58 59 65 67 71 73 76 86 88 92 94<br>96 99 105 109 115 118 125 126 132 136 146 148 150 153 156 159 165 169 175 178<br>183 189 192 194 200 201 212 221 223 230 256 263 266 268 275 276 280 286 288 293<br>294 296 299 300 325 349 359 381 385 387 397 410 425 427 442 457 478 488 528 542<br>548 548 550 550 552 553 556 558 560 562 609 611 626 632 634 638 656 677 685 722<br>751 754 767 780 789 808 819 820 824 826 828 829 836 838 860 866 941 952 969 972<br>976 977 979 981 983 991 1008 1009 1017 1021 1026 1042 1044 1045 1051 1054 1056 1057 1057 1063<br>1064 1066 1066 1081 1088 1089 1090 1091 1091 1092 1096 1127 1128 1136 1147 1148<br>1150 1151 1151 1156 1193 1193 1194 1194 1202 1207 1213 1248 1261 1277<br>1286 1290 1301 1317 1321 1351 1366 1385 1403 1415 1420 1422 1423 1424 1429 1431 1432 1433 1434 1436<br>1439 1441 1450 1450 1452 1456 1458 1459 1464 1467 1473 1474 1489 1491 1493 1494 1495 1497 1500 1501<br>1502 1502 1503 1503 1505 1505 1507 1508 1509 1510 1512 1513 1514 1515 1515 1519 1521 1528 1529 1530<br>1532 1533 1533 1535 1544 1546 1547 1551 1552 1555 1557 1578 1587 1597 1604 1646 1678 1684 1730 3019<br>3025 3025 3027 3027 3030 3031 3032 3034 3034 3035 3036 3039 3041 3044 3083 3090 3091 3091 3093 3095<br>3095 3095 3100 3100 3104 3109 3112 3114 3115 3124 3128 3130 3133 3140 3145 3146 3150 3152<br>3154 3156 3158 3165 3167 3168 3173 3183 3184 3195 3209 3211 3238 3239 3248 3255 3263
**INT5**	18 20 25 27 32 35 42 47 52 54 56 59 61 70 71 87 89 93 96 99<br>102 105 111 114 116 120 123 127 130 133 138 144 146 152 158 165 166 169 171 181<br>184 187 193 195 199 204 213 219 220 232 233 257 266 270 273 276 278 280 289 290<br>294 298 301 302 306 322 330 365 366 372 381 395 404 427 429 442 465 487 488 511<br>520 549 550 551 552 554 554 557 557 559 560 604 610 611 628 629 632 634 660 680<br>698 718 752 755 777 780 788 818 820 821 822 825 826 828 831 837 865 872 936 945<br>965 972 973 977 980 984 984 989 1010 1011 1013 1019 1026 1045 1046 1052 1054 1056 1057<br>1060 1061 1062 1064 1067 1087 1090 1090 1091 1092 1092 1093 1098 1127 1128 1141 1147 1148<br>1149 1152 1154 1167 1192 1194 1195 1196 1209 1210 1214 1252 1258<br>1266 1286 1290 1301 1329 1352 1354 1368 1388 1407 1413 1421 1422 1424 1426 1429 1430 1433 1434 1434<br>1435 1438 1441 1446 1452 1453 1456 1459 1465 1467 1476 1478 1483 1488 1492 1493 1493 1495 1496 1500<br>1502 1503 1503 1504 1505 1505 1506 1507 1507 1510 1510 1512 1513 1513 1518 1520 1522 1523 1528 1529<br>1532 1532 1534 1534 1535 1545 1548 1548 1552 1554 1556 1556 1579 1586 1599 1622 1644 1680 1683 3023<br>3024 3025 3025 3026 3027 3029 3031 3031 3032 3033 3034 3034 3035 3042 3078 3081 3083 3084 3086 3087<br>3089 3092 3097 3100 3107 3108 3109 3113 3114 3123 3124 3132 3135 3142 3148 3151 3151 3154<br>3155 3157 3157 3162 3169 3173 3173 3178 3181 3182 3210 3224 3238 3244 3247 3253 3264 3467
**TS5b**	-48 16 20 24 30 31 35 43 50 51 53 54 57 60 66 74 82 88 91 97<br>101 103 109 115 116 119 127 128 132 134 139 146 153 156 160 163 167 169 171 177<br>184 186 188 194 200 204 205 207 214 227 231 252 266 272 274 277 279 282 288 290<br>291 292 297 302 303 321 333 367 375 379 384 386 409 425 429 432 467 486 490 515<br>523 549 550 551 551 552 554 555 556 557 561 608 612 623 626 632 634 640 658 679<br>700 720 752 754 773 778 790 819 820 821 822 824 826 828 838 846 864 873 935 961<br>968 969 972 976 978 981 984 987 1007 1009 1012 1015 1026 1045 1047 1052 1053 1057 1058 1060<br>1063 1063 1066 1067 1089 1091 1091 1092 1093 1093 1094 1099 1127 1130 1140 1146<br>1147<br>1147 1152 1153 1173 1194 1194 1196 1196 1209 1212 1212 1250 1265 1272<br>1286 1291 1300 1325 1351 1355 1365 1389 1409 1418 1420 1421 1423 1425 1426 1428 1431 1433 1433 1434

	1438 1440 1450 1452 1455 1456 1460 1463 1470 1476 1477 1485 1487 1490 1492 1493 1495 1497 1500 1501<br>1502 1502 1503 1503 1505 1506 1508 1508 1510 1511 1512 1512 1513 1515 1521 1522 1523 1528 1530<br>1531 1533 1534 1534 1535 1545 1547 1549 1555 1555 1557 1557 1579 1580 1604 1625 1647 1675 1679 3023<br>3025 3026 3026 3028 3029 3030 3031 3032 3033 3033 3034 3035 3036 3038 3085 3086 3088 3090 3090 3091<br>3094 3097 3099 3101 3102 3105 3106 3108 3123 3124 3129 3131 3138 3144 3150 3150 3152 3152 3156 3160<br>3162 3165 3169 3169 3175 3178 3178 3187 3188 3213 3228 3242 3245 3245 3248 3453
**dmap**	2079 140 196 249 274 386 410 483 546 554 679 754 771 821 837 966 986 991 1006 1093<br>1103 1144 1146 1154 1214 1268 1292 1365 1392 1425 1463 1484 1502 1505 1511 1535<br>1550 1577 1610 1672 3026 3034 3082 3083 3169 3170 3173 3177 3241 3242
**TS5c**	-79 12 16 20 25 25 30 33 36 40 45 48 51 63 69 71 75 83 89 92 93<br>94 97 104 106 110 112 116 124 127 129 133 136 141 146 148 149 153 162 169 171<br>180 183 190 194 197 211 218 222 226 228 253 259 271 275 277 281 282 283 284 286<br>287 291 293 293 327 354 361 365 372 375 384 401 420 426 435 485 488 501 535 549<br>550 551 552 552 553 554 556 556 562 566 608 608 630 633 634 639 656 677 678 720<br>750 752 773 779 798 821 821 823 823 824 827 829 837 840 843 935 943 946 967 971<br>972 980 983 984 986 986 1006 1008 1014 1017 1021 1023 1047 1048 1049 1051 1056 1057 1059 1060<br>1062 1065 1065 1091 1091 1091 1093 1094 1094 1097 1101 1129 1129 1135 1147 1147<br>1148 1149 1150 1154 1193 1194 1196 1197 1201 1214 1214 1248 1260<br>1268 1290 1293 1306 1344 1348 1366 1366 1383 1391 1420 1421 1422 1426 1427 1429 1430 1430 1432 1433<br>1435 1437 1440 1451 1452 1456 1457 1459 1464 1465 1476 1477 1488 1491 1492 1494 1495 1497 1498 1502<br>1502 1503 1504 1504 1505 1506 1507 1508 1508 1511 1511 1513 1513 1514 1514 1515 1522 1525<br>1527 1531 1533 1533 1535 1535 1540 1547 1547 1549 1549 1554 1555 1579 1585 1596 1597 1606 1668 1675<br>1683 3021 3022 3025 3025 3026 3026 3028 3028 3028 3029 3029 3031 3037 3037 3044 3082 3083 3084 3087<br>3089 3090 3090 3092 3095 3099 3099 3100 3100 3107 3109 3122 3124 3130 3133 3135 3137 3139 3142 3150<br>3153 3159 3160 3161 3169 3176 3179 3184 3193 3202 3207 3217 3224 3243 3247 3247 3253 3259
**INT5′**	16 22 23 27 31 35 38 43 50 62 68 72 83 84 87 89 92 96 98 101<br>103 107 110 113 118 131 134 139 145 150 151 157 165 169 174 175 183 193 198 215<br>232 258 273 275 278 282 287 288 292 294 295 297 302 326 356 368 374 375 384 402<br>423 427 487 504 534 548 549 551 552 552 556 560 563 567 603 603 629 634 641 642<br>657 674 720 749 777 799 819 821 821 825 826 830 838 842 938 941 944 972 975 976<br>979 980 982 1008 1010 1020 1024 1047 1048 1050 1053 1058 1059 1060 1062 1062 1066 1067 1091 1091<br>1092 1092 1094 1096 1127 1130 1135 1147 1148 1149 1191 1193 1193 1195 1202 1215 1248 1260 1288<br>1308 1344 1353 1368 1383 1423 1423 1425 1428 1431 1432 1432 1433 1437 1437 1439 1442 1448 1449 1452<br>1456 1459 1466 1473 1475 1488 1490 1493 1494 1497 1499 1499 1501 1503 1503 1506 1507 1507 1507 1509<br>1510 1512 1513 1514 1515 1520 1523 1529 1530 1532 1533 1536 1541 1542 1546 1548 1551 1554 1588 1593<br>1598 1669 1683 3027 3028 3028 3029 3029 3030 3030 3031 3033 3033 3035 3040 3047 3078 3083 3084 3088<br>3091 3093 3094 3098 3100 3103 3103 3106 3111 3117 3122 3126 3126 3132 3134 3137 3139 3141 3142 3151<br>3158 3161 3178 3186 3187 3192 3206 3232 3256 3257
**TS5d**	-1509 19 24 26 27 32 37 40 45 52 57 69 83 89 95 95 97 99 103<br>108 109 114 118 125 128 135 139 145 151 153 156 159 166 171 180 182 184 192 202<br>205 213 255 273 276 278 280 283 284 288 288 290 293 295 301 330 364 371 375 377<br>388 409 423 431 488 511 537 550 550 551 552 556 557 560 564 567 603 604 626 626<br>636 640 657 702 721 752 798 801 820 822 822 824 827 827 839 869 925 941 949 970<br>972 976 978 982 986 1008 1017 1025 1047 1048 1052 1053 1057 1059 1061 1062 1062 1067 1068 1087<br>1091 1092 1092 1094 1094 1124 1128 1131 1137 1147 1149 1175 1192 1192 1194 1196 1204 1216 1248 1288<br>1299 1314 1341 1347 1351 1413 1421 1424 1424 1429 1431 1432 1432 1434 1438 1439 1443 1449 1450 1454<br>1456 1457 1460 1472 1473 1473 1489 1490 1492 1495 1496 1498 1500 1503 1505 1505 1506 1506 1509 1509<br>1512 1513 1514 1515 1516 1522 1525 1527 1528 1531 1532 1535 1535 1544 1547 1548 1553 1554 1558 1584<br>1605 1652 1673 1921 3030 3031 3031 3032 3033 3033 3034 3034 3035 3036 3036 3037 3044 3082 3084 3088<br>3091 3094 3095 3099 3099 3101 3102 3106 3107 3111 3125 3135 3135 3138 3139 3140 3141 3142 3143 3144<br>3153 3163 3166 3174 3183 3188 3197 3202 3232 3251
**5′**	18 19 22 31 32 35 41 45 51 61 63 78 86 90 92 94 99 103 109 110<br>113 123 124 128 134 136 141 143 152 153 158 167 172 177 186 189 190 191 195 198<br>206 272 278 281 284 284 285 287 291 291 292 296 299 326 330 366 375 381 391 417<br>424 428 463 493 504 520 547 549 549 550 551 556 557 559 569 604 605 625 627 636<br>640 659 694 718 752 779 790 819 821 822 822 823 826 829 868 872 937 946 966 971<br>976 977 979 987 1011 1015 1027 1047 1048 1051 1052 1058 1058 1059 1061 1062 1065 1068 1079 1092<br>1092 1093 1093 1094 1128 1130 1142 1147 1151 1177 1192 1192 1195 1195 1212 1216 1233 1253 1288 1304<br>1333 1352 1353 1390 1412 1422 1424 1425 1426 1431 1433 1434 1435 1437 1439 1441 1449 1452 1454 1455<br>1457 1473 1473 1474 1479 1488 1490 1491 1492 1496 1496 1498 1501 1503 1505 1505 1507 1508 1508 1510<br>1511 1513 1513 1519 1519 1524 1527 1528 1530 1530 1533 1535 1546 1547 1551 1553 1558 1560 1581 1623<br>1649 1681 3030 3031 3031 3032 3032 3032 3033 3033 3034 3034 3035 3037 3039 3081 3088 3089 3091 3092

	3093 3094 3096 3099 3103 3109 3110 3113 3127 3132 3136 3138 3138 3140 3144 3144 3145 3150 3150 3162 3172 3173 3181 3183 3192 3216 3218 3248 3494

Table S12. The energies (with ZPE correction), enthalpies and free energies (in au at 298K) and corresponding relative values (in kcal/mol), obtained with B3PW91-PCM method

Species	E	H	G
**5**	-2276.783230 (0.0)	-2276.726248 (0.0)	-2276.850870 (0.0)
**TS5a**	-2276.750231 (20.7)	-2276.693894 (20.3)	-2276.816400 (21.6)
**INT5**	-2276.759495(14.9)	-2276.702565(14.9)	-2276.826780(15.1)
**TS5b**	-2276.755092(17.7)	-2276.698846(17.2)	-2276.821570(18.4)
**5′**	-1894.827216	-1894.779683	-1894.885965
**dmap**	-381.947577	-381.937901	-381.967410
**5′+dmap**	-2276.774793(5.3)	-2276.717584(5.4)	-2276.853375(-1.6)
**TS5c**	-2276.776589(4.2)	-2276.71978(4.1)	-2276.843810(4.4)
**INT5′**	-1894.834359	-1894.786714	-1894.893090
**dmap+INT5′**	-2276.781936(0.8)	-2276.724615(1.0)	-2276.860500(-6.0)
**TS5d**	-1894.800859(21.0) [a]	-1894.753846 (20.6)[a]	-1894.859460(21.1)[a]
**TS5d+dmap**	-2276.748436(21.8)	-2276.691747 (21.6)	-2276.826870(15.1)
**5′**	-1894.827216(4.5) [a]	-1894.779683 (4.4) [a]	-1894.885965 (4.4) [a]
**5′+dmap**	-2276.774793(5.3)	-2276.717584(5.4)	-2276.853375(-1.6)

[a] The relative energies in parentheses are with respect to the corresponding energies of **INT5′**.

Table S13. The optimized Cartesian Coordinates (in Å) of stationary points for **31a** ⇌ **31b**, obtained with B3PW91-PCM/6-31G(d,p)∪ECP60MWB method.

Species	Cartesian coordinates				
**31a**	Th	6.601037	11.876870	6.074365	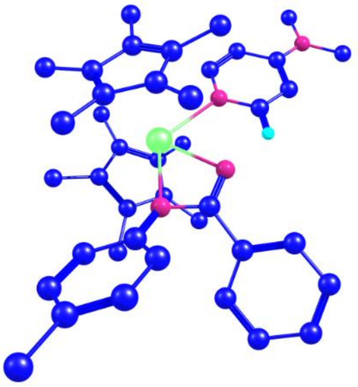 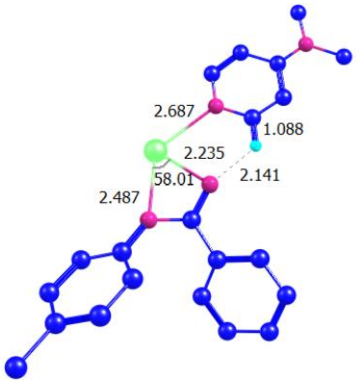
	N	8.073362	13.825682	6.540384	
	N	8.335265	12.391800	4.761099	
	N	6.585600	9.999015	4.152053	
	N	6.684261	7.176020	1.031112	
	C	3.873310	12.096827	5.075779	
	C	3.882218	12.906657	6.247753	
	C	4.702615	14.039772	5.995557	
	C	5.216176	13.926145	4.670227	
	C	4.697155	12.729841	4.099279	
	C	2.939233	10.940758	4.853163	
	H	2.954177	10.211323	5.672417	
	H	3.162305	10.406235	3.926449	
	H	1.902025	11.293717	4.773833	
	C	2.997346	12.715369	7.445141	
	H	2.793928	11.658524	7.645561	
	H	2.021981	13.200159	7.296152	
	H	3.428375	13.149387	8.352444	
	C	4.804703	15.242661	6.886442	
	H	4.869079	14.977691	7.946572	
	H	3.915659	15.878506	6.771496	
	H	5.678517	15.853846	6.655421	
	C	6.030485	14.953075	3.938941	
	H	6.595293	15.584346	4.629696	
	H	5.390658	15.613634	3.337381	
	H	6.750922	14.486127	3.260658	
	C	4.862088	12.329436	2.661728	
	H	5.889048	12.474796	2.312099	
	H	4.213054	12.932570	2.012259	
	H	4.605964	11.280276	2.493436	
	C	6.555009	10.846700	8.808405	
	C	6.127124	9.732904	8.030015	
	C	7.238959	9.281075	7.262662	
	C	8.347070	10.127874	7.549549	
	C	7.932157	11.078802	8.521914	
	C	5.747346	11.541252	9.868563	
	H	5.999885	12.603291	9.958907	
	H	5.913982	11.092637	10.857571	
	H	4.673307	11.479272	9.670329	
	C	4.794815	9.046647	8.135553	
	H	3.986821	9.744598	8.377446	
	H	4.797518	8.284138	8.926816	
	H	4.517175	8.532632	7.209006	
	C	7.335493	7.978729	6.523461	
	H	6.361593	7.613942	6.186890	
	H	7.756501	7.202918	7.178034	
	H	7.984414	8.044214	5.645414	
	C	9.755678	9.928274	7.068444	
	H	9.787148	9.532632	6.048867	
	H	10.299551	9.219711	7.709441	
	H	10.319571	10.865120	7.068076	
	C	8.864522	11.978747	9.279105	
	H	8.327841	12.632699	9.970385	
	H	9.460034	12.615159	8.619556	
	H	9.563249	11.382810	9.881422	
	C	8.239522	15.067657	7.142512	
	C	7.847655	15.262651	8.481004	
	H	7.454386	14.416148	9.034571	
	C	7.944940	16.506430	9.095307	
	H	7.629294	16.609207	10.132221	
	C	8.436241	17.626115	8.413824	
	C	8.808826	17.442484	7.077131	
	H	9.175033	18.292738	6.504158	

	C	8.710820	16.204303	6.449908	
	H	8.993048	16.112188	5.405793	
	C	8.574140	18.963946	9.092021	
	H	9.572022	19.094044	9.531083	
	H	7.847997	19.079444	9.903056	
	H	8.423781	19.788441	8.387509	
	C	8.843328	13.340923	5.506810	
	C	10.280351	13.752267	5.310098	
	C	10.819614	13.698510	4.018982	
	H	10.165552	13.407588	3.202392	
	C	12.160026	13.999790	3.793141	
	H	12.560209	13.965724	2.782907	
	C	12.990981	14.335860	4.863052	
	H	14.039813	14.563052	4.690721	
	C	12.468467	14.375853	6.155649	
	H	13.110658	14.629146	6.995202	
	C	11.120715	14.097983	6.376729	
	H	10.718905	14.150020	7.383490	
	C	7.565339	9.996046	3.222578	
	H	8.309096	10.779560	3.351844	
	C	7.640799	9.088069	2.184271	
	H	8.469705	9.167459	1.491798	
	C	6.651379	8.087403	2.039457	
	C	5.625243	8.095441	3.016339	
	H	4.822518	7.368708	3.001387	
	C	5.647839	9.042185	4.021476	
	H	4.864304	9.030167	4.772467	
	C	7.762100	7.208911	0.058564	
	H	7.781592	8.158038	-0.491161	
	H	7.617822	6.405193	-0.663085	
	H	8.740089	7.065266	0.534310	
	C	5.646308	6.166647	0.925168	
	H	5.616634	5.521180	1.812060	
	H	5.846857	5.537129	0.058544	
	H	4.655063	6.618010	0.792763	
**TS31**	Th	6.401315	11.860751	6.079750	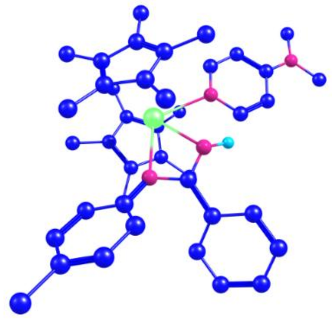 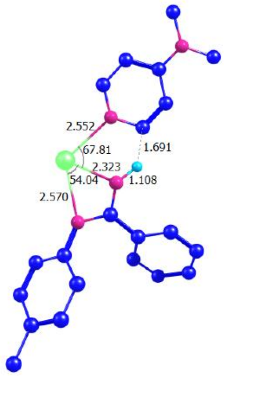
	N	8.034505	13.815092	6.421645	
	N	8.079370	12.509402	4.610072	
	N	6.703929	10.197870	4.168156	
	N	7.162505	7.295188	1.182235	
	C	3.688041	12.117109	5.149055	
	C	3.720938	12.889209	6.348492	
	C	4.540591	14.028352	6.118578	
	C	5.026241	13.958006	4.779977	
	C	4.494925	12.780222	4.179015	
	C	2.758018	10.960259	4.909926	
	H	2.804849	10.199016	5.697952	
	H	2.958189	10.465643	3.956395	
	H	1.716612	11.306800	4.875675	
	C	2.855995	12.663004	7.554515	
	H	2.608650	11.606343	7.692127	
	H	1.902545	13.200943	7.457901	
	H	3.324679	13.020121	8.476899	
	C	4.691584	15.187864	7.059144	
	H	4.745331	14.873094	8.106633	
	H	3.829899	15.864550	6.976815	
	H	5.589184	15.773213	6.851510	
	C	5.821227	15.009194	4.062442	
	H	6.383039	15.636394	4.759368	
	H	5.164305	15.670241	3.480818	
	H	6.539057	14.562935	3.368339	
	C	4.661364	12.413538	2.732932	
	H	5.695273	12.547373	2.400572	
	H	4.026315	13.042827	2.095053	
	H	4.394684	11.371957	2.538550	
	C	6.516978	10.891576	8.813446	
	C	5.969577	9.792116	8.088251	
	C	6.987352	9.276495	7.236894	
	C	8.159162	10.064193	7.421452	

	C 7.877830 11.044594 8.414034<br>C 5.835573 11.632231 9.930225<br>H 6.158933 12.676160 10.000866<br>H 6.047493 11.169866 10.903848<br>H 4.747911 11.636966 9.813127<br>C 4.619879 9.170212 8.309544<br>H 3.885836 9.893439 8.676998<br>H 4.671754 8.366233 9.056452<br>H 4.213905 8.721757 7.396374<br>C 6.943375 7.974186 6.495104<br>H 5.928666 7.694418 6.199246<br>H 7.323866 7.165481 7.134675<br>H 7.557600 7.993887 5.591369<br>C 9.499170 9.792105 6.798645<br>H 9.420479 9.624537 5.718513<br>H 9.967146 8.900653 7.238319<br>H 10.187615 10.628180 6.950108<br>C 8.915007 11.892669 9.092152<br>H 8.469796 12.568066 9.825845<br>H 9.490314 12.501710 8.390253<br>H 9.626701 11.256366 9.634094<br>C 8.265516 15.030969 7.066338<br>C 7.921082 15.186244 8.419881<br>H 7.514573 14.334071 8.953978<br>C 8.081982 16.403369 9.073787<br>H 7.804164 16.479695 10.123307<br>C 8.587546 17.529144 8.413909<br>C 8.905206 17.384201 7.058232<br>H 9.275872 18.243179 6.502027<br>C 8.744112 16.173410 6.392607<br>H 8.979008 16.110537 5.334672<br>C 8.792932 18.836965 9.131773<br>H 9.816527 18.927023 9.517804<br>H 8.115564 18.936488 9.985547<br>H 8.624098 19.690117 8.466969<br>C 8.745073 13.365248 5.361742<br>C 10.177851 13.679824 5.078923<br>C 10.635356 13.597131 3.757125<br>H 9.926616 13.361054 2.969034<br>C 11.977256 13.817253 3.457702<br>H 12.317604 13.756898 2.427661<br>C 12.881554 14.109226 4.478539<br>H 13.930030 14.276436 4.247204<br>C 12.436572 14.183601 5.799184<br>H 13.138295 14.403752 6.598897<br>C 11.092645 13.977987 6.098328<br>H 10.750413 14.047018 7.125586<br>C 7.892149 10.164547 3.461074<br>H 8.452408 11.669783 3.990171<br>C 8.028739 9.168874 2.487555<br>H 8.968097 9.132223 1.942083<br>C 7.016001 8.236488 2.165376<br>C 5.821529 8.330908 2.921911<br>H 4.986998 7.656686 2.771548<br>C 5.733204 9.302815 3.894392<br>H 4.827118 9.359200 4.496895<br>C 8.432532 7.161234 0.495960<br>H 8.711488 8.091703 -0.012814<br>H 8.351620 6.378129 -0.258879<br>H 9.247774 6.893036 1.181841<br>C 6.117544 6.320221 0.942561<br>H 5.951418 5.662868 1.808044<br>H 6.396747 5.694955 0.093728<br>H 5.165533 6.806465 0.696997	
**31b**	C -4.234866 5.628568 3.645308<br>C -3.824519 4.578189 4.513296<br>C -4.392876 4.821341 5.794274<br>C -5.125244 6.039676 5.729629<br>C -5.026034 6.542024 4.397224	

C	-4.046227	5.638756	2.158114
H	-3.034858	5.339750	1.865789
H	-4.232690	6.628544	1.735513
H	-4.740537	4.937117	1.675061
C	-3.083810	3.341699	4.092871
H	-2.363580	3.551778	3.296227
H	-3.772636	2.573230	3.713835
H	-2.526248	2.903207	4.924762
C	-4.409305	3.852074	6.936713
H	-5.235909	3.136373	6.823635
H	-4.546658	4.352695	7.899129
H	-3.483766	3.273074	6.997926
C	-6.001618	6.584398	6.821984
H	-6.245088	7.638567	6.664058
H	-5.542286	6.497379	7.813161
H	-6.955794	6.041649	6.870203
C	-5.740029	7.740328	3.841776
H	-6.686152	7.459541	3.358906
H	-5.136701	8.257442	3.088560
H	-5.987391	8.468845	4.620361
C	-2.149269	9.821689	5.683214
C	-0.837161	9.300198	5.860638
C	-0.802511	8.606151	7.106074
C	-2.096342	8.699042	7.693018
C	-2.928870	9.442948	6.809407
C	-2.595482	10.767023	4.607244
H	-3.603413	10.540921	4.240232
H	-1.918481	10.751860	3.749289
H	-2.620171	11.800968	4.978165
C	0.347713	9.591691	4.986497
H	0.831312	10.535107	5.275596
H	0.066861	9.683837	3.933423
H	1.107068	8.807291	5.052229
C	0.425270	8.039148	7.760019
H	1.161121	7.703860	7.022894
H	0.192314	7.180668	8.395961
H	0.923944	8.789053	8.389983
C	-2.475317	8.287451	9.084927
H	-2.537089	9.161431	9.748016
H	-1.737783	7.607182	9.515847
H	-3.450862	7.789183	9.129494
C	-4.316102	9.927344	7.117310
H	-4.861162	9.234029	7.764572
H	-4.913439	10.076812	6.211797
H	-4.289992	10.894567	7.639263
C	-1.172465	7.420484	2.755762
C	-0.539333	7.835743	1.464955
C	-1.345357	8.075747	0.344351
H	-2.414328	7.890965	0.406533
C	-0.784452	8.524566	-0.849368
H	-1.419977	8.695021	-1.714026
C	0.588573	8.752440	-0.933006
H	1.027130	9.106409	-1.861935
C	1.397687	8.522645	0.180417
H	2.467547	8.702727	0.122435
C	0.840716	8.059897	1.369550
H	1.473615	7.875976	2.231393
C	0.357319	5.627606	3.261865
C	0.299662	4.835450	2.101654
H	-0.553535	4.929895	1.435944
C	1.314892	3.933029	1.798482
H	1.236049	3.333459	0.893548
C	2.426985	3.773517	2.633537
C	2.475447	4.553626	3.794792
H	3.324316	4.454599	4.469086
C	1.465060	5.459655	4.106466
H	1.515231	6.046224	5.018425
C	3.536001	2.816258	2.282430
H	3.148821	1.911608	1.802448

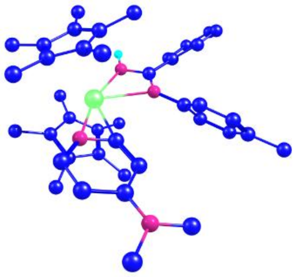

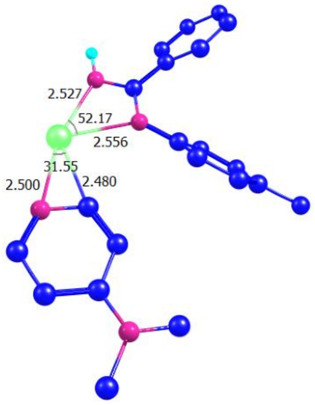

	H 4.098615 2.511492 3.170137<br>H 4.251465 3.269436 1.584325<br>C -1.081356 5.063171 6.475117<br>C -0.218054 3.994333 6.706771<br>H 0.402140 3.640461 5.891120<br>C -0.142445 3.380289 7.979183<br>C -0.995259 3.900808 8.987874<br>H -1.013567 3.483529 9.987411<br>C -1.835299 4.954608 8.687261<br>H -2.504045 5.351780 9.448152<br>C 1.474324 1.752550 7.153873<br>H 0.833415 1.316394 6.375083<br>H 2.112042 0.963164 7.553376<br>H 2.120851 2.498840 6.678538<br>C 0.671128 1.668451 9.522231<br>H 0.886699 2.366602 10.339955<br>H 1.436314 0.891766 9.542619<br>H -0.300087 1.194440 9.724016<br>N -0.656692 6.528455 3.614853<br>N -2.290415 7.989014 3.173743<br>N -1.874541 5.521550 7.471911<br>N 0.704249 2.335735 8.235783<br>Th -2.394058 6.901676 5.452671<br>H -2.590047 8.779769 2.618829	

Table S14. Frequencies of the stationary points optimized for **31a** ⇌ **31b**.

Species	Frequencies (cm^{-1})
**31a**	18 23 24 27 31 34 40 43 52 60 67 72 77 80 87 89 93 96 100 105<br>110 113 121 122 128 131 134 139 143 145 149 152 161 164 166 171 172 180 184 188<br>190 193 198 199 207 222 254 262 271 271 279 281 283 286 289 290 292 294 296 301<br>339 372 377 382 390 393 398 415 418 432 435 469 489 516 540 550 551 551 552 552<br>555 558 559 605 607 619 626 632 634 635 637 644 661 677 696 714 726 748 751 780<br>803 811 818 819 820 824 827 835 838 842 861 930 940 949 959 960 968 971 972 979<br>979 981 998 1005 1014 1020 1027 1036 1036 1038 1041 1045 1047 1047 1050 1052 1053 1054 1056 1057<br>1082 1083 1084 1084 1085 1095 1102 1118 1120 1126 1138 1139 1141 1148 1179 1188 1190 1190 1192 1195<br>1201 1205 1246 1268 1273 1284 1323 1330 1347 1365 1374 1384 1389 1404 1405 1406 1407 1411 1412 1415<br>1416 1417 1419 1423 1425 1445 1445 1450 1450 1452 1455 1471 1472 1474 1475 1476 1477 1479 1480 1481<br>1483 1485 1486 1487 1487 1489 1489 1490 1492 1492 1494 1495 1495 1497 1502 1503 1505 1511 1513 1516<br>1518 1518 1527 1531 1540 1542 1544 1546 1553 1581 1593 1614 1644 1662 1675 1683 3026 3028 3030 3030<br>3031 3031 3031 3032 3033 3036 3039 3039 3041 3082 3093 3093 3094 3095 3097 3104 3105 3106 3107 3107<br>3109 3120 3127 3132 3134 3135 3141 3144 3147 3147 3151 3158 3167 3172 3176 3177 3181 3188 3191 3198<br>3211 3216 3216 3221 3222 3225 3248 3249
**TS31**	-67 21 25 30 33 36 37 45 55 57 59 60 69 73 78 86 92 96 103 107<br>110 116 119 125 127 130 137 138 142 144 147 151 154 157 161 163 169 174 177 184<br>187 191 194 197 200 219 246 270 272 274 275 281 282 284 287 290 293 296 301 306<br>343 378 386 390 393 397 404 413 416 425 430 471 480 511 520 542 549 551 551 552<br>558 559 566 605 606 606 626 633 636 637 640 645 663 685 694 713 720 746 755 784<br>802 806 810 818 819 822 824 836 839 863 867 884 946 950 960 962 967 973 973 974<br>977 982 986 1006 1007 1015 1016 1030 1036 1038 1040 1042 1046 1047 1048 1050 1055 1056 1057 1057<br>1074 1082 1085 1085 1086 1086 1109 1119 1121 1139 1139 1142 1149 1161 1183 1188 1189 1190 1192 1200<br>1203 1205 1212 1247 1278 1293 1308 1321 1326 1337 1350 1378 1404 1406 1406 1407 1407 1411 1412 1416<br>1417 1418 1421 1423 1430 1444 1445 1449 1449 1451 1453 1463 1469 1470 1474 1475 1476 1477 1478 1479<br>1481 1481 1483 1484 1485 1487 1489 1490 1490 1492 1492 1492 1495 1498 1502 1503 1510 1511 1512 1516<br>1517 1517 1522 1536 1538 1540 1541 1544 1546 1556 1577 1620 1644 1646 1664 1677 2189 3017 3028 3029<br>3032 3033 3033 3034 3035 3035 3036 3039 3042 3046 3081 3087 3088 3095 3095 3099 3100 3103 3109 3112<br>3112 3115 3122 3132 3134 3139 3139 3139 3143 3152 3154 3154 3159 3160 3162 3167 3169 3181 3182 3193<br>3196 3206 3216 3217 3224 3227 3230 3240
**31b**	19 21 24 28 29 32 42 46 51 56 60 64 73 75 77 86 92 95 99 104<br>110 113 118 122 126 131 135 138 145 154 154 158 161 165 167 170 175 180 181 184<br>186 191 195 205 213 217 256 269 278 280 282 283 285 289 291 292 293 295 301 302<br>333 370 375 381 386 393 409 412 415 427 428 433 492 502 529 549 551 552 552 554<br>556 557 559 605 606 607 626 630 631 632 636 639 654 662 696 701 715 723 749 754<br>788 797 811 818 820 821 823 824 837 844 863 885 920 943 951 961 964 965 968 973<br>974 983 983 1005 1005 1010 1015 1031 1034 1035 1039 1040 1044 1046 1048 1049 1051 1053 1056 1057<br>1082 1083 1084 1085 1085 1086 1110 1119 1120 1129 1137 1140 1141 1169 1184 1189 1191 1191 1192 1199<br>1203 1203 1206 1244 1281 1296 1317 1325 1337 1349 1350 1376 1399 1400 1403 1404 1405 1408 1410 1412<br>1414 1415 1417 1420 1440 1445 1449 1449 1450 1452 1458 1472 1473 1474 1474 1475 1477 1478 1479 1480<br>1481 1481 1483 1484 1486 1488 1489 1489 1492 1492 1492 1495 1496 1498 1503 1505 1511 1513 1514 1514<br>1516 1518 1539 1541 1542 1543 1545 1546 1554 1558 1576 1623 1644 1646 1667 1675 3019 3026 3027 3029<br>3029 3029 3030 3030 3031 3031 3033 3033 3043 3084 3086 3089 3090 3092 3095 3098 3105 3106 3107 3107<br>3108 3110 3131 3135 3138 3139 3140 3142 3143 3145 3148 3150 3152 3160 3170 3177 3181 3183 3195 3203<br>3209 3212 3221 3222 3230 3231 3245 3625

Table S15. The energies (with ZPE correction), enthalpies and free energies (in au at 298K) and corresponding relative values (in kcal/mol), obtained with B3PW91-PCM/6-31G(d,p)∪ECP60MWB method.

Species	E	H	G
**31a**	-2219.19173<br>(0.0)	-2219.13843<br>(0.0)	-2219.27008<br>(0.0)
**TS31**	-2219.15882<br>(20.6)	-2219.10623<br>(20.2)	-2219.23538<br>(21.8)
**31b**	-2219.19477<br>(-1.9)	-2219.14131<br>(-1.8)	-2219.27346<br>(-2.1)

4. NMR spectra

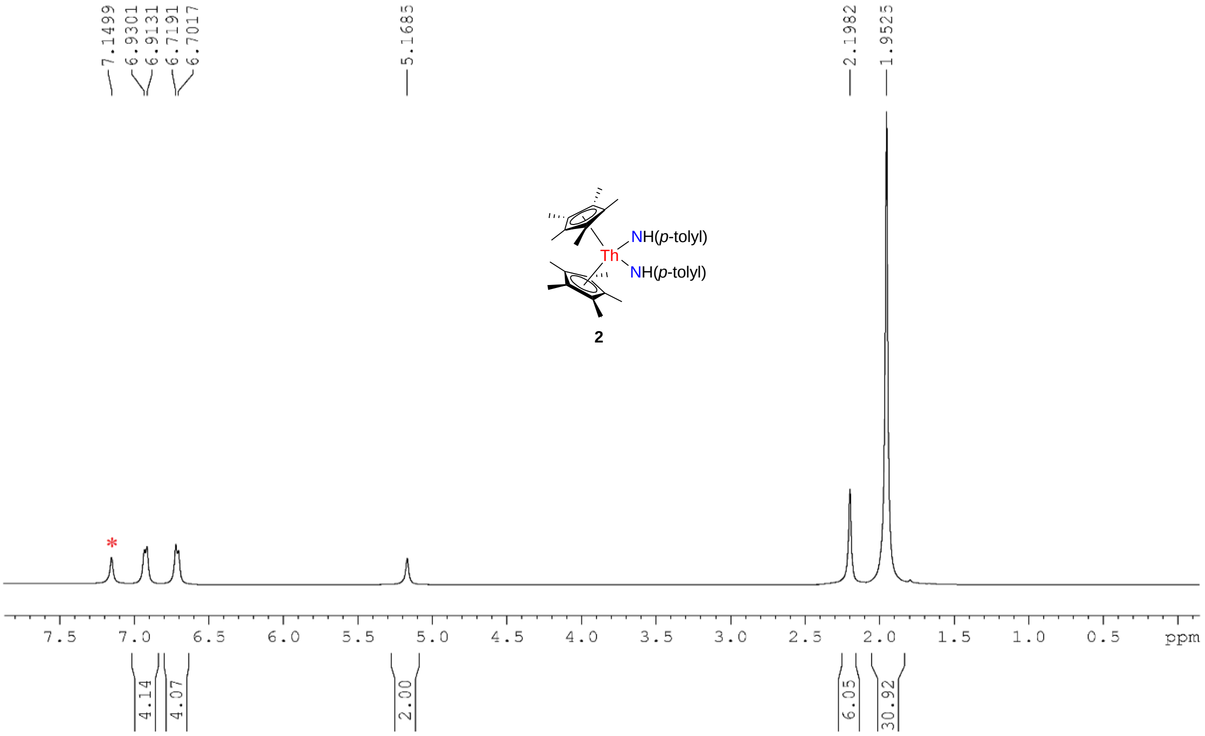

Figure S14. ^{1}H NMR (C_6D_6; 20 ℃) spectrum for compound (η^5-C_5Me_5)$_2$Th(NH-*p*-tolyl)$_2$ (**2**) (* solvent). ^{1}H NMR (C_6D_6): δ 6.92 (d, J = 6.8 Hz, 4H, phenyl), 6.71 (d, J = 7.0 Hz, 4H, phenyl), 5.17 (s, 2H, N*H*), 2.20 (s, 6H, tolylC*H*$_3$), 1.95 (s, 30H, CpC*H*$_3$) ppm.

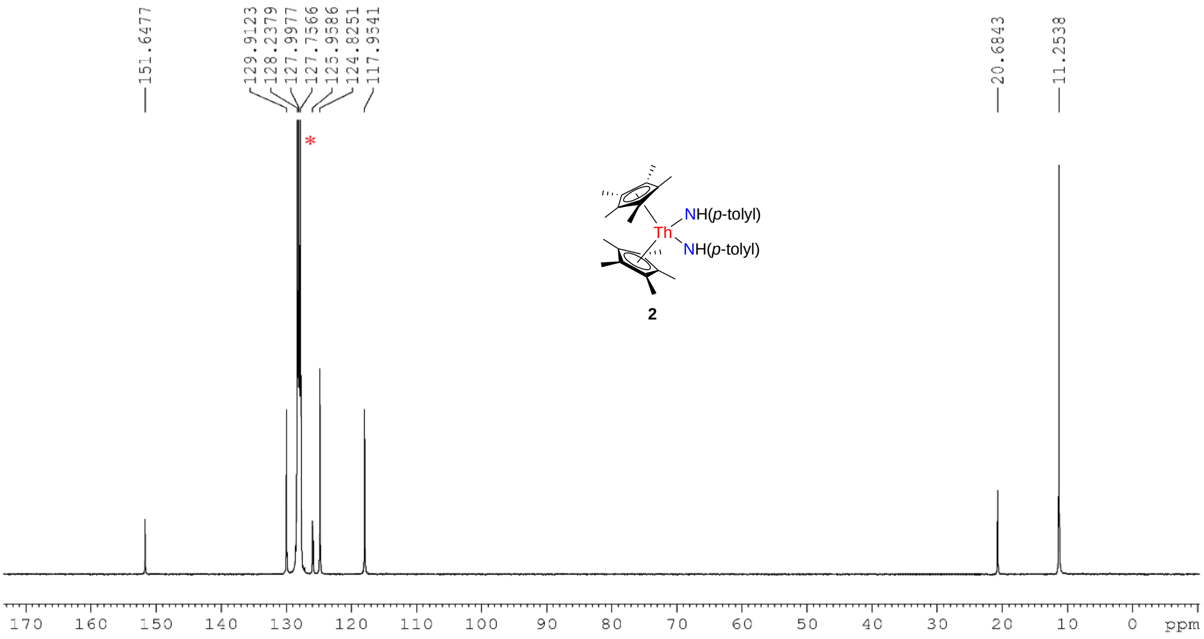

Figure S15. ^{13}C{^{1}H} NMR (C_6D_6; 20 ℃) spectrum for compound (η^5-C_5Me_5)$_2$Th(NH-*p*-tolyl)$_2$ (**2**) (* solvent). ^{13}C{^{1}H} NMR (C_6D_6): δ 151.6 (phenyl *C*), 129.9 (phenyl *C*), 126.0 (phenyl *C*), 124.8 (phenyl *C*), 118.0 (ring *C*), 20.7 (tolyl*C*H$_3$), 11.3 (Cp*C*H$_3$) ppm.

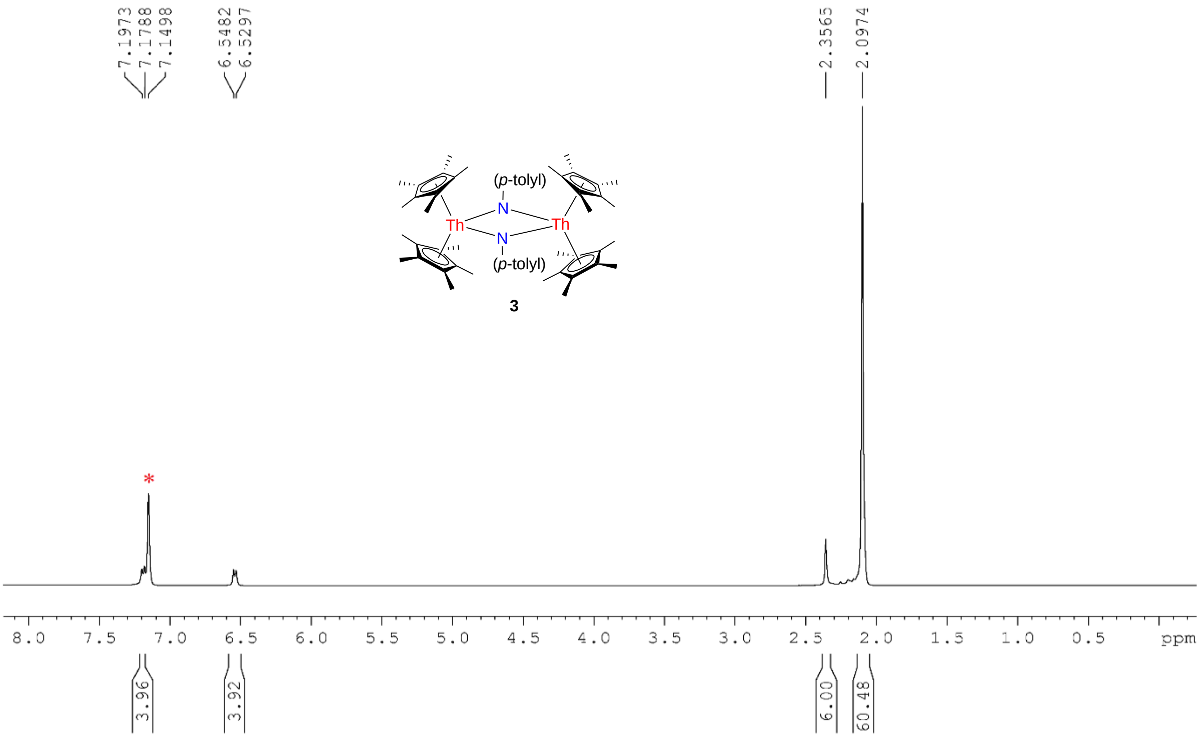

Figure S16. ¹H NMR (C_6D_6; 20 ℃) spectrum for compound $[(\eta^5\text{-}C_5Me_5)_2Th]_2[\mu\text{-}N(p\text{-tolyl})]_2$ C_6H_6 (**3** C_6H_6) (* solvent). ¹H NMR (C_6D_6): δ 7.19 (d, J = 7.4 Hz, 4H, phenyl), 7.15 (s, 6H, C_6H_6), 6.54 (d, J = 7.4 Hz, 4H, phenyl), 2.36 (s, 6H, tolylCH_3), 2.10 (s, 60H, CpCH_3) ppm.

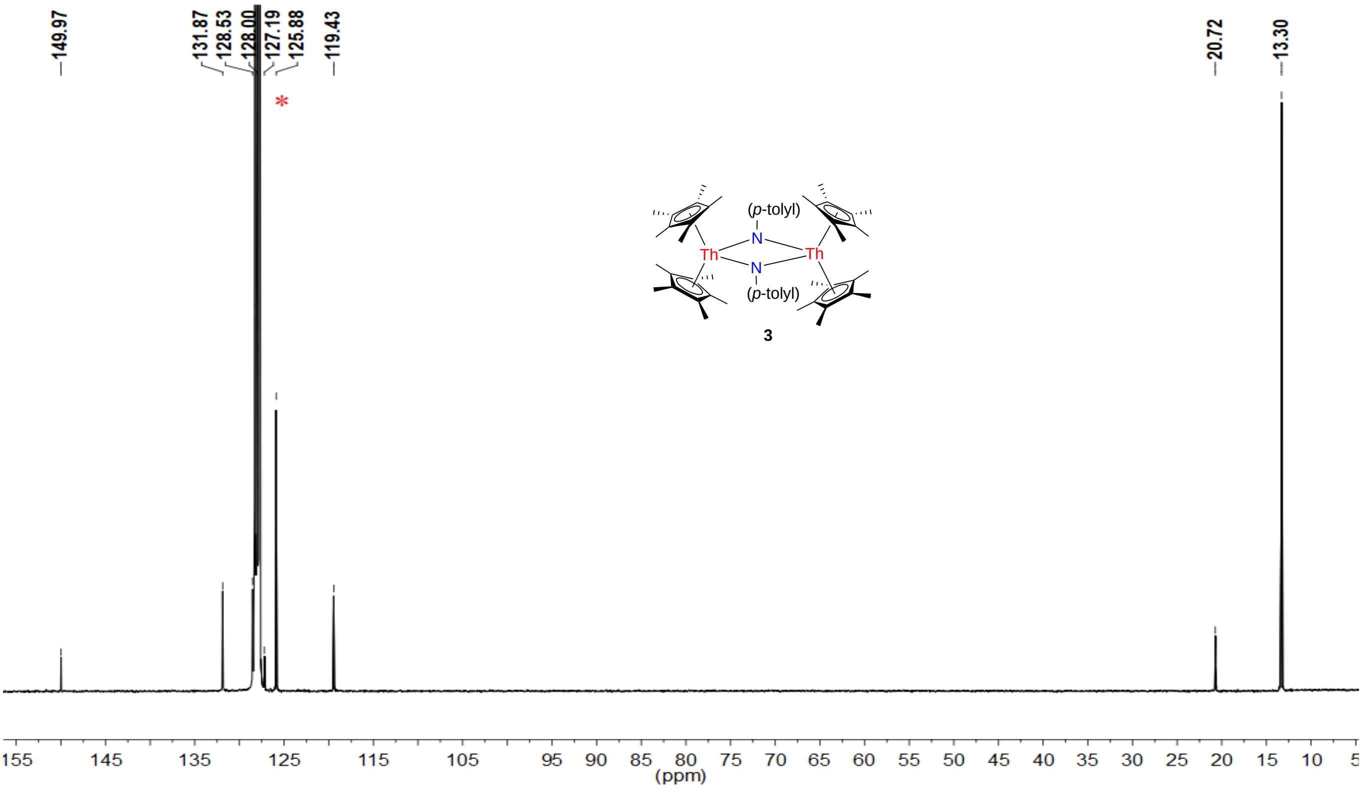

Figure S17. ¹³C{¹H} NMR (C_6D_6; 20 ℃) spectrum for compound $[(\eta^5\text{-}C_5Me_5)_2Th]_2[\mu\text{-}N(p\text{-tolyl})]_2$ C_6H_6 (**3** C_6H_6) (* solvent). ¹³C{¹H} NMR (C_6D_6): δ 150.0 (phenyl *C*), 131.9 (phenyl *C*), 128.5 (C_6H_6), 127.2 (phenyl *C*), 125.9 (phenyl *C*), 119.4 (ring *C*), 20.7 (tolylCH_3), 13.3 (CpCH_3) ppm.

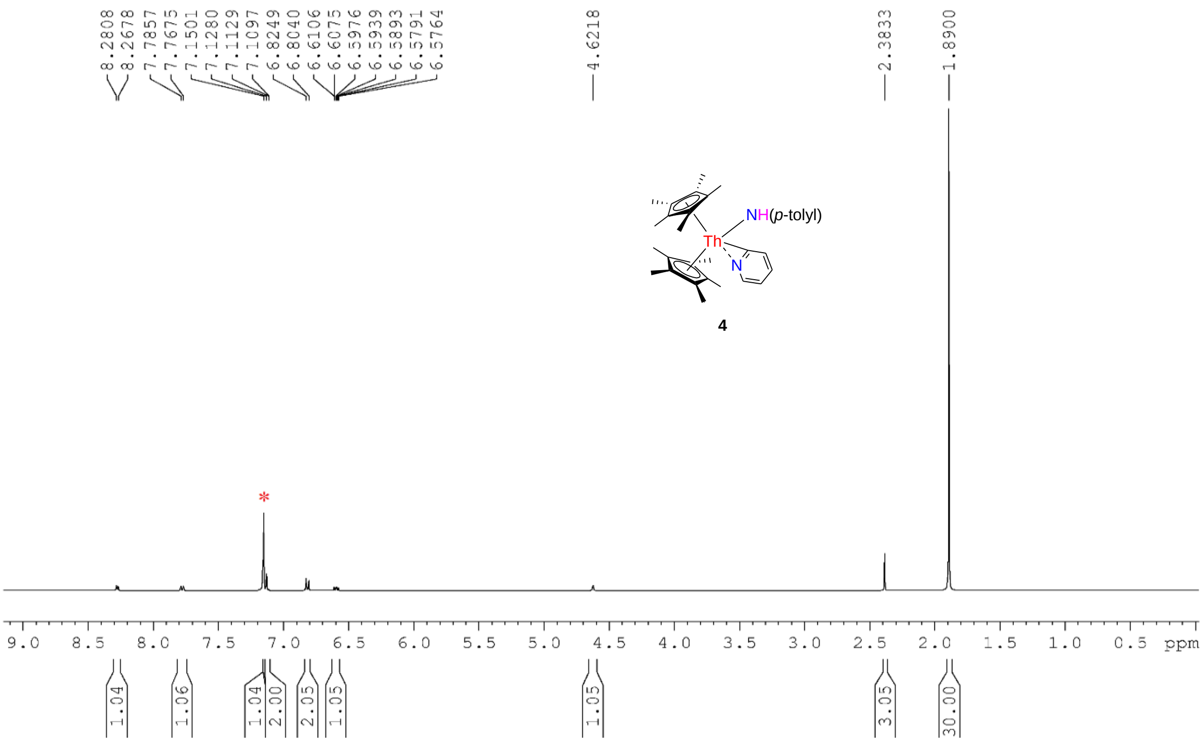

Figure S18. ^{1}H NMR (C_6D_6; 20 ℃) spectrum for compound (η^5-C_5Me_5)$_2$Th[NH(*p*-tolyl)](κ^2-*C*,*N*-C_5H_4N) (**4**) (* solvent). ^{1}H NMR (C_6D_6): δ 8.27 (d, J = 5.2 Hz, 1H, py), 7.78 (d, J = 7.3 Hz, 1H, py), 7.13 (m, 3H, py and phenyl), 6.81 (d, J = 8.4 Hz, 2H, phenyl), 6.59 (m, 1H, py), 4.62 (s, 1H, N*H*), 2.38 (s, 3H, tolylCH_3), 1.89 (s, 30H, CpCH_3) ppm.

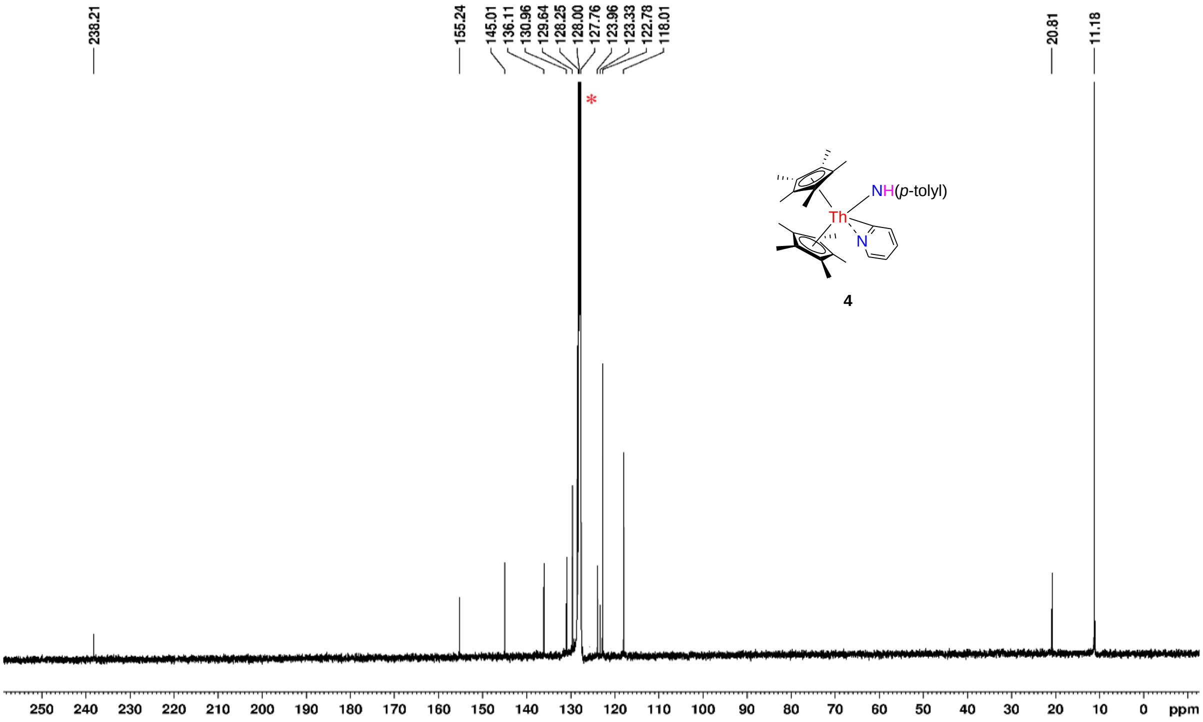

Figure S19. ^{13}C{^{1}H} NMR (C_6D_6; 20 ℃) spectrum for compound (η^5-C_5Me_5)$_2$Th[NH(*p*-tolyl)](κ^2-*C*,*N*-C_5H_4N) (**4**) (* solvent). ^{13}C{^{1}H} NMR (C_6D_6): δ 238.2 (Th*C*), 155.2 (aryl *C*), 145.0 (aryl *C*), 136.1 (aryl *C*), 131.0 (aryl *C*), 129.6 (aryl *C*), 124.0 (aryl *C*), 123.3 (aryl *C*), 122.8 (aryl *C*), 118.0 (ring *C*), 20.8 (tolyl*C*H_3), 11.2 (Cp*C*H_3) ppm.

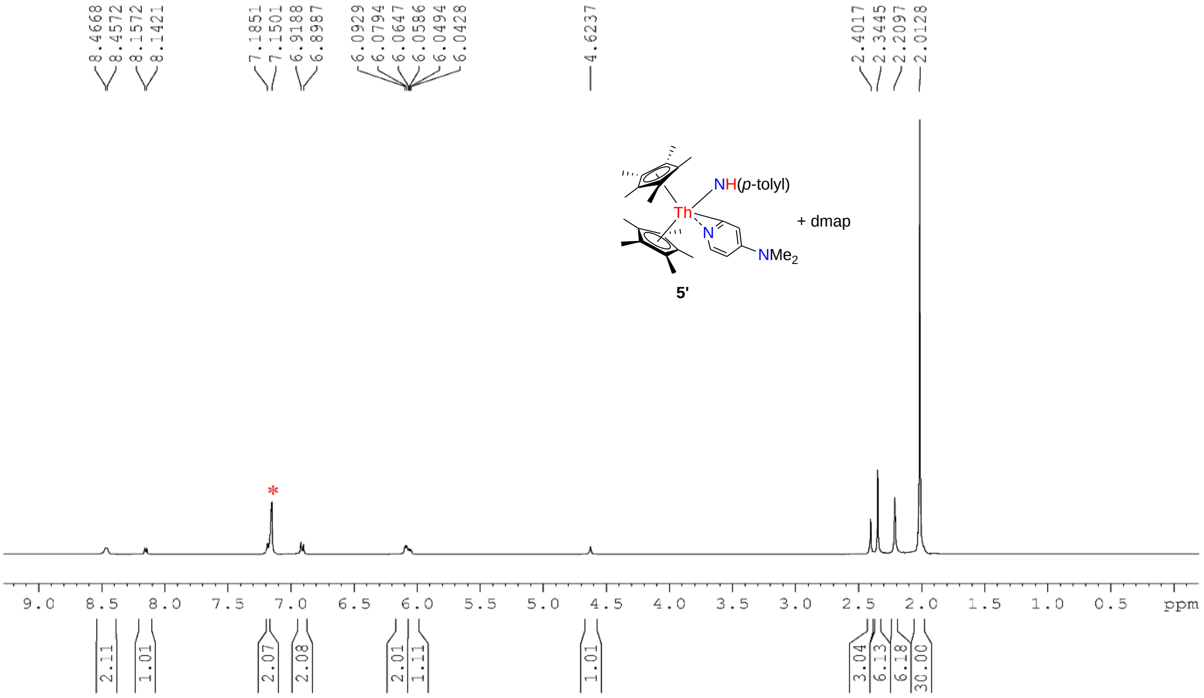

Figure S20. ^{1}H NMR (C_6D_6; 20 °C) spectrum for compound (η^5-C_5Me_5)$_2$Th[NH(*p*-tolyl)][κ^2-*C*,*N*-4-(Me_2N)C_5H_3N] (**5′**)+dmap (* solvent). ^{1}H NMR (C_6D_6): δ 8.46 (d, J = 3.8 Hz, 2H, dmap), 8.15 (d, J = 6.0 Hz, 1H, py), 7.17 (m, 3H, phenyl and py), 6.91 (d, J = 8.0 Hz, 2H, phenyl), 6.09 (d, J = 5.4 Hz, 2H, dmap), 6.05 (m, 1H, py), 4.62 (s, 1H, N*H*), 2.40 (s, 3H, tolylC*H*$_3$), 2.34 (s, 6H, N(C*H*$_3$)$_2$), 2.21 (s, 6H, (C*H*$_3$)$_2$N, dmap), 2.01 (s, 30H, CpC*H*$_3$) ppm.

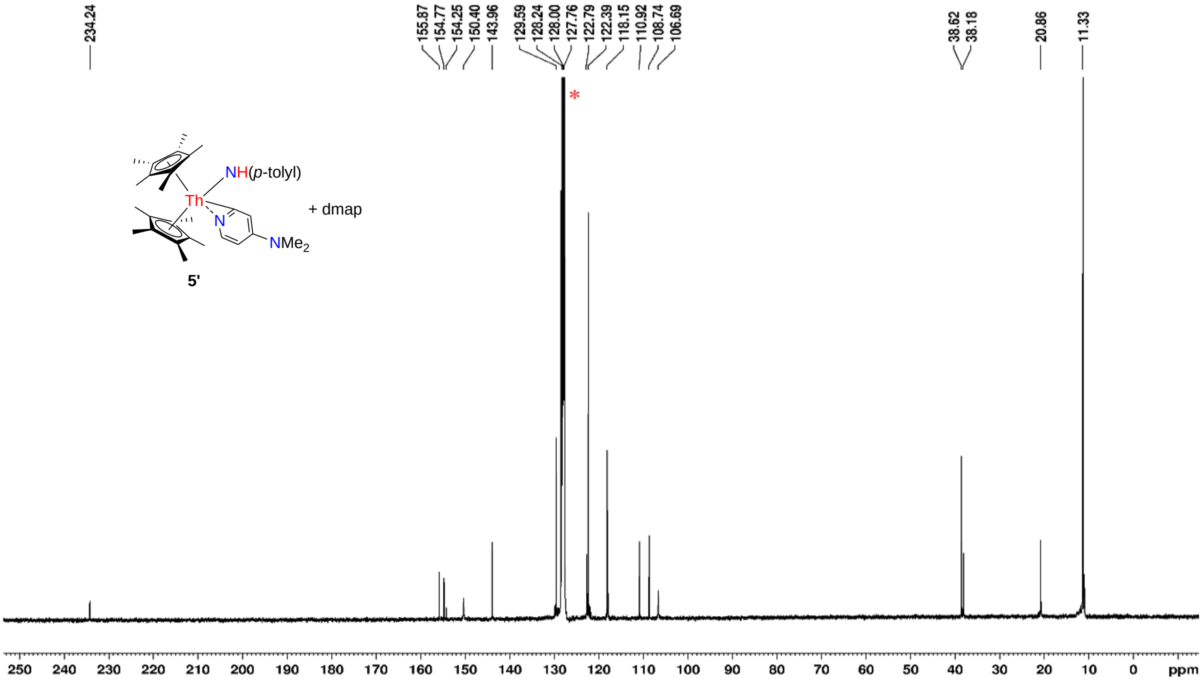

Figure S21. $^{13}C\{^{1}H\}$ NMR (C_6D_6; 20 °C) spectrum for compound (η^5-C_5Me_5)$_2$Th[NH(*p*-tolyl)][κ^2-*C*,*N*-4-(Me_2N)C_5H_3N] (**5′**)+dmap (* solvent). $^{13}C\{^{1}H\}$ NMR (C_6D_6): δ 234.2 (Th*C*), 155.9 (aryl *C*), 154.8 (aryl *C*), 154.3 (dmap *C*), 150.4 (dmap *C*), 143.9 (aryl *C*), 129.6 (aryl *C*), 122.8 (aryl *C*), 122.4 (ring *C*), 118.2 (aryl *C*), 110.9 (aryl *C*), 108.7 (aryl *C*), 106.7 (dmap *C*), 38.6 (N*C*H$_3$, dmap), 38.2 (N*C*H$_3$), 20.9 (tolyl*C*H$_3$), 11.3 (Cp*C*H$_3$) ppm.

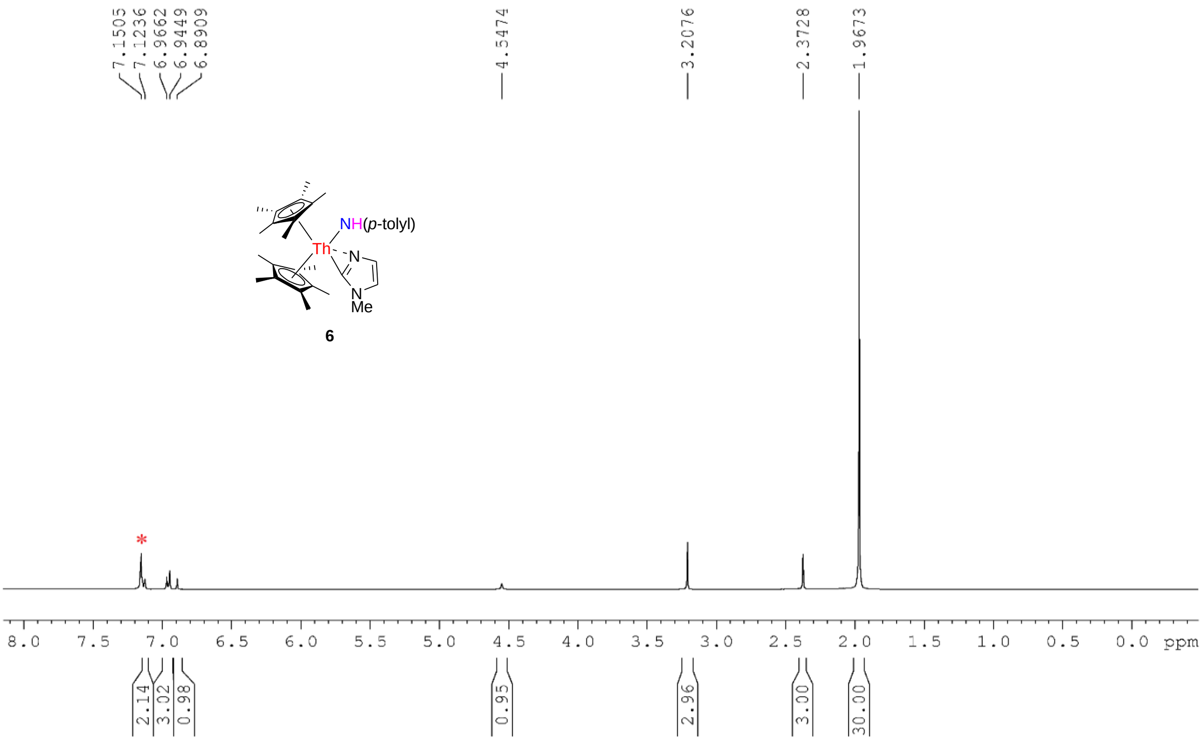

Figure S22. ^{1}H NMR (C_6D_6; 20 °C) spectrum for compound (η^5-C_5Me_5)$_2$Th[NH(*p*-tolyl)](κ^2-*C*,*N*-1-Me$C_3H_2N_2$) (**6**) (* solvent). ^{1}H NMR (C_6D_6): δ 7.13 (d, J = 8.0 Hz, 2H, aryl), 6.95 (m, 3H, aryl), 6.89 (s, 1H, aryl), 4.55 (s, 1H, N*H*), 3.21 (s, 3H, NC*H*$_3$), 2.37 (s, 3H, tolylC*H*$_3$), 1.97 (s, 30H, CpC*H*$_3$) ppm.

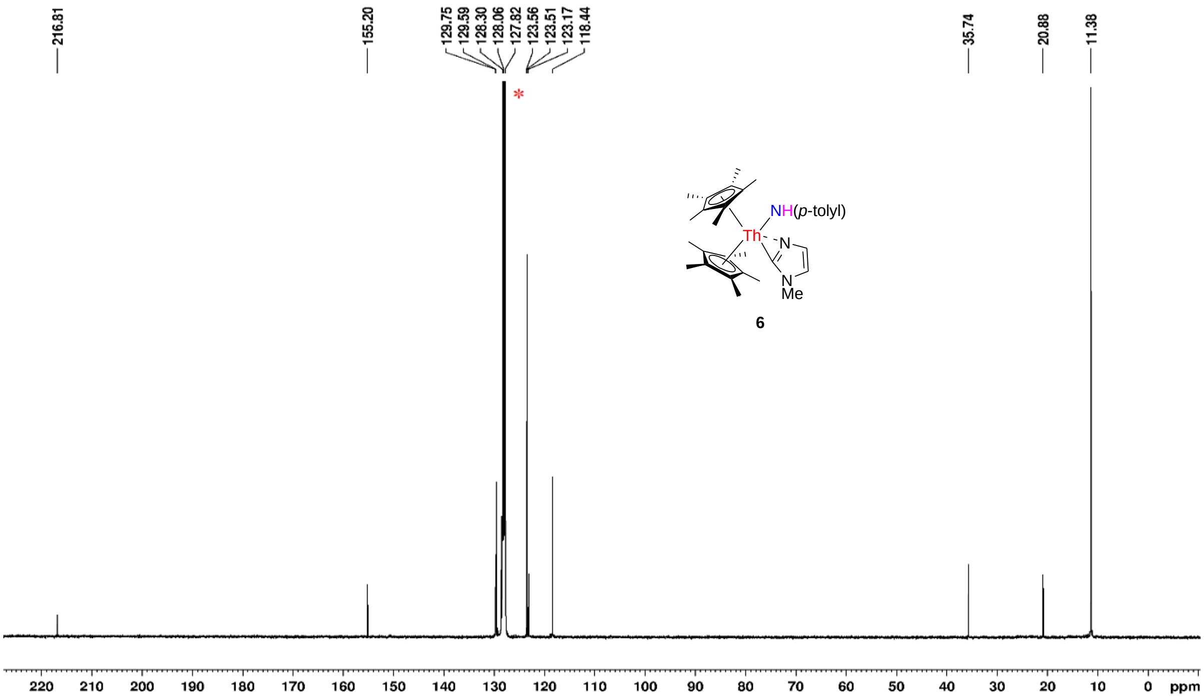

Figure S23. ^{13}C{^{1}H} NMR (C_6D_6; 20 °C) spectrum for compound (η^5-C_5Me_5)$_2$Th[NH(*p*-tolyl)](κ^2-*C*,*N*-1-Me$C_3H_2N_2$) (**6**) (* solvent). ^{13}C{^{1}H} NMR (C_6D_6): δ 216.8 (Th*C*), 155.2 (aryl *C*), 129.8 (aryl *C*), 129.6 (aryl *C*), 123.6 (aryl *C*), 123.5 (aryl *C*), 123.2 (aryl *C*), 118.4 (ring *C*), 35.7 (N*C*H$_3$), 20.9 (tolyl*C*H$_3$), 11.4 (Cp*C*H$_3$) ppm.

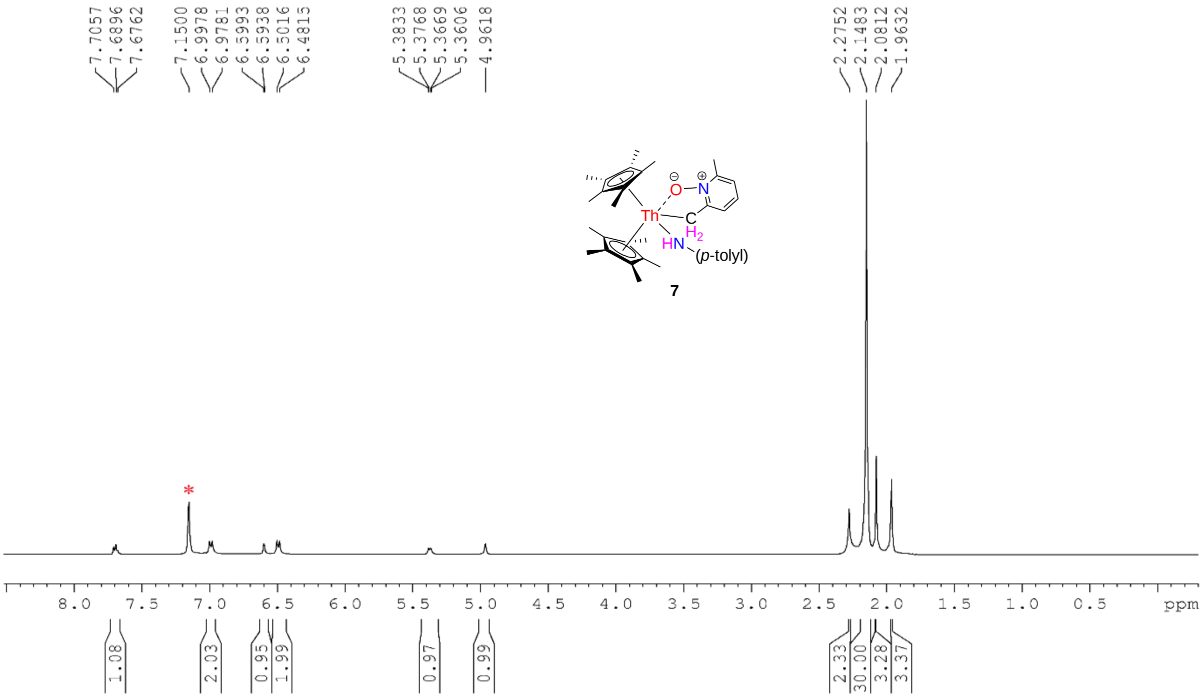

Figure S24. ^{1}H NMR (C_6D_6; 20 ℃) spectrum for compound (η^5-C_5Me_5)$_2$Th[NH(*p*-tolyl)](κ^2-*C*,*O*-2-CH_2-6-MeC_5H_3NO) (**7**) (* solvent). ^{1}H NMR (C_6D_6): δ 7.69 (m, 1H, py), 6.99 (d, J = 8.0 Hz, 2H, phenyl), 6.59 (d, J = 2.2 Hz, 1H, py), 6.49 (d, J = 8.0 Hz, 2H, phenyl), 5.37 (dd, J = 6.6 Hz and 2.6 Hz, 1H, py), 4.96 (s, 1H, N*H*), 2.28 (s, 2H, CH_2), 2.15 (s, 30H, CpCH_3), 2.08 (s, 3H, CH_3), 1.96 (s, 3H, CH_3) ppm.

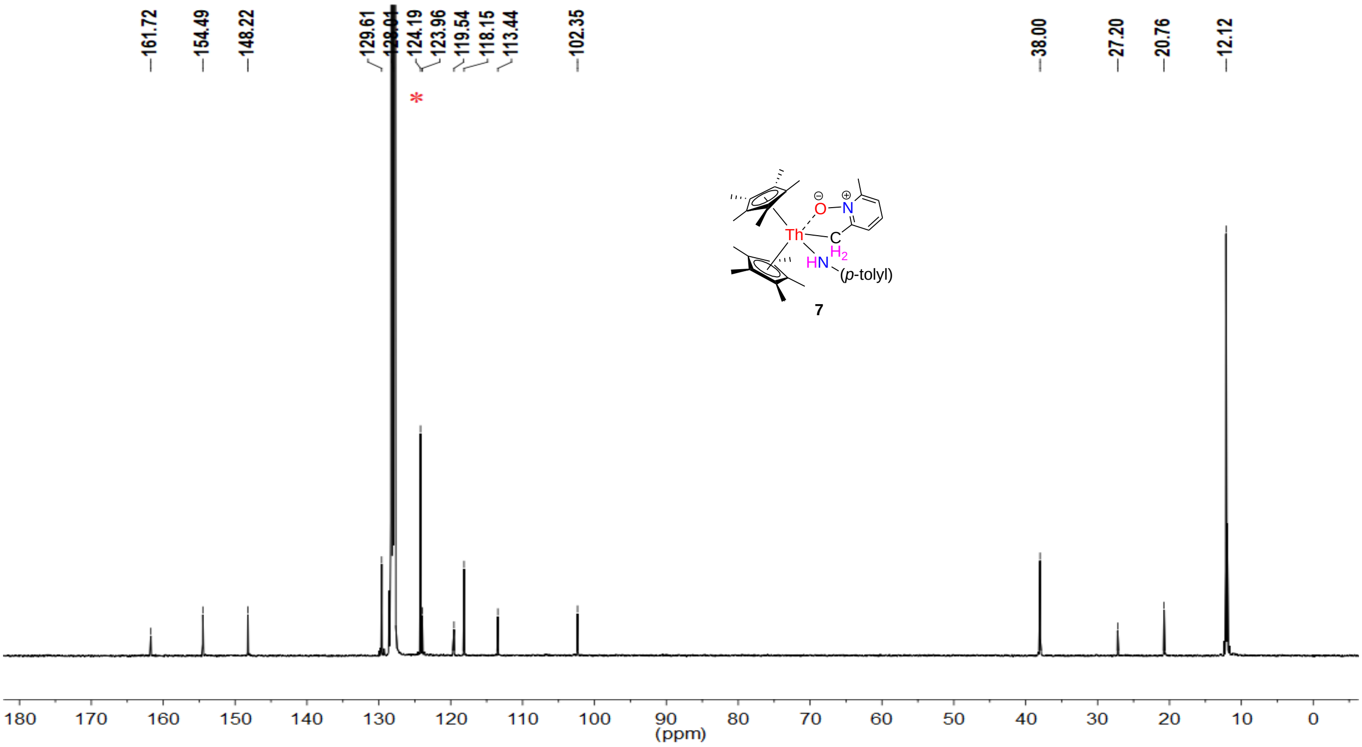

Figure S25. ^{13}C{^{1}H} NMR (C_6D_6; 20 ℃) spectrum for compound (η^5-C_5Me_5)$_2$Th[NH(*p*-tolyl)](κ^2-*C*,*O*-2-CH_2-6-MeC_5H_3NO) (**7**) (* solvent). ^{13}C{^{1}H} NMR (C_6D_6): δ 161.7 (aryl *C*), 154.5 (aryl *C*), 148.2 (aryl *C*), 129.6 (aryl *C*), 124.2 (aryl *C*), 124.0 (aryl *C*), 119.5 (aryl *C*), 118.1 (ring *C*), 113.4 (aryl *C*), 102.4 (aryl *C*), 38.0 (Th*C*H_2), 27.2 (py*C*H_3), 20.8 (tolyl*C*H_3), 12.1 (Cp*C*H_3) ppm.

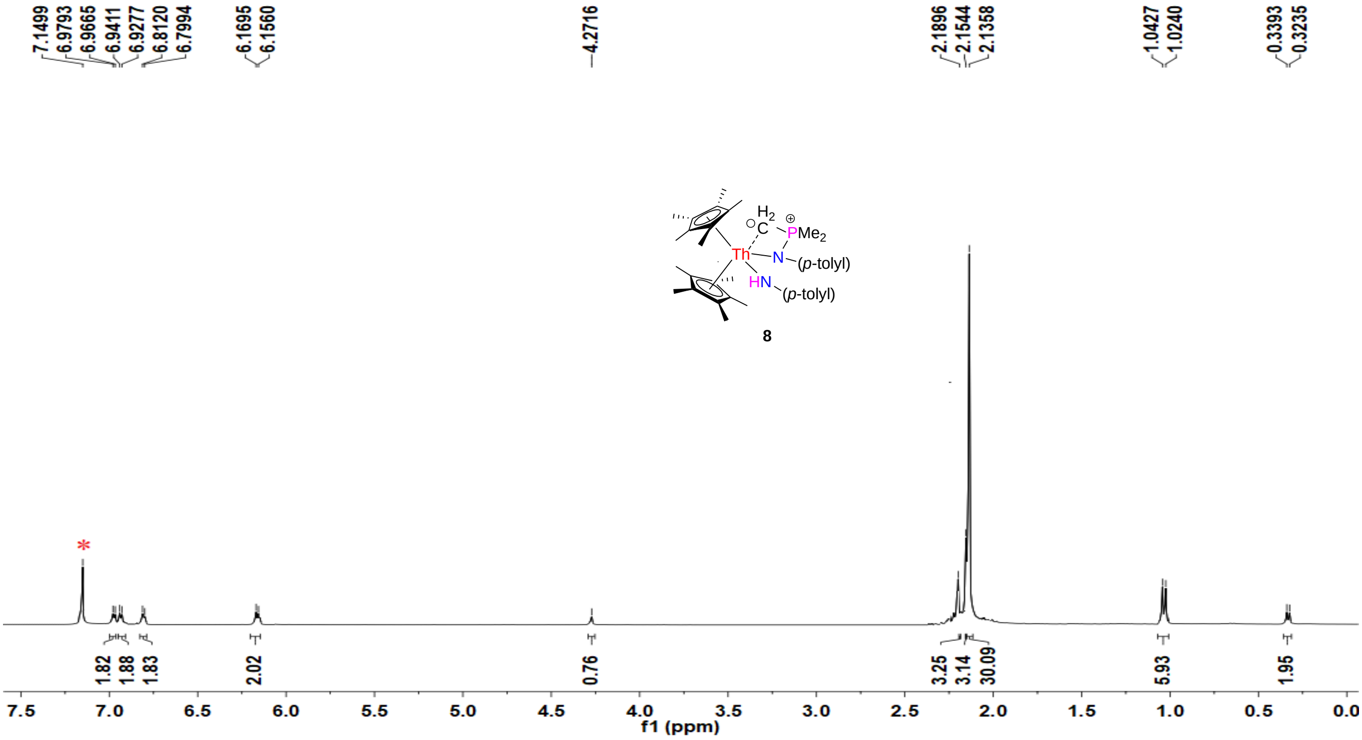

Figure S26. ^{1}H NMR (C_6D_6; 20 °C) spectrum for compound (η^5-C_5Me_5)$_2$Th[NH(*p*-tolyl)][κ^2-*C*,*N*-N(*p*-tolyl)P(Me$_2$)CH$_2$] (**8**) (* solvent). ^{1}H NMR (C_6D_6): δ 6.97 (d, *J* = 7.6 Hz, 2H, phenyl), 6.93 (d, *J* = 8.0 Hz, 2H, phenyl), 6.81 (d, *J* = 7.6 Hz, 2H, phenyl), 6.16 (d, *J* = 8.0 Hz, 2H, phenyl), 4.27 (s, 1H, N*H*), 2.19 (s, 3H, tolylC*H*$_3$), 2.15 (s, 3H, tolylC*H*$_3$), 2.14 (s, 30H, CpC*H*$_3$), 1.03 (d, $J_{P\text{-}H}$ = 11.2 Hz, 6H, P(C*H*$_3$)$_2$), 0.33 (d, $J_{P\text{-}H}$ = 9.5 Hz, 2H, C*H*$_2$) ppm.

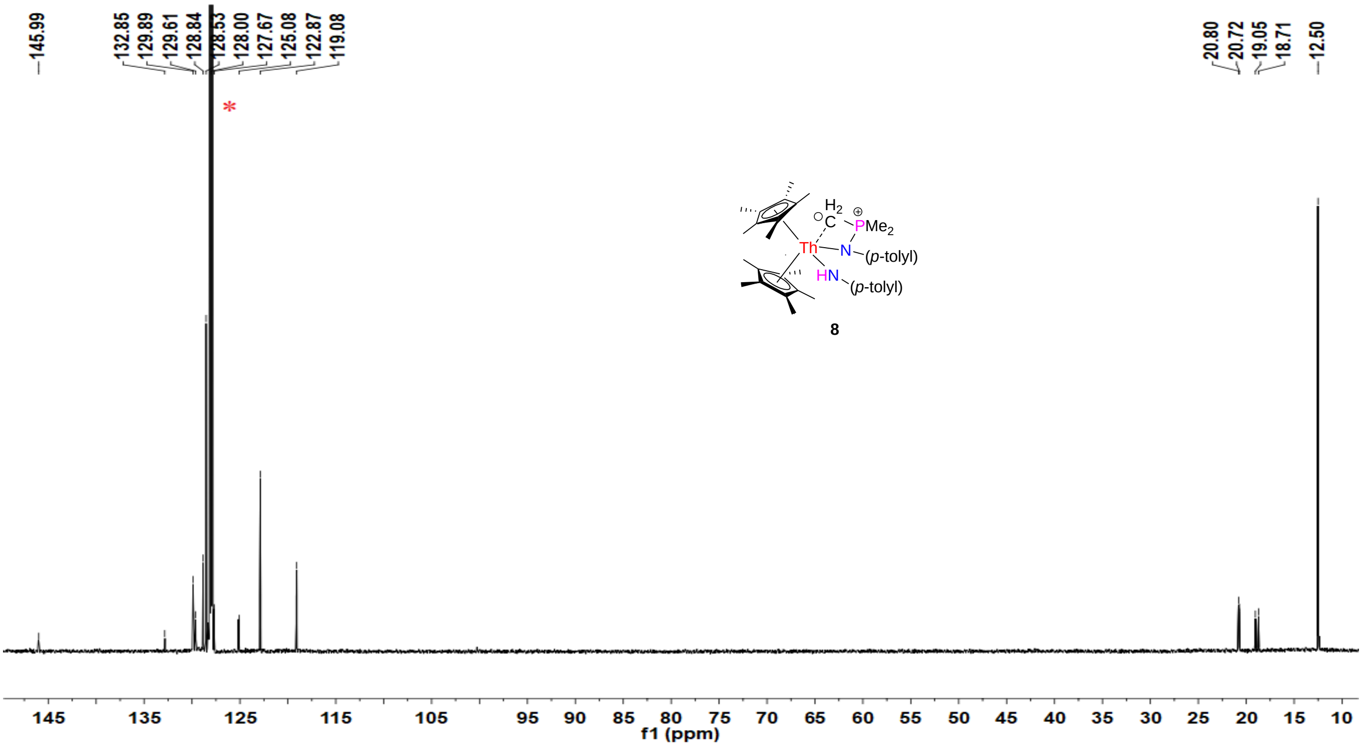

Figure S27. ^{13}C{^{1}H} NMR (C_6D_6; 20 °C) spectrum for compound (η^5-C_5Me_5)$_2$Th[NH(*p*-tolyl)][κ^2-*C*,*N*-N(*p*-tolyl)P(Me$_2$)CH$_2$] (**8**) (* solvent). ^{13}C{^{1}H} NMR (C_6D_6): δ 146.0 (P*C*H$_2$), 132.9 (phenyl *C*), 129.9 (phenyl *C*), 129.6 (phenyl *C*), 128.8 (phenyl *C*), 128.5 (phenyl *C*), 127.7 (phenyl *C*), 125.1 (ring *C*), 122.9 (phenyl *C*), 119.1 (phenyl *C*), 20.7 (d, $J_{P\text{-}C}$ = 46.2 Hz, P(*C*H$_3$)$_2$), 19.1 (tolyl*C*H$_3$), 18.7 (tolyl*C*H$_3$), 12.5 (Cp*C*H$_3$) ppm.

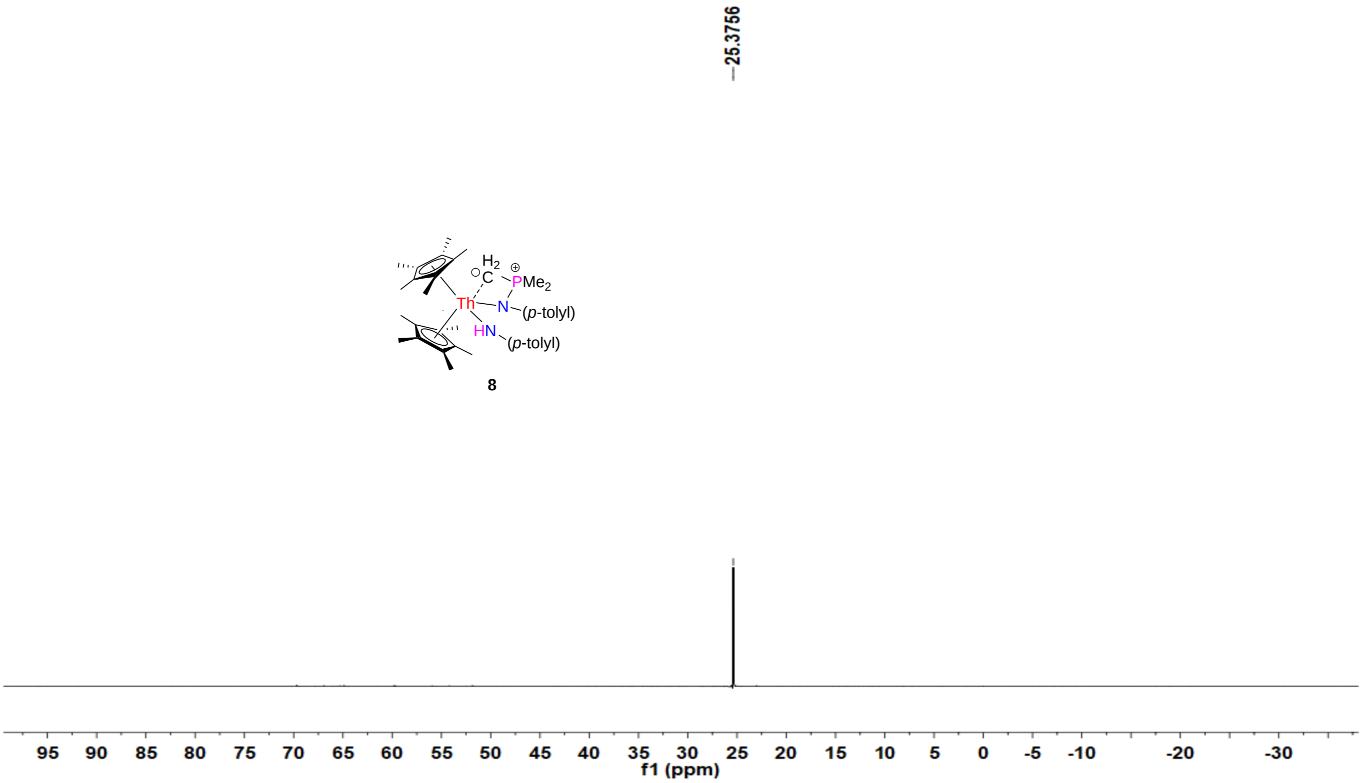

Figure S28. $^{31}P\{^{1}H\}$ NMR (C_6D_6; 20 °C) spectrum for compound (η^5-C_5Me_5)$_2$Th[NH(*p*-tolyl)][κ^2-*C*,*N*-N(*p*-tolyl)P(Me_2)CH_2] (**8**). $^{31}P\{^{1}H\}$ NMR (C_6D_6): δ 25.4 ppm.

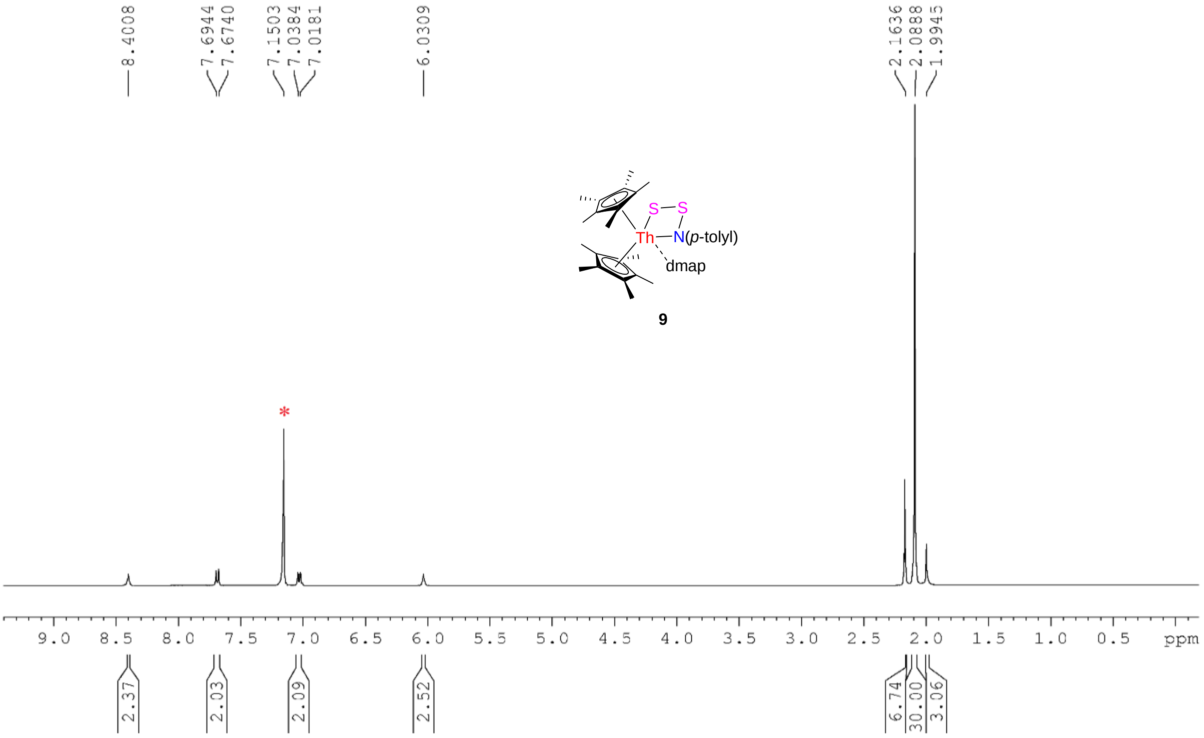

Figure S29. ^{1}H NMR (C_6D_6; 20 °C) spectrum for compound (η^5-C_5Me_5)$_2$Th[N(*p*-tolyl)SS](dmap) (**9**) (* solvent). ^{1}H NMR (C_6D_6): δ 8.40 (s, 2H, py), 7.68 (d, J = 8.2 Hz, 2H, phenyl), 7.03 (d, J = 8.2 Hz, 2H, phenyl), 6.03 (s, 2H, py), 2.16 (s, 6H, N(CH_3)$_2$), 2.09 (s, 30H, CpCH_3), 1.99 (s, 3H, tolylCH_3) ppm.

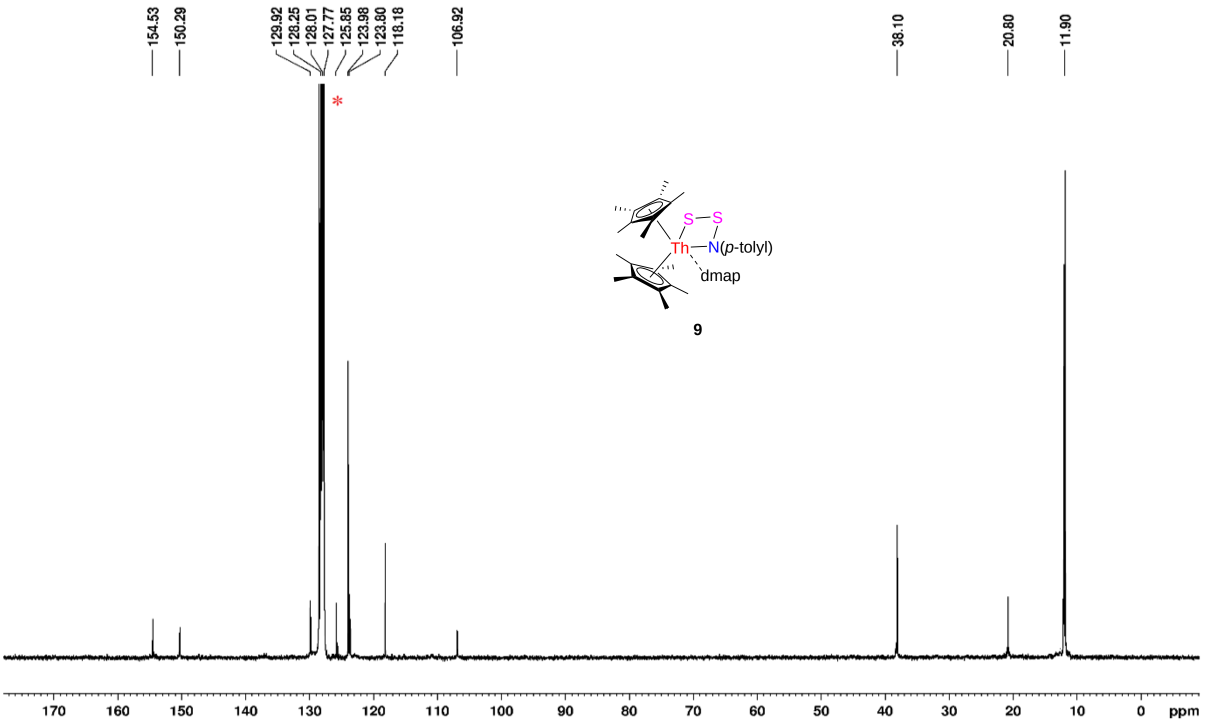

Figure S30. $^{13}C\{^{1}H\}$ NMR (C_6D_6; 20 ℃) spectrum for compound (η^5-C_5Me_5)$_2$Th[N(*p*-tolyl)SS](dmap) (**9**) (* solvent). $^{13}C\{^{1}H\}$ NMR (C_6D_6): δ 154.5 (py *C*), 150.3 (py *C*), 129.9 (phenyl *C*), 125.9 (phenyl *C*), 124.0 (phenyl *C*), 123.8 (phenyl *C*), 118.2 (ring *C*), 106.9 (py *C*), 38.1 (N*C*H$_3$), 20.8 (tolyl*C*H$_3$), 11.9 (Cp*C*H$_3$) ppm.

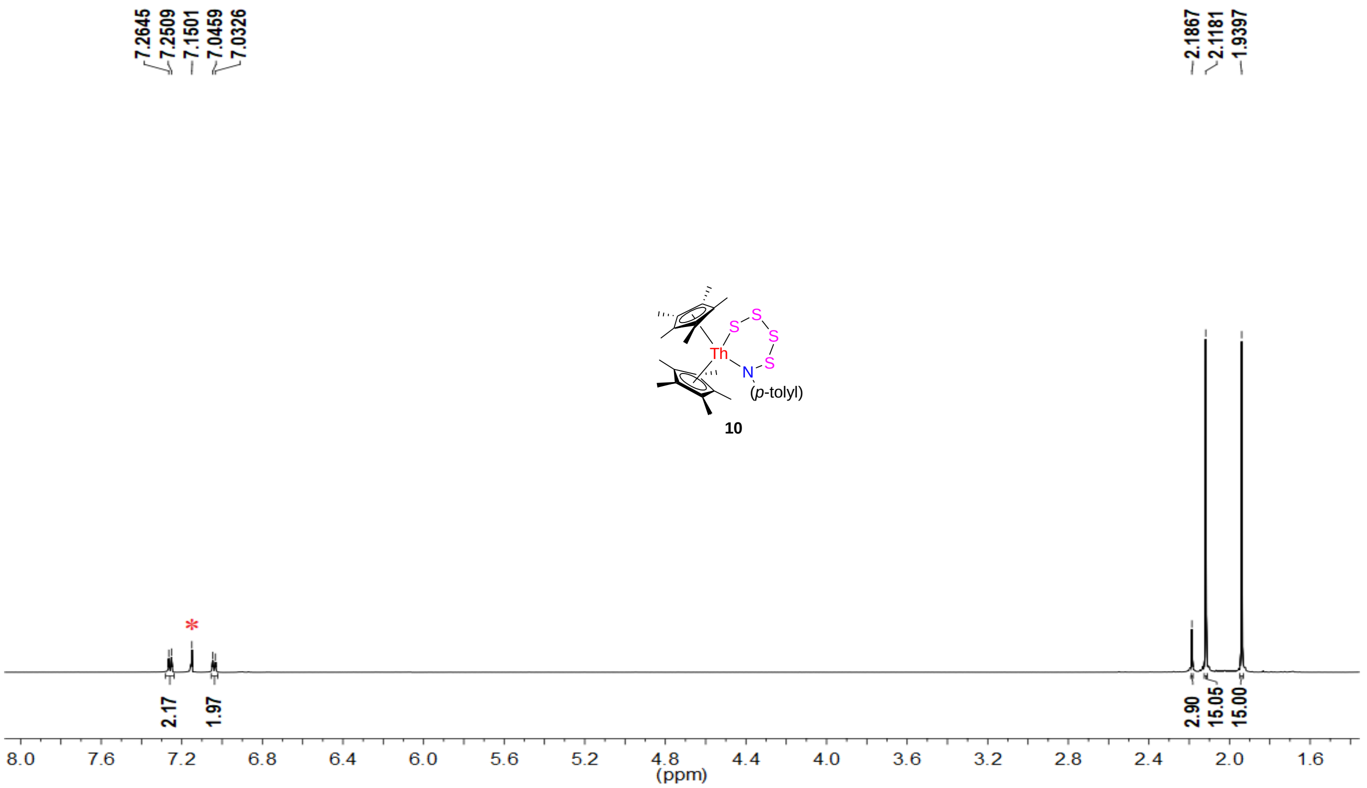

Figure S31. ^{1}H NMR (C_6D_6; 20 ℃) spectrum for compound (η^5-C_5Me_5)$_2$Th[N(*p*-tolyl)S$_4$] (**10**) (* solvent). ^{1}H NMR (C_6D_6): δ 7.26 (d, J = 8.2 Hz, 2H, phenyl), 7.04 (d, J = 8.2 Hz, 2H, phenyl), 2.19 (s, 3H, tolylCH_3), 2.12 (s, 15H, CpCH_3), 1.94 (s, 15H, CpCH_3) ppm.

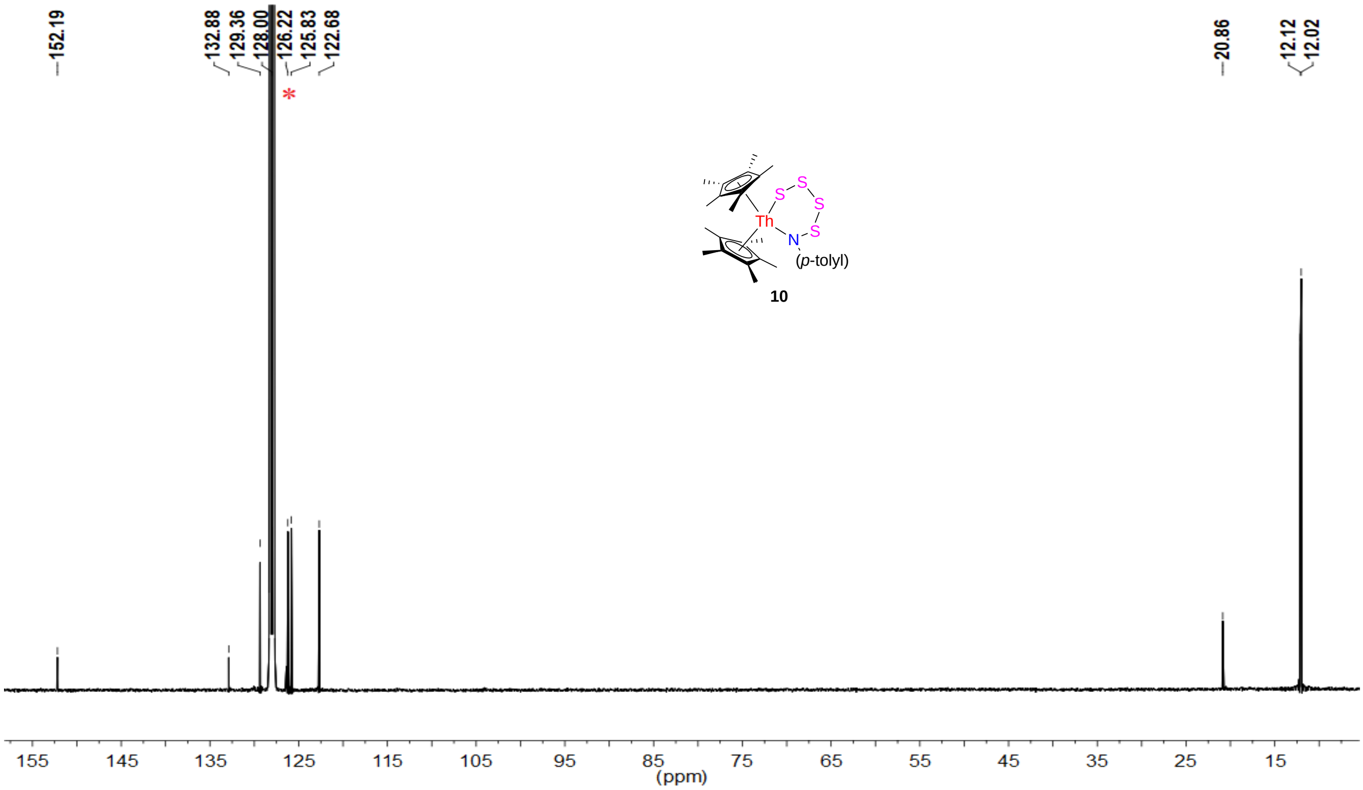

Figure S32. $^{13}C\{^{1}H\}$ NMR (C_6D_6; 20 ℃) spectrum for compound (η^5-C_5Me_5)$_2$Th[N(*p*-tolyl)S_4] (**10**) (* solvent). $^{13}C\{^{1}H\}$ NMR (C_6D_6): δ 152.2 (phenyl *C*), 132.9 (phenyl *C*), 129.4 (phenyl *C*), 126.2 (ring *C*), 125.8 (ring *C*), 122.7 (phenyl *C*), 20.9 (tolyl*C*H_3), 12.1 (Cp*C*H_3), 12.0 (Cp*C*H_3) ppm.

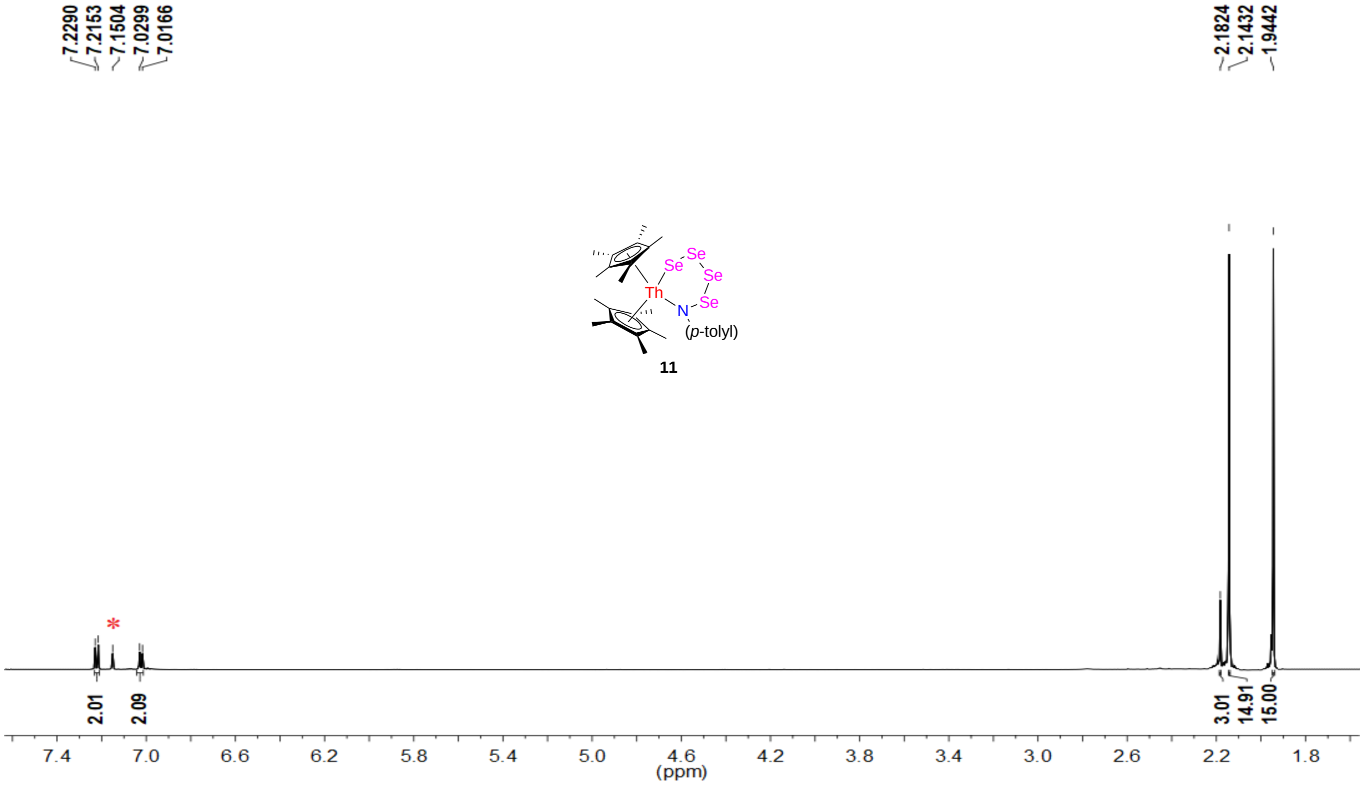

Figure S33. ^{1}H NMR (C_6D_6; 20 ℃) spectrum for compound (η^5-C_5Me_5)$_2$Th[N(*p*-tolyl)Se_4] (**11**) (* solvent). ^{1}H NMR (C_6D_6): δ 7.22 (d, J = 8.2 Hz, 2H, phenyl), 7.02 (d, J = 8.2 Hz, 2H, phenyl), 2.18 (s, 3H, tolylCH_3), 2.14 (s, 15H, CpCH_3), 1.94 (s, 15H, CpCH_3) ppm.

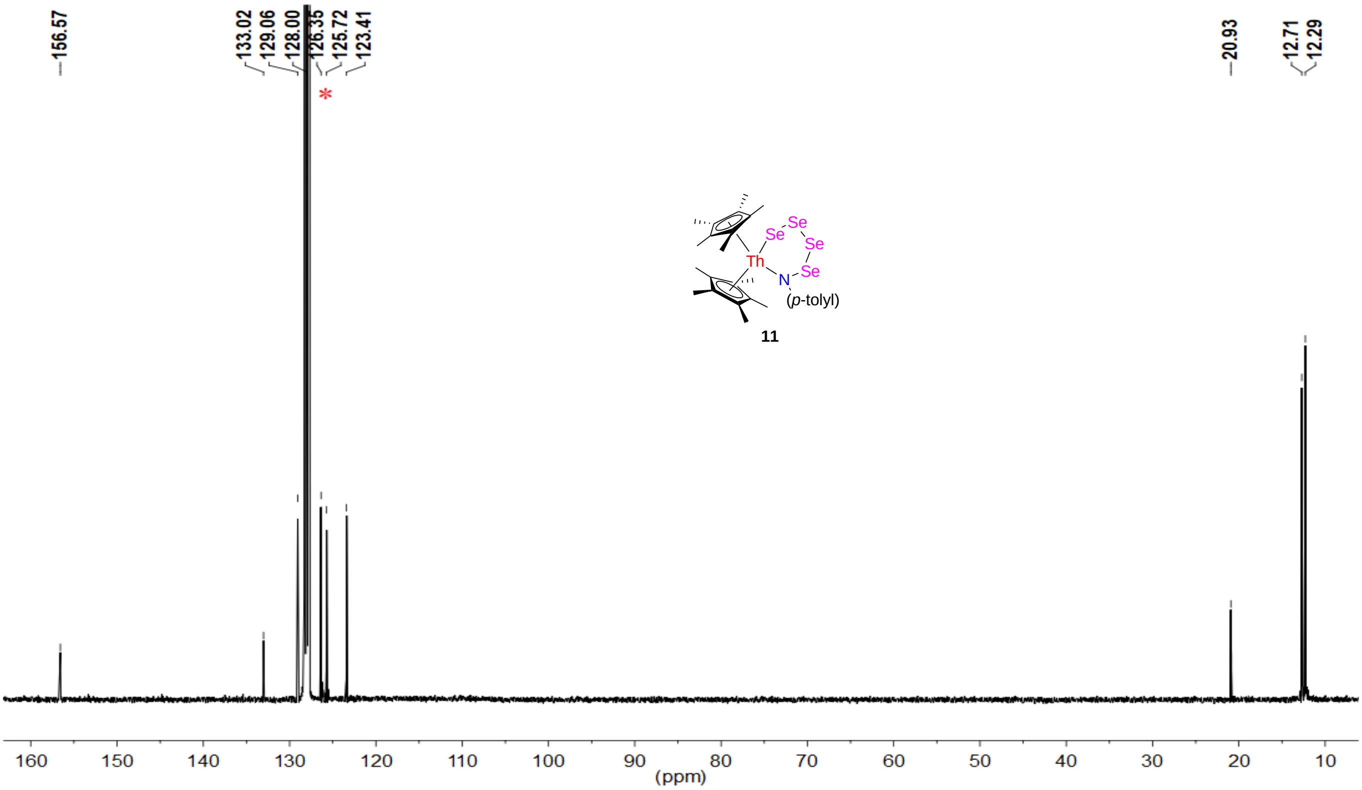

Figure S34. $^{13}C\{^1H\}$ NMR (C_6D_6; 20 °C) spectrum for compound (η^5-C_5Me_5)$_2$Th[N(*p*-tolyl)Se_4] (**11**) (* solvent). $^{13}C\{^1H\}$ NMR (C_6D_6): δ 156.6 (phenyl *C*), 133.0 (phenyl *C*), 129.1 (phenyl *C*), 126.4 (ring *C*), 125.7 (ring *C*), 123.4 (phenyl *C*), 20.9 (tolylCH_3), 12.7 (CpCH_3), 12.3 (CpCH_3) ppm.

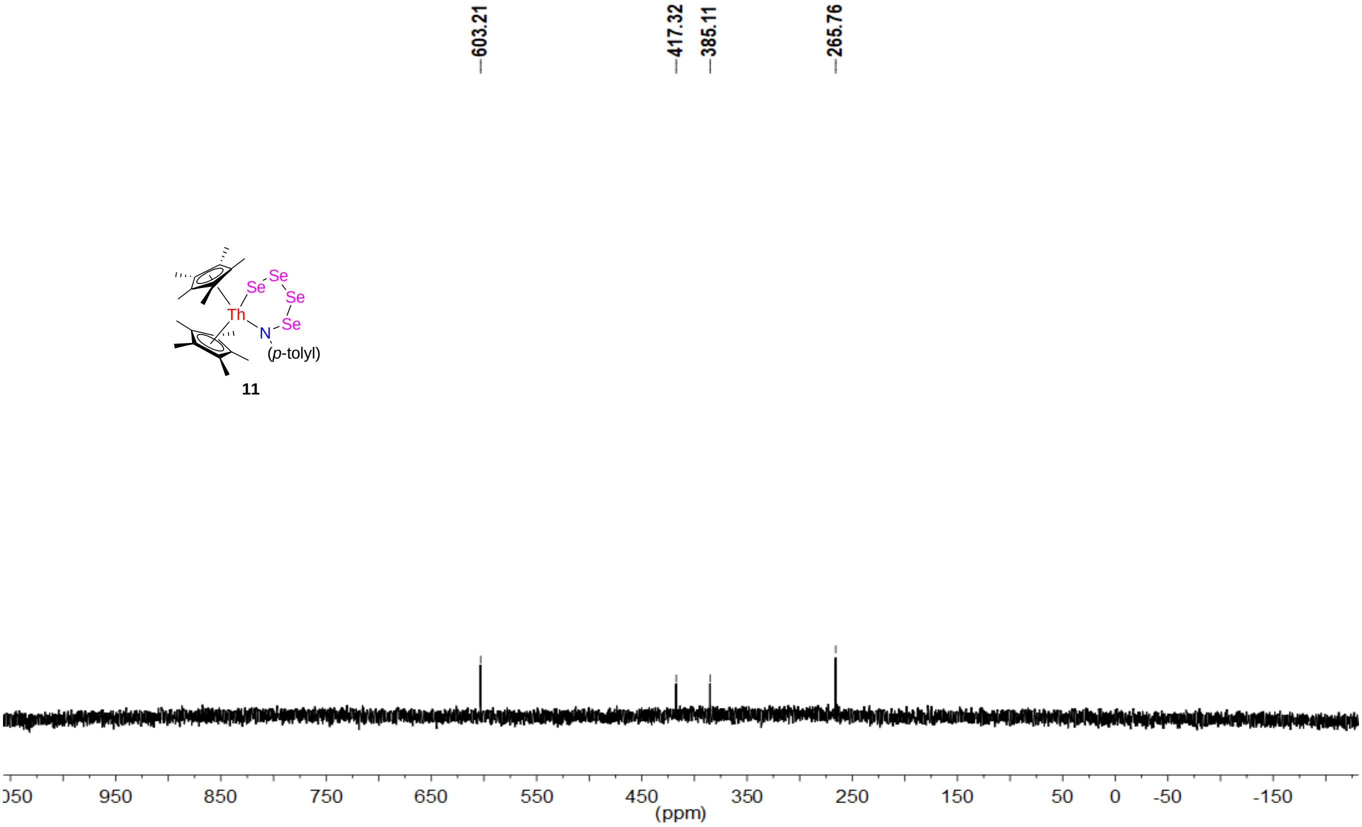

Figure S35. $^{77}Se\{^1H\}$ NMR (C_6D_6; 20 °C) spectrum for compound (η^5-C_5Me_5)$_2$Th[N(*p*-tolyl)Se_4] (**11**). $^{77}Se\{^1H\}$ NMR (C_6D_6): δ 603.2, 417.3, 385.1, 265.8 ppm.

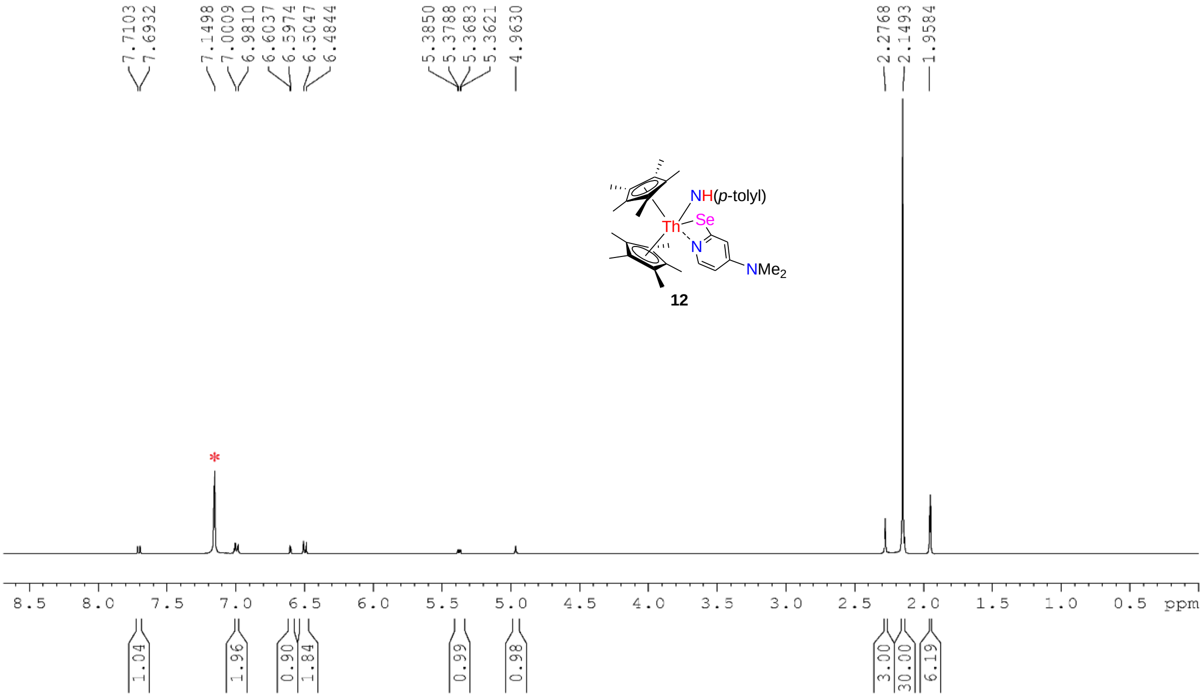

Figure S36. ^{1}H NMR (C_6D_6; 20 ℃) spectrum for compound (η^5-C_5Me_5)$_2$Th[NH(*p*-tolyl)][κ^2-*N*,*Se*-2-Se-4-(Me_2N)C_5H_3N] (**12**) (* solvent). ^{1}H NMR (C_6D_6): δ 7.70 (d, J = 6.8 Hz, 1H, py), 6.99 (d, J = 8.0 Hz, 2H, phenyl), 6.60 (d, J = 2.5 Hz, 1H, py), 6.49 (d, J = 8.0 Hz, 2H, phenyl), 5.37 (dd, J = 6.7 Hz and 2.5Hz, 1H, py), 4.96 (s, 1H, N*H*), 2.28 (s, 3H, tolylC*H*$_3$), 2.15 (s, 30H, CpC*H*$_3$), 1.96 (s, 6H, N(C*H*$_3$)$_2$) ppm.

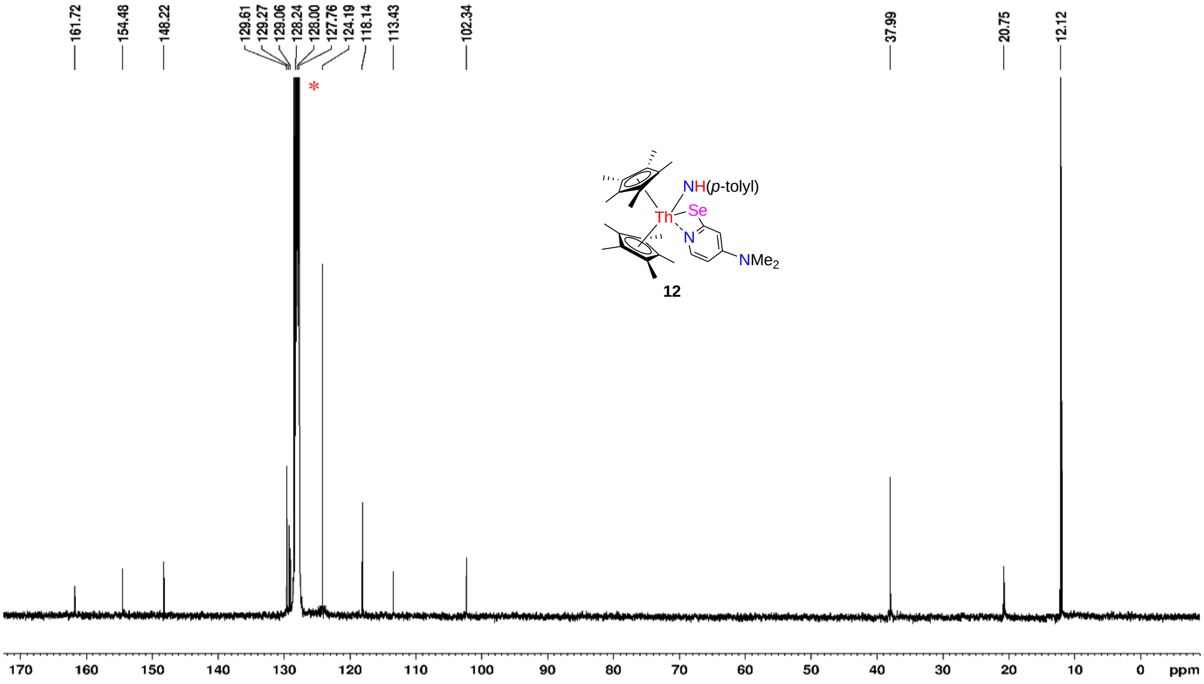

Figure S37. ^{13}C{^{1}H} NMR (C_6D_6; 20 ℃) spectrum for compound (η^5-C_5Me_5)$_2$Th[NH(*p*-tolyl)][κ^2-*N*,*Se*-2-Se-4-(Me_2N)C_5H_3N] (**12**) (* solvent). ^{13}C{^{1}H} NMR (C_6D_6): δ 161.7 (py *C*), 154.5 (py *C*), 148.2 (py *C*), 129.6 (phenyl *C*), 129.3 (phenyl *C*), 129.1 (phenyl *C*), 124.2 (phenyl *C*), 118.1 (ring *C*), 113.4 (py *C*), 102.3 (py *C*), 38.0 (N*C*H$_3$), 20.8 (tolyl*C*H$_3$), 12.1 (Cp*C*H$_3$) ppm.

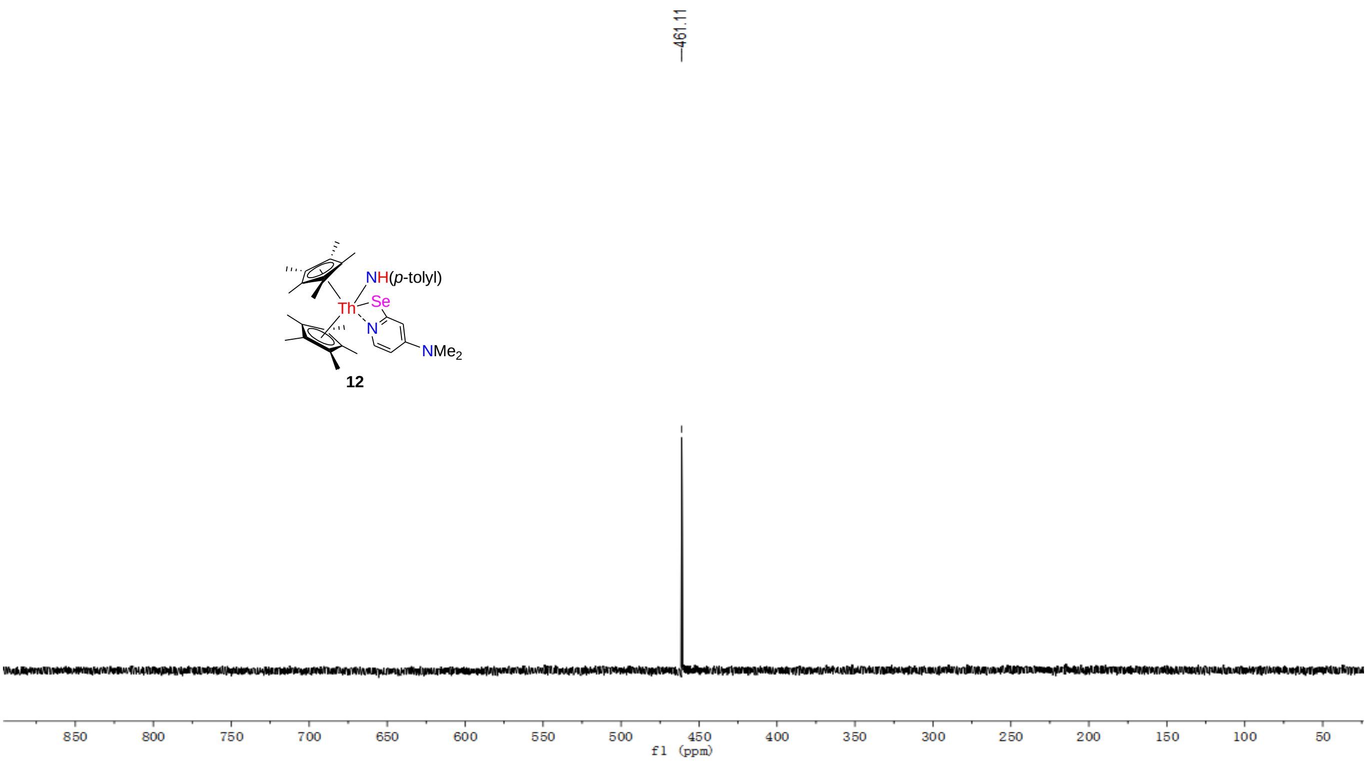

Figure S38. ^{77}Se{^{1}H} NMR (C_6D_6; 20 ℃) spectrum for compound (η^5-C_5Me_5)$_2$Th[NH(*p*-tolyl)][κ^2-*N,Se*-2-Se-4-(Me_2N)C_5H_3N] (**12**). ^{77}Se{^{1}H} NMR (C_6D_6): δ 461.1 ppm.

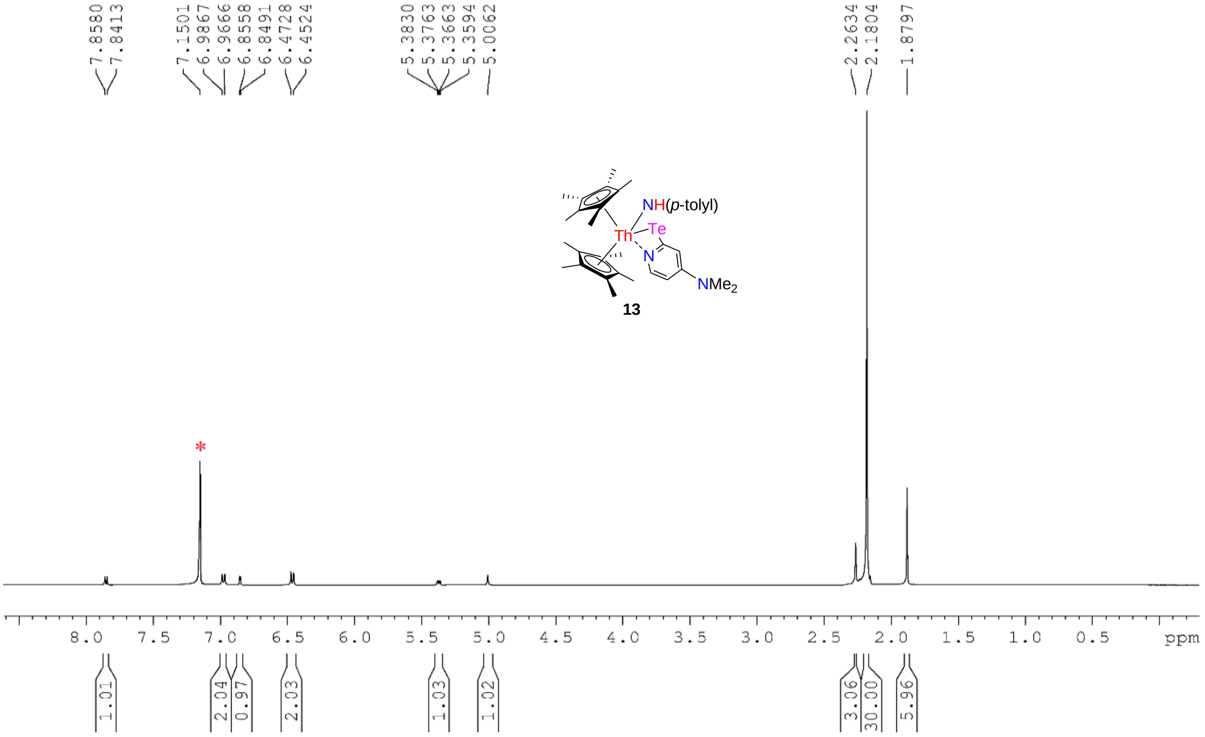

Figure S39. ^{1}H NMR (C_6D_6; 20 ℃) spectrum for compound (η^5-C_5Me_5)$_2$Th[NH(*p*-tolyl)][κ^2-*N,Te*-2-Te-4-(Me_2N)C_5H_3N] (**13**) (* solvent). ^{1}H NMR (C_6D_6): δ 7.85 (d, J = 6.7 Hz, 1H, py), 6.98 (d, J = 8.0 Hz, 2H, phenyl), 6.85 (d, J = 2.7 Hz, 1H, py), 6.46 (d, J = 8.0 Hz, 2H, phenyl), 5.37 (dd, J = 6.7 Hz and 2.7Hz, 1H, py), 5.01 (s, 1H, N*H*), 2.26 (s, 3H, tolylC*H*$_3$), 2.18 (s, 30H, CpC*H*$_3$), 1.88 (s, 6H, N(C*H*$_3$)$_2$) ppm.

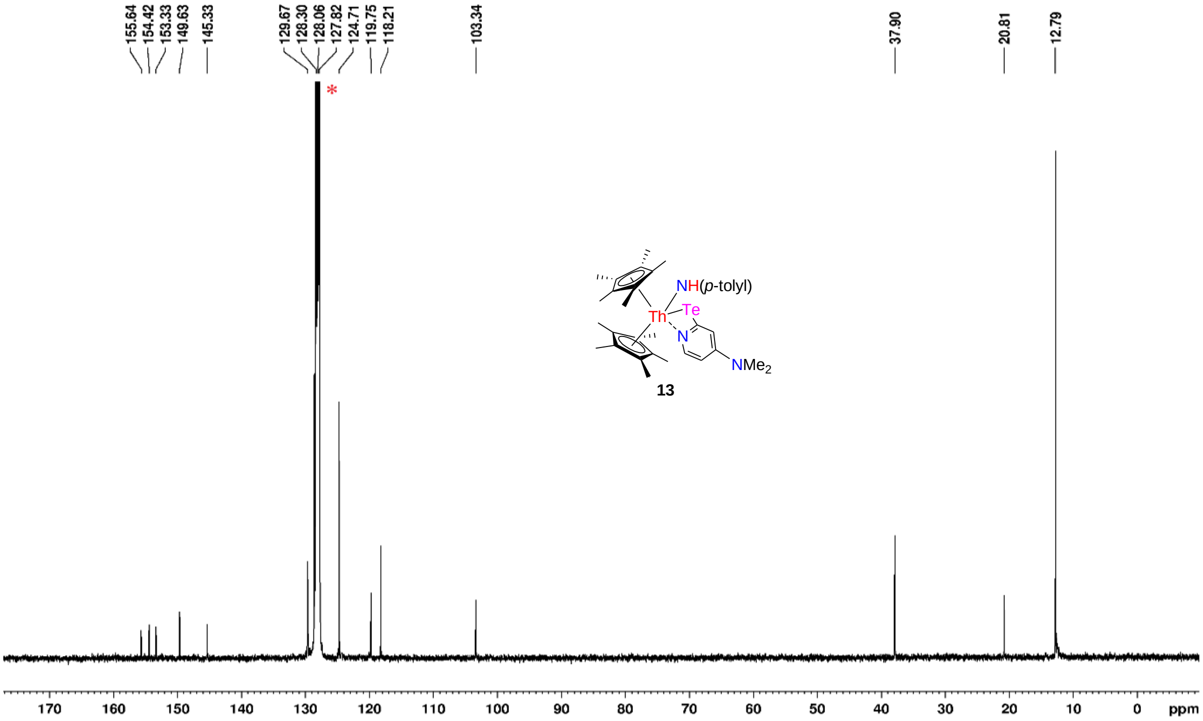

Figure S40. $^{13}C\{^{1}H\}$ NMR (C_6D_6; 20 ℃) spectrum for compound (η^5-$C_5Me_5)_2$Th[NH(*p*-tolyl)][κ^2-*N*,*Te*-2-Te-4-($Me_2N)C_5H_3N$] (**13**) (* solvent). $^{13}C\{^{1}H\}$ NMR (C_6D_6): δ 155.6 (py *C*), 154.4 (py *C*), 153.3 (py *C*), 149.6 (phenyl *C*), 145.3 (phenyl *C*), 129.7 (phenyl *C*), 124.7 (phenyl *C*), 119.8 (ring *C*), 118.2 (py *C*), 103.3 (py *C*), 37.9 (NCH_3), 20.8 (tolylCH_3), 12.8 (CpCH_3) ppm.

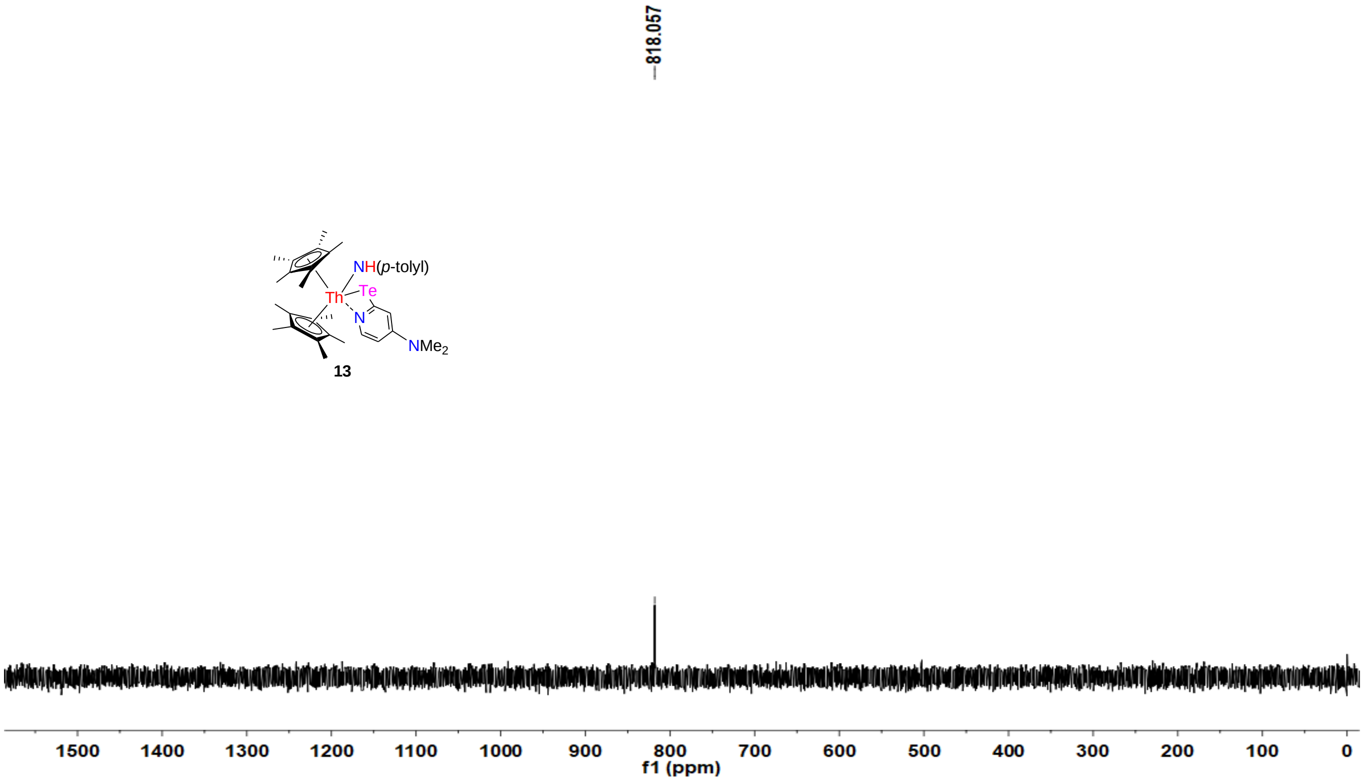

Figure S41. $^{125}Te\{^{1}H\}$ NMR (C_6D_6; 20 ℃) spectrum for compound (η^5-$C_5Me_5)_2$Th[NH(*p*-tolyl)][κ^2-*N*,*Te*-2-Te-4-($Me_2N)C_5H_3N$] (**13**). $^{125}Te\{^{1}H\}$ NMR (C_6D_6): δ 818.1 ppm.

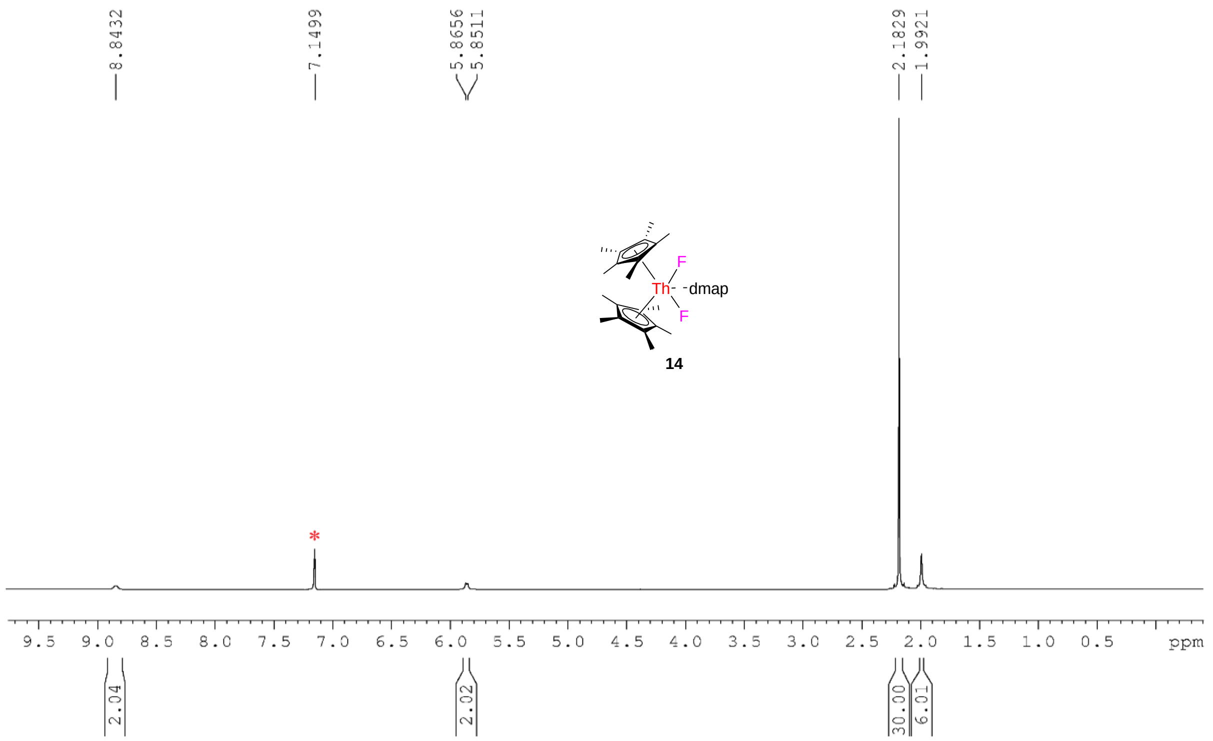

Figure S42. ^{1}H NMR (C_6D_6; 20 °C) spectrum for compound (η^5-C_5Me_5)$_2$ThF_2(dmap) (**14**) (* solvent). ^{1}H NMR (C_6D_6): δ 8.84 (s, 2H, py), 5.86 (d, J = 5.8 Hz, 2H, py), 2.18 (s, 30H, CpCH_3), 1.99 (s, 6H, NCH_3) ppm.

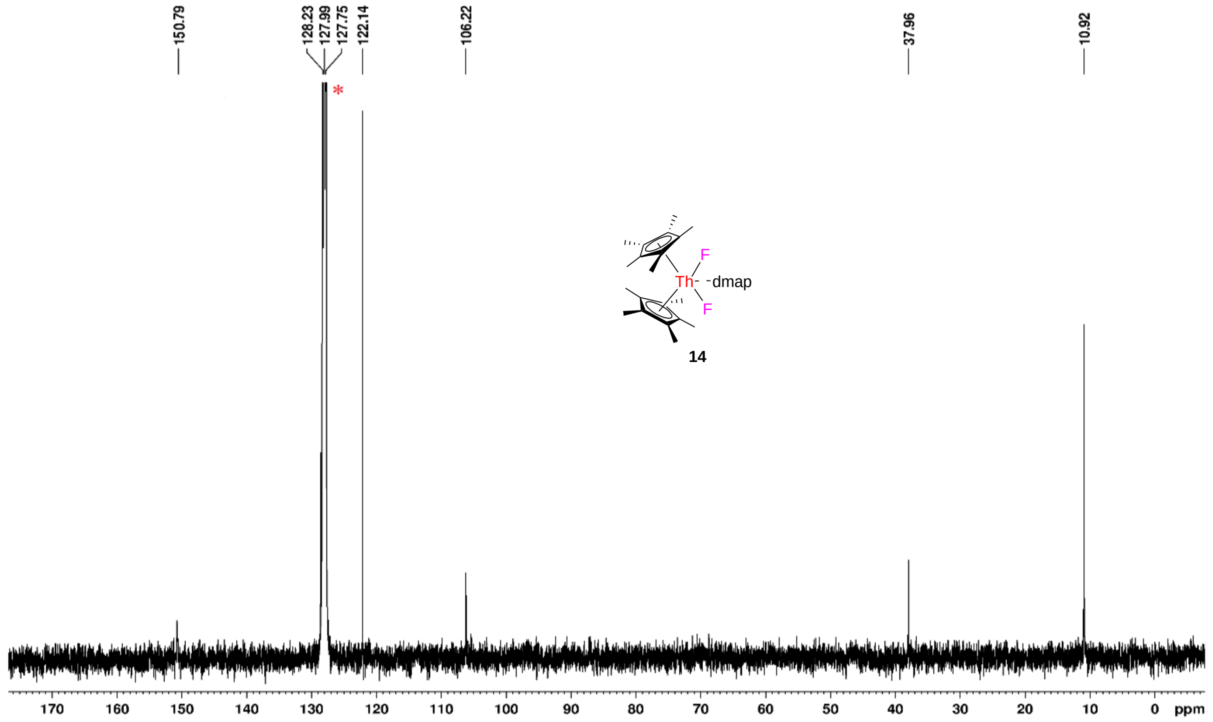

Figure S43. $^{13}C\{^{1}H\}$ NMR (C_6D_6; 20 °C) spectrum for compound (η^5-C_5Me_5)$_2$ThF_2(dmap) (**14**) (* solvent). $^{13}C\{^{1}H\}$ NMR (C_6D_6): δ 150.8 (py C), 122.1 (ring C), 106.2 (py C), 38.0 (NCH$_3$), 10.9 (CpCH$_3$) ppm; one carbon of dmap was not observed.

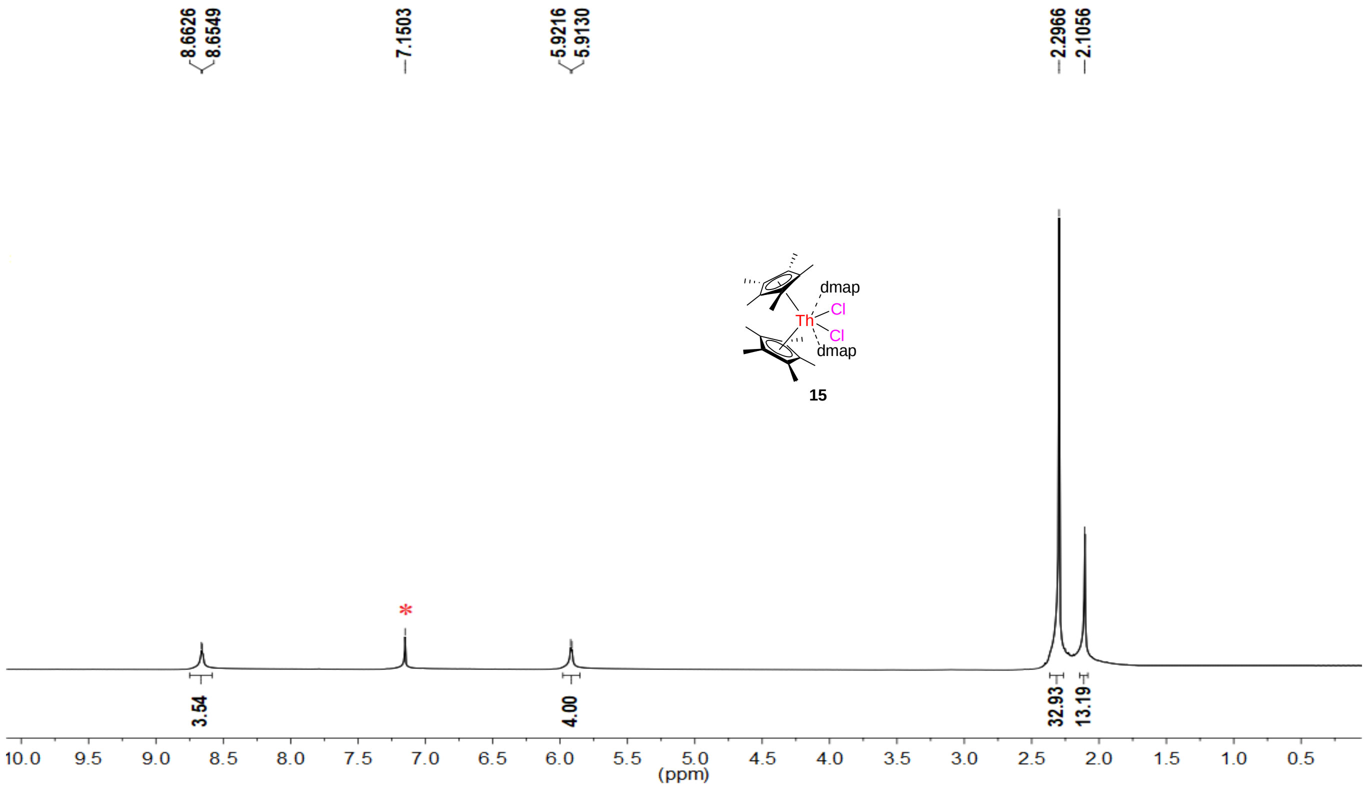

Figure S44. ^{1}H NMR (C_6D_6; 20 °C) spectrum for compound (η^5-C_5Me_5)$_2$ThCl_2(dmap)$_2$ (**15**) (* solvent). ^{1}H NMR (C_6D_6): δ 8.66 (d, J = 6.5 Hz, 4H, py), 5.92 (d, J = 6.2 Hz, 4H, py), 2.30 (s, 30H, CpCH_3), 2.11 (s, 12H, N(CH_3)$_2$) ppm.

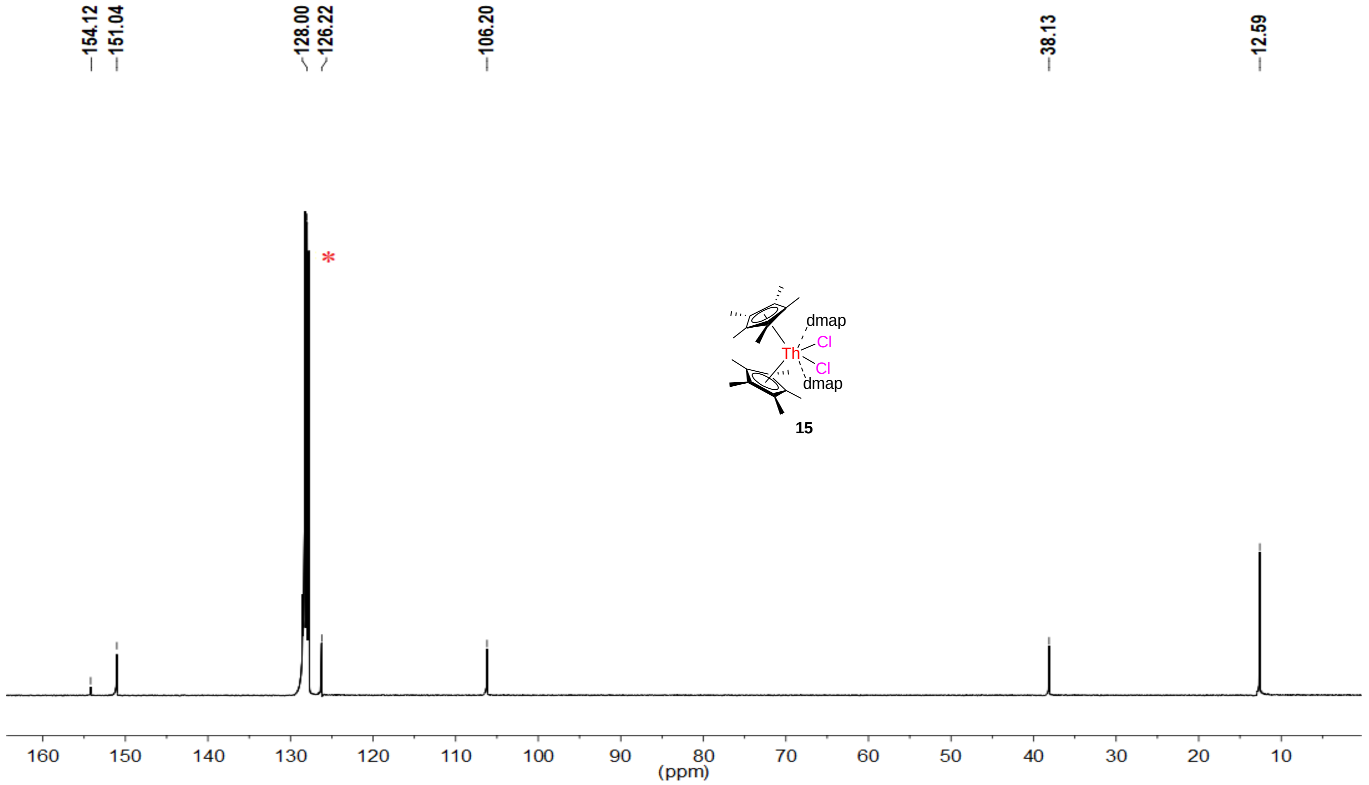

Figure S45. ^{13}C{^{1}H} NMR (C_6D_6; 20 °C) spectrum for compound (η^5-C_5Me_5)$_2$ThCl_2(dmap)$_2$ (**15**) (* solvent). ^{13}C{^{1}H} NMR (C_6D_6): δ 154.1 (py *C*), 151.0 (py *C*), 126.2 (ring *C*), 106.2 (py *C*), 38.1 (NCH_3), 12.6 (CpCH_3) ppm.

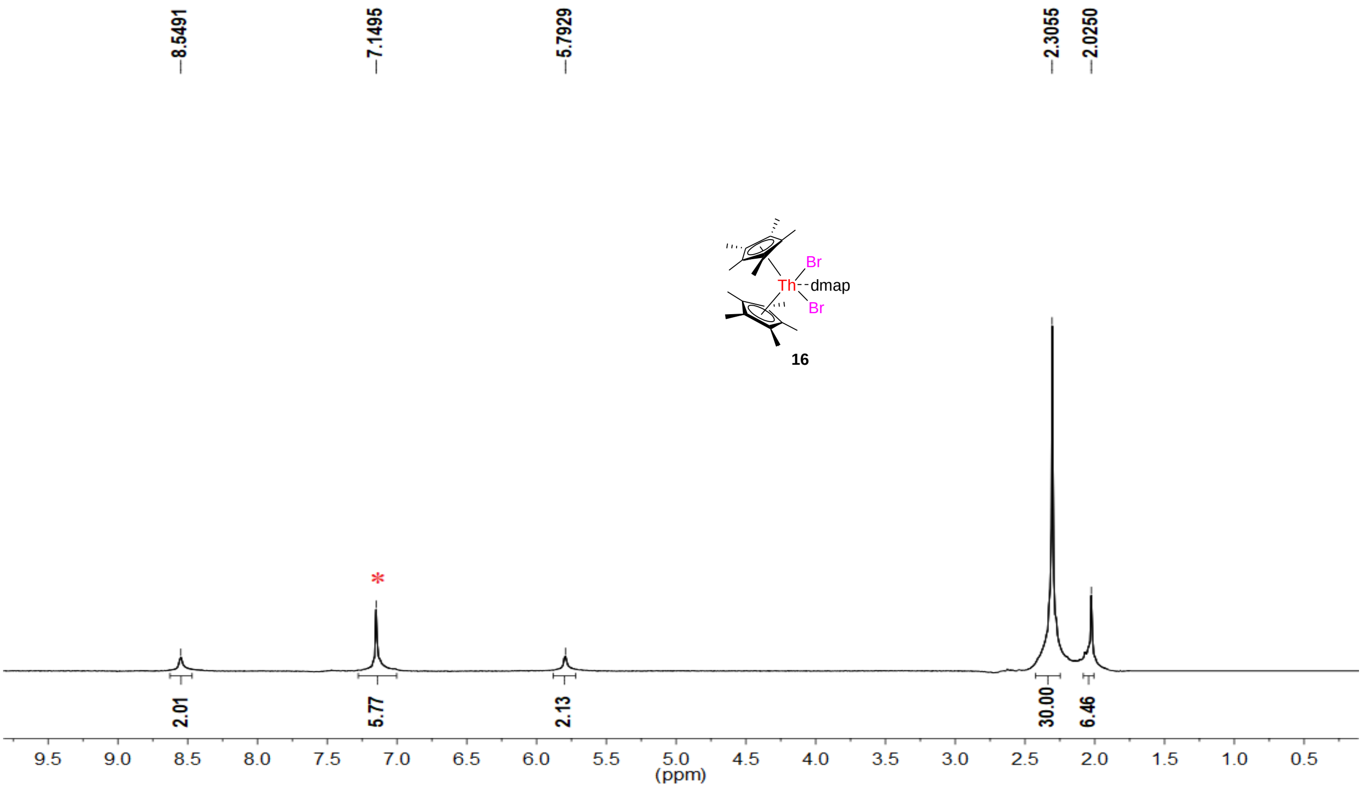

Figure S46. ^{1}H NMR (C_6D_6; 20 ℃) spectrum for compound (η^5-C_5Me_5)$_2$ThBr$_2$(dmap)·2C_6H_6 (**16**·2C_6H_6) (* solvent). ^{1}H NMR (C_6D_6): δ 8.55 (s, 2H, py), 7.15 (s, 12H, C_6*H_6*), 5.79 (s, 2H, py), 2.31 (s, 30H, CpC*H_3*), 2.03 (s, 6H, N(C*H_3*)$_2$) ppm.

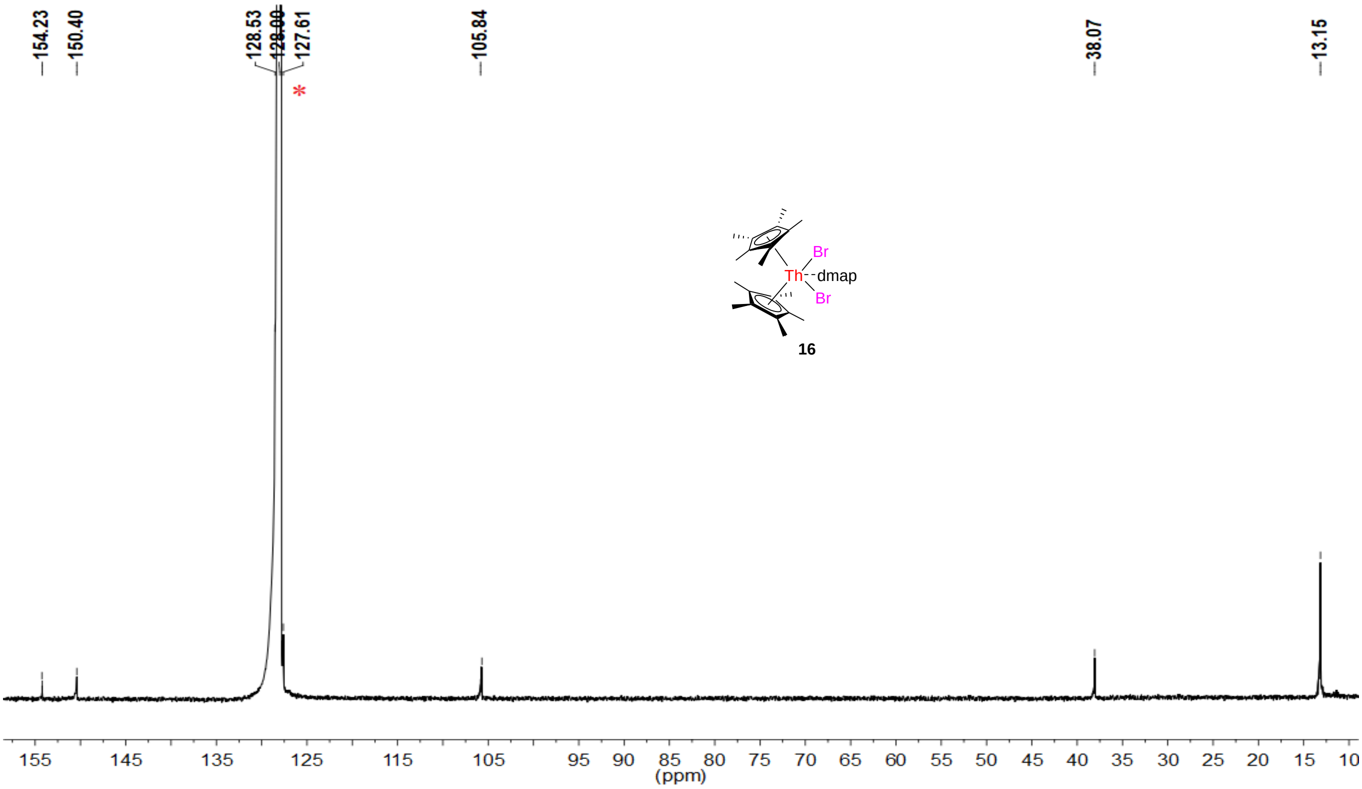

Figure S47. ^{13}C{^{1}H} NMR (C_6D_6; 20 ℃) spectrum for compound (η^5-C_5Me_5)$_2$ThBr$_2$(dmap)·2C_6H_6 (**16**·2C_6H_6) (* solvent). ^{13}C{^{1}H} NMR (C_6D_6): δ 154.2 (py *C*), 150.4 (py *C*), 128.5 (*C_6*H_6),127.6 (ring *C*), 105.8 (py *C*), 38.1 (N*C*H_3), 13.2 (Cp*C*H_3) ppm.

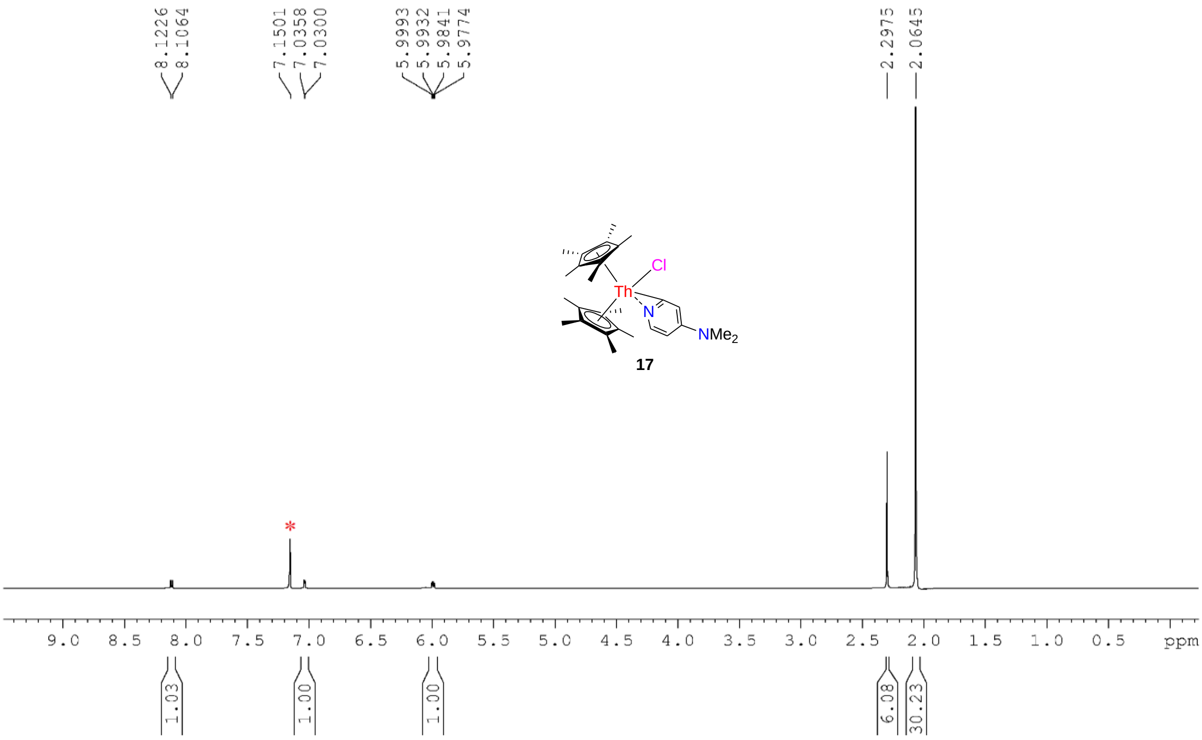

Figure S48. ^{1}H NMR (C_6D_6; 20 ℃) spectrum for compound (η^5-C_5Me_5)$_2$Th(Cl)[κ^2-*C*,*N*-4-(Me_2N)C_5H_3N] (**17**) (* solvent). ^{1}H NMR (C_6D_6): δ 8.11 (d, J = 6.5 Hz, 1H, py), 7.03 (d, J = 2.3 Hz, 1H, py), 5.99 (dd, J = 6.2 Hz and 2.6 Hz, 1H, py), 2.30 (s, 6H, N(CH_3)$_2$), 2.06 (s, 30H, CpCH_3) ppm.

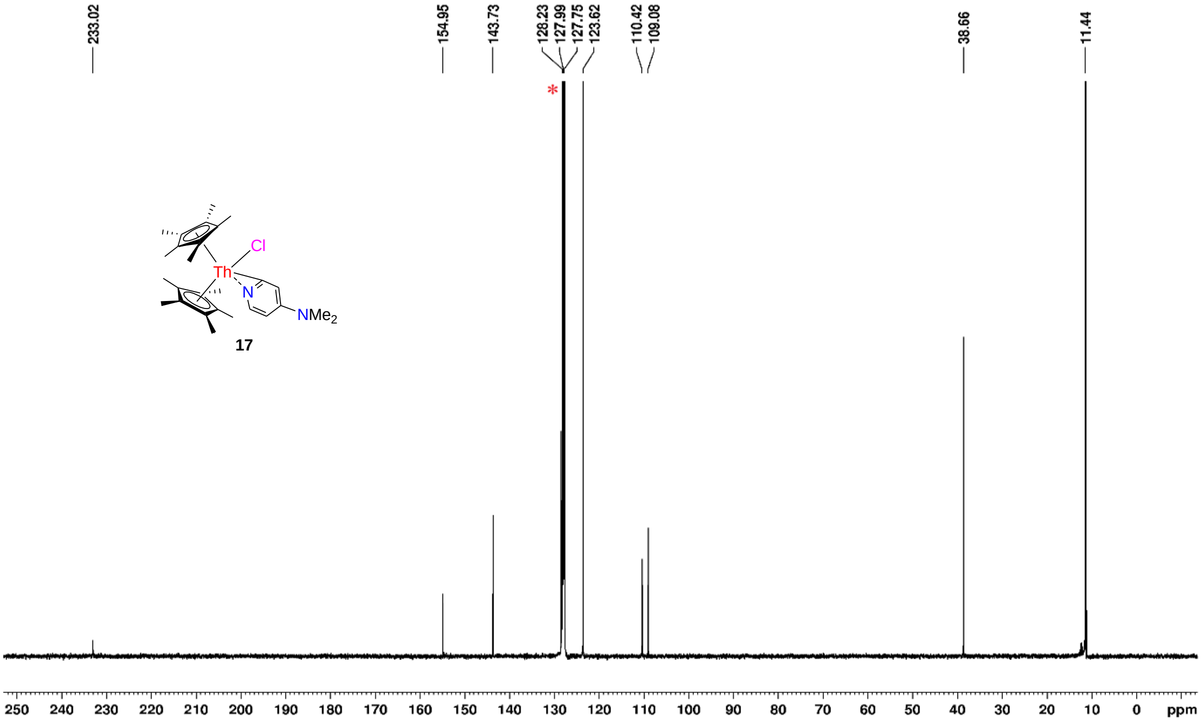

Figure S49. ^{13}C{^{1}H} NMR (C_6D_6; 20 ℃) spectrum for compound (η^5-C_5Me_5)$_2$Th(Cl)[κ^2-*C*,*N*-4-(Me_2N)C_5H_3N] (**17**) (* solvent). ^{13}C{^{1}H} NMR (C_6D_6): δ 233.0 (Th*C*), 154.9 (py *C*), 143.7 (py *C*), 123.6 (ring *C*), 110.4 (py *C*), 109.1 (py *C*), 38.7 (N*C*H_3), 11.4 (Cp*C*H_3) ppm.

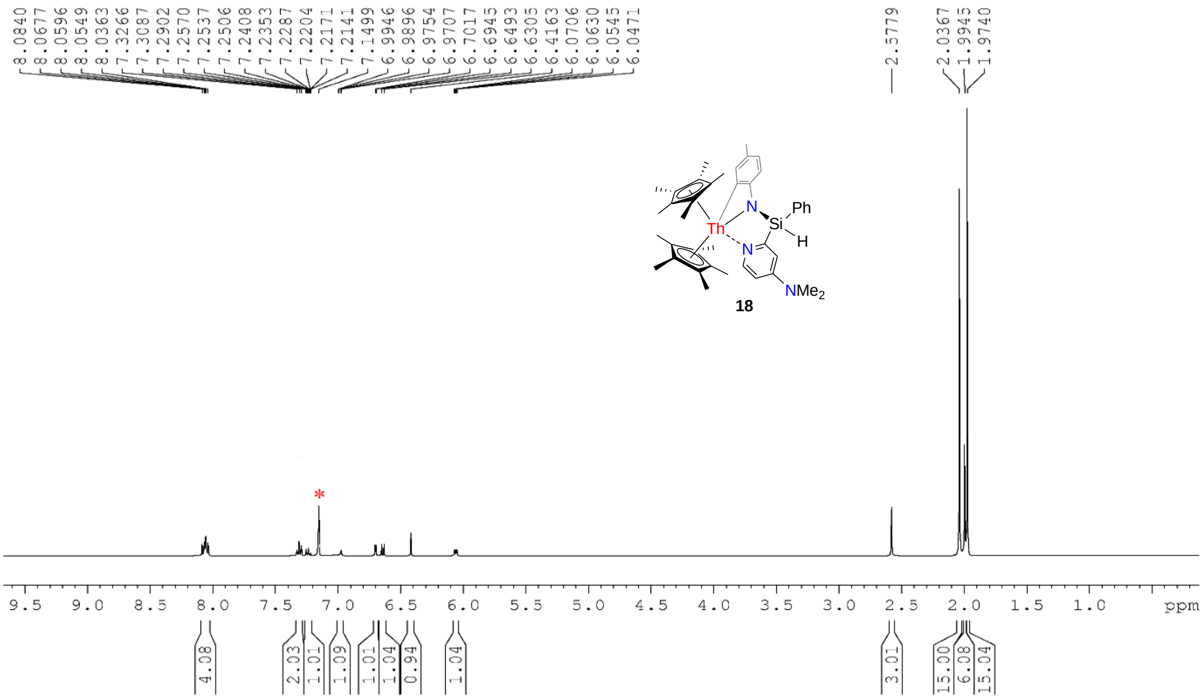

Figure S50. ^{1}H NMR (C_6D_6; 20 ℃) spectrum for compound (η^5-C_5Me_5)$_2$Th[κ^3-*C*,*N*,*N*-(4-$Me_2NC_5H_3N$)SiH(Ph)N(4-MeC_6H_3)] (**18**) (* solvent). ^{1}H NMR (C_6D_6): δ 8.06 (m, 4H, phenyl), 7.31 (t, *J* = 7.3 Hz, 2H, phenyl), 7.23 (m, 1H, py), 6.98 (m, 1H, phenyl), 6.70 (d, *J* = 2.9 Hz, 1H, py), 6.64 (d, *J* = 7.5 Hz, 1H, phenyl), 6.42 (s, 1H, Si*H*), 6.06 (dd, *J* = 6.4 Hz and 3.0 Hz, 1H, py), 2.58 (s, 3H, tolylC*H*$_3$), 2.04 (s, 15H, CpC*H*$_3$), 1.99 (s, 6H, N(C*H*$_3$)$_2$), 1.97 (s, 15H, CpC*H*$_3$) ppm.

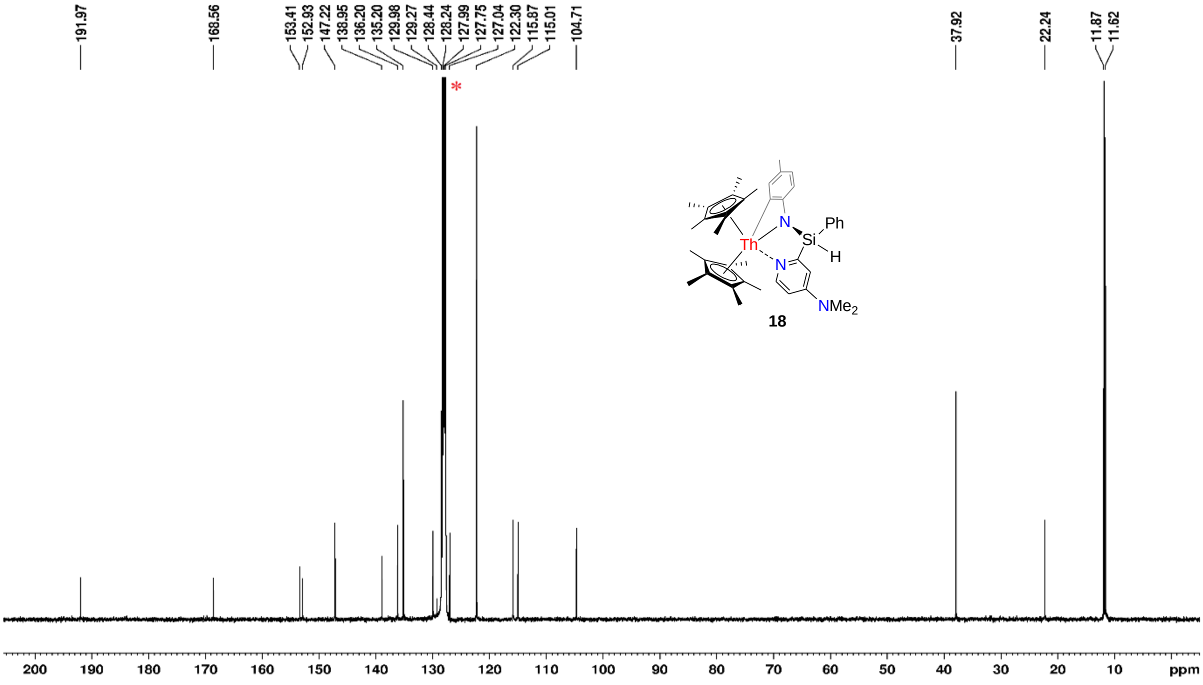

Figure S51. $^{13}C\{^{1}H\}$ NMR (C_6D_6; 20 ℃) spectrum for compound (η^5-C_5Me_5)$_2$Th[κ^3-*C*,*N*,*N*-(4-$Me_2NC_5H_3N$)SiH(Ph)N(4-MeC_6H_3)] (**18**) (* solvent). $^{13}C\{^{1}H\}$ NMR (C_6D_6): δ 192.0 (Th*C*), 168.6 (aryl *C*), 153.4 (aryl *C*), 152.9 (aryl *C*), 147.2 (aryl *C*), 139.0 (aryl *C*), 136.2 (aryl *C*), 135.2 (aryl *C*), 130.0 (aryl *C*), 129.3 (aryl *C*), 128.4 (aryl *C*), 127.0 (aryl *C*), 122.3 (ring *C*), 115.9 (aryl *C*), 115.0 (aryl *C*), 104.7 (aryl *C*), 37.9 (N*C*H$_3$), 22.2 (tolyl*C*H$_3$), 11.9 (Cp*C*H$_3$), 11.6 (Cp*C*H$_3$) ppm.

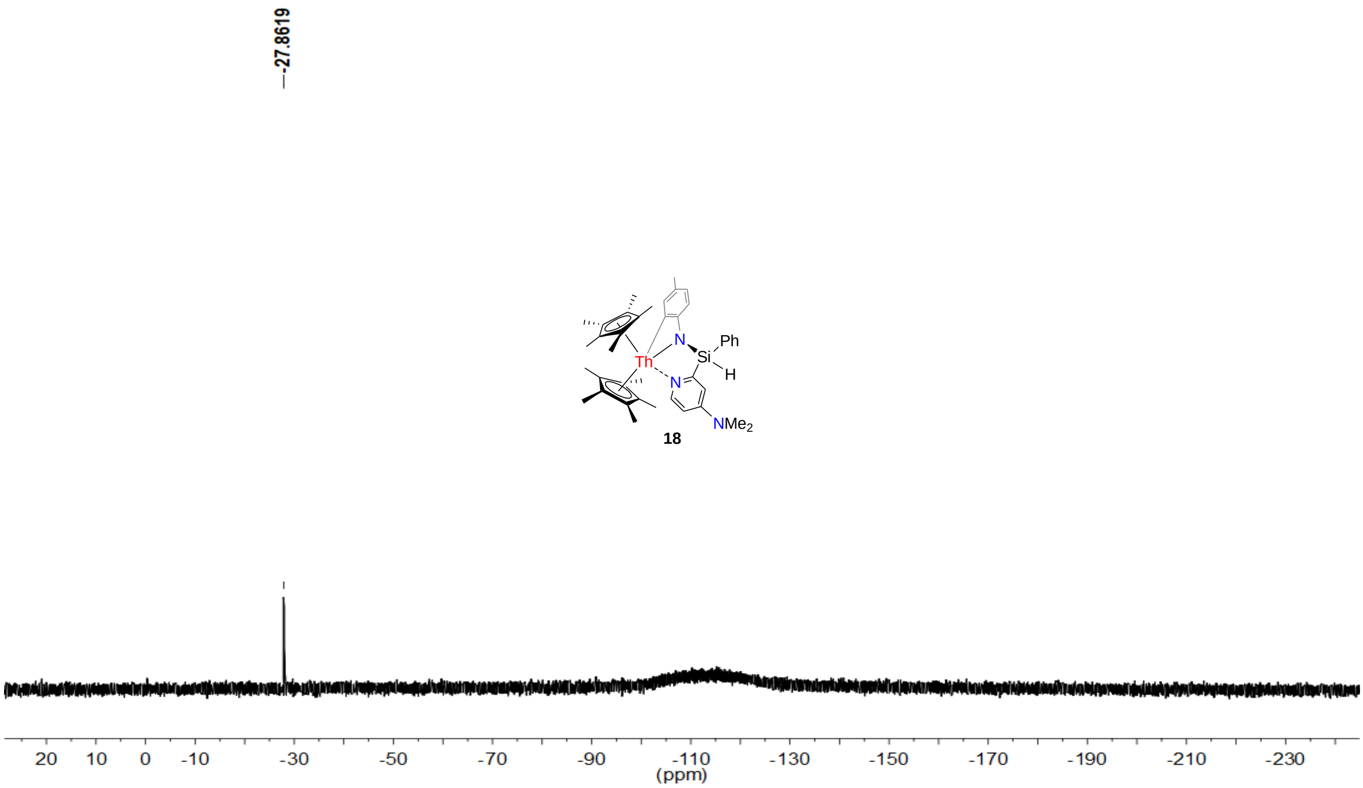

Figure S52. ^{29}Si{^{1}H} NMR (C_6D_6; 20 ℃) spectrum for compound (η^5-C_5Me_5)$_2$Th[κ^3-*C*,*N*,*N*-(4-$Me_2NC_5H_3N$)SiH(Ph)N(4-MeC_6H_3)] (**18**). ^{29}Si{^{1}H} NMR (C_6D_6): δ -27.9 ppm.

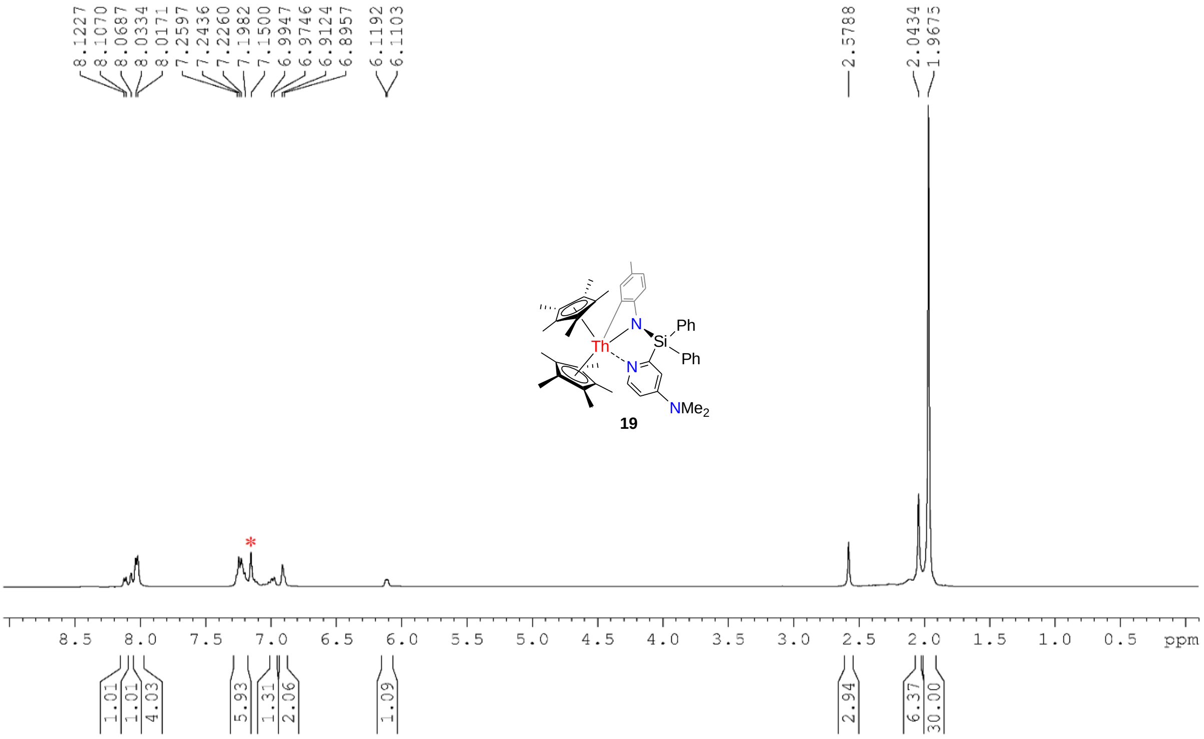

Figure S53. ^{1}H NMR (C_6D_6; 20 ℃) spectrum for compound (η^5-C_5Me_5)$_2$Th[κ^3-*C*,*N*,*N*-(4-$Me_2NC_5H_3N$)$SiPh_2N$(4-MeC_6H_3)] C_6H_6 (**19** C_6H_6) (* solvent). ^{1}H NMR (C_6D_6): δ 8.11 (d, *J* = 6.3 Hz, 1H, py), 8.07 (s, 1H, phenyl), 8.03 (d, *J* = 6.5 Hz, 4H, phenyl), 7.23 (m, 6H, phenyl), 7.15 (s, 6H, C_6H_6), 6.99 (d, *J* = 8.0 Hz, 1H, phenyl), 6.90 (m, 2H, phenyl and py), 6.11 (d, 1H, *J* = 3.6 Hz, py), 2.58 (s, 3H, tolylCH_3), 2.04 (s, 6H, $N(CH_3)_2$), 1.97 (s, 30H, CpCH_3) ppm.

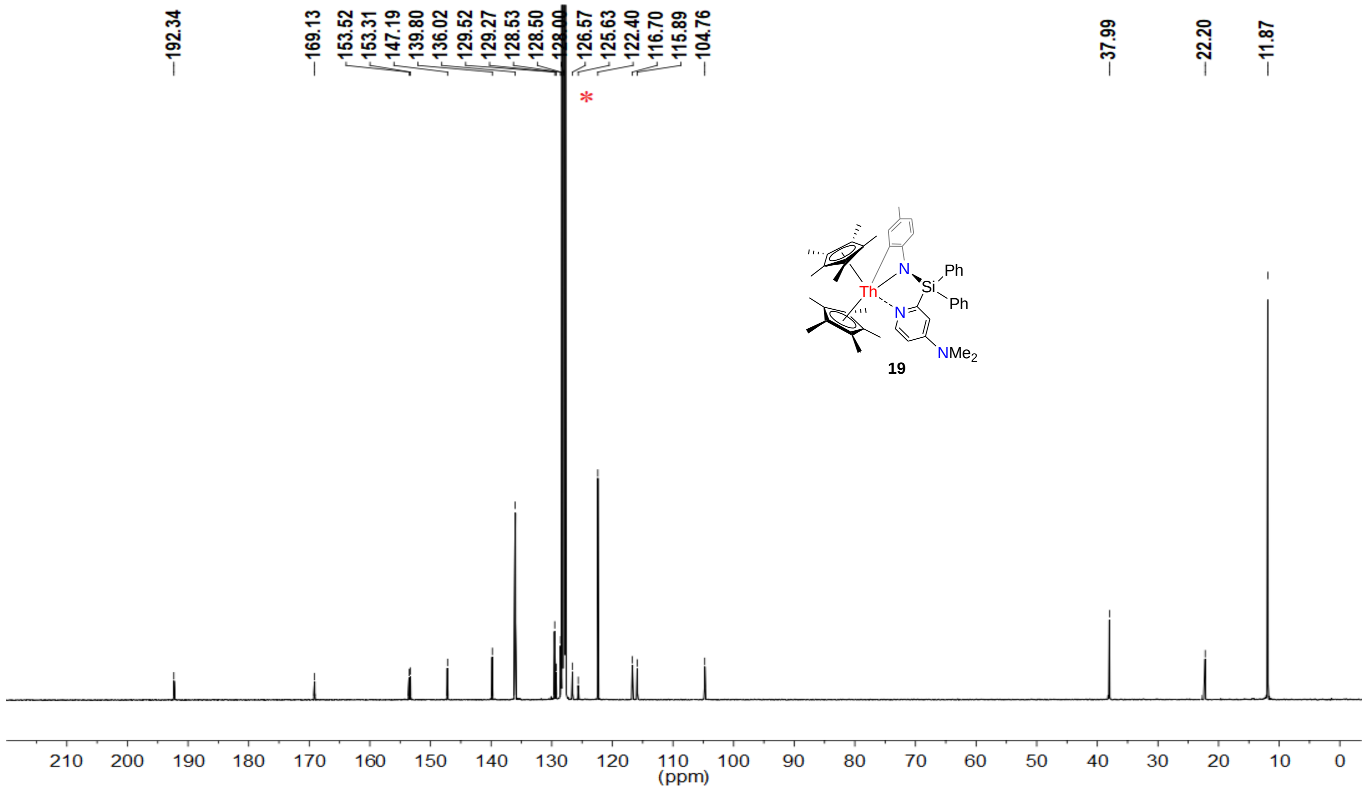

Figure S54. $^{13}C\{^{1}H\}$ NMR (C_6D_6; 20 ℃) spectrum for compound (η^5-C_5Me_5)$_2$Th[κ^3-*C*,*N*,*N*-(4-$Me_2NC_5H_3N$)$SiPh_2N$(4-MeC_6H_3)]·C_6H_6 (**19**·C_6H_6) (* solvent). $^{13}C\{^{1}H\}$ NMR (C_6D_6): δ 192.3 (Th*C*), 169.1 (aryl *C*), 153.5 (aryl *C*), 153.3 (aryl *C*), 147.2 (aryl *C*), 139.8 (aryl *C*), 136.0 (aryl *C*), 129.5 (aryl *C*), 129.3 (aryl *C*), 128.53 (aryl *C*), 128.50 (C_6H_6), 126.6 (aryl *C*), 125.6 (aryl *C*), 122.4 (ring *C*), 116.7 (aryl *C*), 115.9 (aryl *C*), 104.8 (aryl *C*), 38.0 (NCH_3), 22.2 (tolylCH_3), 11.9 (CpCH_3) ppm.

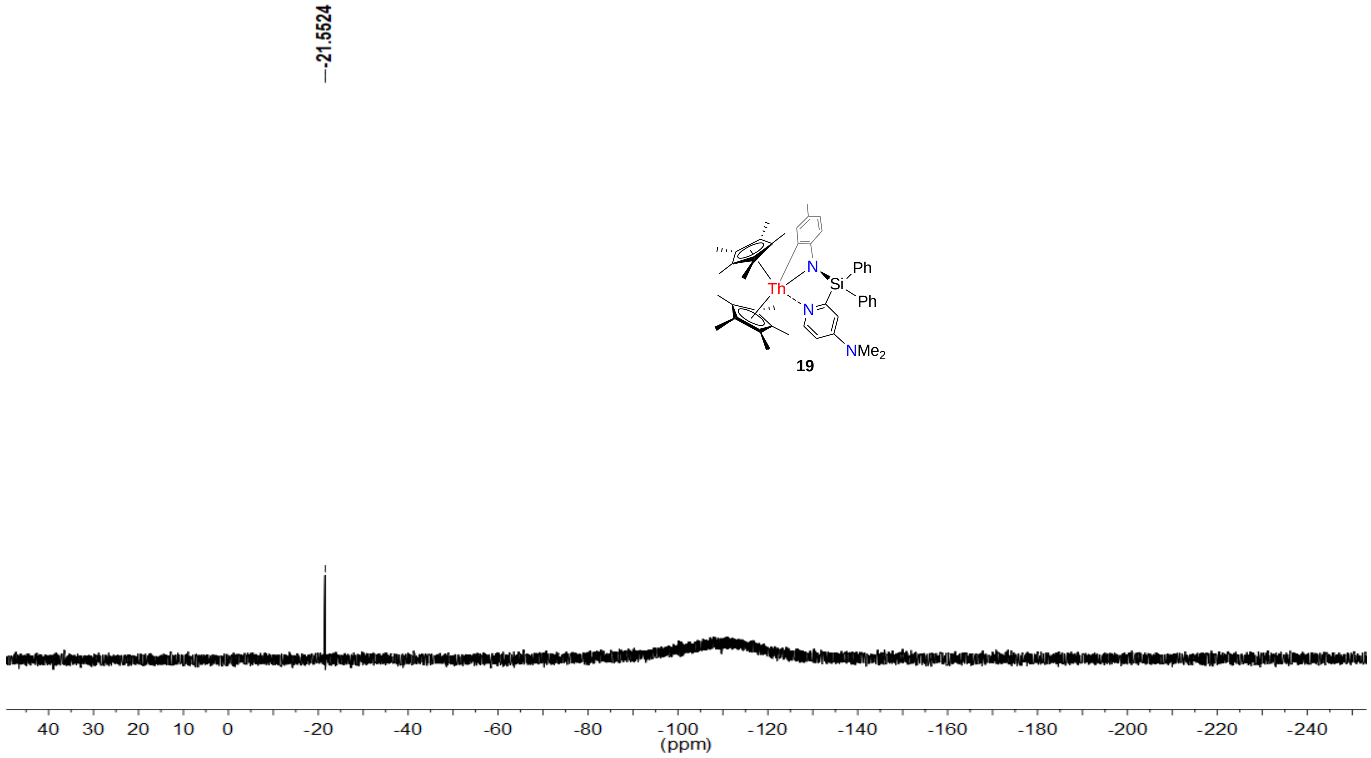

Figure S55. $^{29}Si\{^{1}H\}$ NMR (C_6D_6; 20 ℃) spectrum for compound (η^5-C_5Me_5)$_2$Th[κ^3-*C*,*N*,*N*-(4-$Me_2NC_5H_3N$)$SiPh_2N$(4-MeC_6H_3)]·C_6H_6 (**19**·C_6H_6). $^{29}Si\{^{1}H\}$ NMR (C_6D_6): δ -21.6 ppm.

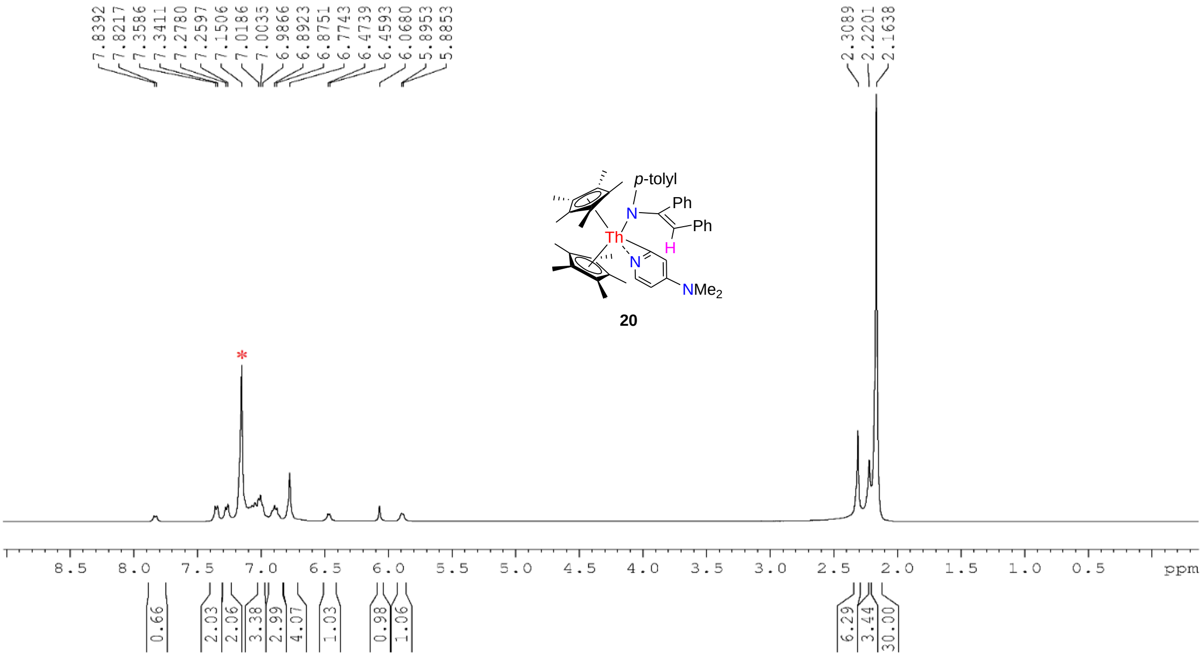

Figure S56. ^{1}H NMR (C_6D_6; 20 ℃) spectrum for compound (η^5-C_5Me_5)$_2$Th[N(*p*-tolyl)C(Ph)=CHPh][κ^2-*C*,*N*-4-(Me_2N)C_5H_3N] (**20**) (* solvent). ^{1}H NMR (C_6D_6): δ 7.83 (d, *J* = 7.0 Hz, 1H, py), 7.35 (d, *J* = 7.0 Hz, 2H, phenyl), 7.27 (d, *J* = 7.3 Hz, 2H, phenyl), 7.00 (m, 3H, phenyl), 6.88 (m, 3H, phenyl), 6.77 (s, 4H, phenyl), 6.46 (d, *J* = 5.9 Hz, 1H, py), 6.06 (s, 1H, C*H*=C), 5.89 (d, *J* = 4.0 Hz, 1H, py), 2.31 (s, 6H, N(CH_3)$_2$), 2.22 (s, 3H, tolylCH_3), 2.16 (s, 30H, CpCH_3) ppm.

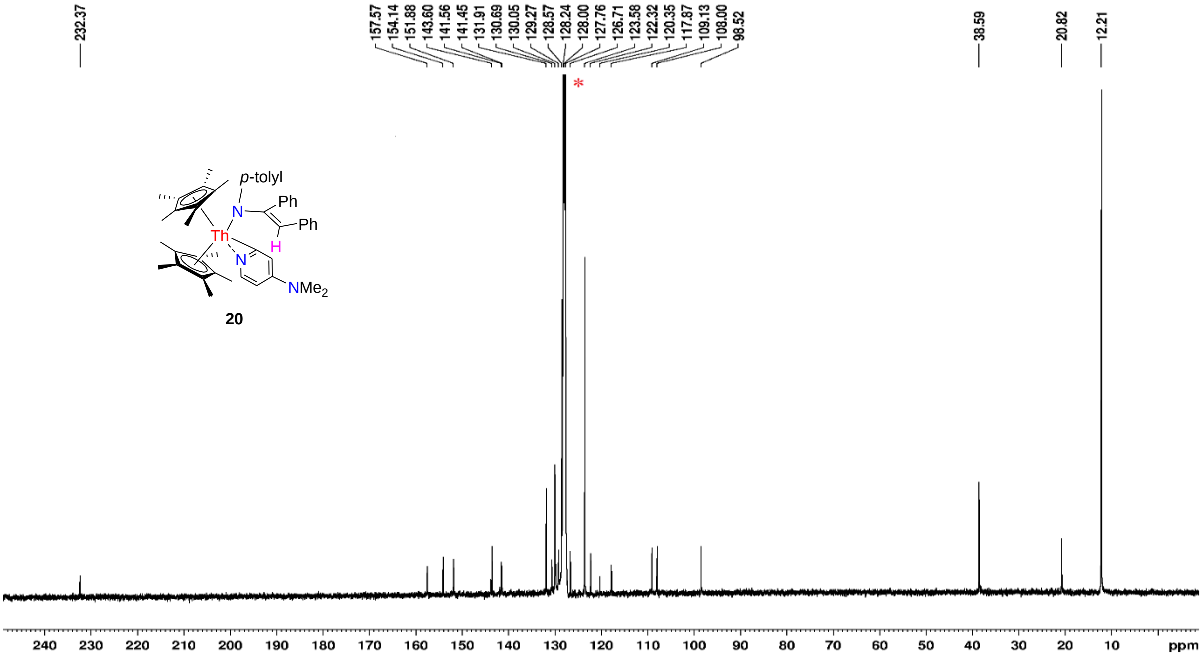

Figure S57. $^{13}C\{^{1}H\}$ NMR (C_6D_6; 20 ℃) spectrum for compound (η^5-C_5Me_5)$_2$Th[N(*p*-tolyl)C(Ph)=CHPh][κ^2-*C*,*N*-4-(Me_2N)C_5H_3N] (**20**) (* solvent). $^{13}C\{^{1}H\}$ NMR (C_6D_6): δ 232.4 (Th*C*), 157.6 (aryl *C*), 154.1 (aryl *C*), 151.9 (aryl *C*), 143.6 (aryl *C*), 141.6 (aryl *C*), 141.5 (aryl *C*), 131.9 (aryl *C*), 130.7 (aryl *C*), 130.1 (aryl *C*), 129.3 (aryl *C*), 128.6 (aryl *C*), 126.7 (aryl *C*), 123.6 (ring *C*), 122.3 (aryl *C*), 120.4 (aryl *C*), 117.9 (aryl *C*), 109.1 (aryl *C*), 108.0 (*C*=CH), 98.5 (C=*C*H), 38.6 (N*C*H_3), 20.8 (tolyl*C*H_3), 12.2 (Cp*C*H_3) ppm.

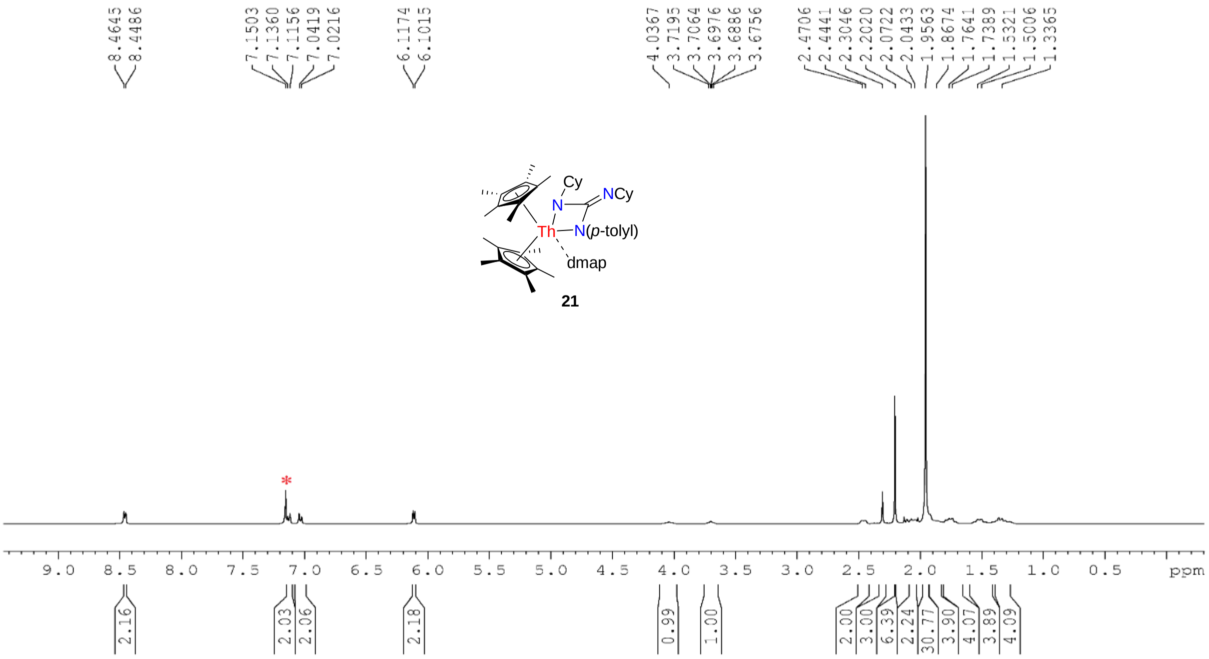

Figure S58. ^{1}H NMR (C_6D_6; 20 °C) spectrum for compound (η^5-C_5Me_5)$_2$Th[N(*p*-tolyl)C(=NC_6H_{11})N(C_6H_{11})](dmap) (**21**) (* solvent). ^{1}H NMR (C_6D_6): δ 8.46 (d, J = 6.4 Hz, 2H, py), 7.13 (d, J = 8.2 Hz, 2H, phenyl), 7.03 (d, J = 8.2 Hz, 2H, phenyl), 6.11 (d, J = 6.4 Hz, 2H, py), 4.04 (m, 1H, NC*H*), 3.70 (m, 1H, NC*H*), 2.46 (m, 2H, Cy), 2.30 (s, 3H, tolylCH_3), 2.20 (s, 6H, N(CH_3)$_2$), 2.06 (m, 2H, CH_2), 1.96 (s, 30H, CpCH_3), 1.87 (m, 4H, CH_2), 1.75 (m, 4H, CH_2), 1.52 (m, 4H, CH_2), 1.34 (m, 4H, CH_2) ppm.

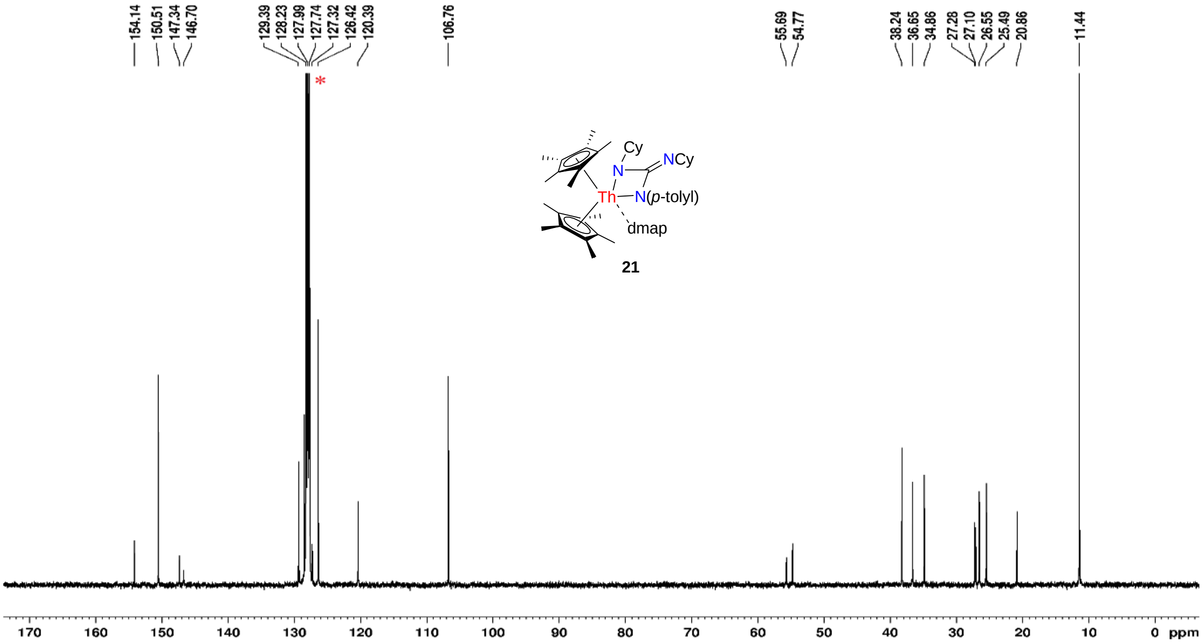

Figure S59. ^{13}C{^{1}H} NMR (C_6D_6; 20 °C) spectrum for compound (η^5-C_5Me_5)$_2$Th[N(*p*-tolyl)C(=NC_6H_{11})N(C_6H_{11})](dmap) (**21**) (* solvent). ^{13}C{^{1}H} NMR (C_6D_6): δ 154.1 (py *C*), 150.5 (py *C*), 147.3 (phenyl *C*), 146.7 (phenyl *C*), 129.4 (phenyl *C*), 127.3 (phenyl *C*), 126.4 (ring *C*), 120.4 (*C*=N), 106.8 (py *C*), 55.7 (N*C*H), 54.8 (N*C*H), 38.2 (N*C*H_3), 36.7 (Cy *C*), 34.9 (Cy *C*), 27.3 (Cy *C*), 27.1 (Cy *C*), 26.6 (Cy *C*), 25.5 (Cy *C*), 20.9 (tolyl*C*H_3), 11.4 (Cp*C*H_3) ppm.

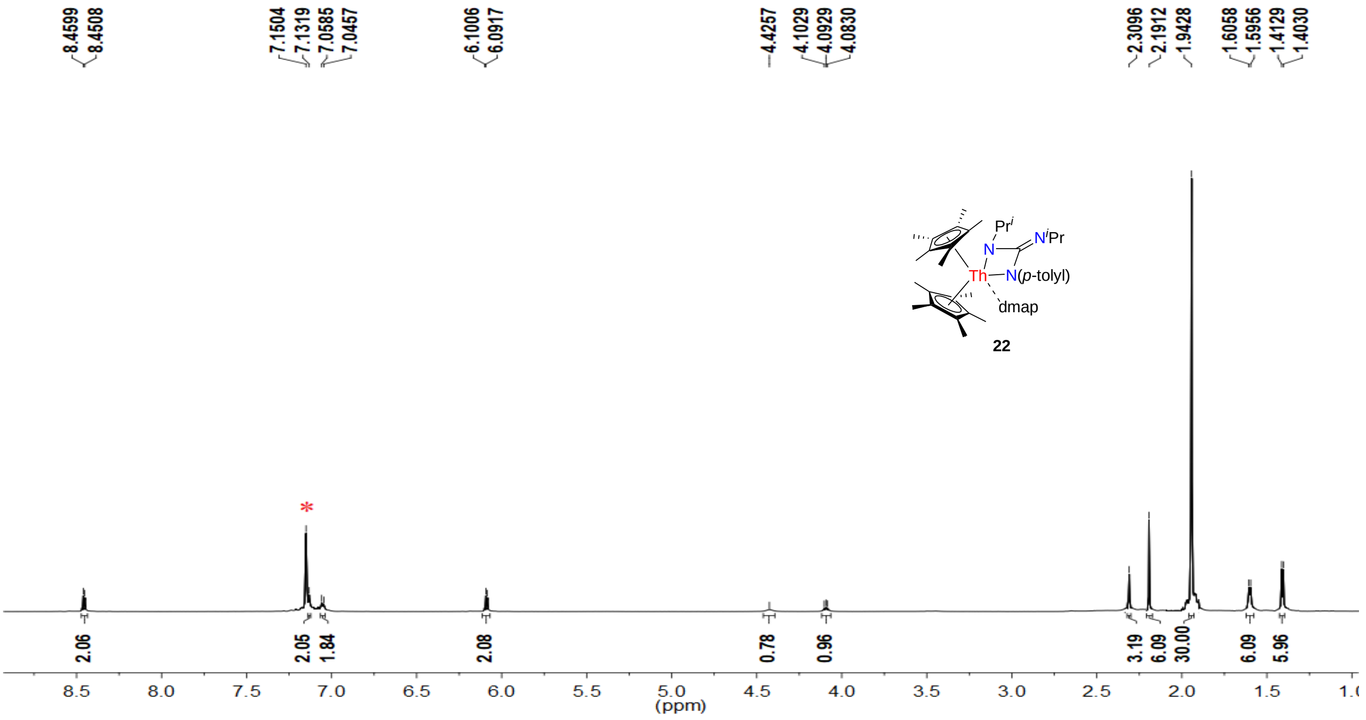

Figure S60. ^{1}H NMR (C_6D_6; 20 ℃) spectrum for compound (η^5-C_5Me_5)$_2$Th[N(*p*-tolyl)C(=N^{i}Pr)N(^{i}Pr)](dmap) (**22**) (* solvent). ^{1}H NMR (C_6D_6): δ 8.46 (d, J = 5.5 Hz, 2H, py), 7.13 (d, J = 7.7 Hz, 2H, phenyl), 7.05 (d, J = 7.7 Hz, 2H, phenyl), 6.10 (d, J = 5.3 Hz, 2H, py), 4.43 (s, 1H, NC*H*), 4.09 (m, 1H, NC*H*), 2.31 (s, 3H, tolylCH_3), 2.19 (s, 6H, N(CH_3)$_2$), 1.94 (s, 30H, CpCH_3), 1.60 (d, J = 6.1 Hz, 6H, CH(CH_3)$_2$), 1.41 (d, J = 5.9 Hz, 6H, CH(CH_3)$_2$) ppm.

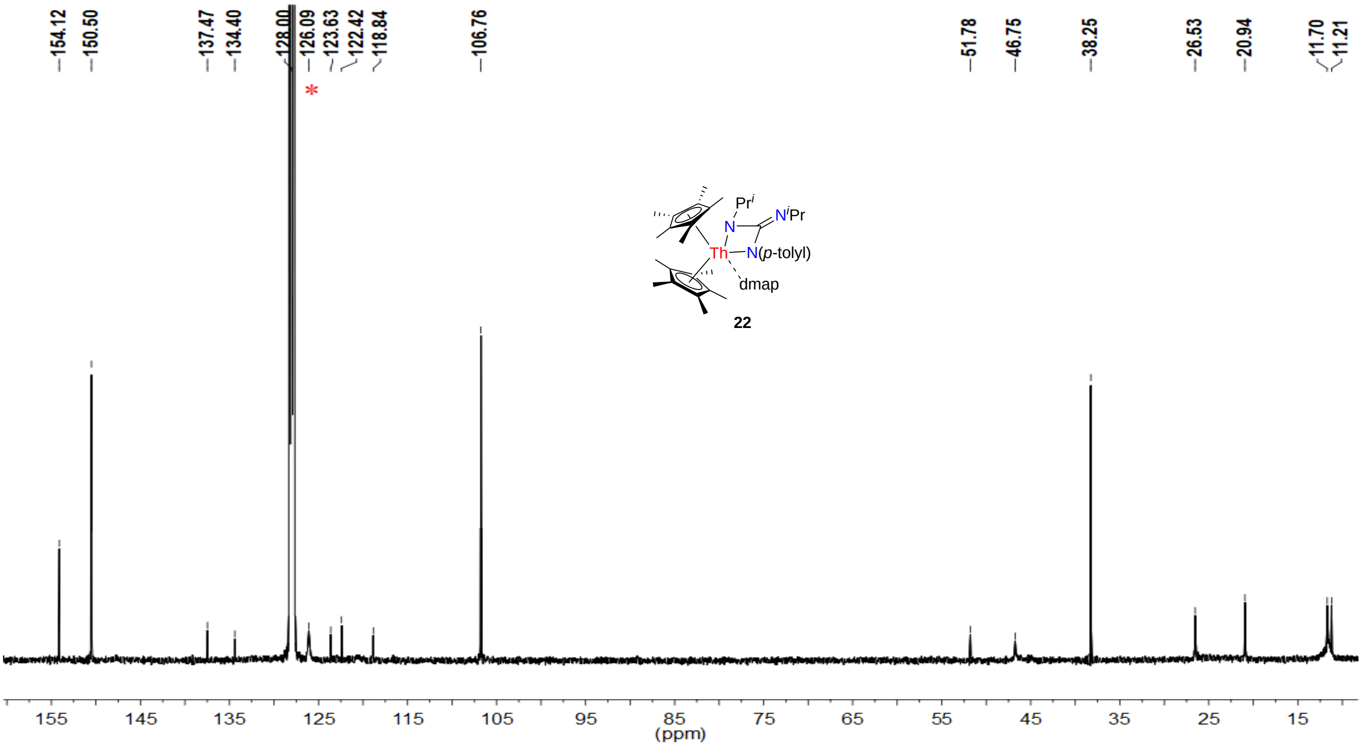

Figure S61. ^{13}C{^{1}H} NMR (C_6D_6; 20 ℃) spectrum for compound (η^5-C_5Me_5)$_2$Th[N(*p*-tolyl)C(=N^{i}Pr)N(^{i}Pr)](dmap) (**22**) (* solvent). ^{13}C{^{1}H} NMR (C_6D_6): δ 154.1 (py *C*), 150.5 (py *C*), 137.5 (phenyl *C*), 134.4 (phenyl *C*), 126.1 (ring *C*), 123.6 (phenyl *C*), 122.4 (phenyl *C*), 118.8 (*C*=N), 106.8 (py *C*), 51.8 (N*C*H), 46.7 (N*C*H), 38.3 (N*C*H$_3$), 26.5 (CH(*C*H$_3$)$_2$), 20.9 (tolyl*C*H$_3$), 11.7 (CH(*C*H$_3$)$_2$), 11.2 (Cp*C*H$_3$) ppm.

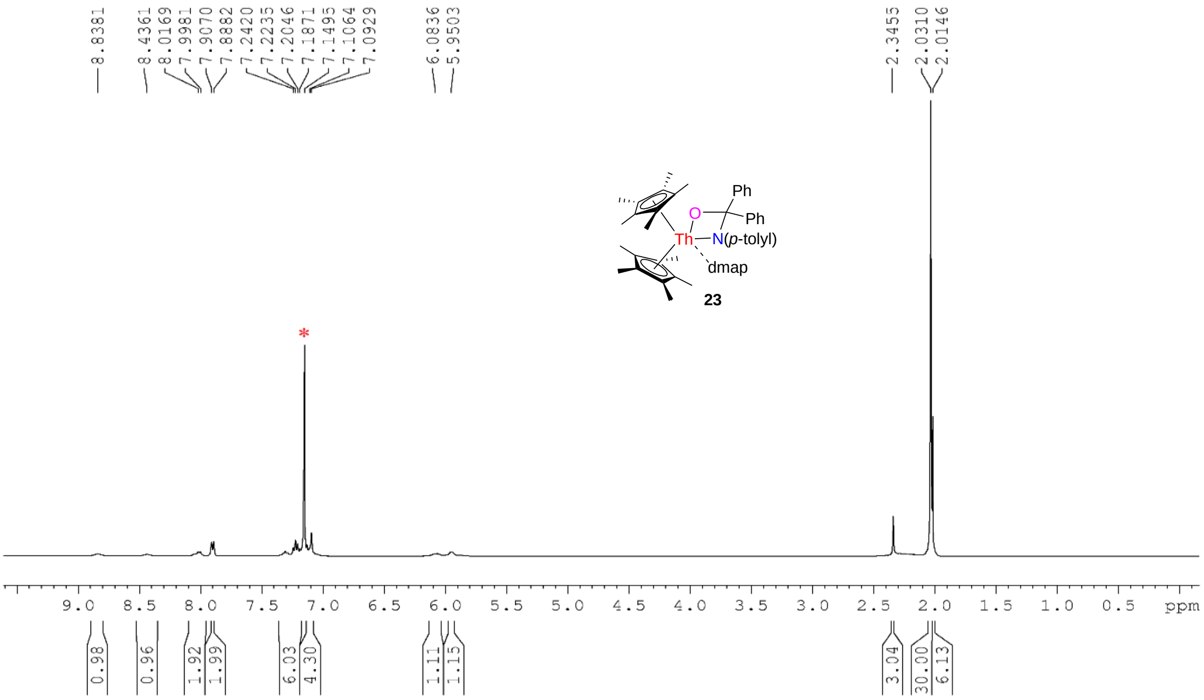

Figure S62. ^{1}H NMR (C_6D_6; 20 ℃) spectrum for compound (η^5-C_5Me_5)$_2$Th[N(*p*-tolyl)CPh_2O](dmap) (**23**) (* solvent). ^{1}H NMR (C_6D_6): δ 8.84 (s, 1H, py), 8.44 (s, 1H, py), 8.01 (d, J = 7.5 Hz, 2H, phenyl), 7.90 (d, J = 7.5 Hz, 2H, phenyl), 7.21 (m, 6H, phenyl), 7.10 (m, 4H, phenyl), 6.08 (s, 1H, py), 5.95 (s, 1H, py), 2.35 (s, 3H, tolylCH_3), 2.03 (s, 30H, CpCH_3), 2.01 (s, 6H, N(CH_3)$_2$) ppm.

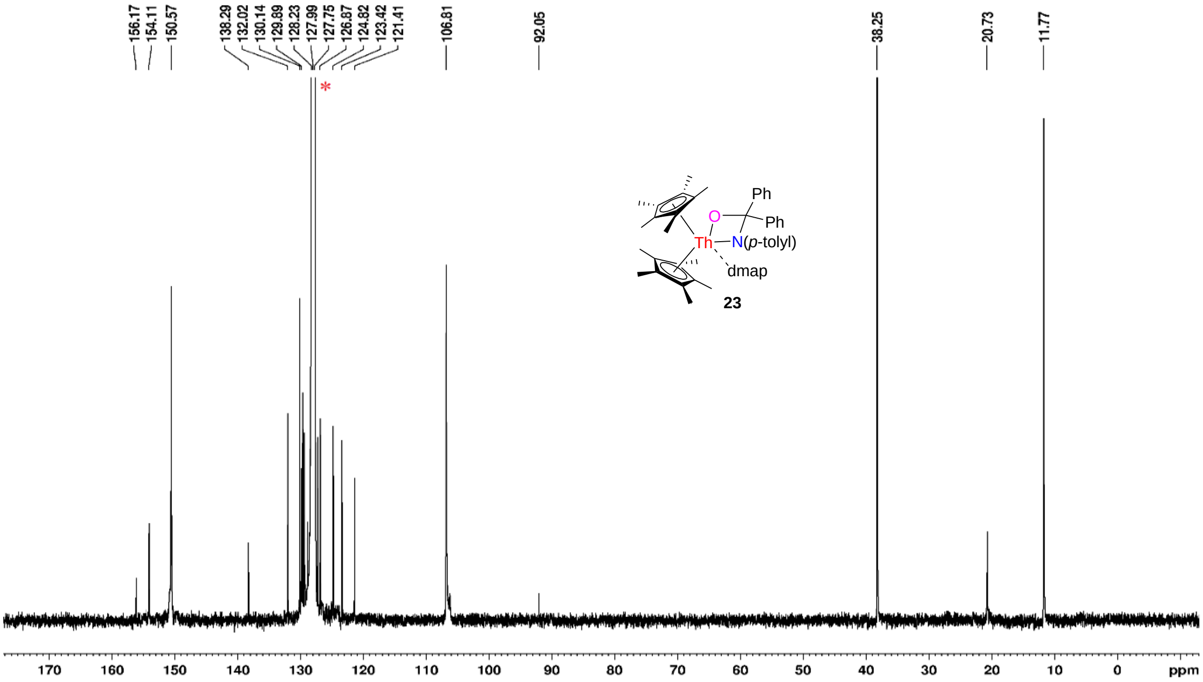

Figure S63. $^{13}C\{^{1}H\}$ NMR (C_6D_6; 20 ℃) spectrum for compound (η^5-C_5Me_5)$_2$Th[N(*p*-tolyl)CPh_2O](dmap) (**23**) (* solvent). $^{13}C\{^{1}H\}$ NMR (C_6D_6): δ 156.2 (phenyl *C*), 154.1 (py *C*), 150.6 (py *C*), 138.3 (phenyl *C*), 132.0 (phenyl *C*), 130.1 (phenyl *C*), 129.9 (phenyl *C*), 126.9 (phenyl *C*), 124.8 (phenyl *C*), 123.4 (ring *C*), 121.4 (phenyl *C*), 106.8 (py *C*), 92.1 (*C*O), 38.3 (N*C*H_3), 20.7 (tolyl*C*H_3), 11.8 (Cp*C*H_3) ppm.

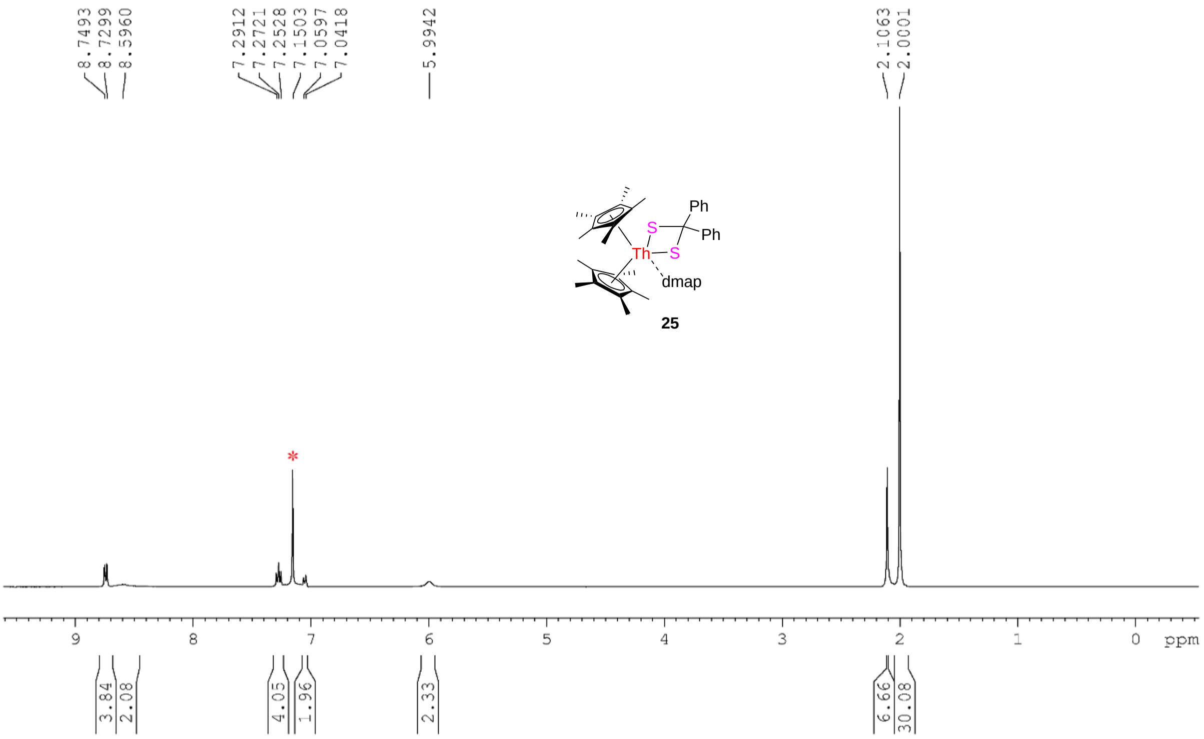

Figure S64. ^{1}H NMR (C_6D_6; 20 ℃) spectrum for compound (η^5-C_5Me_5)$_2$Th[SCPh$_2$S](dmap) (**25**) (* solvent). ^{1}H NMR (C_6D_6): δ 8.74 (d, J = 7.8 Hz, 4H, phenyl), 8.60 (br s, 2H, py), 7.27 (t, J = 7.6 Hz, 4H, phenyl), 7.05 (m, 2H, phenyl), 5.99 (br s, 2H, py), 2.11 (s, 6H, N(CH_3)$_2$), 2.00 (s, 30H, CpCH_3) ppm.

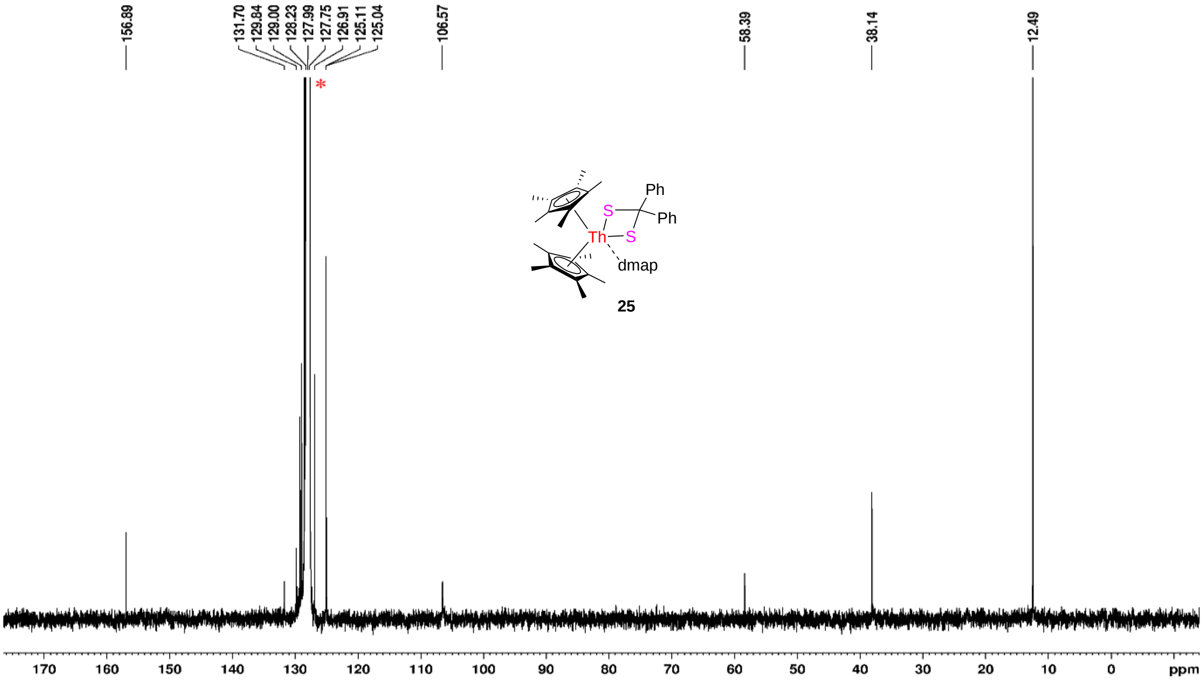

Figure S65. ^{13}C{^{1}H} NMR (C_6D_6; 20 ℃) spectrum for compound (η^5-C_5Me_5)$_2$Th[SCPh$_2$S](dmap) (**25**) (* solvent). ^{13}C{^{1}H} NMR (C_6D_6): δ 156.8 (py *C*), 131.7 (py *C*), 129.8 (phenyl *C*), 129.0 (phenyl *C*), 126.9 (phenyl *C*), 125.1 (phenyl *C*), 125.0 (ring *C*), 106.6 (py *C*), 58.4 (*C*S$_2$), 38.1 (N*C*H$_3$), 12.5 (Cp*C*H$_3$) ppm.

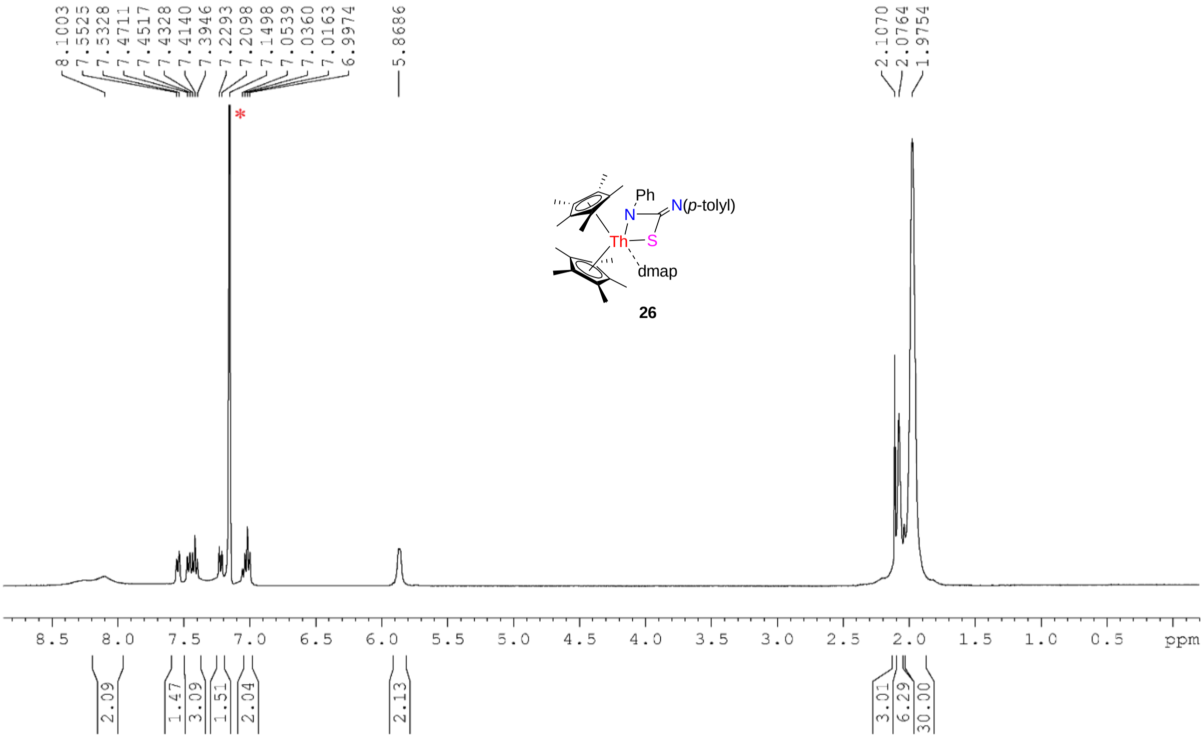

Figure S66. ^{1}H NMR (C_6D_6; 20 ℃) spectrum for compound (η^5-C_5Me_5)$_2$Th[SC=N(*p*-tolyl)NPh](dmap) (**26**) (* solvent). ^{1}H NMR (C_6D_6): δ 8.10 (br s, 2H, py), 7.54 (d, *J* = 7.8 Hz, 2H, tolyl), 7.43 (m, 3H, phenyl), 7.22 (d, *J* = 7.8 Hz, 2H, tolyl), 7.02 (m, 2H, phenyl), 5.87 (s, 2H, py), 2.11 (s, 3H, tolylCH_3), 2.08 (s, 6H, N(CH_3)$_2$), 1.98 (s, 30H, CpCH_3) ppm.

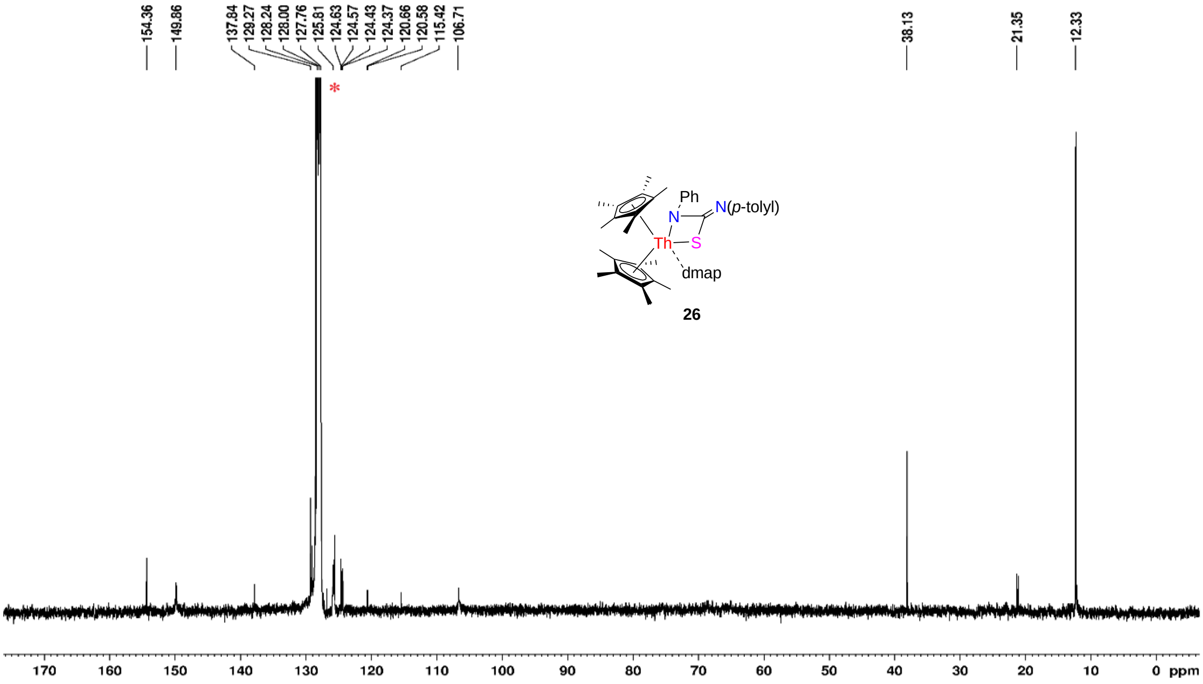

Figure S67. ^{13}C{^{1}H} NMR (C_6D_6; 20 ℃) spectrum for compound (η^5-C_5Me_5)$_2$Th[SC=N(*p*-tolyl)NPh](dmap) (**26**) (* solvent). ^{13}C{^{1}H} NMR (C_6D_6): δ 154.4 (py *C*), 149.9 (py *C*), 137.8 (phenyl *C*), 129.3 (phenyl *C*), 125.8 (ring *C*), 124.6 (phenyl *C*), 124.5 (phenyl *C*), 124.4 (phenyl *C*), 124.3 (phenyl *C*), 120.7 (phenyl *C*), 120.6 (phenyl *C*), 115.4 (*C*=N), 106.7 (py *C*), 38.1 (N*C*H_3), 21.4 (tolyl*C*H_3), 12.3 (Cp*C*H_3) ppm.

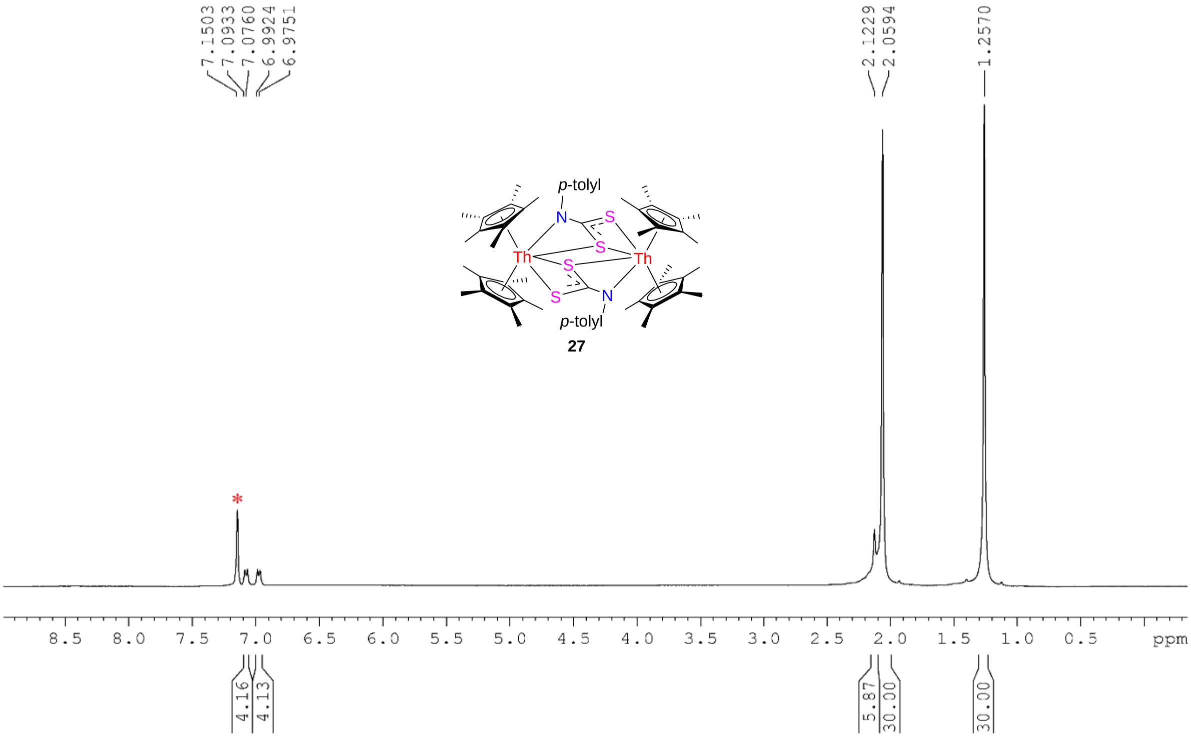

Figure S68. ^{1}H NMR (C_6D_6; 20 ℃) spectrum for compound $[(\eta^5\text{-}C_5Me_5)_2Th]_2\{\mu\text{-}[N(p\text{-tolyl})C(S)S]\}_2 \cdot C_6H_6$ (**27**$\cdot C_6H_6$) (* solvent). ^{1}H NMR (C_6D_6): δ 7.15 (s, 6H, C_6H_6), 7.08 (d, J = 6.9 Hz, 4H, phenyl), 6.98 (d, J = 6.9 Hz, 4H, phenyl), 2.12 (s, 6H, tolylCH_3), 2.06 (s, 30H, CpCH_3), 1.26 (s, 30H, CpCH_3) ppm.

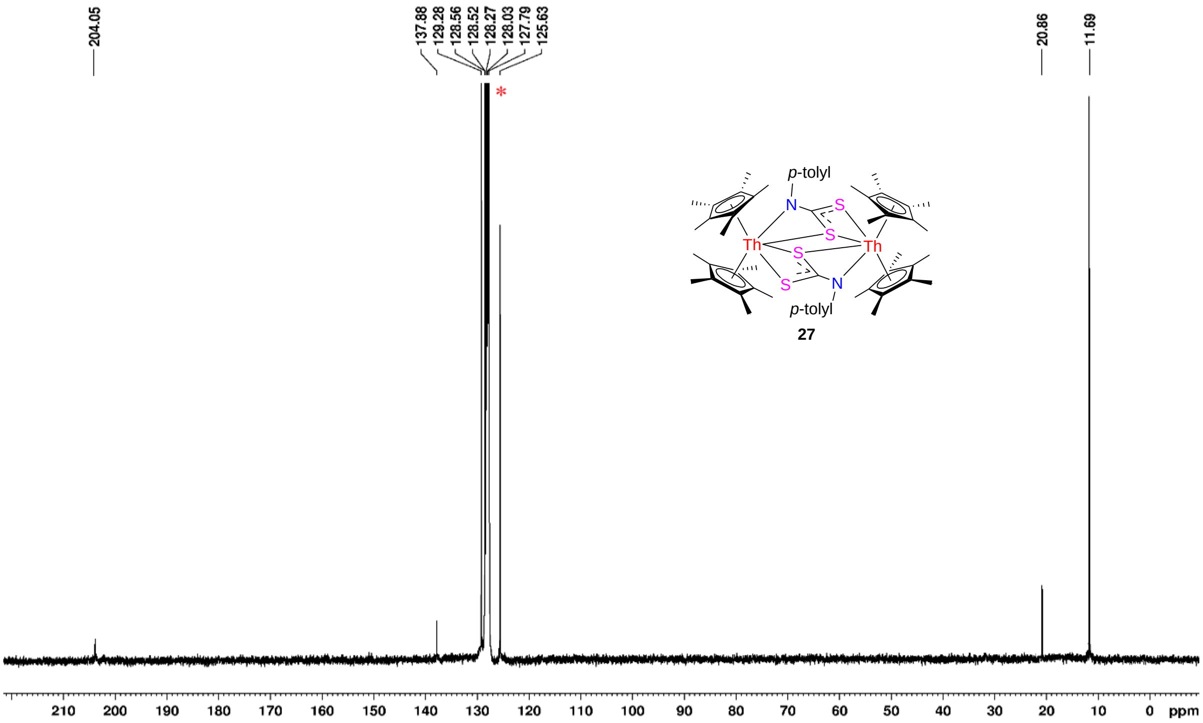

Figure S69. $^{13}C\{^{1}H\}$ NMR (C_6D_6; 20 ℃) spectrum for compound $[(\eta^5\text{-}C_5Me_5)_2Th]_2\{\mu\text{-}[N(p\text{-tolyl})C(S)S]\}_2 \cdot C_6H_6$ (**27**$\cdot C_6H_6$) (* solvent). $^{13}C\{^{1}H\}$ NMR (C_6D_6): δ 204.1 (CS_2), 137.9 (phenyl C), 129.3 (phenyl C), 128.6 (phenyl C), 128.5 (C_6H_6), 128.2 (phenyl C), 125.6 (ring C), 20.9 (tolylCH_3), 11.7 (CpCH_3) ppm.

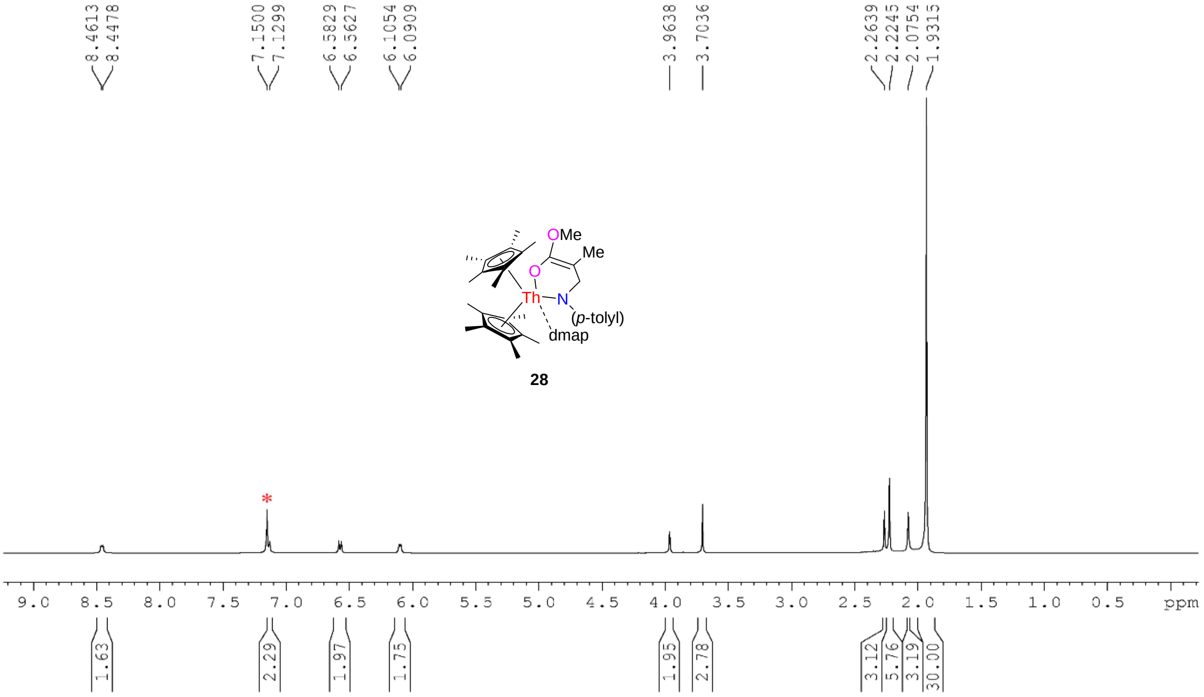

Figure S70. ^{1}H NMR (C_6D_6; 20 ℃) spectrum for compound (η^5-C_5Me_5)$_2$Th[N(*p*-tolyl)CH_2C(Me)=C(OMe)O](dmap) (**28**) (* solvent). ^{1}H NMR (C_6D_6): δ 8.45 (d, J = 5.4 Hz, 2H, py), 7.13 (d, 2H, J = 8.1 Hz, phenyl), 6.57 (d, 2H, J = 8.1 Hz, phenyl), 6.10 (d, J = 5.8 Hz, 2H, py), 3.96 (s, 2H, CH_2), 3.70 (s, 3H, OCH_3), 2.26 (s, 3H, CH_3), 2.22 (s, 6H, N(CH_3)$_2$), 2.08 (s, 3H, tolylCH_3), 1.93 (s, 30H, CpCH_3) ppm.

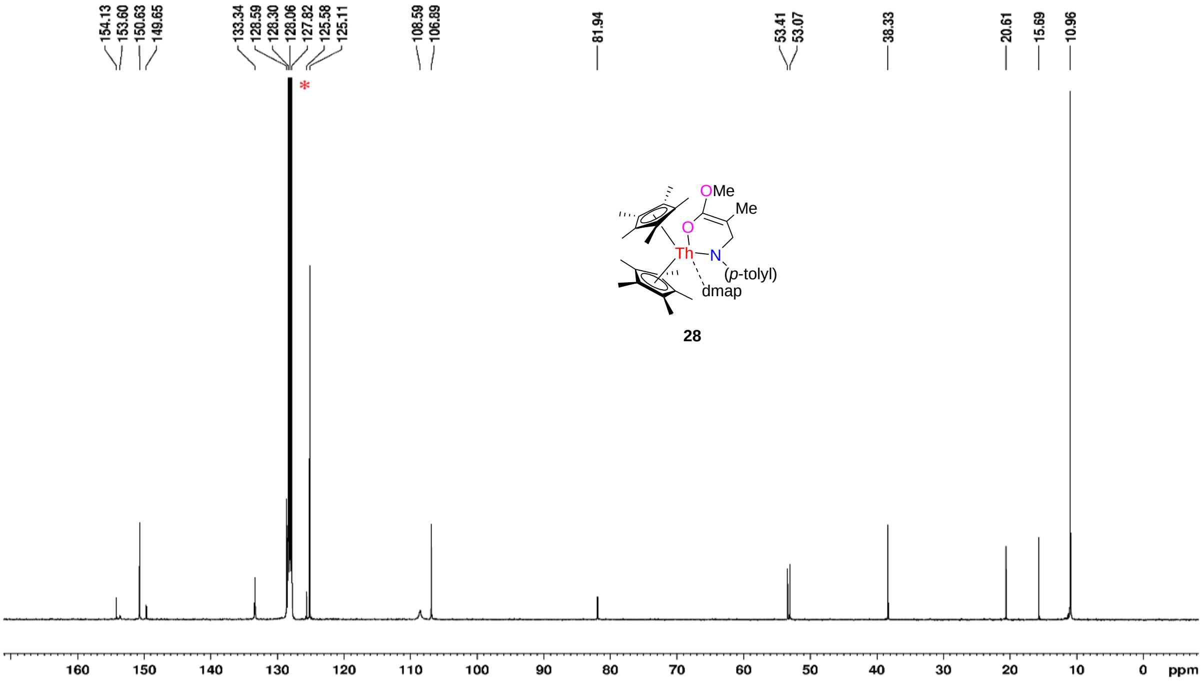

Figure S71. $^{13}C\{^{1}H\}$ NMR (C_6D_6; 20 ℃) spectrum for compound (η^5-C_5Me_5)$_2$Th[N(*p*-tolyl)CH_2C(Me)=C(OMe)O](dmap) (**28**) (* solvent). $^{13}C\{^{1}H\}$ NMR (C_6D_6): δ 154.1 (py *C*), 153.6 (phenyl *C*), 150.6 (py *C*), 149.7 (phenyl *C*), 133.3 (phenyl *C*), 128.6 (phenyl *C*), 125.6 (phenyl *C*), 125.1 (ring *C*), 108.6 (*C*O), 106.9 (py *C*), 81.9 (*C*=CO), 53.4 (O*C*H_3), 53.1 (*C*H_2), 38.3 (N*C*H_3), 20.6 (tolyl*C*H_3), 15.7 (*C*H_3), 11.0 (Cp*C*H_3) ppm.

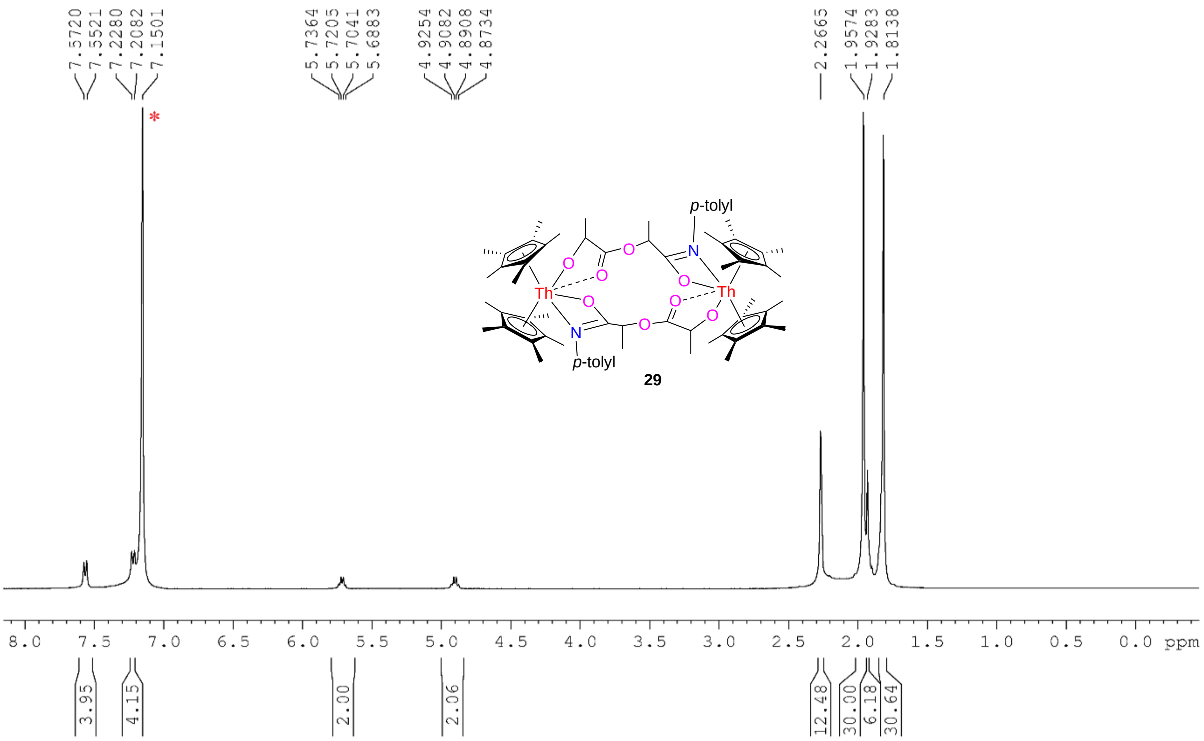

Figure S72. ^{1}H NMR (C_6D_6; 20 °C) spectrum for compound $[(\eta^5\text{-}C_5Me_5)_2Th]_2\{\mu$-[OCH(Me)C(=O)OCH(Me)C(=N-*p*-tolyl)O]$\}_2$ (**29**) (* solvent). ^{1}H NMR (C_6D_6): δ 7.56 (d, J = 8.0 Hz, 4H, phenyl), 7.21 (d, J = 8.0 Hz, 4H, phenyl), 5.71 (m, 2H, OC*H*), 4.90 (m, 2H, OC*H*), 2.27 (s, 12H, CH_3), 1.96 (s, 30H, CpCH_3), 1.93 (s, 6H, tolylCH_3), 1.81 (s, 30H, CpCH_3) ppm.

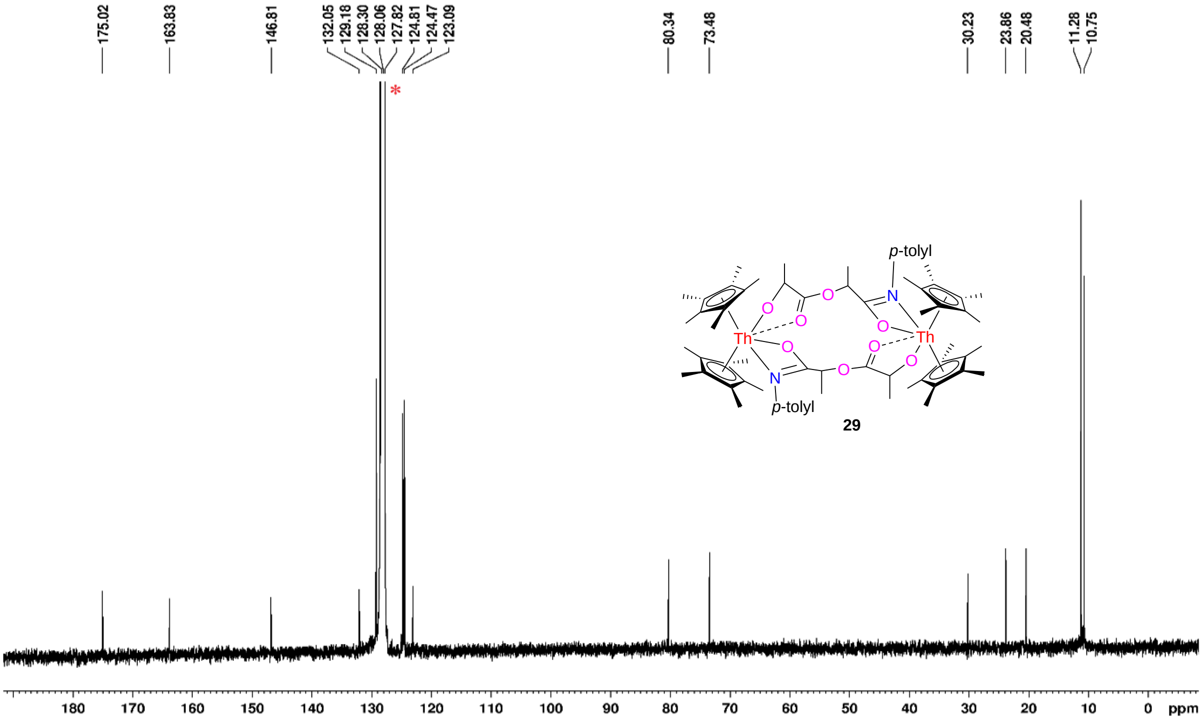

Figure S73. ^{13}C{^{1}H} NMR (C_6D_6; 20 °C) spectrum for compound $[(\eta^5\text{-}C_5Me_5)_2Th]_2\{\mu$-[OCH(Me)C(=O)OCH(Me)C(=N-*p*-tolyl)O]$\}_2$ (**29**) (* solvent). ^{13}C{^{1}H} NMR (C_6D_6): δ 175.0 (*C*O_2), 163.8 (*C*ON), 146.8 (phenyl *C*), 132.1 (phenyl *C*), 129.2 (phenyl *C*), 124.8 (ring *C*), 124.5 (ring *C*), 123.1 (phenyl *C*), 80.3 (O*C*H), 73.5 (O*C*H), 30.2 (*C*H_3), 23.9 (*C*H_3), 20.5 (tolyl*C*H_3), 11.3 (Cp*C*H_3), 10.8 (Cp*C*H_3) ppm.

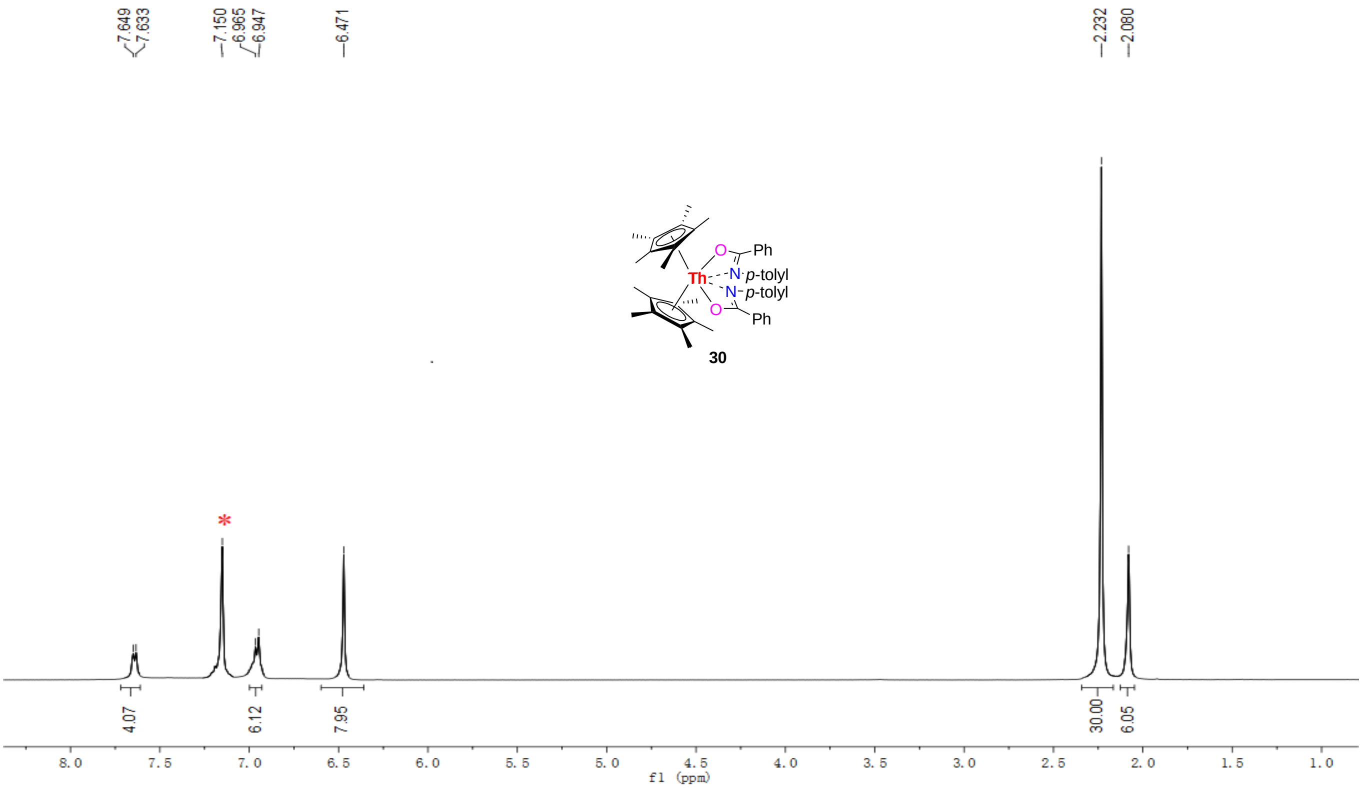

Figure S74. ^{1}H NMR (C_6D_6; 20 ℃) spectrum for compound (η^5-C_5Me_5)$_2$Th[OC(Ph)N(*p*-tolyl)]$_2$ (**30**) (* solvent). ^{1}H NMR (C_6D_6): δ 7.64 (d, J = 6.4 Hz, 4H, phenyl), 6.95 (m, 6H, phenyl), 6.47 (s, 8H, phenyl), 2.23 (s, 30H, CpCH_3), 2.08 (s, 6H, tolylCH_3) ppm.

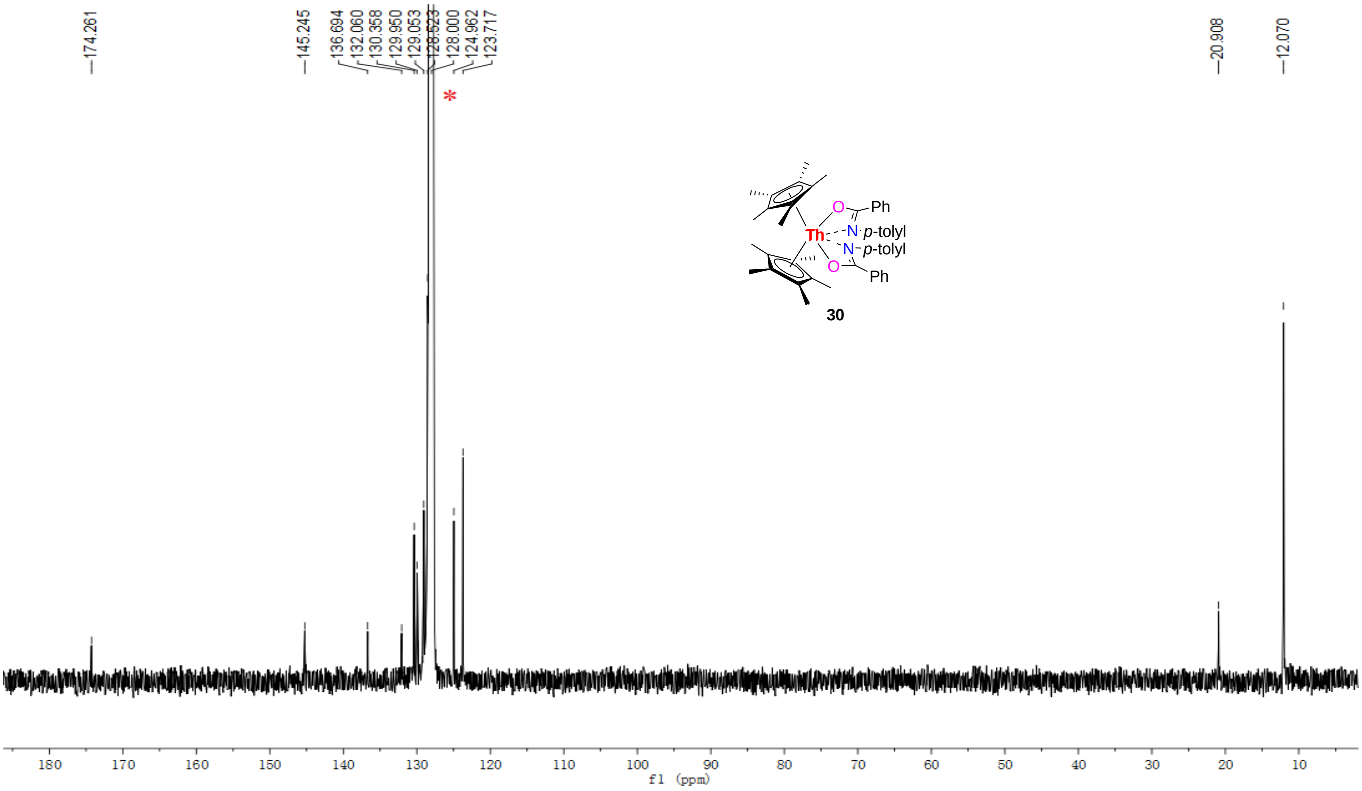

Figure S75. $^{13}C\{^{1}H\}$ NMR (C_6D_6; 20 ℃) spectrum for compound (η^5-C_5Me_5)$_2$Th[OC(Ph)N(*p*-tolyl)]$_2$ (**30**) (* solvent). $^{13}C\{^{1}H\}$ NMR (C_6D_6): δ 174.3 (*C*O), 145.2 (phenyl *C*), 136.7 (phenyl *C*), 132.1 (phenyl *C*), 130.4 (phenyl *C*), 130.0 (phenyl *C*), 129.1 (phenyl *C*), 128.5 (phenyl *C*), 125.0 (phenyl *C*), 123.7 (ring *C*), 20.9 (tolylCH_3), 12.1 (CpCH_3) ppm.

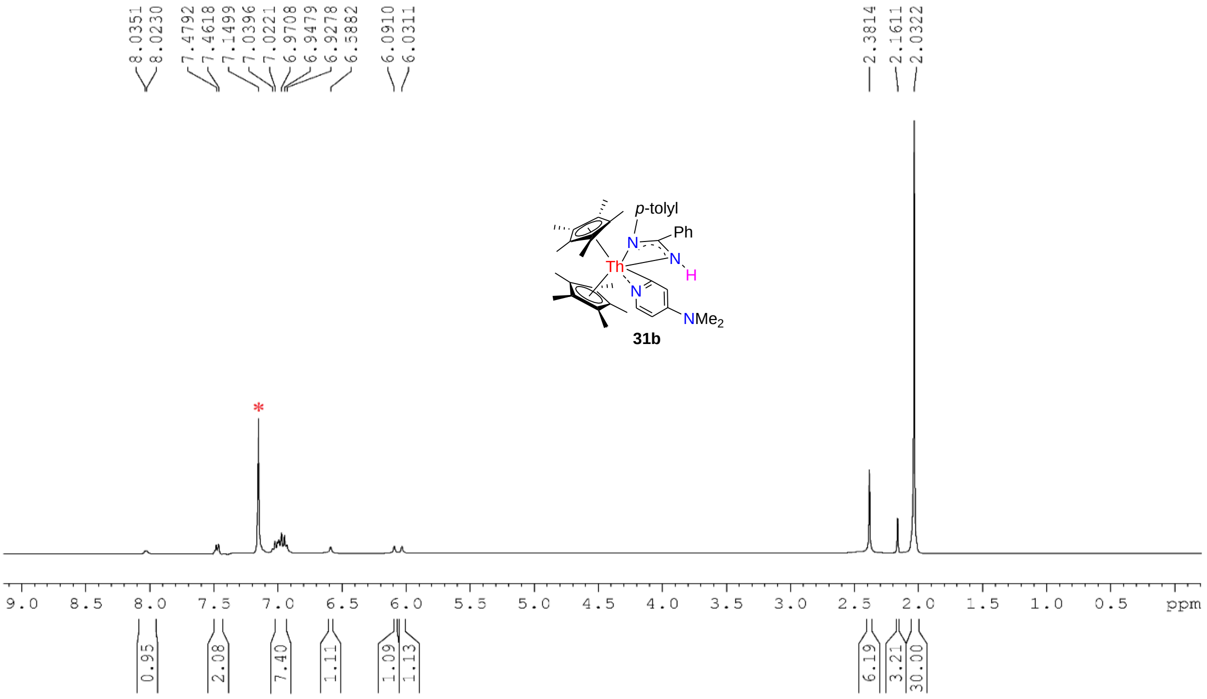

Figure S76. ^{1}H NMR (C_6D_6; 20 ℃) spectrum for compound (η^5-C_5Me_5)$_2$Th[η^3-NHC(Ph)N(*p*-tolyl)][κ^2-*C*,*N*-4-(Me_2N)C_5H_3N] (**31b**) (* solvent). ^{1}H NMR (C_6D_6): δ 8.03 (d, J = 4.8 Hz, 1H, py), 7.47 (d, J = 7.0 Hz, 2H, phenyl), 6.97 (m, 7H, phenyl), 6.59 (s, 1H, py), 6.09 (s, 1H, py), 6.03 (s, 1H, N*H*), 2.38 (s, 6H, N(C*H*$_3$)$_2$), 2.16 (s, 3H, tolylC*H*$_3$), 2.03 (s, 30H, CpC*H*$_3$) ppm.

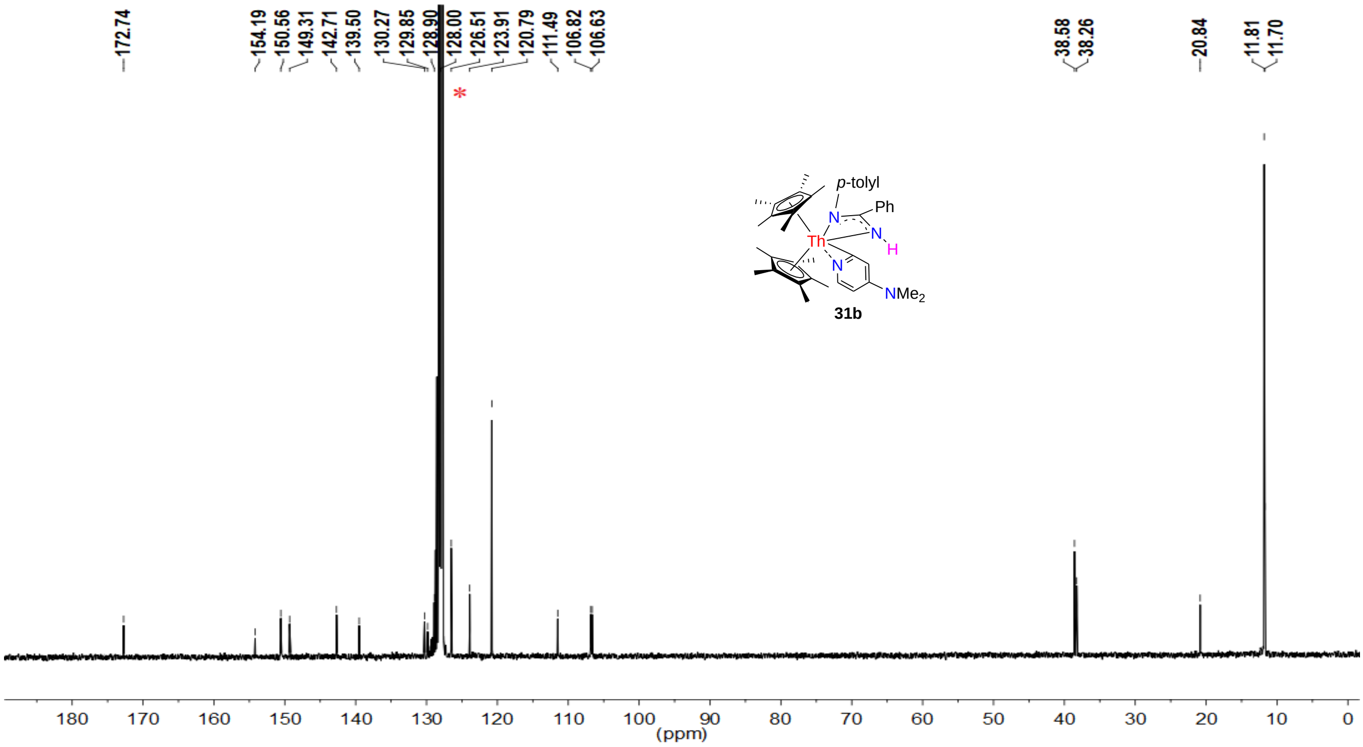

Figure S77. ^{13}C{^{1}H} NMR (C_6D_6; 20 ℃) spectrum for compound (η^5-C_5Me_5)$_2$Th[η^3-NHC(Ph)N(*p*-tolyl)][κ^2-*C*,*N*-4-(Me_2N)C_5H_3N] (**31b**) (* solvent). ^{13}C{^{1}H} NMR (C_6D_6): δ 172.7 (Th*C*), 154.2 (aryl *C*), 150.6 (aryl *C*), 149.3 (aryl *C*), 142.7 (aryl *C*), 139.5 (aryl *C*), 130.3 (aryl *C*), 129.9 (aryl *C*), 128.9 (aryl *C*), 126.5 (aryl *C*), 123.9 (aryl *C*), 120.8 (ring *C*), 111.5 (aryl *C*), 106.8 (aryl *C*), 106.6 (*C*=N), 38.6 (N*C*H$_3$), 38.3 (N*C*H$_3$), 20.8 (tolyl*C*H$_3$), 11.8 (Cp*C*H$_3$), 11.7 (Cp*C*H$_3$) ppm.

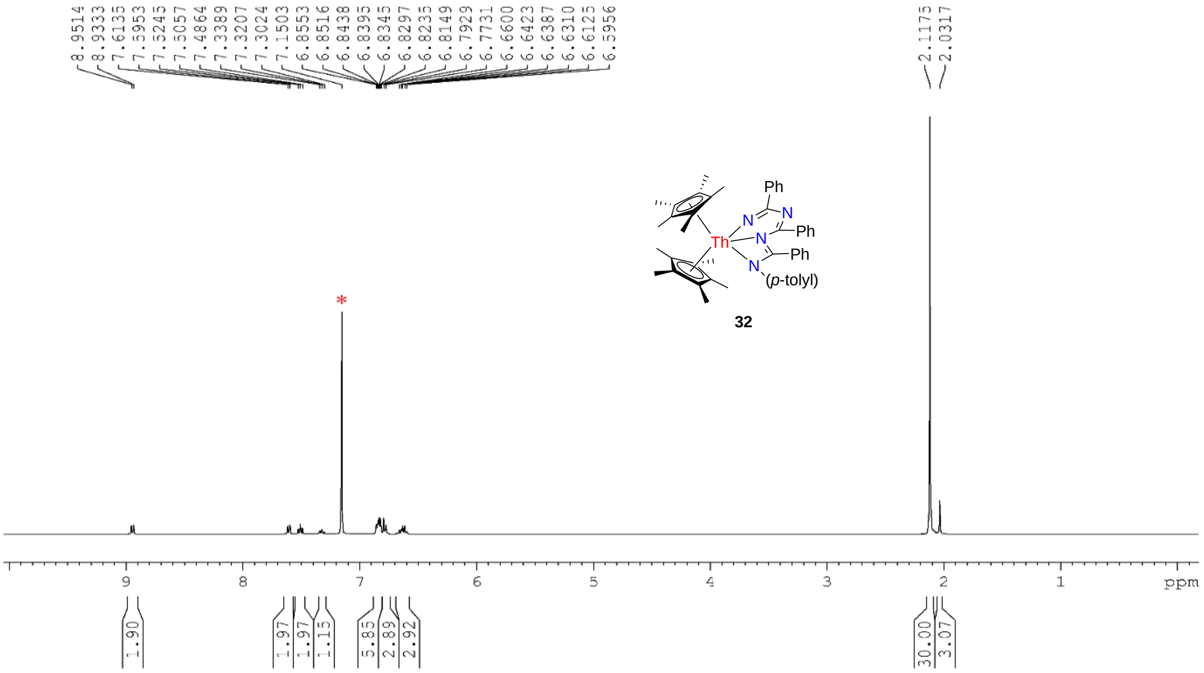

Figure S78. ^{1}H NMR (C_6D_6; 20 ℃) spectrum for compound (η^5-C_5Me_5)$_2$Th[η^7-N(*p*-tolyl)C(Ph)NC(Ph)NC(Ph)N] C_6H_6 (**32** C_6H_6) (* solvent). ^{1}H NMR (C_6D_6): δ 8.94 (d, J = 7.2 Hz, 2H, phenyl), 7.60 (d, J = 7.3 Hz, 2H, phenyl), 7.51 (t, J = 7.6 Hz, 2H, phenyl), 7.32 (t, J = 7.3 Hz, 1H, phenyl), 7.15 (s, 6H, C_6H_6), 6.83 (m, 6H, phenyl), 6.79 (m, 3H, phenyl), 6.63 (m, 3H, phenyl), 2.12 (s, 30H, CpCH_3), 2.03 (s, 3H, tolylCH_3) ppm.

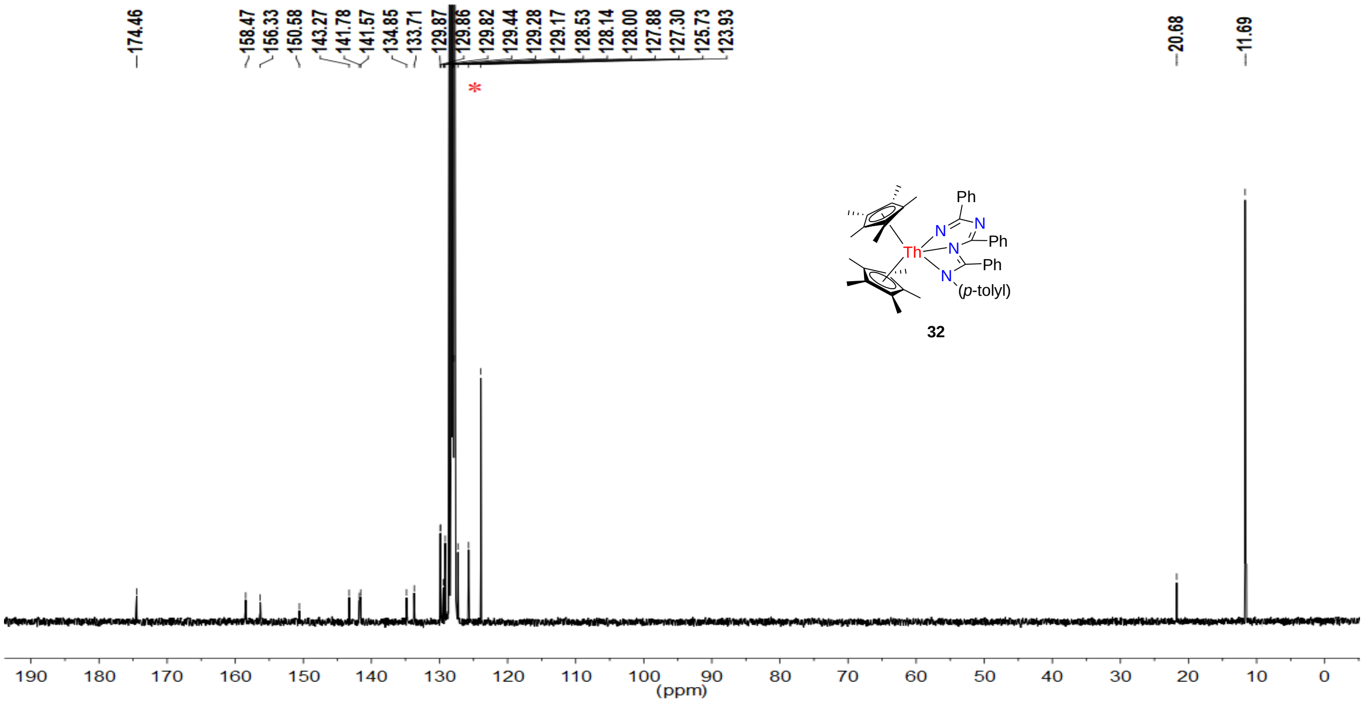

Figure S79. ^{13}C{^{1}H} NMR (C_6D_6; 20 ℃) spectrum for compound (η^5-C_5Me_5)$_2$Th[η^7-N(*p*-tolyl)C(Ph)NC(Ph)NC(Ph)N] C_6H_6 (**32** C_6H_6) (* solvent). ^{13}C{^{1}H} NMR (C_6D_6): δ 174.5 (ThN=*C*), 158.5 (phenyl *C*), 156.3 (phenyl *C*), 150.6 (phenyl *C*), 143.3 (phenyl *C*), 141.8 (phenyl *C*), 141.6 (phenyl *C*), 134.9 (phenyl *C*), 133.7 (phenyl *C*), 129.87 (phenyl *C*), 129.86 (phenyl *C*), 129.82 (phenyl *C*), 129.4 (phenyl *C*), 129.3 (phenyl *C*), 129.2 (phenyl *C*), 128.5 (C_6H_6), 128.1 (phenyl *C*), 127.9 (phenyl *C*), 127.3 (*C*=N), 125.7 (*C*=N), 123.9 (ring *C*), 20.7 (tolylCH_3), 11.7 (CpCH_3) ppm.

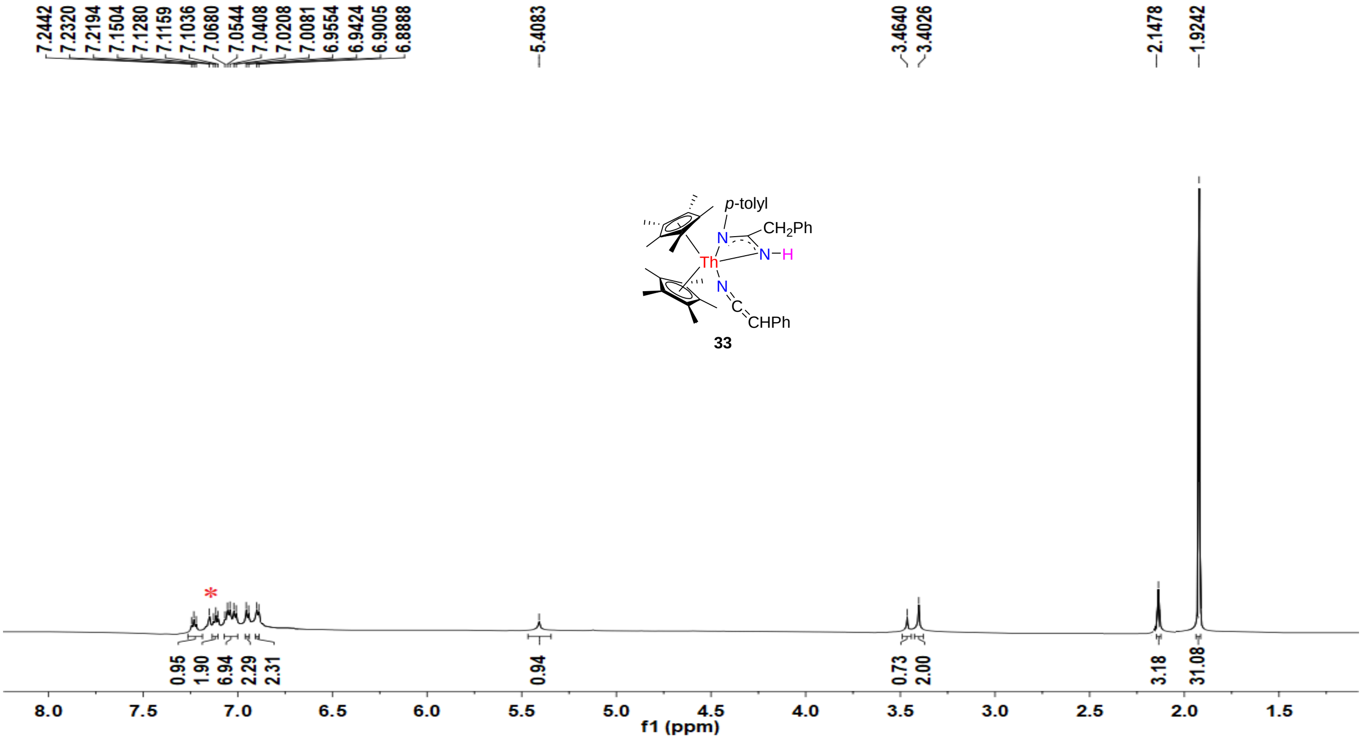

Figure S80. ^{1}H NMR (C_6D_6; 20 ℃) spectrum for compound (η^5-C_5Me_5)$_2$Th[η^3-N(*p*-tolyl)C(CH_2Ph)NH](N=C=CHPh) (**33**) (* solvent). ^{1}H NMR (C_6D_6): δ 7.23 (t, *J* = 7.4 Hz, 1H, phenyl), 7.12 (d, *J* = 7.2 Hz, 2H, phenyl), 7.04 (m, 7H, phenyl), 6.95 (d, *J* = 7.8 Hz, 2H, phenyl), 6.89 (d, *J* = 7.0 Hz, 2H, phenyl), 5.41 (s, 1H, C=C*H*), 3.46 (s, 1H, N*H*), 3.40 (s, 2H, C*H*$_2$), 2.15 (s, 3H, tolylC*H*$_3$), 1.92 (s, 30H, CpC*H*$_3$) ppm.

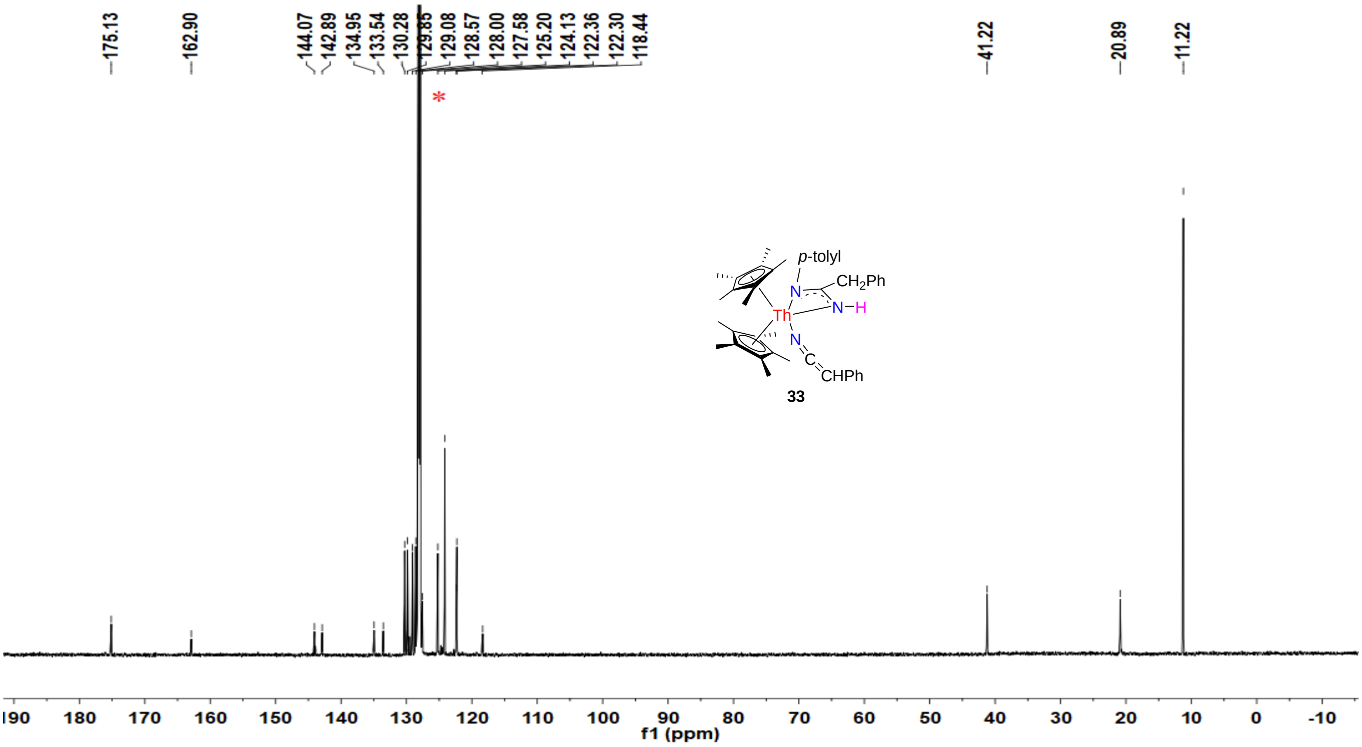

Figure S81. ^{13}C{^{1}H} NMR (C_6D_6; 20 ℃) spectrum for compound (η^5-C_5Me_5)$_2$Th[η^3-N(*p*-tolyl)C(CH_2Ph)NH](N=C=CHPh) (**33**) (* solvent). ^{13}C{^{1}H} NMR (C_6D_6): δ 175.1(*C*=NH), 162.9 (*C*=N), 144.1 (phenyl *C*), 142.9 (phenyl *C*), 135.0 (phenyl *C*), 133.5 (phenyl *C*), 130.3 (phenyl *C*), 129.9 (phenyl *C*), 129.1 (phenyl *C*), 128.6 (phenyl *C*), 127.6 (phenyl *C*), 125.2 (phenyl *C*), 124.1 (ring *C*), 122.4 (phenyl *C*), 122.3 (phenyl *C*), 118.4 (H*C*=C), 41.2 (*C*H_2), 20.9 (tolyl*C*H_3), 11.2 (Cp*C*H_3) ppm.

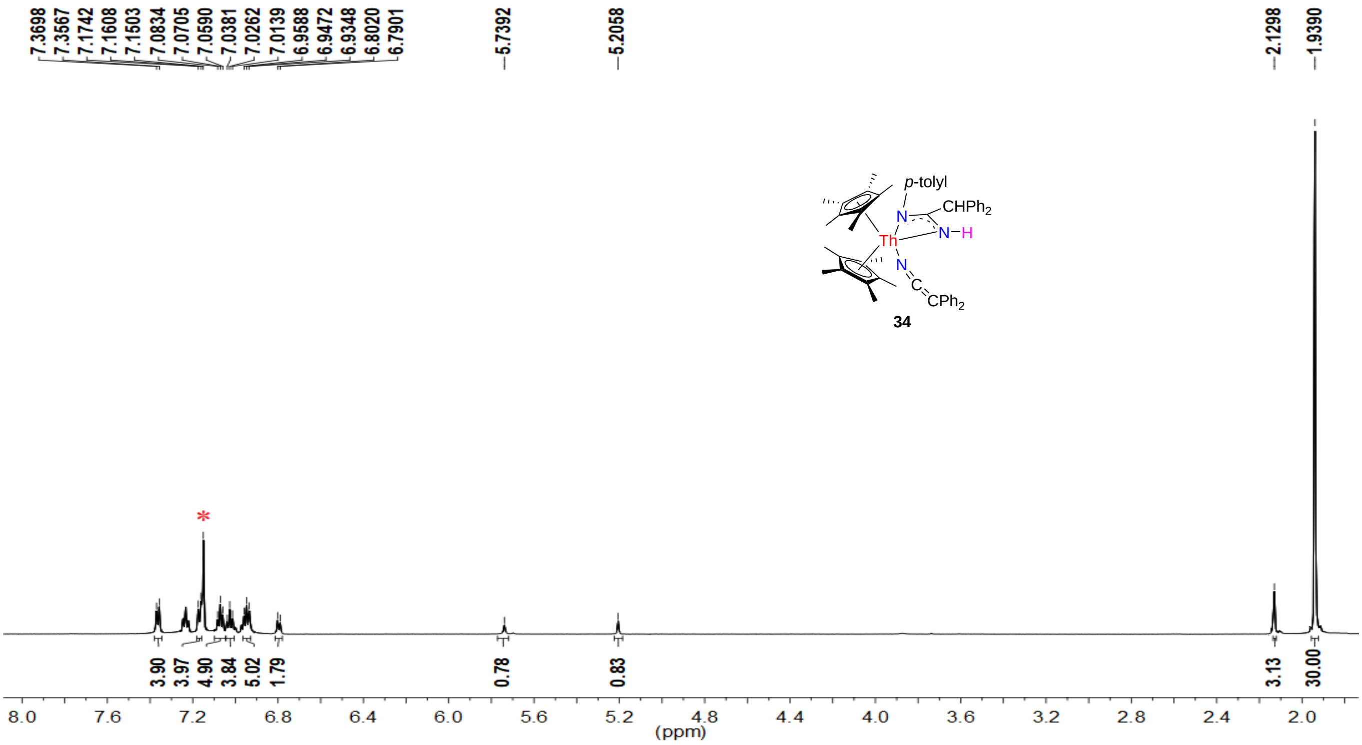

Figure S82. ^{1}H NMR (C_6D_6; 20 °C) spectrum for compound (η^5-C_5Me_5)$_2$Th[η^3-N(*p*-tolyl)C(CHPh$_2$)NH](N=C=CPh$_2$) (**34**) (* solvent). ^{1}H NMR (C_6D_6): δ 7.36 (d, J = 7.9 Hz, 4H, phenyl), 7.16 (d, J = 8.0 Hz, 4H, phenyl), 7.07 (m, 5H, phenyl), 7.03 (t, J = 7.2 Hz, 4H, phenyl), 6.94 (m, 5H, phenyl), 6.80 (d, J = 7.2 Hz, 2H, phenyl), 5.74 (s, 1H, N*H*), 5.21 (s, 1H, C*H*), 2.13 (s, 3H, tolylC*H*$_3$), 1.94 (s, 30H, CpC*H*$_3$) ppm.

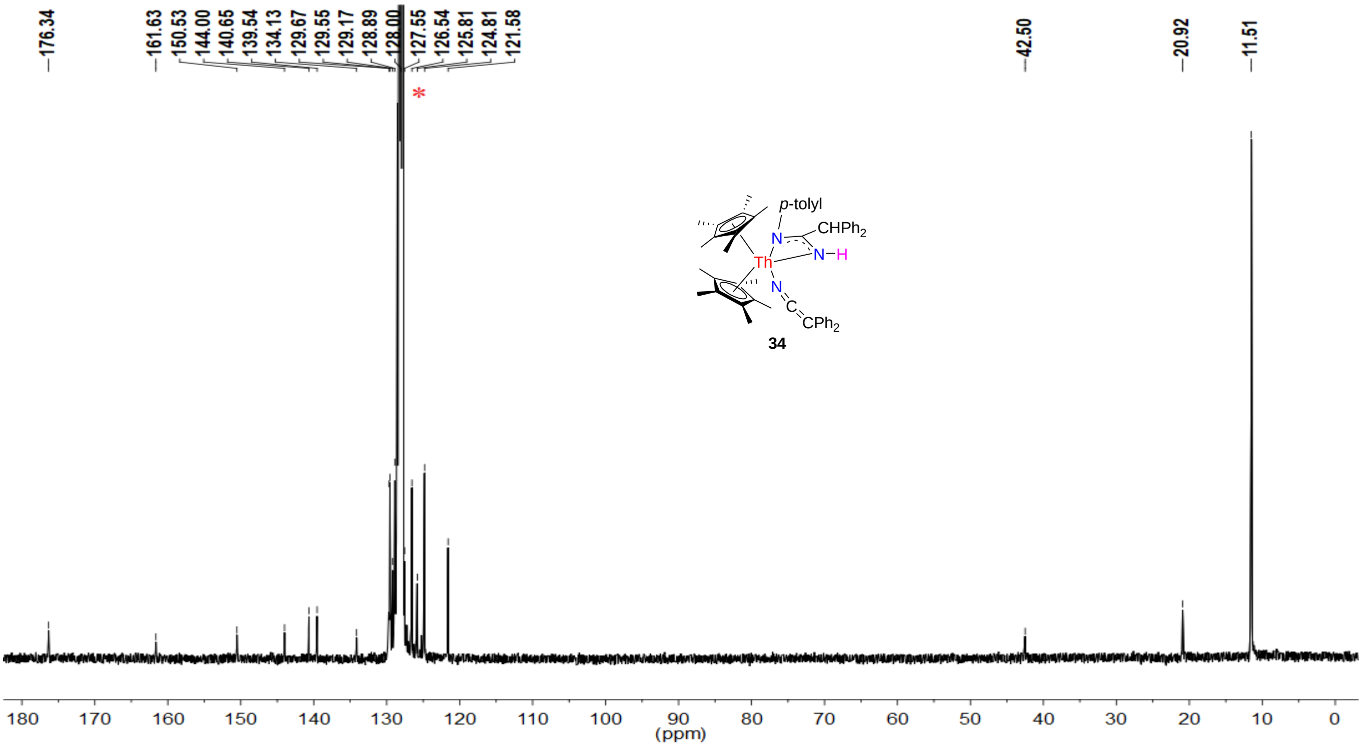

Figure S83. ^{13}C{^{1}H} NMR (C_6D_6; 20 °C) spectrum for compound (η^5-C_5Me_5)$_2$Th[η^3-N(*p*-tolyl)C(CHPh$_2$)NH](N=C=CPh$_2$) (**34**) (* solvent). ^{13}C{^{1}H} NMR (C_6D_6): δ 176.3 (*C*=NH), 161.6 (*C*=N), 150.5 (phenyl *C*), 144.0 (phenyl *C*), 140.7 (phenyl *C*), 139.5 (phenyl *C*), 134.1 (phenyl *C*), 129.7 (phenyl *C*), 129.6 (phenyl *C*), 129.2 (phenyl *C*), 128.9 (phenyl *C*), 127.6 (phenyl *C*), 126.5 (phenyl *C*), 125.8 (phenyl *C*), 124.8 (ring *C*), 121.6 (Ph$_2$*C*=C), 42.5 (*C*H), 20.9 (tolyl*C*H$_3$), 11.5 (Cp*C*H$_3$) ppm.

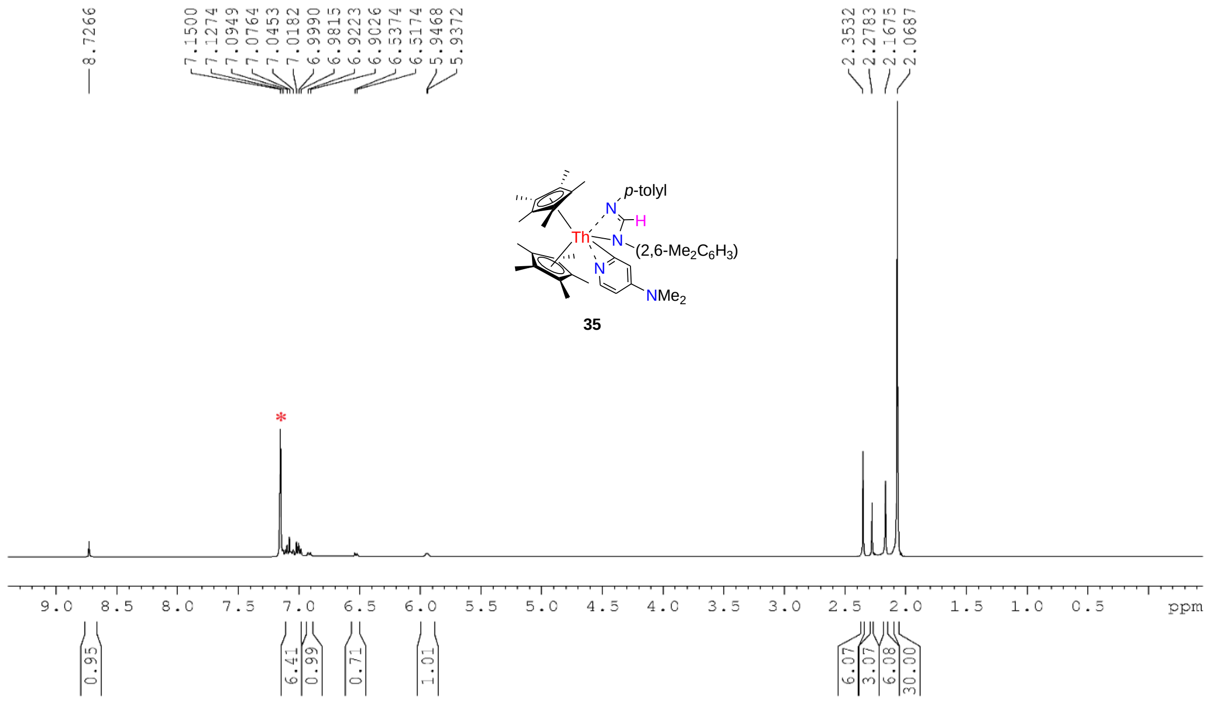

Figure S84. ^{1}H NMR (C_6D_6; 20 ℃) spectrum for compound (η^5-C_5Me_5)$_2$Th[N(*p*-tolyl)=CHN(2,6-$Me_2C_6H_3$)][κ^2-*C*,*N*-4-(Me_2N)C_5H_3N] 0.5C_6H_6 (**35** 0.5C_6H_6) (* solvent). ^{1}H NMR (C_6D_6): δ 8.73 (s, 1H, C*H*=N), 7.15 (s, 3H, C_6*H*$_6$), 7.12-6.98 (m, 7H, phenyl and py), 6.91 (d, *J* = 8.0 Hz, 1H, phenyl), 6.53 (d, *J* = 8.0 Hz, 1H, py), 5.94 (d, *J* = 3.8 Hz, 1H, py), 2.35 (s, 6H, N(C*H*$_3$)$_2$), 2.28 (s, 3H, tolylC*H*$_3$), 2.17 (s, 6H, arylC*H*$_3$), 2.07 (s, 30H, CpC*H*$_3$) ppm.

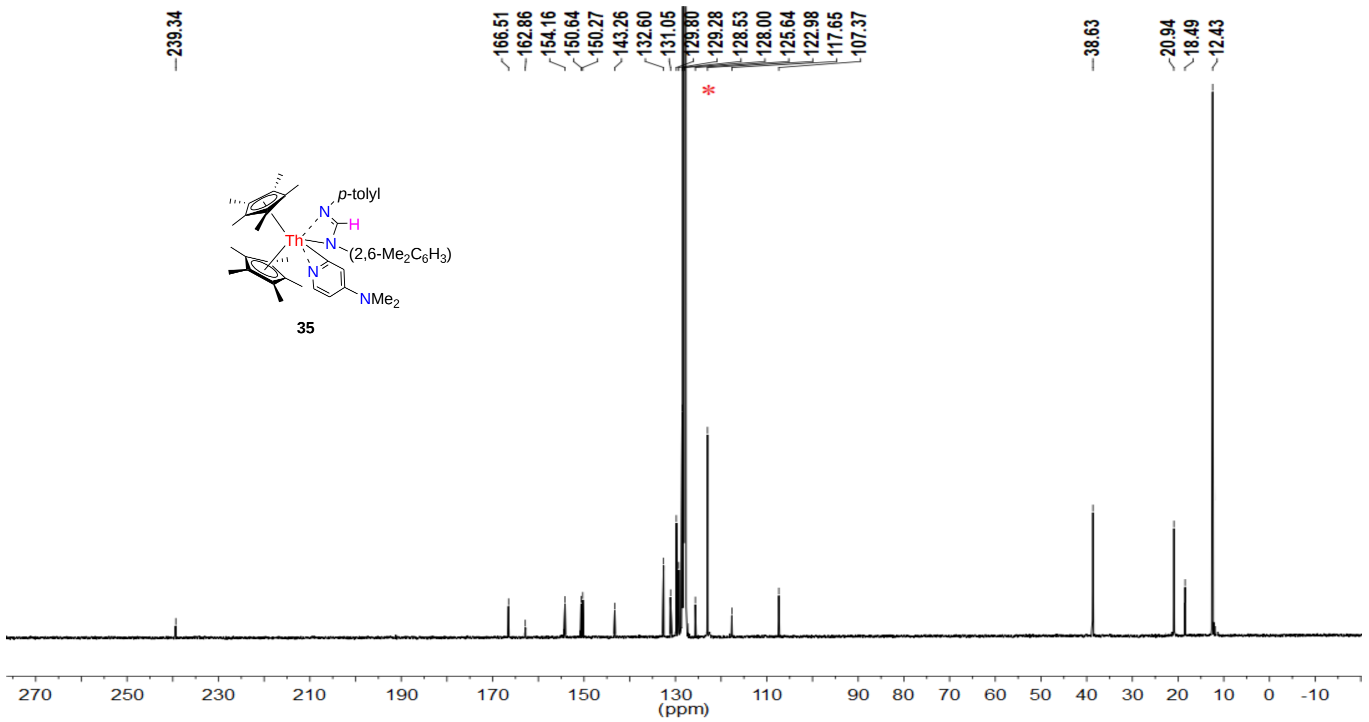

Figure S85. ^{13}C{^{1}H} NMR (C_6D_6; 20 ℃) spectrum for compound (η^5-C_5Me_5)$_2$Th[N(*p*-tolyl)=CHN(2,6-$Me_2C_6H_3$)][κ^2-*C*,*N*-4-(Me_2N)C_5H_3N] 0.5C_6H_6 (**35** 0.5C_6H_6) (* solvent). ^{13}C{^{1}H} NMR (C_6D_6): δ 239.3 (Th*C*), 166.5 (*C*H=N), 162.9 (aryl *C*), 154.2 (aryl *C*), 150.6 (aryl *C*), 150.3 (aryl *C*), 143.3 (aryl *C*), 132.6 (aryl *C*), 131.1 (aryl *C*), 129.8 (aryl *C*), 129.3 (aryl *C*), 128.5 (*C*$_6$H$_6$), 125.6 (aryl *C*), 123.0 (ring *C*), 117.7 (aryl *C*), 107.4 (aryl *C*), 38.6 (N*C*H$_3$), 20.9 (tolyl*C*H$_3$), 18.5 (aryl*C*H$_3$), 12.4 (Cp*C*H$_3$) ppm.

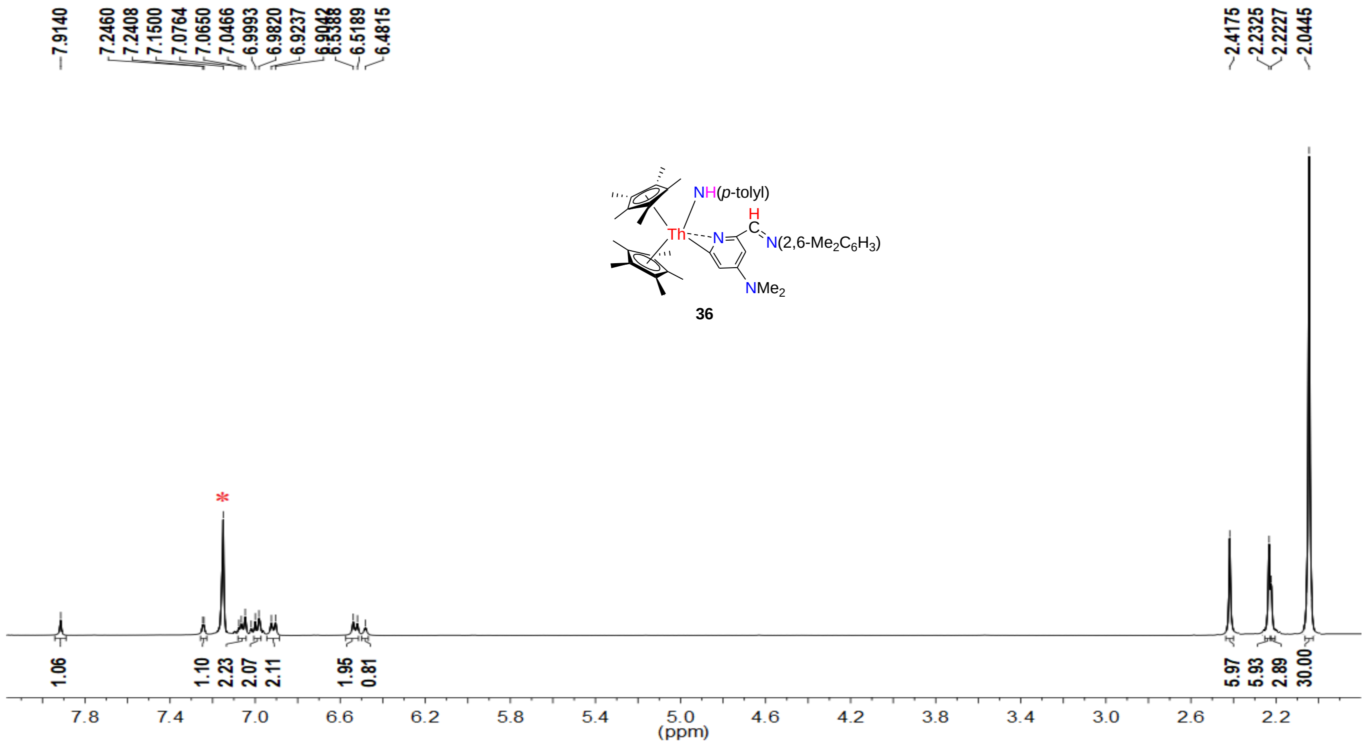

Figure S86. ^{1}H NMR (C_6D_6; 20 ℃) spectrum for compound (η^5-C_5Me_5)$_2$Th[NH(*p*-tolyl)][κ^2-*C*,*N*-4-(Me_2N)-6-(2,6-$Me_2C_6H_3N$=CH)C_5H_2N] (**36**) (* solvent). ^{1}H NMR (C_6D_6): δ 7.91 (s, 1H, C*H*=N), 7.24 (d, *J* = 1.8 Hz, 1H, py), 7.06 (m, 2H, aryl), 6.99 (m, 2H, aryl), 6.91 (d, *J* = 7.8 Hz, 2H, phenyl), 6.53 (d, *J* = 7.8 Hz, 2H, phenyl), 6.48 (s, 1H, N*H*), 2.42 (s, 6H, N(CH_3)$_2$), 2.23 (s, 6H, arylCH_3), 2.22 (s, 3H, tolylCH_3), 2.04 (s, 30H, CpCH_3) ppm.

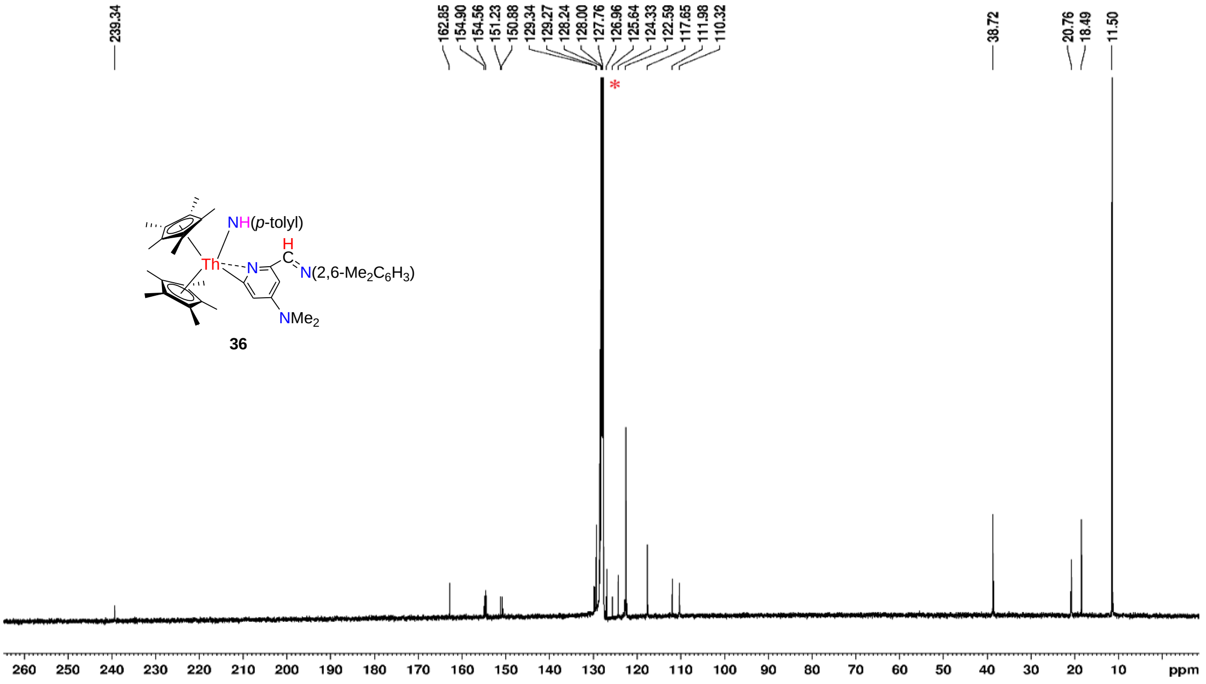

Figure S87. ^{13}C{^{1}H} NMR (C_6D_6; 20 ℃) spectrum for compound (η^5-C_5Me_5)$_2$Th[NH(*p*-tolyl)][κ^2-*C*,*N*-4-(Me_2N)-6-(2,6-$Me_2C_6H_3N$=CH)C_5H_2N] (**36**) (* solvent). ^{13}C{^{1}H} NMR (C_6D_6): δ 239.3 (Th*C*), 162.9 (*C*H=N), 154.9 (aryl *C*), 154.6 (aryl *C*), 151.2 (aryl *C*), 150.9 (aryl *C*), 129.3 (aryl *C*), 129.2 (aryl *C*), 127.0 (aryl *C*), 125.6 (aryl *C*), 124.3 (aryl *C*), 122.6 (ring *C*), 117.7 (aryl *C*), 112.0 (aryl *C*), 110.3 (aryl *C*), 38.7 (N*C*H_3), 20.8 (tolyl*C*H_3), 18.5 (aryl*C*H_3), 11.5 (Cp*C*H_3) ppm.

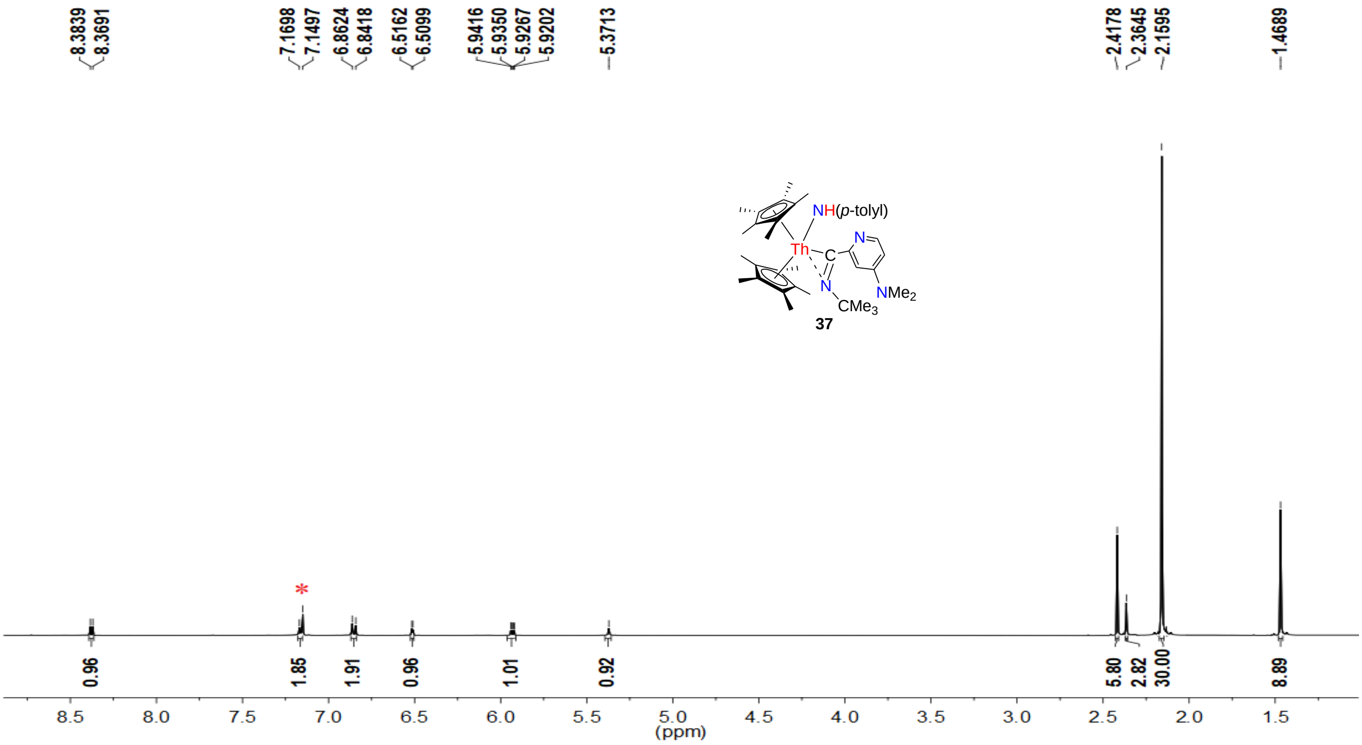

Figure S88. ^{1}H NMR (C_6D_6; 20 ℃) spectrum for compound (η^5-C_5Me_5)$_2$Th[NH(*p*-tolyl)][κ^2-*C*,*N*-2-(Me_3CNC)-4-(Me_2N)C_5H_3N] (**37**) (* solvent). ^{1}H NMR (C_6D_6): δ 8.38 (d, *J* = 5.9 Hz, 1H, py), 7.16 (d, *J* = 8.2 Hz, 2H, phenyl), 6.85 (d, *J* = 8.2 Hz, 2H, phenyl), 6.51 (d, *J* = 2.5 Hz, 1H, py), 5.93 (m, 1H, py), 5.37 (s, 1H, N*H*), 2.42 (s, 6H, N(CH_3)$_2$), 2.36 (s, 3H, tolylCH_3), 2.16 (s, 30H, CpCH_3), 1.47 (s, 9H, C(CH_3)$_3$) ppm.

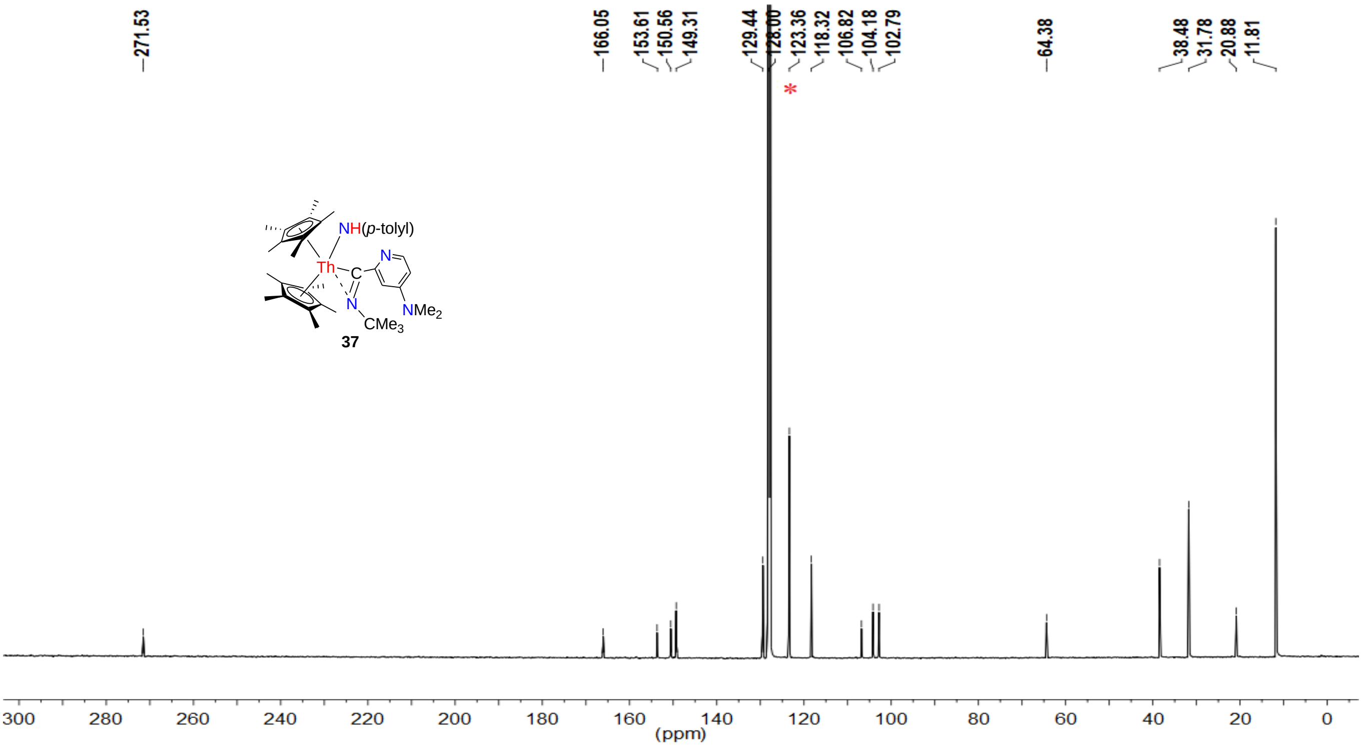

Figure S89. ^{13}C{^{1}H} NMR (C_6D_6; 20 ℃) spectrum for compound (η^5-C_5Me_5)$_2$Th[NH(*p*-tolyl)][κ^2-*C*,*N*-2-(Me_3CNC)-4-(Me_2N)C_5H_3N] (**37**) (* solvent). ^{13}C{^{1}H} NMR (C_6D_6): δ 271.5 (Th*C*), 166.1 (py *C*), 153.6 (py *C*), 150.6 (py *C*), 149.3 (phenyl *C*), 129.4 (phenyl *C*), 123.4 (ring *C*), 118.3 (phenyl *C*), 106.8 (phenyl *C*), 104.2 (py *C*), 102.8 (py *C*), 64.4 (*C*(CH_3)), 38.5 (N*C*H_3), 31.8 (C(*C*H_3)$_3$), 20.9 (tolyl*C*H_3), 11.8 (Cp*C*H_3) ppm.

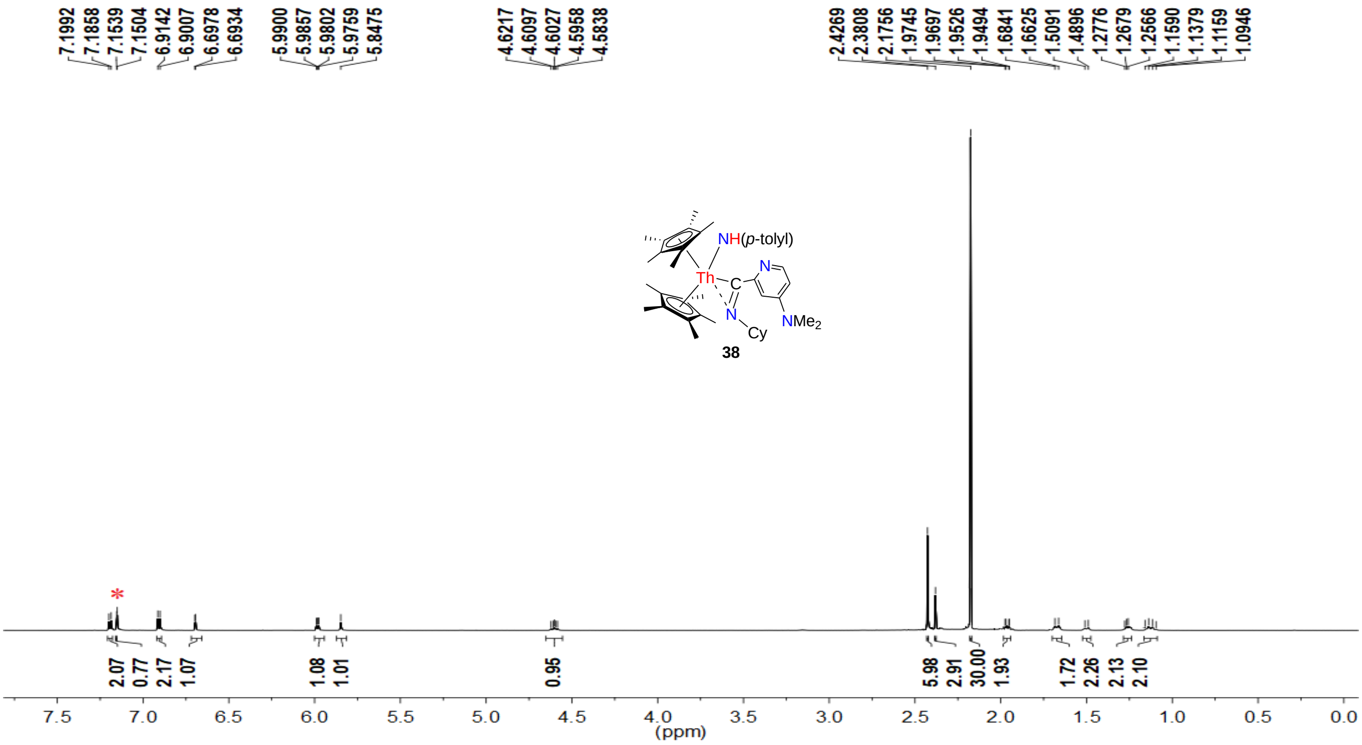

Figure S90. ^{1}H NMR (C_6D_6; 20 ℃) spectrum for compound (η^5-C_5Me_5)$_2$Th[NH(*p*-tolyl)][κ^2-*C*,*N*-2-(C_6H_{11}NC)-4-(Me_2N)C_5H_3N] (**38**) (* solvent). ^{1}H NMR (C_6D_6): δ 7.19 (d, *J* = 8.1 Hz, 2H, phenyl), 7.154 (s, 1H, py), 6.91 (d, *J* = 8.1 Hz, 2H, phenyl), 6.70 (d, *J* = 2.6 Hz, 1H, py), 5.98 (m, 1H, py), 5.85 (s, 1H, N*H*), 4.60 (m, 1H, C*H*), 2.43 (s, 6H, N(CH_3)$_2$), 2,38 (s, 3H, tolylCH_3), 2.18 (s, 30H, CpCH_3), 1.96 (m, 2H, CH_2), 1.67 (m, 2H, CH_2), 1.50 (m, 2H, CH_2), 1.26 (m, 2H, CH_2), 1.13 (m, 2H, CH_2) ppm.

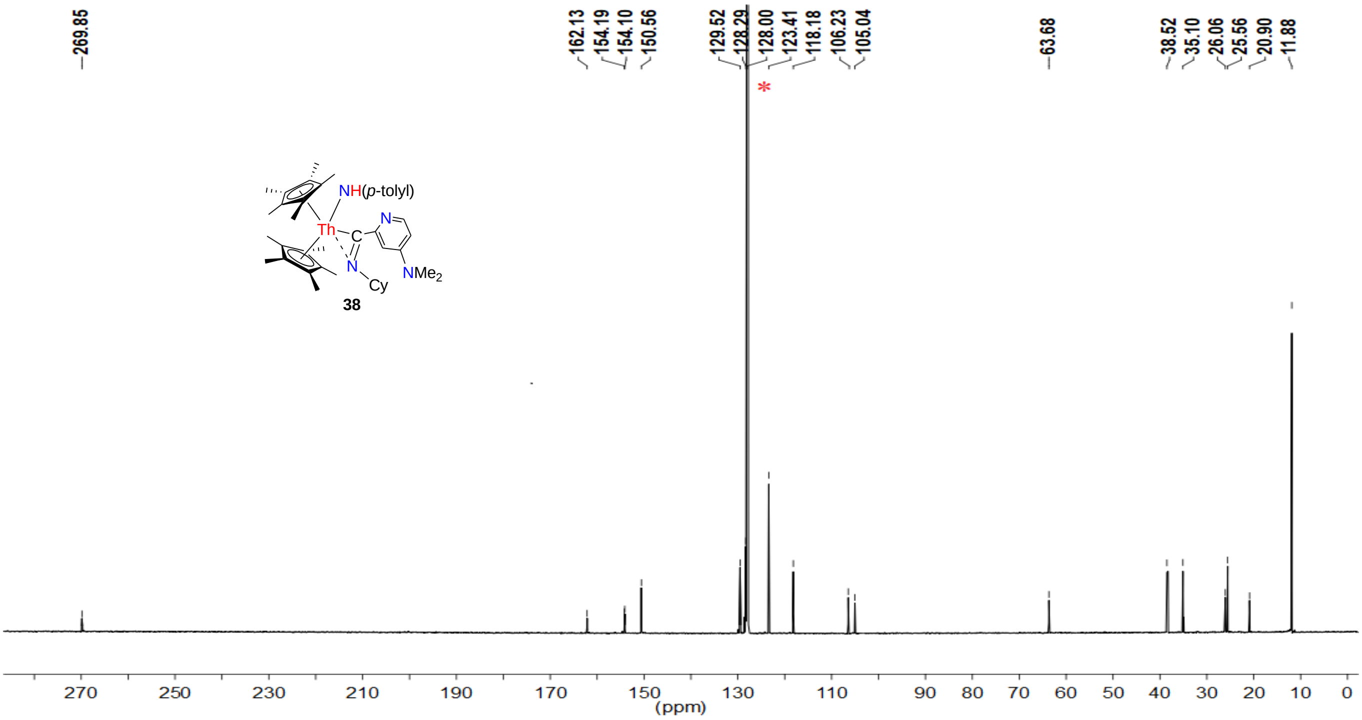

Figure S91. ^{13}C{^{1}H} NMR (C_6D_6; 20 ℃) spectrum for compound (η^5-C_5Me_5)$_2$Th[NH(*p*-tolyl)][κ^2-*C*,*N*-2-(C_6H_{11}NC)-4-(Me_2N)C_5H_3N] (**38**) (* solvent). ^{13}C{^{1}H} NMR (C_6D_6): δ 269.9 (Th*C*), 162.1 (py *C*), 154.2 (py *C*), 154.1 (py *C*), 150.6 (phenyl *C*), 129.5 (phenyl *C*), 128.3 (phenyl *C*), 123.4 (ring *C*), 118.2 (phenyl *C*), 106.2 (py *C*), 105.0 (py *C*), 63.7 (Cy *C*), 38.5 (N*C*H_3), 35.1 (Cy *C*), 26.1 (Cy *C*), 25.6 (Cy *C*), 20.9 (tolyl*C*H_3), 11.9 (Cp*C*H_3) ppm.

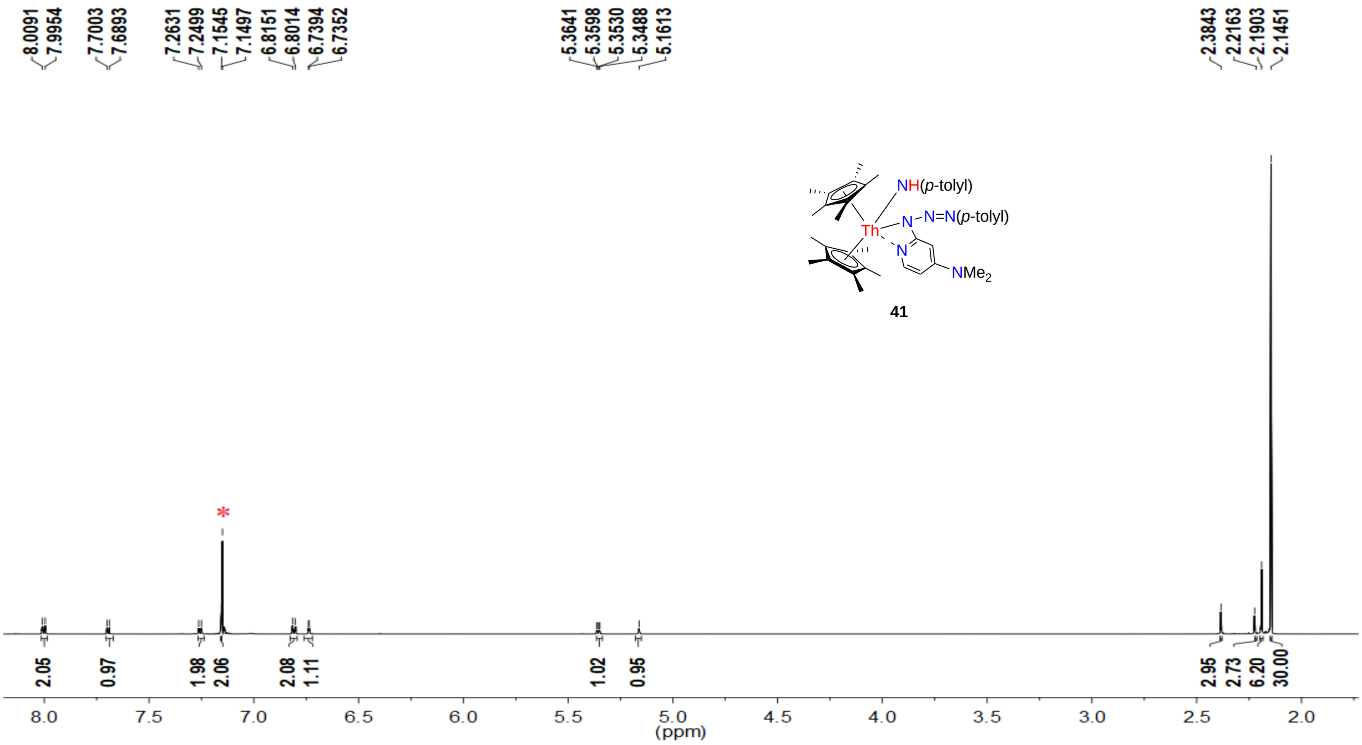

Figure S92. ^{1}H NMR (C_6D_6; 20 ℃) spectrum for compound (η^5-C_5Me_5)$_2$Th[NH(*p*-tolyl)][κ^2-*N*,*N*-2-N(NN-*p*-tolyl)-4-(Me_2N)C_5H_3N] (**41**) (* solvent). ^{1}H NMR (C_6D_6): δ 8.00 (d, J = 8.2 Hz, 2H, phenyl), 7.69 (d, J = 6.6 Hz, 1H, py), 7.26 (d, J = 7.9 Hz, 2H, phenyl), 7.15 (d, J = 7.9 Hz, 2H, phenyl), 6.81 (d, J = 8.2 Hz, 2H, phenyl), 6.74 (d, J = 2.5 Hz, 1H, py), 5.36 (dd, J = 6.6 and 2.5 Hz, 1H, py), 5.16 (s, 1H, N*H*), 2.38 (s, 3H, tolylCH_3), 2.22 (s, 3H, tolylCH_3), 2.19 (s, 6H, N(CH_3)$_2$), 2.15 (s, 30H, CpCH_3) ppm.

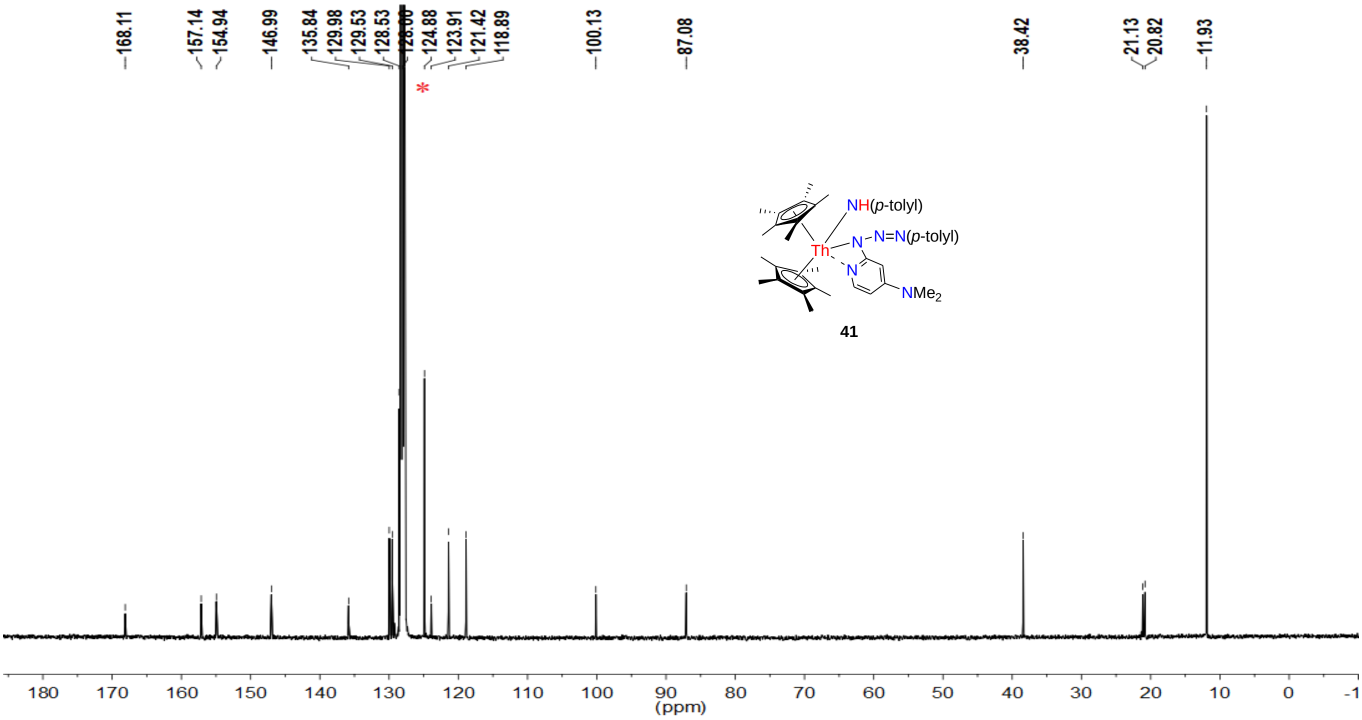

Figure S93. ^{13}C{^{1}H} NMR (C_6D_6; 20 ℃) spectrum for compound (η^5-C_5Me_5)$_2$Th[NH(*p*-tolyl)][κ^2-*N*,*N*-2-N(NN-*p*-tolyl)-4-(Me_2N)C_5H_3N] (**41**) (* solvent). ^{13}C{^{1}H} NMR (C_6D_6): δ 168.1 (py *C*), 157.1 (py *C*), 154.9 (py *C*), 147.0 (phenyl *C*), 135.8 (phenyl *C*), 130.0 (phenyl *C*), 129.5 (phenyl *C*), 128.5 (phenyl *C*), 124.9 (ring *C*), 123.9 (phenyl *C*), 121.4 (phenyl *C*), 118.9 (phenyl *C*), 100.1 (py *C*), 87.1 (py *C*), 38.4 (N*C*H_3), 21.1 (tolyl*C*H_3), 20.8 (tolyl*C*H_3), 11.9 (Cp*C*H_3) ppm.

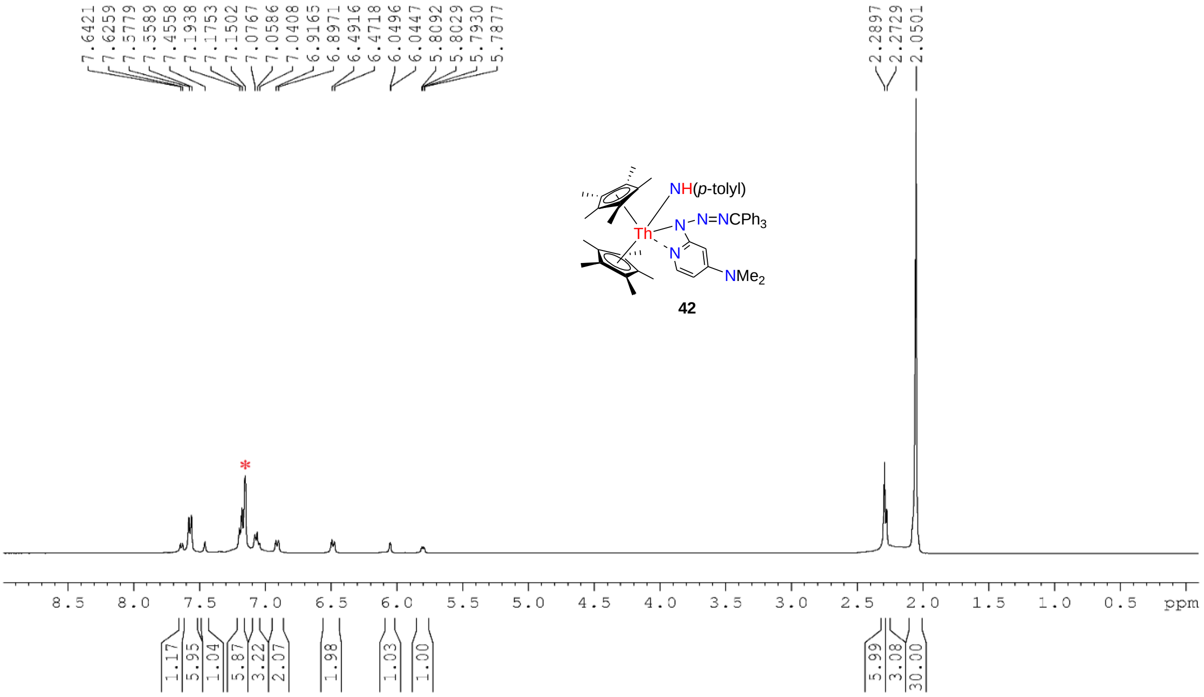

Figure S94. ^{1}H NMR (C_6D_6; 20 ℃) spectrum for compound (η^5-C_5Me_5)$_2$Th[NH(*p*-tolyl)][κ^2-*N*,*N*-2-N(NNCPh$_3$)-4-(Me$_2$N)C$_5$H$_3$N] (**42**) (* solvent). ^{1}H NMR (C_6D_6): δ 7.63 (d, *J* = 6.5 Hz, 1H, py), 7.57 (d, *J* = 7.6 Hz, 6H, phenyl), 7.46 (s, 1H, N*H*), 7.18 (m, 6H, phenyl), 7.06 (m, 3H, phenyl), 6.91 (d, *J* = 7.8 Hz, 2H, phenyl), 6.49 (d, *J* = 7.8 Hz, 2H, phenyl), 6.05 (d, *J* = 2.0 Hz, 1H, py), 5.80 (m, 1H, py), 2.29 (s, 6H, N(C*H*$_3$)$_2$), 2.27 (s, 3H, tolylC*H*$_3$), 2.05 (s, 30H, CpC*H*$_3$) ppm.

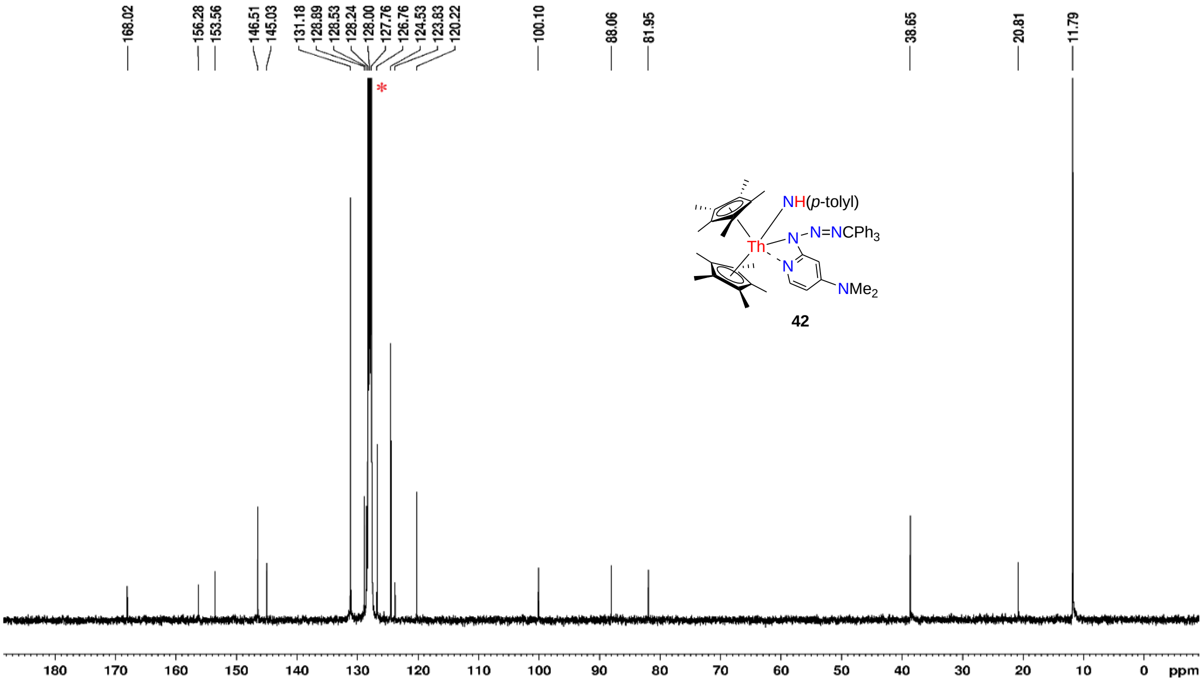

Figure S95. $^{13}C\{^{1}H\}$ NMR (C_6D_6; 20 ℃) spectrum for compound (η^5-C_5Me_5)$_2$Th[NH(*p*-tolyl)][κ^2-*N*,*N*-2-N(NNCPh$_3$)-4-(Me$_2$N)C$_5$H$_3$N] (**42**) (* solvent). $^{13}C\{^{1}H\}$ NMR (C_6D_6): δ 168.0 (py *C*), 156.3 (py *C*), 153.6 (py *C*), 146.5 (phenyl *C*), 145.0 (phenyl *C*), 131.2 (phenyl *C*), 128.9 (phenyl *C*), 128.5 (phenyl *C*), 126.8 (phenyl *C*), 124.5 (ring *C*), 123.8 (phenyl *C*), 120.2 (phenyl *C*), 100.1 (py *C*), 88.1 (py *C*), 82.0 (N*C*Ph$_3$), 38.7 (N*C*H$_3$), 20.8 (tolyl*C*H$_3$), 11.8 (Cp*C*H$_3$) ppm.

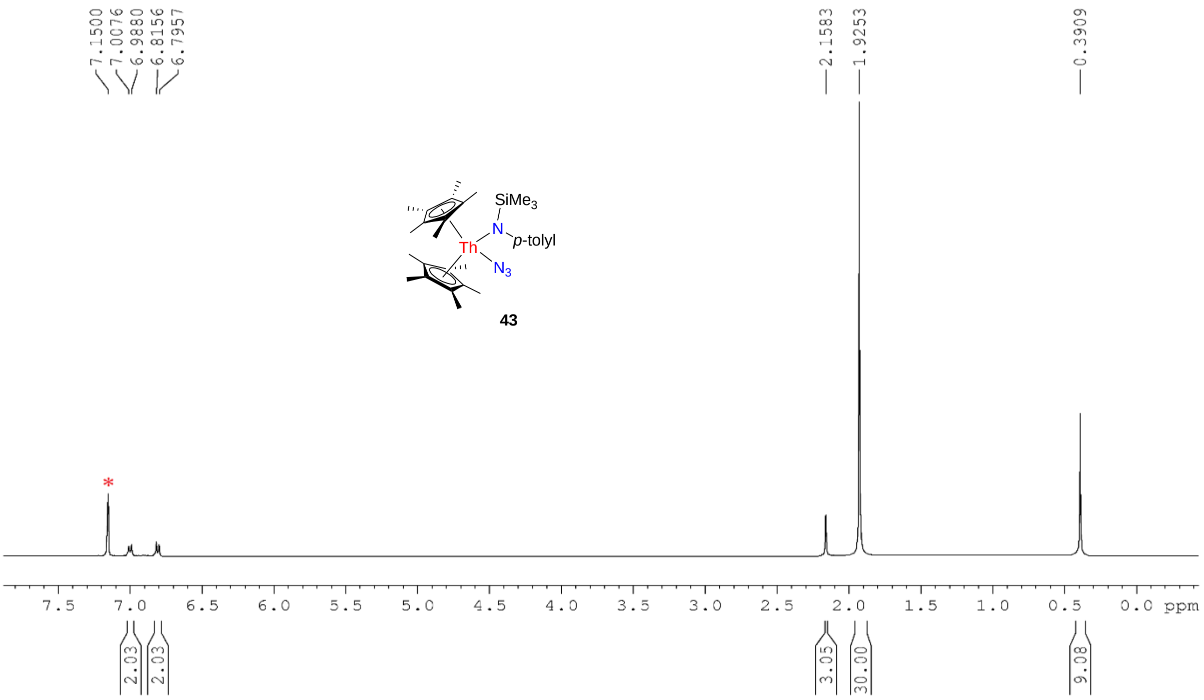

Figure S96. ^{1}H NMR (C_6D_6; 20 ℃) spectrum for compound (η^5-C_5Me_5)$_2$Th(N_3)[N(*p*-tolyl)$SiMe_3$] (**43**) (* solvent). ^{1}H NMR (C_6D_6): δ 7.00 (d, J = 7.8 Hz, 2H, phenyl), 6.80 (d, J = 8.0 Hz, 2H, phenyl), 2.16 (s, 3H, tolylCH_3), 1.93 (s, 30H, CpCH_3), 0.39 (s, 9H, SiCH_3) ppm.

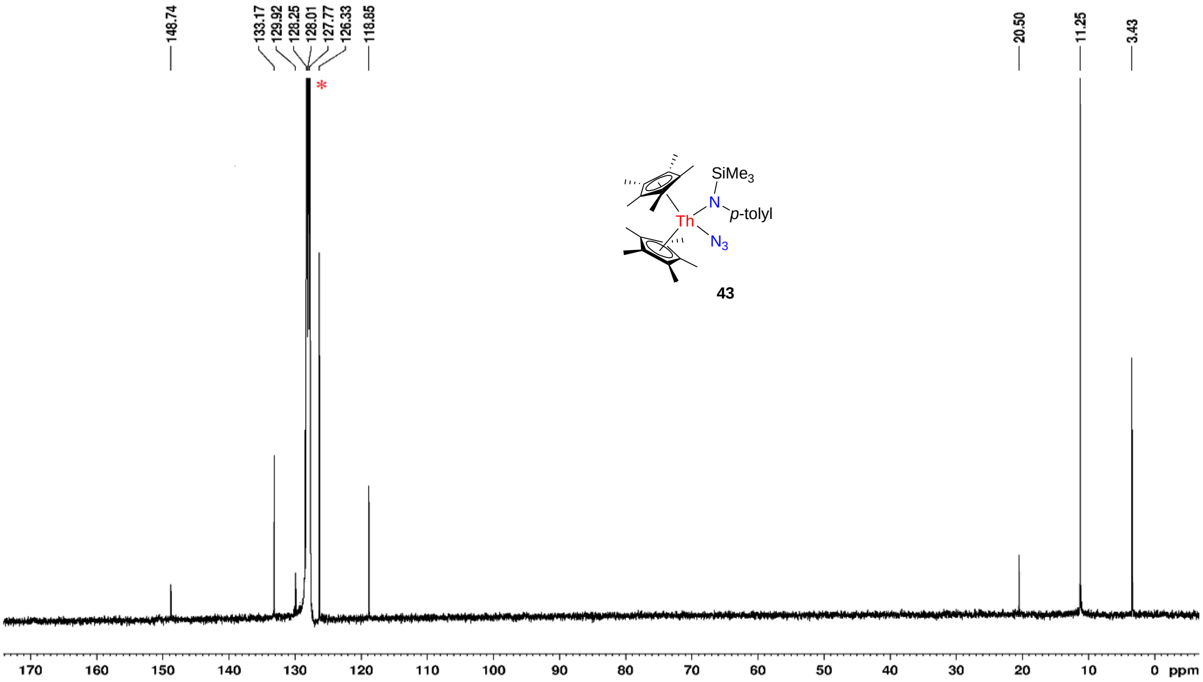

Figure S97. ^{13}C{^{1}H} NMR (C_6D_6; 20 ℃) spectrum for compound (η^5-C_5Me_5)$_2$Th(N_3)[N(*p*-tolyl)$SiMe_3$] (**43**) (* solvent). ^{13}C{^{1}H} NMR (C_6D_6): δ 148.7 (phenyl *C*), 133.2 (phenyl *C*), 129.9 (phenyl *C*), 126.3 (phenyl *C*), 118.9 (ring *C*), 20.5 (tolyl*C*H_3), 11.2 (Cp*C*H_3), 3.4 (Si*C*H_3) ppm.

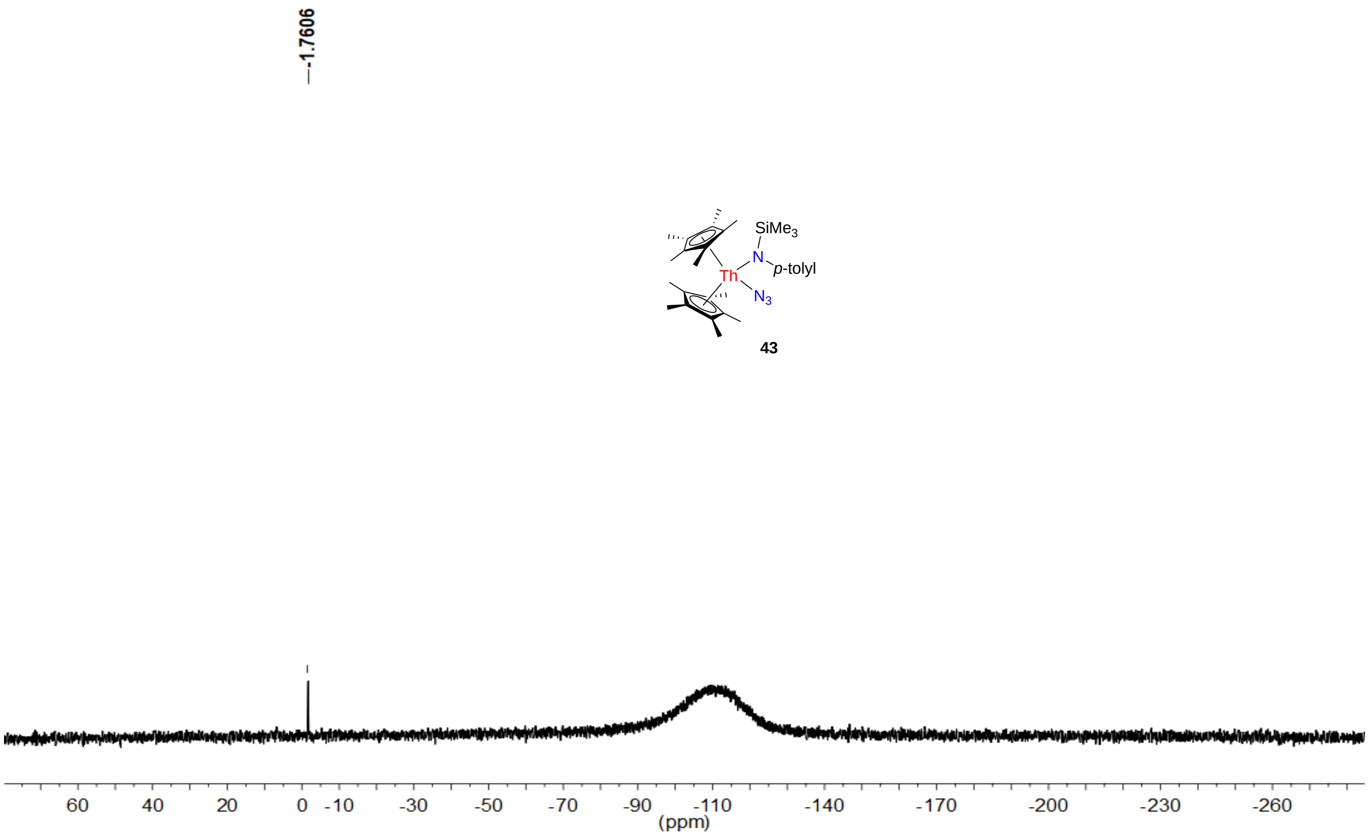

Figure S98. $^{29}Si\{^1H\}$ NMR (C_6D_6; 20 ℃) spectrum for compound (η^5-C_5Me_5)$_2$Th(N_3)[N(*p*-tolyl)$SiMe_3$] (**43**). $^{29}Si\{^1H\}$ NMR (C_6D_6): δ -1.76 ppm.

5. References

(1) Frisch, M. J.; Trucks, G. W.; Schlegel, H. B.; Scuseria, G. E.; Robb, M. A.; Cheeseman, J. R.; Scalmani, G.; Barone, V.; Mennucci, B.; Petersson, G. A.; Nakatsuji, H.; Caricato, M.; Li, X.; Hratchian, H. P.; Izmaylov, A. F.; Bloino, J.; Zheng, G.; Sonnenberg, J. L.; Hada, M.; Ehara, M.; Toyota, K.; Fukuda, R.; Hasegawa, J.; Ishida, M.; Nakajima, T.; Honda, Y.; Kitao, O.; Nakai, H.; Vreven, T.; Montgomery, J. A. Jr.; Peralta, J. E.; Ogliaro, F.; Bearpark, M.; Heyd, J. J.; Brothers, E.; Kudin, K. N.; Staroverov, V. N.; Kobayashi, R.; Normand, J.; Raghavachari, K.; Rendell, A.; Burant, J. C.; Iyengar, S. S.; Tomasi, J.; Cossi, M.; Rega, N.; Millam, J. M.; Klene, M.; Knox, J. E.; Cross, J. B.; Bakken, V.; Adamo, C.; Jaramillo, J.; Gomperts, R.; Stratmann, R. E.; Yazyev, O.; Austin, A. J.; Cammi, R.; Pomelli, C.; Ochterski, J. W.; Martin, R. L.; Morokuma, K.; Zakrzewski, V. G.; Voth, G. A.; Salvador, P.; Dannenberg, J. J.; Dapprich, S.; Daniels, A. D.; Farkas, O.; Foresman, J. B.; Ortiz, J. V.; Cioslowski, J.; Fox, D. J. *Gaussian 09*, Revision A.02, Gaussian, Inc.: Wallingford CT, 2009.

(2) Küchle, W.; Dolg, M.; Stoll, H.; Preuss, H. Energy-adjusted pseudopotentials for the actinides. Parameter sets and test calculations for thorium and thorium monoxide. *J. Chem. Phys.* **1994**, *100*, 7535-7542.

(3) Cao, X.; Dolg, M.; Stoll, H. Valence basis sets for relativistic energy-consistent small-core actinide pseudopotentials. *J. Chem. Phys.* **2003**, *118*, 487-496.

(4) Cao, X.; Dolg, M. Segmented contraction scheme for small-core actinide pseudopotential basis sets. *J. Mol. Struct. (THEOCHEM)* **2004**, *673*, 203-209.
